# Development of an Agent-Based Model (ABM) to Simulate the Immune System and Integration of a Regression Method to Estimate the Key ABM Parameters by Fitting the Experimental Data

**DOI:** 10.1371/journal.pone.0141295

**Published:** 2015-11-04

**Authors:** Xuming Tong, Jinghang Chen, Hongyu Miao, Tingting Li, Le Zhang

**Affiliations:** 1 College of Computer and Information Science, Southwest University, Chongqing, P. R. China; 2 Department of Biostatistics and Computational Biology, Center for Biodefense Immune Modeling, University of Rochester, New York, United States of America; 3 Department of Biostatistics, School of Public Health, University of Texas Houston, Houston, Texas, United States of America; 4 School of Mathematics and Statistics, Southwest University, Chongqing, China; Pennsylvania State University, UNITED STATES

## Abstract

Agent-based models (ABM) and differential equations (DE) are two commonly used methods for immune system simulation. However, it is difficult for ABM to estimate key parameters of the model by incorporating experimental data, whereas the differential equation model is incapable of describing the complicated immune system in detail. To overcome these problems, we developed an integrated ABM regression model (IABMR). It can combine the advantages of ABM and DE by employing ABM to mimic the multi-scale immune system with various phenotypes and types of cells as well as using the input and output of ABM to build up the Loess regression for key parameter estimation. Next, we employed the greedy algorithm to estimate the key parameters of the ABM with respect to the same experimental data set and used ABM to describe a 3D immune system similar to previous studies that employed the DE model. These results indicate that IABMR not only has the potential to simulate the immune system at various scales, phenotypes and cell types, but can also accurately infer the key parameters like DE model. Therefore, this study innovatively developed a complex system development mechanism that could simulate the complicated immune system in detail like ABM and validate the reliability and efficiency of model like DE by fitting the experimental data.

## Introduction

Currently, system biologists employ agent-based models (ABM) [1–5] and differential equation models (DE) [6–9] to simulate the immune system. Detailed definitions of ABM and DE are illustrated in the S1 File.

Recently, researchers did develop several ABMs for the immune system simulation. For example, The Basic Immune Simulator (BIS) [10] is an agent-based model (ABM) that can be used to study the interactions between cells of the innate and adaptive immune systems. The BIS demonstrated that the degree of the initial innate response was a crucial determinant for an appropriate adaptive response [10]. Also, the ImmunoGrid project [11] is to develop a natural-scale model of the human immune system using an ABM, that can reflect both the diversity and the relative proportions of the molecules and cells. This model will be of great value for specific applications in the field of immunology[11].

ABM has several significant advantages. First, its natural representational formalism can be employed to denote a cell’s biological properties and behavior in detail [1]. Second, its flexible features can be employed to reflect the real complex dynamic environment [12]. However, it is difficult for ABM to incorporate experimental data, because ABM describes the system at the level of its constituent units but not at the top level [13].

DE is broadly employed to approximate experimental data and predict the progression of the immune system. For example, researchers have applied it to the case of influenza A virus (IAV) infection. Miao et al., [14] developed a differential equation model to describe the dynamic interactions among the components (i.e., epithelial cells, virus, CD8 CTLs, and antibody) in the lung. The model was used to quantify the immune responses and to estimate the key parameters in primary infection. Not limited to IAV infection, DE can also be widely used for other virus infections, such as HIV in the study of Miao et al. [9]. The researchers developed statistical estimation, model selection, and multi-model averaging methods for in vitro HIV viral fitness experiments using a set of nonlinear ordinary differential equations and addressed the parameter identifiability of the model [9].

The DE has been the focus of a great deal of attention due to its great potential as a new optimization technique to solve complex nonlinear problems and widespread use in various areas [15]. Compared to ABM, DE can be easily employed to solve the optimization problem by estimating a few control parameters [15]. However, it has difficulty describing the details of biological systems because DE falls short in constructing a biological model to a sufficient degree, especially when faced with the simulation of complex phenomena.

To integrate the advantages of these two commonly used models, we developed an integrated ABM regression model (IABMR) and employed the IAV data set [14] to evaluate its efficiency and accuracy. IABMR employed ABM to denote each cell as an agent with three phenotypes (i.e., quiescence, proliferation and apoptosis). Then, it employed Loess regression to build a Loess model based on the input and output of ABM. The model’s key parameters were optimized using the particle swarm optimization algorithm (PSO)[16–21]. The concept of PSO is illustrated in the S1 File.

Next, we employed the classical greedy algorithm [22–24] to optimize the ABM parameter and compare the efficiency of ABM with the greedy algorithm and IABMR. The results demonstrated that IABMR not only described the immune response at the cellular level using various cells’ phenotypes and possessed great potential for investigating interactions and special information for the cells but also overcame the limitations of ABM in parameter estimation.

## Methods

### 2.1. Using ABM to describe the immune system

**Fig 1:**
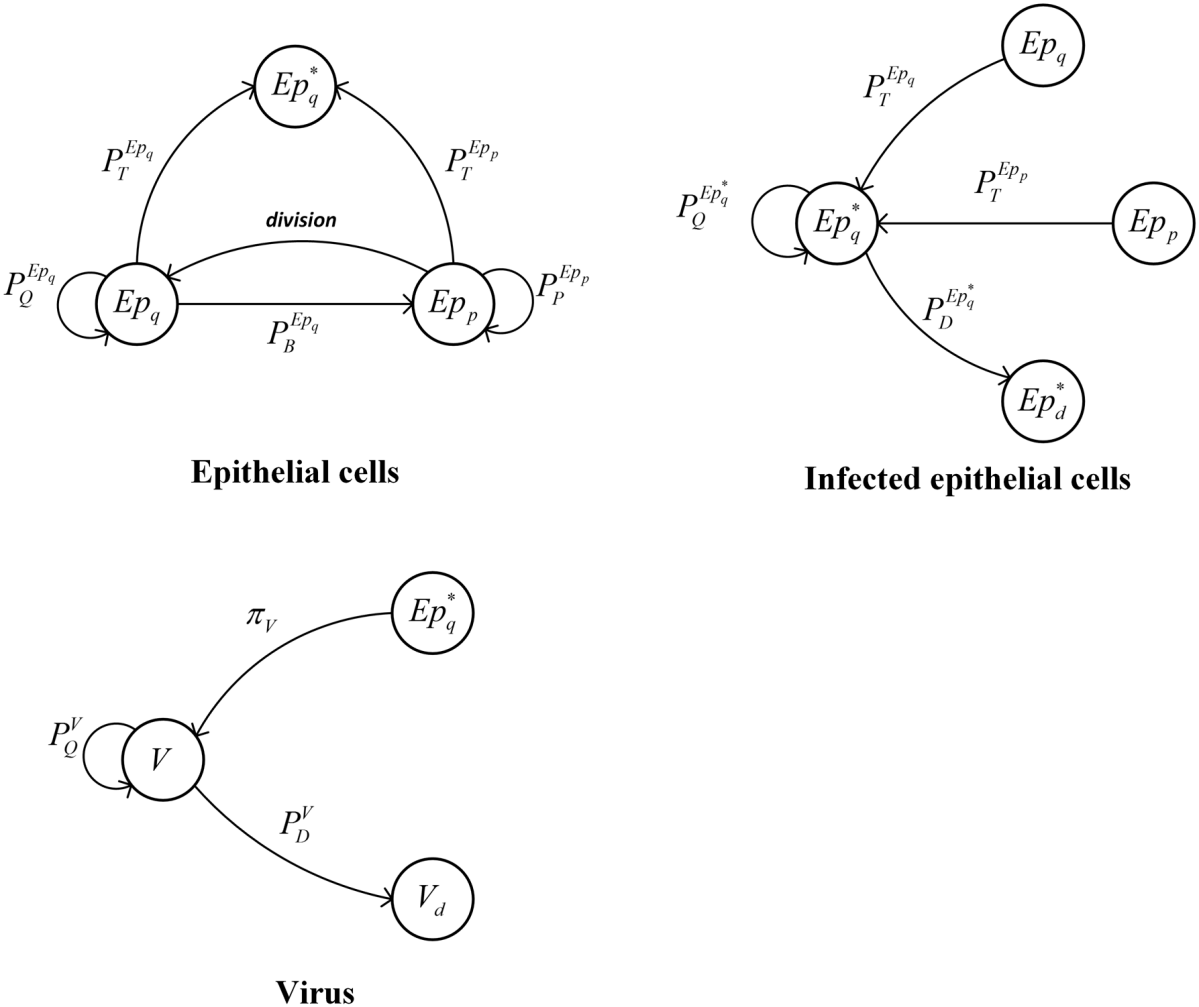
State transition diagrams of epithelial cells, infected epithelial cells and virus.

To describe the dynamic interactions among the components (i.e., epithelial cells, infected epithelial cells and virus) in the lung, Fig 1 is used to quantify immune responses in primary infections.

An epithelial cell in a quiescent state *Ep*_*q*_ can be transited to three other states. Two of these states belong to the *Ep* cell, where $P_{B}^{Ep_{q}}$ and $P_{Q}^{Ep_{q}}$ are the probabilities for *Ep*_*q*_ to change its state. *Ep*_*q*_ and *Ep*_*p*_ are two states of the *Ep* cell. The *Ep*_*q*_ cell can also be differentiated into another type of the cell (Ep*) with a probability $P_{T}^{Ep_{q}}$. Once the *Ep*_*q*_ state transits to the *Ep*_*p*_ state with a probability of $P_{B}^{Ep_{q}}$, it will have $P_{P}^{Ep_{p}}$ and $P_{T}^{Ep_{p}}$ probabilities to become *Ep*_*p*_ and $Ep_{q}^{*}$, respectively.

With respect to the above state transition diagram (Fig 1 **(Epithelial cells)**), the state transition equations for epithelial cells are developed as follows.

$$Ep_{q\left(t\right)}=Ep_{q\left(t-1\right)}P_{Q}^{Ep_{q}}+2Ndiv_{t}^{Ep}-Ep_{q\left(t-1\right)}P_{T}^{Ep_{q}}V_{t-1}-Ep_{q\left(t-1\right)}P_{B}^{Ep_{q}}$$ (1.1)

$$Ep_{p\left(t\right)}=\left(Ep_{p\left(t-1\right)}-Ndiv_{t}^{Ep}\right)P_{p}^{Ep_{p}}+Ep_{q\left(t-1\right)}P_{B}^{Ep_{q}}-Ep_{p\left(t-1\right)}P_{T}^{Ep_{p}}V_{t-1}$$ (1.2)

Here, *V* represents the infective viral titer and $Ndiv_{t}^{Ep}$ is used to represent the number of cells which will divided into two cells. The case of an infected epithelial cell is shown in Fig 1 **(Infected epithelial cells)**.

The $Ep_{q}^{*}$ state can transit to itself and $Ep_{d}^{*}$ with the probability $P_{Q}^{Ep_{q}^{*}}$ and $P_{D}^{Ep_{q}^{*}}$, respectively. The transition equations are described as the following equations.

$$Ep_{q\left(t\right)}^{*}=Ep_{q\left(t-1\right)}^{*}P_{Q}^{Ep_{q}^{*}}+Ep_{q\left(t-1\right)}P_{T}^{Ep_{q}}+Ep_{p\left(t-1\right)}P_{T}^{Ep_{p}}-Ep_{q\left(t-1\right)}^{*}P_{D}^{Ep_{q}^{*}}$$ (2.1)

$$Ep_{d\left(t\right)}^{*}=Ep_{q\left(t-1\right)}^{*}P_{D}^{Ep_{q}^{*}}+Ep_{d\left(t-1\right)}^{*}$$ (2.2)

Different from the epithelial cell and infected epithelial cell, the virus is too small to be described as a discrete variable. In Fig 1 **(Virus)**, the virus is described as a continuous variable with $P_{D}^{V}$ percentage of dying (*V*_*d*_ state) and $P_{Q}^{V}$ percentage of living. Here, we set $P_{D}^{V}+P_{Q}^{V}=1$. Additionally, the virus can be produced by $Ep_{q}^{*}$ with respect to the rate of *π*_*v*_.

The case of the virus can described using the following equations.

$$V_{t}=V_{t-1}P_{Q}^{V}+Ep_{q\left(t-1\right)}^{*}\pi_{v}-V_{t-1}P_{D}^{V}$$ (3.1)

$$V_{d\left(t\right)}=V_{t-1}P_{D}^{V}+V_{d\left(t-1\right)}$$ (3.2)

To simulate the process of cellular immunity among the epithelial cells, virus and infected epithelial cells, an agent based model (ABM) is developed based on the diagrams and equations provided above. The parameters listed in Table 1 agree with the following rules.

**Table 1:** Parameters and variables definitions for agent based model.

Parameter	Definition	Value
$P_{T}^{Ep_{q}}$	Infection rate of *Ep*_*q*_ (*hour* ^−1^)	2.42×10^−7^
$P_{T}^{Ep_{p}}$	Infection rate of *Ep*_*p*_ (*hour* ^−1^)	2.42×10^−7^
$P_{B}^{Ep_{q}}$	Proliferation rate of *Ep*_*q*_ (*hour* ^−1^)	6.2×10^−9^
$P_{Q}^{Ep_{q}}$	Quiescence rate of *Ep*_*q*_ (*hour* ^−1^)	9.999997518×10^−1^
$P_{P}^{Ep_{p}}$	Probability value for *Ep*_*p*_ to stain resting(*hour* ^−1^)	9.999997518×10^−1^
$P_{Q}^{Ep_{q}^{*}}$	Probability value for $Ep_{q}^{*}$ to stain resting(*hour* ^−1^)	9.402×10^−1^
**$P_{D}^{Ep_{q}^{*}}$**	Death rate of $Ep_{q}^{*}$ (*hour* ^−1^)	5.98×10^−2^
*π*_*v*_	Virus productivity of $Ep_{q}^{*}$ (*hour* ^−1^)	1.0×10^1^
$P_{D}^{V}$	Death rate of *V* (*hour* ^−1^)	4.23×10^−1^
$P_{Q}^{V}$	Survival rate of *V* (*hour* ^−1^)	5.77×10^−1^

$$P_{T}^{Ep_{q}}+P_{B}^{Ep_{q}}+P_{Q}^{Ep_{q}}=1$$ (4.1)

$$P_{T}^{Ep_{p}}+P_{P}^{Ep_{p}}=1$$ (4.2)

$$P_{T}^{Ep_{q}}=P_{T}^{Ep_{p}}$$ (4.3)

$$P_{Q}^{Ep_{q}^{*}}+P_{D}^{Ep_{q}^{*}}=1$$ (4.4)

$$P_{Q}^{V}+P_{D}^{V}=1$$ (4.5)

### 2.2. Parameter Estimation

To estimate the parameters in this study, parameter vector space (***H***) is generated by the Sparse Grid method [25], which consists of a set of parameter vectors; each vector has 4 dimensions. The Sparse Grid method always chooses the most important points in the high dimension space to approximate the complicated surface [26–28].

In what follows, the input parameter of ABM is denoted by a four-dimensional vector ***θ***, where the components θ_k_, k = 1,2,3,4 represents ($P_{B}^{Ep_{q}},P_{T}^{Ep_{q}},P_{D}^{Ep_{q}^{*}},P_{D}^{V}$) respectively. Reported by the previous research [14], the input data ***θ*** are estimated as (6.2×10^−9^,2.42×10^−7^,5.98×10^−2^,4.23×10^−1^), which we call as the initial parameter ***θ***_**0**_. In this study, we set the input parameter of ABM in the region (0,2***θ***_**0**_) = {(θ_1_, θ_2_, θ_3_, θ_4_)∈ *R* ^4^,0≤θ_K_≤2θ_0k_,k = 1,2,3,4}. However, according to the rules of the Sparse Grid, each component of parameter vector ***h***∈***H*** is between 0 and 1. Therefore, we need to map the parameter vector space ***H*** generated by Sparse Grid into the region (0,2***θ***_**0**_). The mapping function is:

$$\theta_{1}=\left(b-a\right)h+a$$ (5)

Where ***h*** is a parameter vector in the space ***H***, ***a* = 0, *b* = 2*θ***_**0**_.

***θ***_***1***_ is employed as the input parameters for the ABM to generate *L* sets of output data (*G*_1_), which represents the number of cells in 5 days. To generate randomness for ABM, we performed *Lr* replicates for each set of ***θ***_**1**_. Next, ***θ***_**1**_ and *G*_1_ are employed to develop a Loess regression [29–32] mode *M*_0_.

In our model *M*_0_, the Loess regression is described in Eq 6.

$$\chi^{2}=\Sigma_{i}w{\left(\theta_{1i}-x;g\right)}^{2}{\left(\alpha +\beta \left(\theta_{1i}-x\right)-G_{1i}\right)}^{2}$$ (6)

Here, *w* is a weighting function and ***θ***_**1i**_ is an input parameter of ABM, where *i* denotes the *i*-th sampling point in the parameter vector space. ***θ***_**1i**_ represents the points in the neighborhood of ***x*** to be weighted by *w* depending on the distance to ***x***. *g* is a key parameter in the procedure called the "bandwidth" or "smoothing parameter” that determines how much of the data is used to fit each local polynomial. ***G***_**1i**_ is the output data value of ABM corresponding to the input data ***θ***_**1i**_. *α* and *β* are two coefficients of the least squares method [33] that is employed to approximate their value by minimizing the value of *χ* ^2^ in Eq 6.

Next, the particle swarm optimization algorithm (PSO) [16] is employed to locate the optimal parameter by fitting the real experimental data. PSO [17–21] is illustrated in the S1 File in detail, and its key equations are described by Eqs 7.1 and 7.2.

$$v_{i\left(t+1\right)}=wv_{i\left(t\right)}+c_{1}\cdot rand()\cdot \left(p_{i\left(t\right)}-x_{i\left(t\right)}\right)+c_{2}\cdot Rand()\cdot \left(p_{g\left(t\right)}-x_{i\left(t\right)}\right)$$ (7.1)

$$x_{i\left(t+1\right)}=x_{i}+v_{i\left(t+1\right)}$$ (7.2)

First, let *S* be the number of particles in the swarm. Then initialize the particle's position with a uniformly distributed random vector *x*_*i*_∼*U*(*lb*,*ub*), where *lb* and *ub* are the lower and upper boundaries of the search-space, here (*lb*,*ub*) = (**0**,2***θ***_**0**_). Obviously, *x*_*i*_ can be considered as the input parameter. The particle's initial velocity is: *v*_*i*_∼*U*(−|*ub*−*lb*|,|*ub*−*lb*|). Here, *w* is a weight function used to maintain the inertia force of each particle. Let *p*_*i*_ be the best known position of particle *i* and let *p*_*g*_ be the best known position of the entire swarm. Then, Eq 8 is employed as the object function for the parameter estimation.

$$f_{obj}=\Sigma_{j=1}^{m}\Sigma_{i=1}^{n}{\left(y_{i}-V_{1}\right)}^{2}$$ (8)

Here, *m* is the time point, and *n* is the replicates at each time point, *V*_1_ is the real experimental data in five days. *y*_*i*_ is the predictive value from the Loess model based on input value *x*_*i*_.

By using the PSO algorithm and Loess model, we can minimize the object function *f*_*obj*_ to locate the local optimal parameter ***θ*** * in the region (**0,2*θ***_**0**_).

Next, we reemployed the mapping function (Eq 5) to map parameter vector space ***H*** on region (**0,2*θ*** *) to generate *L* sets of input parameters ***θ***_**2**_ and *n* replicates for each set of ***θ***_**2**_. These data will be employed as input parameters in the ABM; then, we can obtain *G*_2_ output data with *m* time points. Next, ***θ*** * will be employed as the input data of ABM to generate the simulated experimental data set *V*_2_ with *n* replicates, which will replace *V*_1_ by adding random noise.

The normal distribution method (Eqs 9.1 and 9.2) [34] is used to add noise for each replicate of the *V*_2_ data set and develop the simulated experimental data set $V_{2}^{*}$.

$$V_{\Delta }\sim N\left(0,\alpha_{i}\right)\;\;\;\;\;\;\;\;\;\;\;\;\;i=1,2,3.$$ (9.1)

$$V_{2}^{*}=V_{2}+V_{\Delta }$$ (9.2)

*N*(0,*α*_*i*_) denotes a normal distribution with mean 0 and standard deviation *α*_*i*_.

Next, a new Loess regression model *M*_1_ is built based on ***θ***_**2**_ and *G*_2_ in a process similar to *M*_0_. We used PSO [35] to explore the optimal local parameter ***Estθ***_***i***_ by fitting the simulated experimental data $V_{2}^{*}$. Finally, we can compute average relative error (ARE) [9] for each ***Estθ***_***i***_ using Eq 10.

$$ARE=\Sigma_{i=1}^{M}\frac{\left|Est\theta_{i}-\theta^{*}\right|}{M\times \left|\theta^{*}\right|}\times 100\%$$ (10)

**Fig 2:**
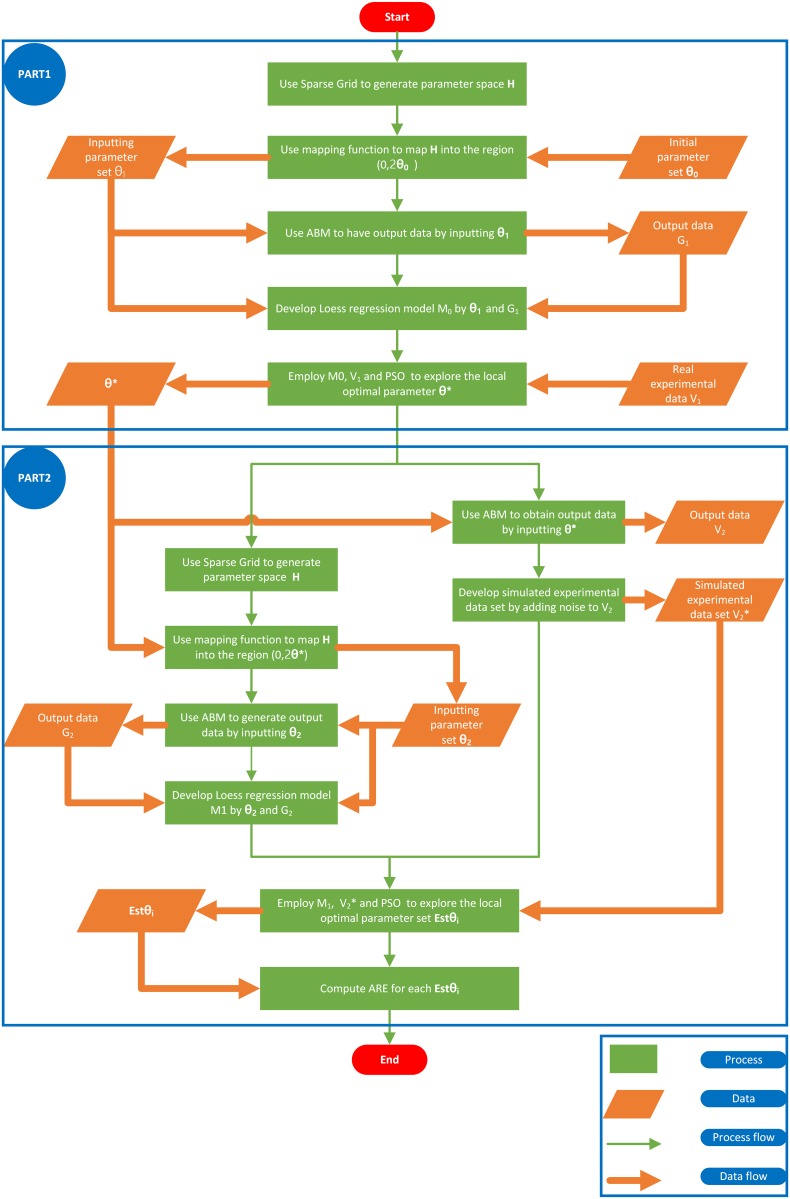
Parameter estimation process.

Here, *M* is the total number of ABM simulation runs for each sample. This parameter estimation process is illustrated in Fig 2.

## Results

The IABMR model is developed using C++ and R program language and works in the Linux environment.

### 3.1. Primary data for model fitting

We used real experimental data *V*_1_[14] from infection of mice with the H3N2 influenza virus A/X31 strain to fit the model. This study employs data from the initial preadaptive phase constituting 0 to 5 days post-infection. The real experimental data contains 6 samples and each sample has 13 time points. The detailed experimental data information is listed in Table 2. The initial key parameters of ABM are also from the literature [14].

**Table 2:** Real experimental data between 0 to 5 days.

	Time points (day^−1^)												
samples	0	0.125	0.25	0.5	1	1.5	2	2.5	3	3.5	4	4.5	5
1	4.25	2.5	3.5	4.25	5.5	6.5	6.33	6.75	6.5	6.5	6.5	7	6.33
2	3.75	2.5	4.75	3.25	6.75	6.75	7.5	3.5	7.33	7.25	6.25	6.5	5.5
3	4.25	3.5	4.75	5.25	6.5	7.75	7.75	7.5	7.33	7.25	6.5	6.25	5.75
4	3.75	3.5	4.13	5.75	7.25	NA	7.25	6.5	6.25	5.5	NA	NA	NA
5	4.55	2.75	2.5	5.75	NA	NA	NA	7.5	6.75	6.5	NA	NA	NA
6	4.25	NA	4.75	5.5	NA	NA	NA	NA	7.25	5.75	NA	NA	NA

### 3.2. Obtain the sampling data using Sparse Grid function

We employed the “createIntegrationGrid” function of the R “SparseGrid” package to create three sampling data sets in the region (**0**, **1**) (sample size: 41, 137 and 385) (listed in S1–S3 Tables). Then, these sampling data are mapped to the input parameters sets of ABM (***θ***_**1**_ **)** by Eq 5. The values of ***θ***_**1**_ are listed in S4–S6 Tables.

### 3.3. Estimate the parameter of ABM by fitting the real experimental data

To obtain randomness, we run data sample 41, 137 and 385 with 9,9 and 6 times. And then, we denote them as model 41×9, 137×9 and 385×6, respectively. The output data set *G*_1_ (S7–S9 Tables) of ABM is obtained by inputting ***θ***_**1**_. Eqs 7.1 and 7.2 is employed to explore the local optimum parameter θ* for each sampling data set listed in Table 3.

**Table 3:** Optimum parameter for each sampling model.

	optimum parameter (θ*) for each model			
Sampling model	$P_{B}^{Ep_{q}}$	$P_{T}^{Ep_{q}}$	$P_{D}^{Ep_{q}^{*}}$	$P_{D}^{V}$
41×9	0.000000006200000	0.00000005454770	0.01372567	0.7506544
137×9	0.000000006170504	0.00000003664683	0.01600081	0.6807255
385×6	0.000000006146936	0.00000004597134	0.01651409	0.6676635

### 3.4. Generate the simulated experimental data by ABM

We can obtain an output of ABM *V*_2_ by inputting θ*. The simulated experimental data $V_{2}^{*}$ is developed from *V*_2_ by Eq 9 by adding three levels of noise (*α*_*i*_), such as $\sqrt{0.75}$,$\sqrt{1.50}$ and $\sqrt{3.00}$ regarding to our previous study [36]. Part of the simulated experimental data is listed in S10–S12 Tables.

### 3.5. Average relative error computing

After fitting the model to the simulated experimental data using Eqs 7.1 and 7.2, we obtain the local optimal parameter ***Estθ***_***i***_. Then, Eq 10 is employed to compute the average relative error for each set of simulated experimental data. Here, we set the total number of ABM simulation runs as *M* = 100 and the three sample sizes as 5×3 (5 is time points (*m*), 3 is the replicates (*n*)),10×6 and 15×9. The values of ARE for each sample size are listed in Tables 4–6.

**Table 4:** The summary table of ARE values for model 41×9.

Sample size (m×n)		$ARE=\Sigma_{i=1}^{M}\frac{\left|Est\theta_{i}-\theta^{*}\right|}{M\times \left|\theta^{*}\right|}\times 100\%$			
*m*:time point *n*:replicates	Noise level	$P_{B}^{Ep_{q}}$	$P_{T}^{Ep_{q}}$	$P_{D}^{Ep_{q}^{*}}$	$P_{D}^{V}$
5×3	$\sqrt{0.75}$	0.396374135606522	0.443644533769457	0.056352115904734	0.443648916301695
10×6	$\sqrt{0.75}$	0.397479215621607	0.288728219873243	0.056352115904734	0.288729750509077
15×9	$\sqrt{0.75}$	0.396050197041251	0.288728219873243	0.056352115904734	0.288729750509077
5×3	$\sqrt{1.50}$	0.401597828194687	0.133811905977029	0.056352115904734	0.443648916301695
10×6	$\sqrt{1.50}$	0.355114873293543	0.211270062925136	0.056352115904734	0.443648916301695
15×9	$\sqrt{1.50}$	0.303420318533482	0.288728219873243	0.056352115904734	0.443648916301695
5×3	$\sqrt{3.00}$	0.056351812916928	0.095082827502976	0.056352115904734	0.169609252886410
10×6	$\sqrt{3.00}$	0.056351812916928	0.133811905977029	0.056352115904734	0.120094487389536
15×9	$\sqrt{3.00}$	0.056351812916928	0.095082827502976	0.056352115904734	0.155694584660783

**Table 5:** The summary table of ARE values for model 137×9.

Sample size (m×n)		$ARE=\Sigma_{i=1}^{M}\frac{\left|Est\theta_{i}-\theta^{*}\right|}{M\times \left|\theta^{*}\right|}\times 100\%$			
*m*:time point *n*:replicates	Noise level	$P_{B}^{Ep_{q}}$	$P_{T}^{Ep_{q}}$	$P_{D}^{Ep_{q}^{*}}$	$P_{D}^{V}$
5×3	$\sqrt{0.75}$	0.643522575254619	0.930561319026966	0.399535317487816	0.930566159038856
10×6	$\sqrt{0.75}$	0.544230567172763	0.356479937741299	0.087188402282124	0.912747140886330
15×9	$\sqrt{0.75}$	0.370431446243038	0.579838310802485	0.069451181484305	0.686363123221724
5×3	$\sqrt{1.50}$	0.920087152935404	0.777707452418573	0.319657912364586	0.930566159038856
10×6	$\sqrt{1.50}$	0.678690815711458	0.643520628384133	0.165464682512184	0.930566159038856
15×9	$\sqrt{1.50}$	0.356478932929542	0.513177041198269	0.069451181484305	0.376377320727785
5×3	$\sqrt{3.00}$	0.643522575254619	0.930561319026966	0.494169438477352	0.930566159038856
10×6	$\sqrt{3.00}$	0.497055974244236	0.643520628384133	0.107579631811147	0.922833014750482
15×9	$\sqrt{3.00}$	0.426123861132435	0.235982026649789	0.147703266116924	0.643522053012952

**Table 6:** The summary table of ARE values for model 385×6.

Sample size (m×n)		$ARE=\Sigma_{i=1}^{M}\frac{\left|Est\theta_{i}-\theta^{*}\right|}{M\times \left|\theta^{*}\right|}\times 100\%$			
*m*:time point *n*:replicates	Noise level	$P_{B}^{Ep_{q}}$	$P_{T}^{Ep_{q}}$	$P_{D}^{Ep_{q}^{*}}$	$P_{D}^{V}$
5×3	$\sqrt{0.75}$	0.558195074749436	0.377709938409453	0.734336363674898	0.467353749306350
10×6	$\sqrt{0.75}$	0.550373063913468	0.281480374511598	0.646693956494121	0.428673366149265
15×9	$\sqrt{0.75}$	0.317992736543865	0.259980152851755	0.474146550006691	0.215504157408635
5×3	$\sqrt{1.50}$	0.640824859734996	0.421591800456545	0.820570179767702	0.578291010366749
10×6	$\sqrt{1.50}$	0.563205571035716	0.329446998934554	0.686081473456909	0.497756369788074
15×9	$\sqrt{1.50}$	0.545574901056396	0.296157823548324	0.551033947374636	0.382253155968538
5×3	$\sqrt{3.00}$	0.734766947305129	0.497921805194280	0.821690326260787	0.587863332352300
10×6	$\sqrt{3.00}$	0.606827857000626	0.425248426519653	0.762389535239302	0.543190334652111
15×9	$\sqrt{3.00}$	0.568049903236344	0.415941323441953	0.563435357322141	0.407836342708565

### 3.6. Evaluate the accuracy and efficiency of the IABMR model

**Fig 3:**
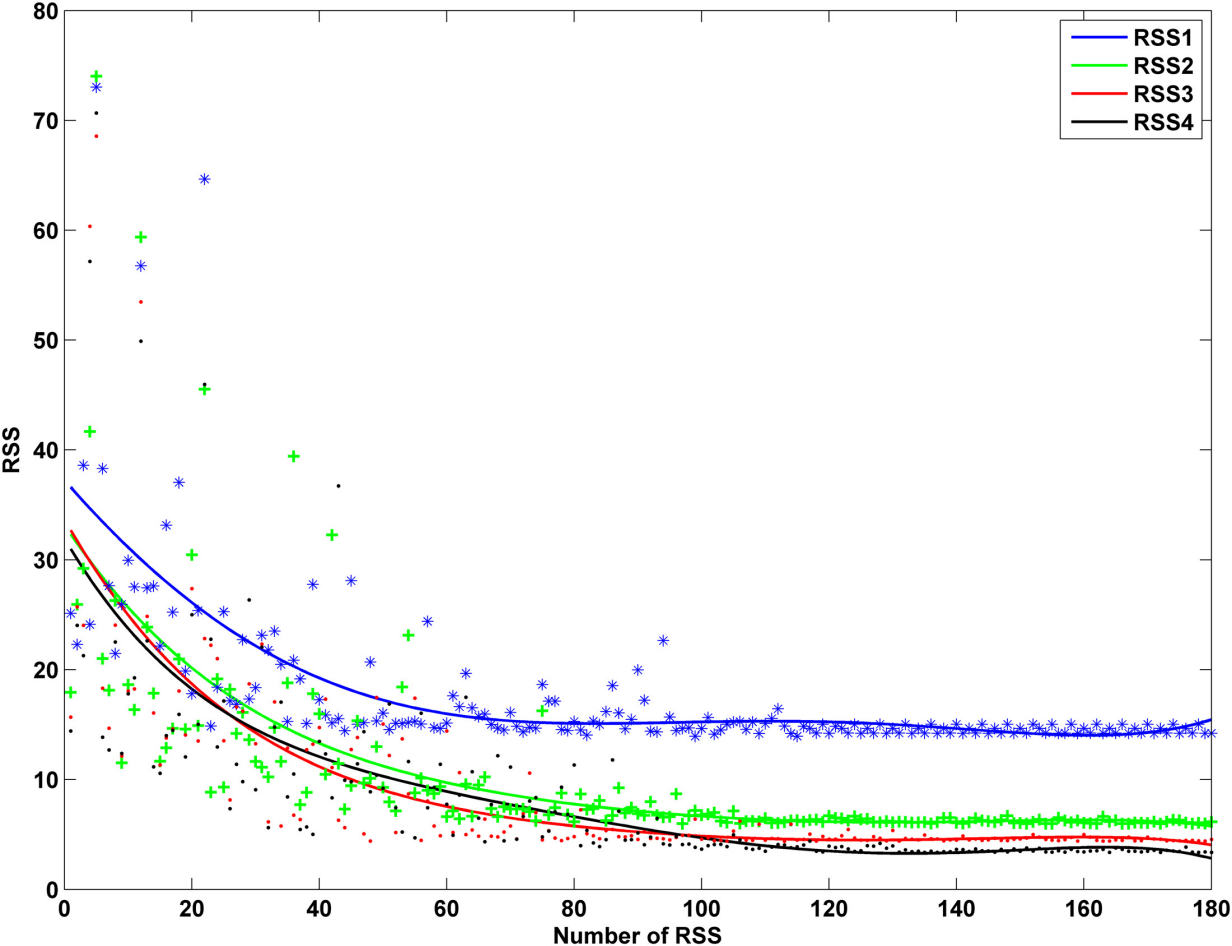
Comparison among RSS1, RSS2, RSS3 and RSS4.

To evaluate the accuracy and efficiency of the IABMR model in parameter estimation, we employed the greedy algorithm [22,37] with ABM to estimate the parameters. Fig 3 compares their residual errors (RSS). Here, RSS1 is the residual errors of the greedy algorithm as well as RSS2, RSS3 and RSS4 are the residual errors of the three sampling data sets from IABMR (model 41×9,137×9 and 385×6).

### 3.7. Using IABMR to approximate primary data

**Fig 4:**
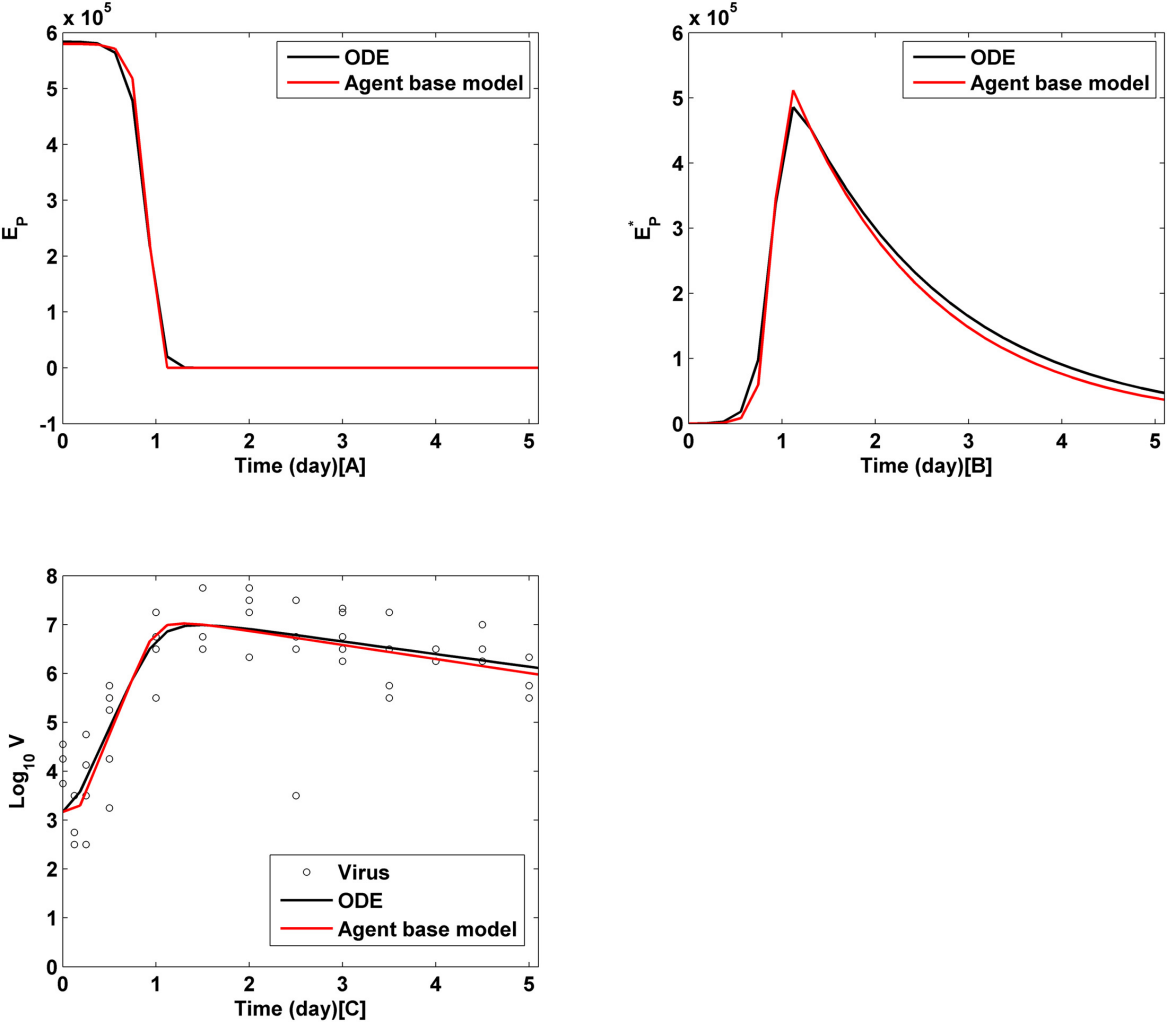
Comparison between IABMR and ODE.

Fig 4 illustrates that IABMR can approximate the primary data with a similar effect as the ODE model [14].

## Discussion

In this work, we developed an agent-based model (ABM) to simulate influenza A virus (IAV) infection and integrated the ABM with Loess regression to develop an integrated ABM regression model (IABMR). This model can be employed to locate the key ABM parameter by fitting the real experimental data.

By inheriting the advantages of ABM, IABMR is capable of mimicking the biological system in detail. Here, IABMR not only showed quantitative changes in the system but also simulated the phenotypic switch for each cell type. Compared to the previous well-developed ODE model [14], it was possible to describe a multi-scale biological system in a very complicated external environment. IABMR Integrated with Loess regression [29] can employ classical numerical optimization methods such as the genetic algorithm [38,39] to estimate key parameter of the model, which is much faster than the greedy algorithm [22–24] used by ABM. These two theoretical advantages made IABMR an attractive application to simulate biological systems, compared to the ODE and ABM.

The average relative error (ARE) is commonly employed to evaluate the capacity of parameter estimation for statistical models. The smaller the ARE, the better the model’s performance. Tables 4–6 showed the ARE values of four key probabilities of the IABMR under the control of the following two aspects: the number of time points collected from the preadaptive phase and the level of noise added to the simulated experimental data.

Table 6 showed two trends of ARE under different noise levels and numbers of time points. First, the ARE values decrease when the number of time points increases from 5 to 15 at the same noise level. For example, the ARE value of $P_{B}^{Ep_{q}}$ has the order $P_{B}^{Ep_{q}}\left(5\times 3\right)>P_{B}^{Ep_{q}}\left(10\times 6\right)>P_{B}^{Ep_{q}}\left(15\times 9\right)$ under noise $\sqrt{0.75}$, which indicates more time points and replicates can obtain better parameter estimation accuracy.

Second, the ARE values increase when the noise level increases from $\sqrt{0.75}$ to $\sqrt{3.00}$ under the same number of time points, which demonstrates that the parameter estimation accuracy is higher with a smaller noise level. For instance, in the case of sample 5×3 (Table 6), the ARE value of $P_{B}^{Ep_{q}}$ has the order:

$P_{B}^{Ep_{q}}\left(\sqrt{0.75}\right)<P_{B}^{Ep_{q}}\left(\sqrt{1.50}\right)<P_{B}^{Ep_{q}}\left(\sqrt{3.00}\right)$ The additional three probabilities ($P_{T}^{Ep_{q}},P_{D}^{Ep_{q}^{*}}\;and\;P_{D}^{V}$) in the parameter have similar trends to $P_{B}^{Ep_{q}}$ (Table 6).

Fig 3 compared the accuracy and parameter estimation speed between the IABMR and ABM models. IABMR is much faster than ABM in terms of locating key parameter. For example, it takes at least 54,600 runs for ABM with the greedy algorithm to make the RSS converge, but only 2310 runs for IABMR with the largest size of parameter space to make the RSS converge. Additionally, the size of the parameter vector space has high impact on the parameter estimation accuracy. The larger the size, the more accurate the estimated results. As described by the Fig 3, model 41×9 has the greatest RSS and model 385×6 has the least RSS. Meanwhile, the trends of the ARE values in Tables 4 and 5 are not as perfect as in Table 6. Lastly, Fig 4 demonstrated that the IABMR simulation results had high similarity like the ODE to approximate the real experiential data, which validated the efficiency and accuracy of the IABMR.

In conclusion, this study developed an IABMR method to simulate detailed biological systems and locate their key parameter using classical numerical optimization methods. By integrating the advantages of both the ABM and ODE modes, it not only described the complicated microenvironment of the biological system and the cell’s behavior in multiple scales in detail, but also easily to incorporate real experimental data. To evaluate the efficiency and accuracy of IABMR, we employed primary influenza infection data as the case study to exhibit the advantages of the IABMR. The validation results demonstrated that IABMR could mimic the immune system on multiple levels similar to ABM and approximate real experimental data similar to ODE with a reasonable parameter estimation cost.

## Supporting Information

S1 File The introduction of ABM, DE and PSO. (PDF)

S1 Table Sample size 41 generated by Sparse Grid. (PDF)

S2 Table Sample size 137 generated by Sparse Grid. (PDF)

S3 Table Sample size 385 generated by Sparse Grid. (PDF)

S4 Table Input data of ABM mapped from sample size 41. (PDF)

S5 Table Input data of ABM mapped from sample size 137. (PDF)

S6 Table Input data of ABM mapped from sample size 385. (PDF)

S7 Table Output data sets of model 41×9. (PDF)

S8 Table Output data sets of model 137×9. (PDF)

S9 Table Output data sets of model 385×6. (PDF)

S10 TableSample size 5×3 with noise $\sqrt{0.75}$.(PDF)

S11 TableSample size 5×3 with noise $\sqrt{1.50}$.(PDF)

S12 TableSample size 5×3 with noise $\sqrt{3.00}$.(PDF)
