# Correction: Development of an Agent-Based Model (ABM) to Simulate the Immune System and Integration of a Regression Method to Estimate the Key ABM Parameters by Fitting the Experimental Data

**DOI:** 10.1371/journal.pone.0156823

**Published:** 2016-05-31

**Authors:** Xuming Tong, Jinghang Chen, Hongyu Miao, Tingting Li, Le Zhang

There is an error in the byline of the article. Dr. Tingting Li is not the Corresponding Author and is solely a co-author. Please direct all correspondence to Dr. Jinghang Chen and Dr. Le Zhang.
