# Supplementary material for: Development of an Agent-Based Model (ABM) to Simulate the Immune System and Integration of a Regression Method to Estimate the Key ABM Parameters by Fitting the Experimental Data
Source: PLoS One. 2015 Nov 4;10(11):e0141295. doi: 10.1371/journal.pone.0141295 (PMC4633145; doi:10.1371/journal.pone.0141295)
Supplement: S1 File — (PDF) [file pone.0141295.s001.pdf]

S1 File. The introduction of ABM, DE and PSO.

1. Agent-based modeling (ABM)

Agent-based model (ABM) is a computational technique model for simulating the actions and interactions of autonomous individuals named as agents in a complex system. Each agent is employed with a set of designed rules, so that it can individually execute a series of operations and make decisions just as described in detail previously [1]. It has been widely used in biology, including human immune system [2], the model of brain tumors [3] and even for developing decision support systems such as for breast cancer [4].

2. differential equation (DE)

A differential equation (DE) is a mathematical equation that relates some function with its derivatives. In applications, the functions usually represent physical quantities, the derivatives represent their rates of change, and the equation defines a relationship between the two [5]. Because such relations are extremely common, differential equations play a prominent role in biology and have long been used to investigate viral dynamics and immune responses to viral infections [6].

3. particle swarm optimization algorithm (PSO)

PSO algorithm works by having a population (called a swarm) of candidate solutions (called particles) [7]. These particles are moved around in the search-space according to a few simple formulae. The movements of the particles are guided by their own best known position in the search-space as well as the entire swarm's best known position. When improved positions are being discovered these will then come to guide

the movements of the swarm [8, 9].

References

1. Zhang L, Wang Z, Sagotsky JA, Deisboeck TS (2009) Multiscale agent-based cancer modeling. Journal of mathematical biology 58: 545-559.

2. Jacob C, Litorco J, Lee L (2004) Immunity through swarms: Agent-based simulations of the human immune system. Artificial Immune Systems: Springer. pp. 400-412.

3. Mansury Y, Diggory M, Deisboeck TS (2006) Evolutionary game theory in an agent-based brain tumor model: exploring the 'genotype–phenotype'link. Journal of theoretical biology 238: 146-156.

4. Siddiqa A, Niazi M, Mustafa F, Bokhari H, Hussain A, et al. A new hybrid agent-based modeling & simulation decision support system for breast cancer data analysis; 2009. IEEE. pp. 134-139.

5. Jones DS, Plank M, Sleeman BD (2011) Differential equations and mathematical biology: CRC press.

6. Miao H, Xia X, Perelson AS, Wu H (2011) On identifiability of nonlinear ODE models and applications in viral dynamics. SIAM review 53: 3-39.

7. Kennedy J, Kennedy JF, Eberhart RC (2001) Swarm intelligence: Morgan Kaufmann.

8. Poli R (2008) Analysis of the publications on the applications of particle swarm optimisation. Journal of Artificial Evolution and Applications 2008: 3.

9. Pedersen MEH, Chipperfield AJ (2010) Simplifying particle swarm optimization. Applied Soft Computing 10: 618-628.
