# Supplementary material for: Development of an Agent-Based Model (ABM) to Simulate the Immune System and Integration of a Regression Method to Estimate the Key ABM Parameters by Fitting the Experimental Data
Source: PLoS One. 2015 Nov 4;10(11):e0141295. doi: 10.1371/journal.pone.0141295 (PMC4633145; doi:10.1371/journal.pone.0141295)
Supplement: S1 Table — (PDF) [file pone.0141295.s002.pdf]

S1 Table. Sample size 41 genetated by Sparse Grid

samples	P1	P2	P3	P4
1	0.112702	0.5	0.5	0.5
2	0.211325	0.211325	0.5	0.5
3	0.211325	0.5	0.211325	0.5
4	0.211325	0.5	0.5	0.211325
5	0.211325	0.5	0.5	0.5
6	0.211325	0.5	0.5	0.788675
7	0.211325	0.5	0.788675	0.5
8	0.211325	0.788675	0.5	0.5
9	0.5	0.112702	0.5	0.5
10	0.5	0.211325	0.211325	0.5
11	0.5	0.211325	0.5	0.211325
12	0.5	0.211325	0.5	0.5
13	0.5	0.211325	0.5	0.788675
14	0.5	0.211325	0.788675	0.5
15	0.5	0.5	0.112702	0.5
16	0.5	0.5	0.211325	0.211325
17	0.5	0.5	0.211325	0.5
18	0.5	0.5	0.211325	0.788675
19	0.5	0.5	0.5	0.112702
20	0.5	0.5	0.5	0.211325
21	0.5	0.5	0.5	0.5
22	0.5	0.5	0.5	0.788675
23	0.5	0.5	0.5	0.887298
24	0.5	0.5	0.788675	0.211325
25	0.5	0.5	0.788675	0.5
26	0.5	0.5	0.788675	0.788675
27	0.5	0.5	0.887298	0.5
28	0.5	0.788675	0.211325	0.5
29	0.5	0.788675	0.5	0.211325
30	0.5	0.788675	0.5	0.5
31	0.5	0.788675	0.5	0.788675
32	0.5	0.788675	0.788675	0.5
33	0.5	0.887298	0.5	0.5
34	0.788675	0.211325	0.5	0.5
35	0.788675	0.5	0.211325	0.5
36	0.788675	0.5	0.5	0.211325
37	0.788675	0.5	0.5	0.5
38	0.788675	0.5	0.5	0.788675
39	0.788675	0.5	0.788675	0.5
40	0.788675	0.788675	0.5	0.5
41	0.887298	0.5	0.5	0.5
