# Supplementary material for: Development of an Agent-Based Model (ABM) to Simulate the Immune System and Integration of a Regression Method to Estimate the Key ABM Parameters by Fitting the Experimental Data
Source: PLoS One. 2015 Nov 4;10(11):e0141295. doi: 10.1371/journal.pone.0141295 (PMC4633145; doi:10.1371/journal.pone.0141295)
Supplement: S2 Table — (PDF) [file pone.0141295.s003.pdf]

S2 Table.Sample size 137 genetated by Sparse Grid

samples	P1	P2	P3	P4
1	0.069432	0.5	0.5	0.5
2	0.112702	0.211325	0.5	0.5
3	0.112702	0.5	0.211325	0.5
4	0.112702	0.5	0.5	0.211325
5	0.112702	0.5	0.5	0.5
6	0.112702	0.5	0.5	0.788675
7	0.112702	0.5	0.788675	0.5
8	0.112702	0.788675	0.5	0.5
9	0.211325	0.112702	0.5	0.5
10	0.211325	0.211325	0.211325	0.5
11	0.211325	0.211325	0.5	0.211325
12	0.211325	0.211325	0.5	0.5
13	0.211325	0.211325	0.5	0.788675
14	0.211325	0.211325	0.788675	0.5
15	0.211325	0.5	0.112702	0.5
16	0.211325	0.5	0.211325	0.211325
17	0.211325	0.5	0.211325	0.5
18	0.211325	0.5	0.211325	0.788675
19	0.211325	0.5	0.5	0.112702
20	0.211325	0.5	0.5	0.211325
21	0.211325	0.5	0.5	0.5
22	0.211325	0.5	0.5	0.788675
23	0.211325	0.5	0.5	0.887298
24	0.211325	0.5	0.788675	0.211325
25	0.211325	0.5	0.788675	0.5
26	0.211325	0.5	0.788675	0.788675
27	0.211325	0.5	0.887298	0.5
28	0.211325	0.788675	0.211325	0.5
29	0.211325	0.788675	0.5	0.211325
30	0.211325	0.788675	0.5	0.5
31	0.211325	0.788675	0.5	0.788675
32	0.211325	0.788675	0.788675	0.5
33	0.211325	0.887298	0.5	0.5
34	0.330009	0.5	0.5	0.5
35	0.5	0.069432	0.5	0.5
36	0.5	0.112702	0.211325	0.5
37	0.5	0.112702	0.5	0.211325
38	0.5	0.112702	0.5	0.5
39	0.5	0.112702	0.5	0.788675
40	0.5	0.112702	0.788675	0.5
41	0.5	0.211325	0.112702	0.5
42	0.5	0.211325	0.211325	0.211325
43	0.5	0.211325	0.211325	0.5
44	0.5	0.211325	0.211325	0.788675
45	0.5	0.211325	0.5	0.112702
46	0.5	0.211325	0.5	0.211325
47	0.5	0.211325	0.5	0.5
48	0.5	0.211325	0.5	0.788675

49	0.5	0.211325	0.5	0.887298
50	0.5	0.211325	0.788675	0.211325
51	0.5	0.211325	0.788675	0.5
52	0.5	0.211325	0.788675	0.788675
53	0.5	0.211325	0.887298	0.5
54	0.5	0.330009	0.5	0.5
55	0.5	0.5	0.069432	0.5
56	0.5	0.5	0.112702	0.211325
57	0.5	0.5	0.112702	0.5
58	0.5	0.5	0.112702	0.788675
59	0.5	0.5	0.211325	0.112702
60	0.5	0.5	0.211325	0.211325
61	0.5	0.5	0.211325	0.5
62	0.5	0.5	0.211325	0.788675
63	0.5	0.5	0.211325	0.887298
64	0.5	0.5	0.330009	0.5
65	0.5	0.5	0.5	0.069432
66	0.5	0.5	0.5	0.112702
67	0.5	0.5	0.5	0.211325
68	0.5	0.5	0.5	0.330009
69	0.5	0.5	0.5	0.5
70	0.5	0.5	0.5	0.669991
71	0.5	0.5	0.5	0.788675
72	0.5	0.5	0.5	0.887298
73	0.5	0.5	0.5	0.930568
74	0.5	0.5	0.669991	0.5
75	0.5	0.5	0.788675	0.112702
76	0.5	0.5	0.788675	0.211325
77	0.5	0.5	0.788675	0.5
78	0.5	0.5	0.788675	0.788675
79	0.5	0.5	0.788675	0.887298
80	0.5	0.5	0.887298	0.211325
81	0.5	0.5	0.887298	0.5
82	0.5	0.5	0.887298	0.788675
83	0.5	0.5	0.930568	0.5
84	0.5	0.669991	0.5	0.5
85	0.5	0.788675	0.112702	0.5
86	0.5	0.788675	0.211325	0.211325
87	0.5	0.788675	0.211325	0.5
88	0.5	0.788675	0.211325	0.788675
89	0.5	0.788675	0.5	0.112702
90	0.5	0.788675	0.5	0.211325
91	0.5	0.788675	0.5	0.5
92	0.5	0.788675	0.5	0.788675
93	0.5	0.788675	0.5	0.887298
94	0.5	0.788675	0.788675	0.211325
95	0.5	0.788675	0.788675	0.5
96	0.5	0.788675	0.788675	0.788675
97	0.5	0.788675	0.887298	0.5
98	0.5	0.887298	0.211325	0.5
99	0.5	0.887298	0.5	0.211325
100	0.5	0.887298	0.5	0.5
101	0.5	0.887298	0.5	0.788675
102	0.5	0.887298	0.788675	0.5

103	0.5	0.930568	0.5	0.5
104	0.669991	0.5	0.5	0.5
105	0.788675	0.112702	0.5	0.5
106	0.788675	0.211325	0.211325	0.5
107	0.788675	0.211325	0.5	0.211325
108	0.788675	0.211325	0.5	0.5
109	0.788675	0.211325	0.5	0.788675
110	0.788675	0.211325	0.788675	0.5
111	0.788675	0.5	0.112702	0.5
112	0.788675	0.5	0.211325	0.211325
113	0.788675	0.5	0.211325	0.5
114	0.788675	0.5	0.211325	0.788675
115	0.788675	0.5	0.5	0.112702
116	0.788675	0.5	0.5	0.211325
117	0.788675	0.5	0.5	0.5
118	0.788675	0.5	0.5	0.788675
119	0.788675	0.5	0.5	0.887298
120	0.788675	0.5	0.788675	0.211325
121	0.788675	0.5	0.788675	0.5
122	0.788675	0.5	0.788675	0.788675
123	0.788675	0.5	0.887298	0.5
124	0.788675	0.788675	0.211325	0.5
125	0.788675	0.788675	0.5	0.211325
126	0.788675	0.788675	0.5	0.5
127	0.788675	0.788675	0.5	0.788675
128	0.788675	0.788675	0.788675	0.5
129	0.788675	0.887298	0.5	0.5
130	0.887298	0.211325	0.5	0.5
131	0.887298	0.5	0.211325	0.5
132	0.887298	0.5	0.5	0.211325
133	0.887298	0.5	0.5	0.5
134	0.887298	0.5	0.5	0.788675
135	0.887298	0.5	0.788675	0.5
136	0.887298	0.788675	0.5	0.5
137	0.930568	0.5	0.5	0.5
