# Supplementary material for: Development of an Agent-Based Model (ABM) to Simulate the Immune System and Integration of a Regression Method to Estimate the Key ABM Parameters by Fitting the Experimental Data
Source: PLoS One. 2015 Nov 4;10(11):e0141295. doi: 10.1371/journal.pone.0141295 (PMC4633145; doi:10.1371/journal.pone.0141295)
Supplement: S3 Table — (PDF) [file pone.0141295.s004.pdf]

# S3 Table. Sample size 385 genetated by Sparse Grid

| samples | P1 | P2 | P3 | P4 |
|---|---|---|---|---|
| 1 | 0.04691 | 0.5 | 0.5 | 0.5 |
| 2 | 0.069432 | 0.211325 | 0.5 | 0.5 |
| 3 | 0.069432 | 0.5 | 0.211325 | 0.5 |
| 4 | 0.069432 | 0.5 | 0.5 | 0.211325 |
| 5 | 0.069432 | 0.5 | 0.5 | 0.5 |
| 6 | 0.069432 | 0.5 | 0.5 | 0.788675 |
| 7 | 0.069432 | 0.5 | 0.788675 | 0.5 |
| 8 | 0.069432 | 0.788675 | 0.5 | 0.5 |
| 9 | 0.112702 | 0.112702 | 0.5 | 0.5 |
| 10 | 0.112702 | 0.211325 | 0.211325 | 0.5 |
| 11 | 0.112702 | 0.211325 | 0.5 | 0.211325 |
| 12 | 0.112702 | 0.211325 | 0.5 | 0.5 |
| 13 | 0.112702 | 0.211325 | 0.5 | 0.788675 |
| 14 | 0.112702 | 0.211325 | 0.788675 | 0.5 |
| 15 | 0.112702 | 0.5 | 0.112702 | 0.5 |
| 16 | 0.112702 | 0.5 | 0.211325 | 0.211325 |
| 17 | 0.112702 | 0.5 | 0.211325 | 0.5 |
| 18 | 0.112702 | 0.5 | 0.211325 | 0.788675 |
| 19 | 0.112702 | 0.5 | 0.5 | 0.112702 |
| 20 | 0.112702 | 0.5 | 0.5 | 0.211325 |
| 21 | 0.112702 | 0.5 | 0.5 | 0.5 |
| 22 | 0.112702 | 0.5 | 0.5 | 0.788675 |
| 23 | 0.112702 | 0.5 | 0.5 | 0.887298 |
| 24 | 0.112702 | 0.5 | 0.788675 | 0.211325 |
| 25 | 0.112702 | 0.5 | 0.788675 | 0.5 |
| 26 | 0.112702 | 0.5 | 0.788675 | 0.788675 |
| 27 | 0.112702 | 0.5 | 0.887298 | 0.5 |
| 28 | 0.112702 | 0.788675 | 0.211325 | 0.5 |
| 29 | 0.112702 | 0.788675 | 0.5 | 0.211325 |
| 30 | 0.112702 | 0.788675 | 0.5 | 0.5 |
| 31 | 0.112702 | 0.788675 | 0.5 | 0.788675 |
| 32 | 0.112702 | 0.788675 | 0.788675 | 0.5 |
| 33 | 0.112702 | 0.887298 | 0.5 | 0.5 |
| 34 | 0.211325 | 0.069432 | 0.5 | 0.5 |
| 35 | 0.211325 | 0.112702 | 0.211325 | 0.5 |
| 36 | 0.211325 | 0.112702 | 0.5 | 0.211325 |
| 37 | 0.211325 | 0.112702 | 0.5 | 0.5 |
| 38 | 0.211325 | 0.112702 | 0.5 | 0.788675 |
| 39 | 0.211325 | 0.112702 | 0.788675 | 0.5 |
| 40 | 0.211325 | 0.211325 | 0.112702 | 0.5 |
| 41 | 0.211325 | 0.211325 | 0.211325 | 0.211325 |
| 42 | 0.211325 | 0.211325 | 0.211325 | 0.5 |
| 43 | 0.211325 | 0.211325 | 0.211325 | 0.788675 |
| 44 | 0.211325 | 0.211325 | 0.5 | 0.112702 |
| 45 | 0.211325 | 0.211325 | 0.5 | 0.211325 |
| 46 | 0.211325 | 0.211325 | 0.5 | 0.5 |
| 47 | 0.211325 | 0.211325 | 0.5 | 0.788675 |
| 48 | 0.211325 | 0.211325 | 0.5 | 0.887298 |

| | | | | |
|---|---|---|---|---|
| 49 | 0.211325 | 0.211325 | 0.788675 | 0.211325 |
| 50 | 0.211325 | 0.211325 | 0.788675 | 0.5 |
| 51 | 0.211325 | 0.211325 | 0.788675 | 0.788675 |
| 52 | 0.211325 | 0.211325 | 0.887298 | 0.5 |
| 53 | 0.211325 | 0.330009 | 0.5 | 0.5 |
| 54 | 0.211325 | 0.5 | 0.069432 | 0.5 |
| 55 | 0.211325 | 0.5 | 0.112702 | 0.211325 |
| 56 | 0.211325 | 0.5 | 0.112702 | 0.5 |
| 57 | 0.211325 | 0.5 | 0.112702 | 0.788675 |
| 58 | 0.211325 | 0.5 | 0.211325 | 0.112702 |
| 59 | 0.211325 | 0.5 | 0.211325 | 0.211325 |
| 60 | 0.211325 | 0.5 | 0.211325 | 0.5 |
| 61 | 0.211325 | 0.5 | 0.211325 | 0.788675 |
| 62 | 0.211325 | 0.5 | 0.211325 | 0.887298 |
| 63 | 0.211325 | 0.5 | 0.330009 | 0.5 |
| 64 | 0.211325 | 0.5 | 0.5 | 0.069432 |
| 65 | 0.211325 | 0.5 | 0.5 | 0.112702 |
| 66 | 0.211325 | 0.5 | 0.5 | 0.211325 |
| 67 | 0.211325 | 0.5 | 0.5 | 0.330009 |
| 68 | 0.211325 | 0.5 | 0.5 | 0.5 |
| 69 | 0.211325 | 0.5 | 0.5 | 0.669991 |
| 70 | 0.211325 | 0.5 | 0.5 | 0.788675 |
| 71 | 0.211325 | 0.5 | 0.5 | 0.887298 |
| 72 | 0.211325 | 0.5 | 0.5 | 0.930568 |
| 73 | 0.211325 | 0.5 | 0.669991 | 0.5 |
| 74 | 0.211325 | 0.5 | 0.788675 | 0.112702 |
| 75 | 0.211325 | 0.5 | 0.788675 | 0.211325 |
| 76 | 0.211325 | 0.5 | 0.788675 | 0.5 |
| 77 | 0.211325 | 0.5 | 0.788675 | 0.788675 |
| 78 | 0.211325 | 0.5 | 0.788675 | 0.887298 |
| 79 | 0.211325 | 0.5 | 0.887298 | 0.211325 |
| 80 | 0.211325 | 0.5 | 0.887298 | 0.5 |
| 81 | 0.211325 | 0.5 | 0.887298 | 0.788675 |
| 82 | 0.211325 | 0.5 | 0.930568 | 0.5 |
| 83 | 0.211325 | 0.669991 | 0.5 | 0.5 |
| 84 | 0.211325 | 0.788675 | 0.112702 | 0.5 |
| 85 | 0.211325 | 0.788675 | 0.211325 | 0.211325 |
| 86 | 0.211325 | 0.788675 | 0.211325 | 0.5 |
| 87 | 0.211325 | 0.788675 | 0.211325 | 0.788675 |
| 88 | 0.211325 | 0.788675 | 0.5 | 0.112702 |
| 89 | 0.211325 | 0.788675 | 0.5 | 0.211325 |
| 90 | 0.211325 | 0.788675 | 0.5 | 0.5 |
| 91 | 0.211325 | 0.788675 | 0.5 | 0.788675 |
| 92 | 0.211325 | 0.788675 | 0.5 | 0.887298 |
| 93 | 0.211325 | 0.788675 | 0.788675 | 0.211325 |
| 94 | 0.211325 | 0.788675 | 0.788675 | 0.5 |
| 95 | 0.211325 | 0.788675 | 0.788675 | 0.788675 |
| 96 | 0.211325 | 0.788675 | 0.887298 | 0.5 |
| 97 | 0.211325 | 0.887298 | 0.211325 | 0.5 |
| 98 | 0.211325 | 0.887298 | 0.5 | 0.211325 |
| 99 | 0.211325 | 0.887298 | 0.5 | 0.5 |
| 100 | 0.211325 | 0.887298 | 0.5 | 0.788675 |
| 101 | 0.211325 | 0.887298 | 0.788675 | 0.5 |
| 102 | 0.211325 | 0.930568 | 0.5 | 0.5 |

| | | | | |
|---|---|---|---|---|
| 103 | 0.230765 | 0.5 | 0.5 | 0.5 |
| 104 | 0.330009 | 0.211325 | 0.5 | 0.5 |
| 105 | 0.330009 | 0.5 | 0.211325 | 0.5 |
| 106 | 0.330009 | 0.5 | 0.5 | 0.211325 |
| 107 | 0.330009 | 0.5 | 0.5 | 0.5 |
| 108 | 0.330009 | 0.5 | 0.5 | 0.788675 |
| 109 | 0.330009 | 0.5 | 0.788675 | 0.5 |
| 110 | 0.330009 | 0.788675 | 0.5 | 0.5 |
| 111 | 0.5 | 0.04691 | 0.5 | 0.5 |
| 112 | 0.5 | 0.069432 | 0.211325 | 0.5 |
| 113 | 0.5 | 0.069432 | 0.5 | 0.211325 |
| 114 | 0.5 | 0.069432 | 0.5 | 0.5 |
| 115 | 0.5 | 0.069432 | 0.5 | 0.788675 |
| 116 | 0.5 | 0.069432 | 0.788675 | 0.5 |
| 117 | 0.5 | 0.112702 | 0.112702 | 0.5 |
| 118 | 0.5 | 0.112702 | 0.211325 | 0.211325 |
| 119 | 0.5 | 0.112702 | 0.211325 | 0.5 |
| 120 | 0.5 | 0.112702 | 0.211325 | 0.788675 |
| 121 | 0.5 | 0.112702 | 0.5 | 0.112702 |
| 122 | 0.5 | 0.112702 | 0.5 | 0.211325 |
| 123 | 0.5 | 0.112702 | 0.5 | 0.5 |
| 124 | 0.5 | 0.112702 | 0.5 | 0.788675 |
| 125 | 0.5 | 0.112702 | 0.5 | 0.887298 |
| 126 | 0.5 | 0.112702 | 0.788675 | 0.211325 |
| 127 | 0.5 | 0.112702 | 0.788675 | 0.5 |
| 128 | 0.5 | 0.112702 | 0.788675 | 0.788675 |
| 129 | 0.5 | 0.112702 | 0.887298 | 0.5 |
| 130 | 0.5 | 0.211325 | 0.069432 | 0.5 |
| 131 | 0.5 | 0.211325 | 0.112702 | 0.211325 |
| 132 | 0.5 | 0.211325 | 0.112702 | 0.5 |
| 133 | 0.5 | 0.211325 | 0.112702 | 0.788675 |
| 134 | 0.5 | 0.211325 | 0.211325 | 0.112702 |
| 135 | 0.5 | 0.211325 | 0.211325 | 0.211325 |
| 136 | 0.5 | 0.211325 | 0.211325 | 0.5 |
| 137 | 0.5 | 0.211325 | 0.211325 | 0.788675 |
| 138 | 0.5 | 0.211325 | 0.211325 | 0.887298 |
| 139 | 0.5 | 0.211325 | 0.330009 | 0.5 |
| 140 | 0.5 | 0.211325 | 0.5 | 0.069432 |
| 141 | 0.5 | 0.211325 | 0.5 | 0.112702 |
| 142 | 0.5 | 0.211325 | 0.5 | 0.211325 |
| 143 | 0.5 | 0.211325 | 0.5 | 0.330009 |
| 144 | 0.5 | 0.211325 | 0.5 | 0.5 |
| 145 | 0.5 | 0.211325 | 0.5 | 0.669991 |
| 146 | 0.5 | 0.211325 | 0.5 | 0.788675 |
| 147 | 0.5 | 0.211325 | 0.5 | 0.887298 |
| 148 | 0.5 | 0.211325 | 0.5 | 0.930568 |
| 149 | 0.5 | 0.211325 | 0.669991 | 0.5 |
| 150 | 0.5 | 0.211325 | 0.788675 | 0.112702 |
| 151 | 0.5 | 0.211325 | 0.788675 | 0.211325 |
| 152 | 0.5 | 0.211325 | 0.788675 | 0.5 |
| 153 | 0.5 | 0.211325 | 0.788675 | 0.788675 |
| 154 | 0.5 | 0.211325 | 0.788675 | 0.887298 |
| 155 | 0.5 | 0.211325 | 0.887298 | 0.211325 |
| 156 | 0.5 | 0.211325 | 0.887298 | 0.5 |

| | | | | |
|---|---|---|---|---|
| 157 | 0.5 | 0.211325 | 0.887298 | 0.788675 |
| 158 | 0.5 | 0.211325 | 0.930568 | 0.5 |
| 159 | 0.5 | 0.230765 | 0.5 | 0.5 |
| 160 | 0.5 | 0.330009 | 0.211325 | 0.5 |
| 161 | 0.5 | 0.330009 | 0.5 | 0.211325 |
| 162 | 0.5 | 0.330009 | 0.5 | 0.5 |
| 163 | 0.5 | 0.330009 | 0.5 | 0.788675 |
| 164 | 0.5 | 0.330009 | 0.788675 | 0.5 |
| 165 | 0.5 | 0.5 | 0.04691 | 0.5 |
| 166 | 0.5 | 0.5 | 0.069432 | 0.211325 |
| 167 | 0.5 | 0.5 | 0.069432 | 0.5 |
| 168 | 0.5 | 0.5 | 0.069432 | 0.788675 |
| 169 | 0.5 | 0.5 | 0.112702 | 0.112702 |
| 170 | 0.5 | 0.5 | 0.112702 | 0.211325 |
| 171 | 0.5 | 0.5 | 0.112702 | 0.5 |
| 172 | 0.5 | 0.5 | 0.112702 | 0.788675 |
| 173 | 0.5 | 0.5 | 0.112702 | 0.887298 |
| 174 | 0.5 | 0.5 | 0.211325 | 0.069432 |
| 175 | 0.5 | 0.5 | 0.211325 | 0.112702 |
| 176 | 0.5 | 0.5 | 0.211325 | 0.211325 |
| 177 | 0.5 | 0.5 | 0.211325 | 0.330009 |
| 178 | 0.5 | 0.5 | 0.211325 | 0.5 |
| 179 | 0.5 | 0.5 | 0.211325 | 0.669991 |
| 180 | 0.5 | 0.5 | 0.211325 | 0.788675 |
| 181 | 0.5 | 0.5 | 0.211325 | 0.887298 |
| 182 | 0.5 | 0.5 | 0.211325 | 0.930568 |
| 183 | 0.5 | 0.5 | 0.230765 | 0.5 |
| 184 | 0.5 | 0.5 | 0.330009 | 0.211325 |
| 185 | 0.5 | 0.5 | 0.330009 | 0.5 |
| 186 | 0.5 | 0.5 | 0.330009 | 0.788675 |
| 187 | 0.5 | 0.5 | 0.5 | 0.04691 |
| 188 | 0.5 | 0.5 | 0.5 | 0.069432 |
| 189 | 0.5 | 0.5 | 0.5 | 0.112702 |
| 190 | 0.5 | 0.5 | 0.5 | 0.211325 |
| 191 | 0.5 | 0.5 | 0.5 | 0.230765 |
| 192 | 0.5 | 0.5 | 0.5 | 0.330009 |
| 193 | 0.5 | 0.5 | 0.5 | 0.5 |
| 194 | 0.5 | 0.5 | 0.5 | 0.669991 |
| 195 | 0.5 | 0.5 | 0.5 | 0.769235 |
| 196 | 0.5 | 0.5 | 0.5 | 0.788675 |
| 197 | 0.5 | 0.5 | 0.5 | 0.887298 |
| 198 | 0.5 | 0.5 | 0.5 | 0.930568 |
| 199 | 0.5 | 0.5 | 0.5 | 0.95309 |
| 200 | 0.5 | 0.5 | 0.669991 | 0.211325 |
| 201 | 0.5 | 0.5 | 0.669991 | 0.5 |
| 202 | 0.5 | 0.5 | 0.669991 | 0.788675 |
| 203 | 0.5 | 0.5 | 0.769235 | 0.5 |
| 204 | 0.5 | 0.5 | 0.788675 | 0.069432 |
| 205 | 0.5 | 0.5 | 0.788675 | 0.112702 |
| 206 | 0.5 | 0.5 | 0.788675 | 0.211325 |
| 207 | 0.5 | 0.5 | 0.788675 | 0.330009 |
| 208 | 0.5 | 0.5 | 0.788675 | 0.5 |
| 209 | 0.5 | 0.5 | 0.788675 | 0.669991 |
| 210 | 0.5 | 0.5 | 0.788675 | 0.788675 |

| | | | | |
|---|---|---|---|---|
| 211 | 0.5 | 0.5 | 0.788675 | 0.887298 |
| 212 | 0.5 | 0.5 | 0.788675 | 0.930568 |
| 213 | 0.5 | 0.5 | 0.887298 | 0.112702 |
| 214 | 0.5 | 0.5 | 0.887298 | 0.211325 |
| 215 | 0.5 | 0.5 | 0.887298 | 0.5 |
| 216 | 0.5 | 0.5 | 0.887298 | 0.788675 |
| 217 | 0.5 | 0.5 | 0.887298 | 0.887298 |
| 218 | 0.5 | 0.5 | 0.930568 | 0.211325 |
| 219 | 0.5 | 0.5 | 0.930568 | 0.5 |
| 220 | 0.5 | 0.5 | 0.930568 | 0.788675 |
| 221 | 0.5 | 0.5 | 0.95309 | 0.5 |
| 222 | 0.5 | 0.669991 | 0.211325 | 0.5 |
| 223 | 0.5 | 0.669991 | 0.5 | 0.211325 |
| 224 | 0.5 | 0.669991 | 0.5 | 0.5 |
| 225 | 0.5 | 0.669991 | 0.5 | 0.788675 |
| 226 | 0.5 | 0.669991 | 0.788675 | 0.5 |
| 227 | 0.5 | 0.769235 | 0.5 | 0.5 |
| 228 | 0.5 | 0.788675 | 0.069432 | 0.5 |
| 229 | 0.5 | 0.788675 | 0.112702 | 0.211325 |
| 230 | 0.5 | 0.788675 | 0.112702 | 0.5 |
| 231 | 0.5 | 0.788675 | 0.112702 | 0.788675 |
| 232 | 0.5 | 0.788675 | 0.211325 | 0.112702 |
| 233 | 0.5 | 0.788675 | 0.211325 | 0.211325 |
| 234 | 0.5 | 0.788675 | 0.211325 | 0.5 |
| 235 | 0.5 | 0.788675 | 0.211325 | 0.788675 |
| 236 | 0.5 | 0.788675 | 0.211325 | 0.887298 |
| 237 | 0.5 | 0.788675 | 0.330009 | 0.5 |
| 238 | 0.5 | 0.788675 | 0.5 | 0.069432 |
| 239 | 0.5 | 0.788675 | 0.5 | 0.112702 |
| 240 | 0.5 | 0.788675 | 0.5 | 0.211325 |
| 241 | 0.5 | 0.788675 | 0.5 | 0.330009 |
| 242 | 0.5 | 0.788675 | 0.5 | 0.5 |
| 243 | 0.5 | 0.788675 | 0.5 | 0.669991 |
| 244 | 0.5 | 0.788675 | 0.5 | 0.788675 |
| 245 | 0.5 | 0.788675 | 0.5 | 0.887298 |
| 246 | 0.5 | 0.788675 | 0.5 | 0.930568 |
| 247 | 0.5 | 0.788675 | 0.669991 | 0.5 |
| 248 | 0.5 | 0.788675 | 0.788675 | 0.112702 |
| 249 | 0.5 | 0.788675 | 0.788675 | 0.211325 |
| 250 | 0.5 | 0.788675 | 0.788675 | 0.5 |
| 251 | 0.5 | 0.788675 | 0.788675 | 0.788675 |
| 252 | 0.5 | 0.788675 | 0.788675 | 0.887298 |
| 253 | 0.5 | 0.788675 | 0.887298 | 0.211325 |
| 254 | 0.5 | 0.788675 | 0.887298 | 0.5 |
| 255 | 0.5 | 0.788675 | 0.887298 | 0.788675 |
| 256 | 0.5 | 0.788675 | 0.930568 | 0.5 |
| 257 | 0.5 | 0.887298 | 0.112702 | 0.5 |
| 258 | 0.5 | 0.887298 | 0.211325 | 0.211325 |
| 259 | 0.5 | 0.887298 | 0.211325 | 0.5 |
| 260 | 0.5 | 0.887298 | 0.211325 | 0.788675 |
| 261 | 0.5 | 0.887298 | 0.5 | 0.112702 |
| 262 | 0.5 | 0.887298 | 0.5 | 0.211325 |
| 263 | 0.5 | 0.887298 | 0.5 | 0.5 |
| 264 | 0.5 | 0.887298 | 0.5 | 0.788675 |

| | | | | |
|---|---|---|---|---|
| 265 | 0.5 | 0.887298 | 0.5 | 0.887298 |
| 266 | 0.5 | 0.887298 | 0.788675 | 0.211325 |
| 267 | 0.5 | 0.887298 | 0.788675 | 0.5 |
| 268 | 0.5 | 0.887298 | 0.788675 | 0.788675 |
| 269 | 0.5 | 0.887298 | 0.887298 | 0.5 |
| 270 | 0.5 | 0.930568 | 0.211325 | 0.5 |
| 271 | 0.5 | 0.930568 | 0.5 | 0.211325 |
| 272 | 0.5 | 0.930568 | 0.5 | 0.5 |
| 273 | 0.5 | 0.930568 | 0.5 | 0.788675 |
| 274 | 0.5 | 0.930568 | 0.788675 | 0.5 |
| 275 | 0.5 | 0.95309 | 0.5 | 0.5 |
| 276 | 0.669991 | 0.211325 | 0.5 | 0.5 |
| 277 | 0.669991 | 0.5 | 0.211325 | 0.5 |
| 278 | 0.669991 | 0.5 | 0.5 | 0.211325 |
| 279 | 0.669991 | 0.5 | 0.5 | 0.5 |
| 280 | 0.669991 | 0.5 | 0.5 | 0.788675 |
| 281 | 0.669991 | 0.5 | 0.788675 | 0.5 |
| 282 | 0.669991 | 0.788675 | 0.5 | 0.5 |
| 283 | 0.769235 | 0.5 | 0.5 | 0.5 |
| 284 | 0.788675 | 0.069432 | 0.5 | 0.5 |
| 285 | 0.788675 | 0.112702 | 0.211325 | 0.5 |
| 286 | 0.788675 | 0.112702 | 0.5 | 0.211325 |
| 287 | 0.788675 | 0.112702 | 0.5 | 0.5 |
| 288 | 0.788675 | 0.112702 | 0.5 | 0.788675 |
| 289 | 0.788675 | 0.112702 | 0.788675 | 0.5 |
| 290 | 0.788675 | 0.211325 | 0.112702 | 0.5 |
| 291 | 0.788675 | 0.211325 | 0.211325 | 0.211325 |
| 292 | 0.788675 | 0.211325 | 0.211325 | 0.5 |
| 293 | 0.788675 | 0.211325 | 0.211325 | 0.788675 |
| 294 | 0.788675 | 0.211325 | 0.5 | 0.112702 |
| 295 | 0.788675 | 0.211325 | 0.5 | 0.211325 |
| 296 | 0.788675 | 0.211325 | 0.5 | 0.5 |
| 297 | 0.788675 | 0.211325 | 0.5 | 0.788675 |
| 298 | 0.788675 | 0.211325 | 0.5 | 0.887298 |
| 299 | 0.788675 | 0.211325 | 0.788675 | 0.211325 |
| 300 | 0.788675 | 0.211325 | 0.788675 | 0.5 |
| 301 | 0.788675 | 0.211325 | 0.788675 | 0.788675 |
| 302 | 0.788675 | 0.211325 | 0.887298 | 0.5 |
| 303 | 0.788675 | 0.330009 | 0.5 | 0.5 |
| 304 | 0.788675 | 0.5 | 0.069432 | 0.5 |
| 305 | 0.788675 | 0.5 | 0.112702 | 0.211325 |
| 306 | 0.788675 | 0.5 | 0.112702 | 0.5 |
| 307 | 0.788675 | 0.5 | 0.112702 | 0.788675 |
| 308 | 0.788675 | 0.5 | 0.211325 | 0.112702 |
| 309 | 0.788675 | 0.5 | 0.211325 | 0.211325 |
| 310 | 0.788675 | 0.5 | 0.211325 | 0.5 |
| 311 | 0.788675 | 0.5 | 0.211325 | 0.788675 |
| 312 | 0.788675 | 0.5 | 0.211325 | 0.887298 |
| 313 | 0.788675 | 0.5 | 0.330009 | 0.5 |
| 314 | 0.788675 | 0.5 | 0.5 | 0.069432 |
| 315 | 0.788675 | 0.5 | 0.5 | 0.112702 |
| 316 | 0.788675 | 0.5 | 0.5 | 0.211325 |
| 317 | 0.788675 | 0.5 | 0.5 | 0.330009 |
| 318 | 0.788675 | 0.5 | 0.5 | 0.5 |

| | | | | |
|---|---|---|---|---|
| 319 | 0.788675 | 0.5 | 0.5 | 0.669991 |
| 320 | 0.788675 | 0.5 | 0.5 | 0.788675 |
| 321 | 0.788675 | 0.5 | 0.5 | 0.887298 |
| 322 | 0.788675 | 0.5 | 0.5 | 0.930568 |
| 323 | 0.788675 | 0.5 | 0.669991 | 0.5 |
| 324 | 0.788675 | 0.5 | 0.788675 | 0.112702 |
| 325 | 0.788675 | 0.5 | 0.788675 | 0.211325 |
| 326 | 0.788675 | 0.5 | 0.788675 | 0.5 |
| 327 | 0.788675 | 0.5 | 0.788675 | 0.788675 |
| 328 | 0.788675 | 0.5 | 0.788675 | 0.887298 |
| 329 | 0.788675 | 0.5 | 0.887298 | 0.211325 |
| 330 | 0.788675 | 0.5 | 0.887298 | 0.5 |
| 331 | 0.788675 | 0.5 | 0.887298 | 0.788675 |
| 332 | 0.788675 | 0.5 | 0.930568 | 0.5 |
| 333 | 0.788675 | 0.669991 | 0.5 | 0.5 |
| 334 | 0.788675 | 0.788675 | 0.112702 | 0.5 |
| 335 | 0.788675 | 0.788675 | 0.211325 | 0.211325 |
| 336 | 0.788675 | 0.788675 | 0.211325 | 0.5 |
| 337 | 0.788675 | 0.788675 | 0.211325 | 0.788675 |
| 338 | 0.788675 | 0.788675 | 0.5 | 0.112702 |
| 339 | 0.788675 | 0.788675 | 0.5 | 0.211325 |
| 340 | 0.788675 | 0.788675 | 0.5 | 0.5 |
| 341 | 0.788675 | 0.788675 | 0.5 | 0.788675 |
| 342 | 0.788675 | 0.788675 | 0.5 | 0.887298 |
| 343 | 0.788675 | 0.788675 | 0.788675 | 0.211325 |
| 344 | 0.788675 | 0.788675 | 0.788675 | 0.5 |
| 345 | 0.788675 | 0.788675 | 0.788675 | 0.788675 |
| 346 | 0.788675 | 0.788675 | 0.887298 | 0.5 |
| 347 | 0.788675 | 0.887298 | 0.211325 | 0.5 |
| 348 | 0.788675 | 0.887298 | 0.5 | 0.211325 |
| 349 | 0.788675 | 0.887298 | 0.5 | 0.5 |
| 350 | 0.788675 | 0.887298 | 0.5 | 0.788675 |
| 351 | 0.788675 | 0.887298 | 0.788675 | 0.5 |
| 352 | 0.788675 | 0.930568 | 0.5 | 0.5 |
| 353 | 0.887298 | 0.112702 | 0.5 | 0.5 |
| 354 | 0.887298 | 0.211325 | 0.211325 | 0.5 |
| 355 | 0.887298 | 0.211325 | 0.5 | 0.211325 |
| 356 | 0.887298 | 0.211325 | 0.5 | 0.5 |
| 357 | 0.887298 | 0.211325 | 0.5 | 0.788675 |
| 358 | 0.887298 | 0.211325 | 0.788675 | 0.5 |
| 359 | 0.887298 | 0.5 | 0.112702 | 0.5 |
| 360 | 0.887298 | 0.5 | 0.211325 | 0.211325 |
| 361 | 0.887298 | 0.5 | 0.211325 | 0.5 |
| 362 | 0.887298 | 0.5 | 0.211325 | 0.788675 |
| 363 | 0.887298 | 0.5 | 0.5 | 0.112702 |
| 364 | 0.887298 | 0.5 | 0.5 | 0.211325 |
| 365 | 0.887298 | 0.5 | 0.5 | 0.5 |
| 366 | 0.887298 | 0.5 | 0.5 | 0.788675 |
| 367 | 0.887298 | 0.5 | 0.5 | 0.887298 |
| 368 | 0.887298 | 0.5 | 0.788675 | 0.211325 |
| 369 | 0.887298 | 0.5 | 0.788675 | 0.5 |
| 370 | 0.887298 | 0.5 | 0.788675 | 0.788675 |
| 371 | 0.887298 | 0.5 | 0.887298 | 0.5 |
| 372 | 0.887298 | 0.788675 | 0.211325 | 0.5 |

| | | | | |
|---|---|---|---|---|
| 373 | 0.887298 | 0.788675 | 0.5 | 0.211325 |
| 374 | 0.887298 | 0.788675 | 0.5 | 0.5 |
| 375 | 0.887298 | 0.788675 | 0.5 | 0.788675 |
| 376 | 0.887298 | 0.788675 | 0.788675 | 0.5 |
| 377 | 0.887298 | 0.887298 | 0.5 | 0.5 |
| 378 | 0.930568 | 0.211325 | 0.5 | 0.5 |
| 379 | 0.930568 | 0.5 | 0.211325 | 0.5 |
| 380 | 0.930568 | 0.5 | 0.5 | 0.211325 |
| 381 | 0.930568 | 0.5 | 0.5 | 0.5 |
| 382 | 0.930568 | 0.5 | 0.5 | 0.788675 |
| 383 | 0.930568 | 0.5 | 0.788675 | 0.5 |
| 384 | 0.930568 | 0.788675 | 0.5 | 0.5 |
| 385 | 0.95309 | 0.5 | 0.5 | 0.5 |
