# Supplementary material for: Development of an Agent-Based Model (ABM) to Simulate the Immune System and Integration of a Regression Method to Estimate the Key ABM Parameters by Fitting the Experimental Data
Source: PLoS One. 2015 Nov 4;10(11):e0141295. doi: 10.1371/journal.pone.0141295 (PMC4633145; doi:10.1371/journal.pone.0141295)
Supplement: S4 Table — (PDF) [file pone.0141295.s005.pdf]

S4 Table. Input data of ABM mapped from sample size 41

samples	input data of ABM			
	$P_B^{Ep_q}$	$P_T^{Ep_q}$	$P_D^{Ep_q^*}$	P_D^V
1	0.000000001397501	0.000000242000000	0.059800000000000	0.423000000000000
2	0.000000002620429	0.000000102281252	0.059800000000000	0.423000000000000
3	0.000000002620429	0.000000242000000	0.025274458040000	0.423000000000000
4	0.000000002620429	0.000000242000000	0.059800000000000	0.178780865400000
5	0.000000002620429	0.000000242000000	0.059800000000000	0.423000000000000
6	0.000000002620429	0.000000242000000	0.059800000000000	0.667219134600000
7	0.000000002620429	0.000000242000000	0.094325541960000	0.423000000000000
8	0.000000002620429	0.000000381718748	0.059800000000000	0.423000000000000
9	0.000000006200000	0.000000054547623	0.059800000000000	0.423000000000000
10	0.000000006200000	0.000000102281252	0.025274458040000	0.423000000000000
11	0.000000006200000	0.000000102281252	0.059800000000000	0.178780865400000
12	0.000000006200000	0.000000102281252	0.059800000000000	0.423000000000000
13	0.000000006200000	0.000000102281252	0.059800000000000	0.667219134600000
14	0.000000006200000	0.000000102281252	0.094325541960000	0.423000000000000
15	0.000000006200000	0.000000242000000	0.013479123320000	0.423000000000000
16	0.000000006200000	0.000000242000000	0.025274458040000	0.178780865400000
17	0.000000006200000	0.000000242000000	0.025274458040000	0.423000000000000
18	0.000000006200000	0.000000242000000	0.025274458040000	0.667219134600000
19	0.000000006200000	0.000000242000000	0.059800000000000	0.095345638200000
20	0.000000006200000	0.000000242000000	0.059800000000000	0.178780865400000
21	0.000000006200000	0.000000242000000	0.059800000000000	0.423000000000000
22	0.000000006200000	0.000000242000000	0.059800000000000	0.667219134600000
23	0.000000006200000	0.000000242000000	0.059800000000000	0.750654361800000
24	0.000000006200000	0.000000242000000	0.094325541960000	0.178780865400000
25	0.000000006200000	0.000000242000000	0.094325541960000	0.423000000000000
26	0.000000006200000	0.000000242000000	0.094325541960000	0.667219134600000
27	0.000000006200000	0.000000242000000	0.106120876680000	0.423000000000000
28	0.000000006200000	0.000000381718748	0.025274458040000	0.423000000000000
29	0.000000006200000	0.000000381718748	0.059800000000000	0.178780865400000
30	0.000000006200000	0.000000381718748	0.059800000000000	0.423000000000000
31	0.000000006200000	0.000000381718748	0.059800000000000	0.667219134600000
32	0.000000006200000	0.000000381718748	0.094325541960000	0.423000000000000
33	0.000000006200000	0.000000429452377	0.059800000000000	0.423000000000000
34	0.000000009779571	0.000000102281252	0.059800000000000	0.423000000000000
35	0.000000009779571	0.000000242000000	0.025274458040000	0.423000000000000
36	0.000000009779571	0.000000242000000	0.059800000000000	0.178780865400000
37	0.000000009779571	0.000000242000000	0.059800000000000	0.423000000000000
38	0.000000009779571	0.000000242000000	0.059800000000000	0.667219134600000
39	0.000000009779571	0.000000242000000	0.094325541960000	0.423000000000000
40	0.000000009779571	0.000000381718748	0.059800000000000	0.423000000000000
41	0.000000011002499	0.000000242000000	0.059800000000000	0.423000000000000
