# Supplementary material for: Development of an Agent-Based Model (ABM) to Simulate the Immune System and Integration of a Regression Method to Estimate the Key ABM Parameters by Fitting the Experimental Data
Source: PLoS One. 2015 Nov 4;10(11):e0141295. doi: 10.1371/journal.pone.0141295 (PMC4633145; doi:10.1371/journal.pone.0141295)
Supplement: S5 Table — (PDF) [file pone.0141295.s006.pdf]

S5 Table. Input data of ABM mapped from sample size 137

samples	input data of ABM			
	$P_B^{Ep_q}$	$P_T^{Ep_q}$	$P_D^{Ep_q^*}$	P_D^V
1	0.000000000860955	0.000000242000000	0.059800000000000	0.423000000000000
2	0.000000001397501	0.000000102281235	0.059800000000000	0.423000000000000
3	0.000000001397501	0.000000242000000	0.025274453854000	0.423000000000000
4	0.000000001397501	0.000000242000000	0.059800000000000	0.178780835790000
5	0.000000001397501	0.000000242000000	0.059800000000000	0.423000000000000
6	0.000000001397501	0.000000242000000	0.059800000000000	0.667219164210000
7	0.000000001397501	0.000000242000000	0.094325546146000	0.423000000000000
8	0.000000001397501	0.000000381718765	0.059800000000000	0.423000000000000
9	0.000000002620428	0.000000054547606	0.059800000000000	0.423000000000000
10	0.000000002620428	0.000000102281235	0.025274453854000	0.423000000000000
11	0.000000002620428	0.000000102281235	0.059800000000000	0.178780835790000
12	0.000000002620428	0.000000102281235	0.059800000000000	0.423000000000000
13	0.000000002620428	0.000000102281235	0.059800000000000	0.667219164210000
14	0.000000002620428	0.000000102281235	0.094325546146000	0.423000000000000
15	0.000000002620428	0.000000242000000	0.013479119134000	0.423000000000000
16	0.000000002620428	0.000000242000000	0.025274453854000	0.178780835790000
17	0.000000002620428	0.000000242000000	0.025274453854000	0.423000000000000
18	0.000000002620428	0.000000242000000	0.025274453854000	0.667219164210000
19	0.000000002620428	0.000000242000000	0.059800000000000	0.095345608590000
20	0.000000002620428	0.000000242000000	0.059800000000000	0.178780835790000
21	0.000000002620428	0.000000242000000	0.059800000000000	0.423000000000000
22	0.000000002620428	0.000000242000000	0.059800000000000	0.667219164210000
23	0.000000002620428	0.000000242000000	0.059800000000000	0.750654391410000
24	0.000000002620428	0.000000242000000	0.094325546146000	0.178780835790000
25	0.000000002620428	0.000000242000000	0.094325546146000	0.423000000000000
26	0.000000002620428	0.000000242000000	0.094325546146000	0.667219164210000
27	0.000000002620428	0.000000242000000	0.106120880866000	0.423000000000000
28	0.000000002620428	0.000000381718765	0.025274453854000	0.423000000000000
29	0.000000002620428	0.000000381718765	0.059800000000000	0.178780835790000
30	0.000000002620428	0.000000381718765	0.059800000000000	0.423000000000000
31	0.000000002620428	0.000000381718765	0.059800000000000	0.667219164210000
32	0.000000002620428	0.000000381718765	0.094325546146000	0.423000000000000
33	0.000000002620428	0.000000429452394	0.059800000000000	0.423000000000000
34	0.000000004092118	0.000000242000000	0.059800000000000	0.423000000000000
35	0.000000006200000	0.000000033605012	0.059800000000000	0.423000000000000
36	0.000000006200000	0.000000054547606	0.025274453854000	0.423000000000000
37	0.000000006200000	0.000000054547606	0.059800000000000	0.178780835790000
38	0.000000006200000	0.000000054547606	0.059800000000000	0.423000000000000
39	0.000000006200000	0.000000054547606	0.059800000000000	0.667219164210000
40	0.000000006200000	0.000000054547606	0.094325546146000	0.423000000000000
41	0.000000006200000	0.000000102281235	0.013479119134000	0.423000000000000
42	0.000000006200000	0.000000102281235	0.025274453854000	0.178780835790000
43	0.000000006200000	0.000000102281235	0.025274453854000	0.423000000000000
44	0.000000006200000	0.000000102281235	0.025274453854000	0.667219164210000
45	0.000000006200000	0.000000102281235	0.059800000000000	0.095345608590000
46	0.000000006200000	0.000000102281235	0.059800000000000	0.178780835790000

47	0.000000006200000	0.000000102281235	0.059800000000000	0.423000000000000
48	0.000000006200000	0.000000102281235	0.059800000000000	0.667219164210000
49	0.000000006200000	0.000000102281235	0.059800000000000	0.750654391410000
50	0.000000006200000	0.000000102281235	0.094325546146000	0.178780835790000
51	0.000000006200000	0.000000102281235	0.094325546146000	0.423000000000000
52	0.000000006200000	0.000000102281235	0.094325546146000	0.667219164210000
53	0.000000006200000	0.000000102281235	0.106120880866000	0.423000000000000
54	0.000000006200000	0.000000159724587	0.059800000000000	0.423000000000000
55	0.000000006200000	0.000000242000000	0.008304048542400	0.423000000000000
56	0.000000006200000	0.000000242000000	0.013479119134000	0.178780835790000
57	0.000000006200000	0.000000242000000	0.013479119134000	0.423000000000000
58	0.000000006200000	0.000000242000000	0.013479119134000	0.667219164210000
59	0.000000006200000	0.000000242000000	0.025274453854000	0.095345608590000
60	0.000000006200000	0.000000242000000	0.025274453854000	0.178780835790000
61	0.000000006200000	0.000000242000000	0.025274453854000	0.423000000000000
62	0.000000006200000	0.000000242000000	0.025274453854000	0.667219164210000
63	0.000000006200000	0.000000242000000	0.025274453854000	0.750654391410000
64	0.000000006200000	0.000000242000000	0.039469133568800	0.423000000000000
65	0.000000006200000	0.000000242000000	0.059800000000000	0.058739340024000
66	0.000000006200000	0.000000242000000	0.059800000000000	0.095345608590000
67	0.000000006200000	0.000000242000000	0.059800000000000	0.178780835790000
68	0.000000006200000	0.000000242000000	0.059800000000000	0.279188018388000
69	0.000000006200000	0.000000242000000	0.059800000000000	0.423000000000000
70	0.000000006200000	0.000000242000000	0.059800000000000	0.566811981612000
71	0.000000006200000	0.000000242000000	0.059800000000000	0.667219164210000
72	0.000000006200000	0.000000242000000	0.059800000000000	0.750654391410000
73	0.000000006200000	0.000000242000000	0.059800000000000	0.787260659976000
74	0.000000006200000	0.000000242000000	0.080130866431200	0.423000000000000
75	0.000000006200000	0.000000242000000	0.094325546146000	0.095345608590000
76	0.000000006200000	0.000000242000000	0.094325546146000	0.178780835790000
77	0.000000006200000	0.000000242000000	0.094325546146000	0.423000000000000
78	0.000000006200000	0.000000242000000	0.094325546146000	0.667219164210000
79	0.000000006200000	0.000000242000000	0.094325546146000	0.750654391410000
80	0.000000006200000	0.000000242000000	0.106120880866000	0.178780835790000
81	0.000000006200000	0.000000242000000	0.106120880866000	0.423000000000000
82	0.000000006200000	0.000000242000000	0.106120880866000	0.667219164210000
83	0.000000006200000	0.000000242000000	0.111295951457600	0.423000000000000
84	0.000000006200000	0.000000324275413	0.059800000000000	0.423000000000000
85	0.000000006200000	0.000000381718765	0.013479119134000	0.423000000000000
86	0.000000006200000	0.000000381718765	0.025274453854000	0.178780835790000
87	0.000000006200000	0.000000381718765	0.025274453854000	0.423000000000000
88	0.000000006200000	0.000000381718765	0.025274453854000	0.667219164210000
89	0.000000006200000	0.000000381718765	0.059800000000000	0.095345608590000
90	0.000000006200000	0.000000381718765	0.059800000000000	0.178780835790000
91	0.000000006200000	0.000000381718765	0.059800000000000	0.423000000000000
92	0.000000006200000	0.000000381718765	0.059800000000000	0.667219164210000
93	0.000000006200000	0.000000381718765	0.059800000000000	0.750654391410000
94	0.000000006200000	0.000000381718765	0.094325546146000	0.178780835790000
95	0.000000006200000	0.000000381718765	0.094325546146000	0.423000000000000
96	0.000000006200000	0.000000381718765	0.094325546146000	0.667219164210000
97	0.000000006200000	0.000000381718765	0.106120880866000	0.423000000000000
98	0.000000006200000	0.000000429452394	0.025274453854000	0.423000000000000
99	0.000000006200000	0.000000429452394	0.059800000000000	0.178780835790000
100	0.000000006200000	0.000000429452394	0.059800000000000	0.423000000000000

101	0.000000006200000	0.000000429452394	0.059800000000000	0.667219164210000
102	0.000000006200000	0.000000429452394	0.094325546146000	0.423000000000000
103	0.000000006200000	0.000000450394988	0.059800000000000	0.423000000000000
104	0.000000008307882	0.000000242000000	0.059800000000000	0.423000000000000
105	0.000000009779572	0.000000054547606	0.059800000000000	0.423000000000000
106	0.000000009779572	0.000000102281235	0.025274453854000	0.423000000000000
107	0.000000009779572	0.000000102281235	0.059800000000000	0.178780835790000
108	0.000000009779572	0.000000102281235	0.059800000000000	0.423000000000000
109	0.000000009779572	0.000000102281235	0.059800000000000	0.667219164210000
110	0.000000009779572	0.000000102281235	0.094325546146000	0.423000000000000
111	0.000000009779572	0.000000242000000	0.013479119134000	0.423000000000000
112	0.000000009779572	0.000000242000000	0.025274453854000	0.178780835790000
113	0.000000009779572	0.000000242000000	0.025274453854000	0.423000000000000
114	0.000000009779572	0.000000242000000	0.025274453854000	0.667219164210000
115	0.000000009779572	0.000000242000000	0.059800000000000	0.095345608590000
116	0.000000009779572	0.000000242000000	0.059800000000000	0.178780835790000
117	0.000000009779572	0.000000242000000	0.059800000000000	0.423000000000000
118	0.000000009779572	0.000000242000000	0.059800000000000	0.667219164210000
119	0.000000009779572	0.000000242000000	0.059800000000000	0.750654391410000
120	0.000000009779572	0.000000242000000	0.094325546146000	0.178780835790000
121	0.000000009779572	0.000000242000000	0.094325546146000	0.423000000000000
122	0.000000009779572	0.000000242000000	0.094325546146000	0.667219164210000
123	0.000000009779572	0.000000242000000	0.106120880866000	0.423000000000000
124	0.000000009779572	0.000000381718765	0.025274453854000	0.423000000000000
125	0.000000009779572	0.000000381718765	0.059800000000000	0.178780835790000
126	0.000000009779572	0.000000381718765	0.059800000000000	0.423000000000000
127	0.000000009779572	0.000000381718765	0.059800000000000	0.667219164210000
128	0.000000009779572	0.000000381718765	0.094325546146000	0.423000000000000
129	0.000000009779572	0.000000429452394	0.059800000000000	0.423000000000000
130	0.000000011002499	0.000000102281235	0.059800000000000	0.423000000000000
131	0.000000011002499	0.000000242000000	0.025274453854000	0.423000000000000
132	0.000000011002499	0.000000242000000	0.059800000000000	0.178780835790000
133	0.000000011002499	0.000000242000000	0.059800000000000	0.423000000000000
134	0.000000011002499	0.000000242000000	0.059800000000000	0.667219164210000
135	0.000000011002499	0.000000242000000	0.094325546146000	0.423000000000000
136	0.000000011002499	0.000000381718765	0.059800000000000	0.423000000000000
137	0.000000011539045	0.000000242000000	0.059800000000000	0.423000000000000
