# Supplementary material for: Development of an Agent-Based Model (ABM) to Simulate the Immune System and Integration of a Regression Method to Estimate the Key ABM Parameters by Fitting the Experimental Data
Source: PLoS One. 2015 Nov 4;10(11):e0141295. doi: 10.1371/journal.pone.0141295 (PMC4633145; doi:10.1371/journal.pone.0141295)
Supplement: S6 Table — (PDF) [file pone.0141295.s007.pdf]

S6 Table. Input data of ABM mapped from sample size 385

samples	input data of ABM			
	$P_B^{Ep_q}$	$P_T^{Ep_q}$	$P_D^{Ep_q^*}$	P_D^V
1	0.000000006490842	0.000000363000000	0.089700000000000	0.634500000000000
2	0.000000006630478	0.000000293140650	0.089700000000000	0.634500000000000
3	0.000000006630478	0.000000363000000	0.072437235000000	0.634500000000000
4	0.000000006630478	0.000000363000000	0.089700000000000	0.512390475000000
5	0.000000006630478	0.000000363000000	0.089700000000000	0.634500000000000
6	0.000000006630478	0.000000363000000	0.089700000000000	0.756609525000000
7	0.000000006630478	0.000000363000000	0.106962765000000	0.634500000000000
8	0.000000006630478	0.000000432859350	0.089700000000000	0.634500000000000
9	0.000000006898752	0.000000269273884	0.089700000000000	0.634500000000000
10	0.000000006898752	0.000000293140650	0.072437235000000	0.634500000000000
11	0.000000006898752	0.000000293140650	0.089700000000000	0.512390475000000
12	0.000000006898752	0.000000293140650	0.089700000000000	0.634500000000000
13	0.000000006898752	0.000000293140650	0.089700000000000	0.756609525000000
14	0.000000006898752	0.000000293140650	0.106962765000000	0.634500000000000
15	0.000000006898752	0.000000363000000	0.066539579600000	0.634500000000000
16	0.000000006898752	0.000000363000000	0.072437235000000	0.512390475000000
17	0.000000006898752	0.000000363000000	0.072437235000000	0.634500000000000
18	0.000000006898752	0.000000363000000	0.072437235000000	0.756609525000000
19	0.000000006898752	0.000000363000000	0.089700000000000	0.470672946000000
20	0.000000006898752	0.000000363000000	0.089700000000000	0.512390475000000
21	0.000000006898752	0.000000363000000	0.089700000000000	0.634500000000000
22	0.000000006898752	0.000000363000000	0.089700000000000	0.756609525000000
23	0.000000006898752	0.000000363000000	0.089700000000000	0.798327054000000
24	0.000000006898752	0.000000363000000	0.106962765000000	0.512390475000000
25	0.000000006898752	0.000000363000000	0.106962765000000	0.634500000000000
26	0.000000006898752	0.000000363000000	0.106962765000000	0.756609525000000
27	0.000000006898752	0.000000363000000	0.112860420400000	0.634500000000000
28	0.000000006898752	0.000000432859350	0.072437235000000	0.634500000000000
29	0.000000006898752	0.000000432859350	0.089700000000000	0.512390475000000
30	0.000000006898752	0.000000432859350	0.089700000000000	0.634500000000000
31	0.000000006898752	0.000000432859350	0.089700000000000	0.756609525000000
32	0.000000006898752	0.000000432859350	0.106962765000000	0.634500000000000
33	0.000000006898752	0.000000456726116	0.089700000000000	0.634500000000000
34	0.000000007510215	0.000000258802544	0.089700000000000	0.634500000000000
35	0.000000007510215	0.000000269273884	0.072437235000000	0.634500000000000
36	0.000000007510215	0.000000269273884	0.089700000000000	0.512390475000000
37	0.000000007510215	0.000000269273884	0.089700000000000	0.634500000000000
38	0.000000007510215	0.000000269273884	0.089700000000000	0.756609525000000
39	0.000000007510215	0.000000269273884	0.106962765000000	0.634500000000000
40	0.000000007510215	0.000000293140650	0.066539579600000	0.634500000000000
41	0.000000007510215	0.000000293140650	0.072437235000000	0.512390475000000
42	0.000000007510215	0.000000293140650	0.072437235000000	0.634500000000000
43	0.000000007510215	0.000000293140650	0.072437235000000	0.756609525000000
44	0.000000007510215	0.000000293140650	0.089700000000000	0.470672946000000
45	0.000000007510215	0.000000293140650	0.089700000000000	0.512390475000000
46	0.000000007510215	0.000000293140650	0.089700000000000	0.634500000000000

47	0.000000007510215	0.000000293140650	0.089700000000000	0.756609525000000
48	0.000000007510215	0.000000293140650	0.089700000000000	0.798327054000000
49	0.000000007510215	0.000000293140650	0.106962765000000	0.512390475000000
50	0.000000007510215	0.000000293140650	0.106962765000000	0.634500000000000
51	0.000000007510215	0.000000293140650	0.106962765000000	0.756609525000000
52	0.000000007510215	0.000000293140650	0.112860420400000	0.634500000000000
53	0.000000007510215	0.000000321862178	0.089700000000000	0.634500000000000
54	0.000000007510215	0.000000363000000	0.063952033600000	0.634500000000000
55	0.000000007510215	0.000000363000000	0.066539579600000	0.512390475000000
56	0.000000007510215	0.000000363000000	0.066539579600000	0.634500000000000
57	0.000000007510215	0.000000363000000	0.066539579600000	0.756609525000000
58	0.000000007510215	0.000000363000000	0.072437235000000	0.470672946000000
59	0.000000007510215	0.000000363000000	0.072437235000000	0.512390475000000
60	0.000000007510215	0.000000363000000	0.072437235000000	0.634500000000000
61	0.000000007510215	0.000000363000000	0.072437235000000	0.756609525000000
62	0.000000007510215	0.000000363000000	0.072437235000000	0.798327054000000
63	0.000000007510215	0.000000363000000	0.079534538200000	0.634500000000000
64	0.000000007510215	0.000000363000000	0.089700000000000	0.452369736000000
65	0.000000007510215	0.000000363000000	0.089700000000000	0.470672946000000
66	0.000000007510215	0.000000363000000	0.089700000000000	0.512390475000000
67	0.000000007510215	0.000000363000000	0.089700000000000	0.562593807000000
68	0.000000007510215	0.000000363000000	0.089700000000000	0.634500000000000
69	0.000000007510215	0.000000363000000	0.089700000000000	0.706406193000000
70	0.000000007510215	0.000000363000000	0.089700000000000	0.756609525000000
71	0.000000007510215	0.000000363000000	0.089700000000000	0.798327054000000
72	0.000000007510215	0.000000363000000	0.089700000000000	0.816630264000000
73	0.000000007510215	0.000000363000000	0.099865461800000	0.634500000000000
74	0.000000007510215	0.000000363000000	0.106962765000000	0.470672946000000
75	0.000000007510215	0.000000363000000	0.106962765000000	0.512390475000000
76	0.000000007510215	0.000000363000000	0.106962765000000	0.634500000000000
77	0.000000007510215	0.000000363000000	0.106962765000000	0.756609525000000
78	0.000000007510215	0.000000363000000	0.106962765000000	0.798327054000000
79	0.000000007510215	0.000000363000000	0.112860420400000	0.512390475000000
80	0.000000007510215	0.000000363000000	0.112860420400000	0.634500000000000
81	0.000000007510215	0.000000363000000	0.112860420400000	0.756609525000000
82	0.000000007510215	0.000000363000000	0.115447966400000	0.634500000000000
83	0.000000007510215	0.000000404137822	0.089700000000000	0.634500000000000
84	0.000000007510215	0.000000432859350	0.066539579600000	0.634500000000000
85	0.000000007510215	0.000000432859350	0.072437235000000	0.512390475000000
86	0.000000007510215	0.000000432859350	0.072437235000000	0.634500000000000
87	0.000000007510215	0.000000432859350	0.072437235000000	0.756609525000000
88	0.000000007510215	0.000000432859350	0.089700000000000	0.470672946000000
89	0.000000007510215	0.000000432859350	0.089700000000000	0.512390475000000
90	0.000000007510215	0.000000432859350	0.089700000000000	0.634500000000000
91	0.000000007510215	0.000000432859350	0.089700000000000	0.756609525000000
92	0.000000007510215	0.000000432859350	0.089700000000000	0.798327054000000
93	0.000000007510215	0.000000432859350	0.106962765000000	0.512390475000000
94	0.000000007510215	0.000000432859350	0.106962765000000	0.634500000000000
95	0.000000007510215	0.000000432859350	0.106962765000000	0.756609525000000
96	0.000000007510215	0.000000432859350	0.112860420400000	0.634500000000000
97	0.000000007510215	0.000000456726116	0.072437235000000	0.634500000000000
98	0.000000007510215	0.000000456726116	0.089700000000000	0.512390475000000
99	0.000000007510215	0.000000456726116	0.089700000000000	0.634500000000000
100	0.000000007510215	0.000000456726116	0.089700000000000	0.756609525000000

101	0.000000007510215	0.000000456726116	0.106962765000000	0.634500000000000
102	0.000000007510215	0.000000467197456	0.089700000000000	0.634500000000000
103	0.000000007630743	0.000000363000000	0.089700000000000	0.634500000000000
104	0.000000008246056	0.000000293140650	0.089700000000000	0.634500000000000
105	0.000000008246056	0.000000363000000	0.072437235000000	0.634500000000000
106	0.000000008246056	0.000000363000000	0.089700000000000	0.512390475000000
107	0.000000008246056	0.000000363000000	0.089700000000000	0.634500000000000
108	0.000000008246056	0.000000363000000	0.089700000000000	0.756609525000000
109	0.000000008246056	0.000000363000000	0.106962765000000	0.634500000000000
110	0.000000008246056	0.000000432859350	0.089700000000000	0.634500000000000
111	0.000000009300000	0.000000253352220	0.089700000000000	0.634500000000000
112	0.000000009300000	0.000000258802544	0.072437235000000	0.634500000000000
113	0.000000009300000	0.000000258802544	0.089700000000000	0.512390475000000
114	0.000000009300000	0.000000258802544	0.089700000000000	0.634500000000000
115	0.000000009300000	0.000000258802544	0.089700000000000	0.756609525000000
116	0.000000009300000	0.000000258802544	0.106962765000000	0.634500000000000
117	0.000000009300000	0.000000269273884	0.066539579600000	0.634500000000000
118	0.000000009300000	0.000000269273884	0.072437235000000	0.512390475000000
119	0.000000009300000	0.000000269273884	0.072437235000000	0.634500000000000
120	0.000000009300000	0.000000269273884	0.072437235000000	0.756609525000000
121	0.000000009300000	0.000000269273884	0.089700000000000	0.470672946000000
122	0.000000009300000	0.000000269273884	0.089700000000000	0.512390475000000
123	0.000000009300000	0.000000269273884	0.089700000000000	0.634500000000000
124	0.000000009300000	0.000000269273884	0.089700000000000	0.756609525000000
125	0.000000009300000	0.000000269273884	0.089700000000000	0.798327054000000
126	0.000000009300000	0.000000269273884	0.106962765000000	0.512390475000000
127	0.000000009300000	0.000000269273884	0.106962765000000	0.634500000000000
128	0.000000009300000	0.000000269273884	0.106962765000000	0.756609525000000
129	0.000000009300000	0.000000269273884	0.112860420400000	0.634500000000000
130	0.000000009300000	0.000000293140650	0.063952033600000	0.634500000000000
131	0.000000009300000	0.000000293140650	0.066539579600000	0.512390475000000
132	0.000000009300000	0.000000293140650	0.066539579600000	0.634500000000000
133	0.000000009300000	0.000000293140650	0.066539579600000	0.756609525000000
134	0.000000009300000	0.000000293140650	0.072437235000000	0.470672946000000
135	0.000000009300000	0.000000293140650	0.072437235000000	0.512390475000000
136	0.000000009300000	0.000000293140650	0.072437235000000	0.634500000000000
137	0.000000009300000	0.000000293140650	0.072437235000000	0.756609525000000
138	0.000000009300000	0.000000293140650	0.072437235000000	0.798327054000000
139	0.000000009300000	0.000000293140650	0.079534538200000	0.634500000000000
140	0.000000009300000	0.000000293140650	0.089700000000000	0.452369736000000
141	0.000000009300000	0.000000293140650	0.089700000000000	0.470672946000000
142	0.000000009300000	0.000000293140650	0.089700000000000	0.512390475000000
143	0.000000009300000	0.000000293140650	0.089700000000000	0.562593807000000
144	0.000000009300000	0.000000293140650	0.089700000000000	0.634500000000000
145	0.000000009300000	0.000000293140650	0.089700000000000	0.706406193000000
146	0.000000009300000	0.000000293140650	0.089700000000000	0.756609525000000
147	0.000000009300000	0.000000293140650	0.089700000000000	0.798327054000000
148	0.000000009300000	0.000000293140650	0.089700000000000	0.816630264000000
149	0.000000009300000	0.000000293140650	0.099865461800000	0.634500000000000
150	0.000000009300000	0.000000293140650	0.106962765000000	0.470672946000000
151	0.000000009300000	0.000000293140650	0.106962765000000	0.512390475000000
152	0.000000009300000	0.000000293140650	0.106962765000000	0.634500000000000
153	0.000000009300000	0.000000293140650	0.106962765000000	0.756609525000000
154	0.000000009300000	0.000000293140650	0.106962765000000	0.798327054000000

155	0.000000009300000	0.000000293140650	0.112860420400000	0.512390475000000
156	0.000000009300000	0.000000293140650	0.112860420400000	0.634500000000000
157	0.000000009300000	0.000000293140650	0.112860420400000	0.756609525000000
158	0.000000009300000	0.000000293140650	0.115447966400000	0.634500000000000
159	0.000000009300000	0.000000297845130	0.089700000000000	0.634500000000000
160	0.000000009300000	0.000000321862178	0.072437235000000	0.634500000000000
161	0.000000009300000	0.000000321862178	0.089700000000000	0.512390475000000
162	0.000000009300000	0.000000321862178	0.089700000000000	0.634500000000000
163	0.000000009300000	0.000000321862178	0.089700000000000	0.756609525000000
164	0.000000009300000	0.000000321862178	0.106962765000000	0.634500000000000
165	0.000000009300000	0.000000363000000	0.062605218000000	0.634500000000000
166	0.000000009300000	0.000000363000000	0.063952033600000	0.512390475000000
167	0.000000009300000	0.000000363000000	0.063952033600000	0.634500000000000
168	0.000000009300000	0.000000363000000	0.063952033600000	0.756609525000000
169	0.000000009300000	0.000000363000000	0.066539579600000	0.470672946000000
170	0.000000009300000	0.000000363000000	0.066539579600000	0.512390475000000
171	0.000000009300000	0.000000363000000	0.066539579600000	0.634500000000000
172	0.000000009300000	0.000000363000000	0.066539579600000	0.756609525000000
173	0.000000009300000	0.000000363000000	0.066539579600000	0.798327054000000
174	0.000000009300000	0.000000363000000	0.072437235000000	0.452369736000000
175	0.000000009300000	0.000000363000000	0.072437235000000	0.470672946000000
176	0.000000009300000	0.000000363000000	0.072437235000000	0.512390475000000
177	0.000000009300000	0.000000363000000	0.072437235000000	0.562593807000000
178	0.000000009300000	0.000000363000000	0.072437235000000	0.634500000000000
179	0.000000009300000	0.000000363000000	0.072437235000000	0.706406193000000
180	0.000000009300000	0.000000363000000	0.072437235000000	0.756609525000000
181	0.000000009300000	0.000000363000000	0.072437235000000	0.798327054000000
182	0.000000009300000	0.000000363000000	0.072437235000000	0.816630264000000
183	0.000000009300000	0.000000363000000	0.073599747000000	0.634500000000000
184	0.000000009300000	0.000000363000000	0.079534538200000	0.512390475000000
185	0.000000009300000	0.000000363000000	0.079534538200000	0.634500000000000
186	0.000000009300000	0.000000363000000	0.079534538200000	0.756609525000000
187	0.000000009300000	0.000000363000000	0.089700000000000	0.442842930000000
188	0.000000009300000	0.000000363000000	0.089700000000000	0.452369736000000
189	0.000000009300000	0.000000363000000	0.089700000000000	0.470672946000000
190	0.000000009300000	0.000000363000000	0.089700000000000	0.512390475000000
191	0.000000009300000	0.000000363000000	0.089700000000000	0.520613595000000
192	0.000000009300000	0.000000363000000	0.089700000000000	0.562593807000000
193	0.000000009300000	0.000000363000000	0.089700000000000	0.634500000000000
194	0.000000009300000	0.000000363000000	0.089700000000000	0.706406193000000
195	0.000000009300000	0.000000363000000	0.089700000000000	0.748386405000000
196	0.000000009300000	0.000000363000000	0.089700000000000	0.756609525000000
197	0.000000009300000	0.000000363000000	0.089700000000000	0.798327054000000
198	0.000000009300000	0.000000363000000	0.089700000000000	0.816630264000000
199	0.000000009300000	0.000000363000000	0.089700000000000	0.826157070000000
200	0.000000009300000	0.000000363000000	0.099865461800000	0.512390475000000
201	0.000000009300000	0.000000363000000	0.099865461800000	0.634500000000000
202	0.000000009300000	0.000000363000000	0.099865461800000	0.756609525000000
203	0.000000009300000	0.000000363000000	0.105800253000000	0.634500000000000
204	0.000000009300000	0.000000363000000	0.106962765000000	0.452369736000000
205	0.000000009300000	0.000000363000000	0.106962765000000	0.470672946000000
206	0.000000009300000	0.000000363000000	0.106962765000000	0.512390475000000
207	0.000000009300000	0.000000363000000	0.106962765000000	0.562593807000000
208	0.000000009300000	0.000000363000000	0.106962765000000	0.634500000000000

209	0.000000009300000	0.000000363000000	0.106962765000000	0.706406193000000
210	0.000000009300000	0.000000363000000	0.106962765000000	0.756609525000000
211	0.000000009300000	0.000000363000000	0.106962765000000	0.798327054000000
212	0.000000009300000	0.000000363000000	0.106962765000000	0.816630264000000
213	0.000000009300000	0.000000363000000	0.112860420400000	0.470672946000000
214	0.000000009300000	0.000000363000000	0.112860420400000	0.512390475000000
215	0.000000009300000	0.000000363000000	0.112860420400000	0.634500000000000
216	0.000000009300000	0.000000363000000	0.112860420400000	0.756609525000000
217	0.000000009300000	0.000000363000000	0.112860420400000	0.798327054000000
218	0.000000009300000	0.000000363000000	0.115447966400000	0.512390475000000
219	0.000000009300000	0.000000363000000	0.115447966400000	0.634500000000000
220	0.000000009300000	0.000000363000000	0.115447966400000	0.756609525000000
221	0.000000009300000	0.000000363000000	0.116794782000000	0.634500000000000
222	0.000000009300000	0.000000404137822	0.072437235000000	0.634500000000000
223	0.000000009300000	0.000000404137822	0.089700000000000	0.512390475000000
224	0.000000009300000	0.000000404137822	0.089700000000000	0.634500000000000
225	0.000000009300000	0.000000404137822	0.089700000000000	0.756609525000000
226	0.000000009300000	0.000000404137822	0.106962765000000	0.634500000000000
227	0.000000009300000	0.000000428154870	0.089700000000000	0.634500000000000
228	0.000000009300000	0.000000432859350	0.063952033600000	0.634500000000000
229	0.000000009300000	0.000000432859350	0.066539579600000	0.512390475000000
230	0.000000009300000	0.000000432859350	0.066539579600000	0.634500000000000
231	0.000000009300000	0.000000432859350	0.066539579600000	0.756609525000000
232	0.000000009300000	0.000000432859350	0.072437235000000	0.470672946000000
233	0.000000009300000	0.000000432859350	0.072437235000000	0.512390475000000
234	0.000000009300000	0.000000432859350	0.072437235000000	0.634500000000000
235	0.000000009300000	0.000000432859350	0.072437235000000	0.756609525000000
236	0.000000009300000	0.000000432859350	0.072437235000000	0.798327054000000
237	0.000000009300000	0.000000432859350	0.079534538200000	0.634500000000000
238	0.000000009300000	0.000000432859350	0.089700000000000	0.452369736000000
239	0.000000009300000	0.000000432859350	0.089700000000000	0.470672946000000
240	0.000000009300000	0.000000432859350	0.089700000000000	0.512390475000000
241	0.000000009300000	0.000000432859350	0.089700000000000	0.562593807000000
242	0.000000009300000	0.000000432859350	0.089700000000000	0.634500000000000
243	0.000000009300000	0.000000432859350	0.089700000000000	0.706406193000000
244	0.000000009300000	0.000000432859350	0.089700000000000	0.756609525000000
245	0.000000009300000	0.000000432859350	0.089700000000000	0.798327054000000
246	0.000000009300000	0.000000432859350	0.089700000000000	0.816630264000000
247	0.000000009300000	0.000000432859350	0.099865461800000	0.634500000000000
248	0.000000009300000	0.000000432859350	0.106962765000000	0.470672946000000
249	0.000000009300000	0.000000432859350	0.106962765000000	0.512390475000000
250	0.000000009300000	0.000000432859350	0.106962765000000	0.634500000000000
251	0.000000009300000	0.000000432859350	0.106962765000000	0.756609525000000
252	0.000000009300000	0.000000432859350	0.106962765000000	0.798327054000000
253	0.000000009300000	0.000000432859350	0.112860420400000	0.512390475000000
254	0.000000009300000	0.000000432859350	0.112860420400000	0.634500000000000
255	0.000000009300000	0.000000432859350	0.112860420400000	0.756609525000000
256	0.000000009300000	0.000000432859350	0.115447966400000	0.634500000000000
257	0.000000009300000	0.000000456726116	0.066539579600000	0.634500000000000
258	0.000000009300000	0.000000456726116	0.072437235000000	0.512390475000000
259	0.000000009300000	0.000000456726116	0.072437235000000	0.634500000000000
260	0.000000009300000	0.000000456726116	0.072437235000000	0.756609525000000
261	0.000000009300000	0.000000456726116	0.089700000000000	0.470672946000000
262	0.000000009300000	0.000000456726116	0.089700000000000	0.512390475000000

263	0.000000009300000	0.000000456726116	0.089700000000000	0.634500000000000
264	0.000000009300000	0.000000456726116	0.089700000000000	0.756609525000000
265	0.000000009300000	0.000000456726116	0.089700000000000	0.798327054000000
266	0.000000009300000	0.000000456726116	0.106962765000000	0.512390475000000
267	0.000000009300000	0.000000456726116	0.106962765000000	0.634500000000000
268	0.000000009300000	0.000000456726116	0.106962765000000	0.756609525000000
269	0.000000009300000	0.000000456726116	0.112860420400000	0.634500000000000
270	0.000000009300000	0.000000467197456	0.072437235000000	0.634500000000000
271	0.000000009300000	0.000000467197456	0.089700000000000	0.512390475000000
272	0.000000009300000	0.000000467197456	0.089700000000000	0.634500000000000
273	0.000000009300000	0.000000467197456	0.089700000000000	0.756609525000000
274	0.000000009300000	0.000000467197456	0.106962765000000	0.634500000000000
275	0.000000009300000	0.000000472647780	0.089700000000000	0.634500000000000
276	0.000000010353944	0.000000293140650	0.089700000000000	0.634500000000000
277	0.000000010353944	0.000000363000000	0.072437235000000	0.634500000000000
278	0.000000010353944	0.000000363000000	0.089700000000000	0.512390475000000
279	0.000000010353944	0.000000363000000	0.089700000000000	0.634500000000000
280	0.000000010353944	0.000000363000000	0.089700000000000	0.756609525000000
281	0.000000010353944	0.000000363000000	0.106962765000000	0.634500000000000
282	0.000000010353944	0.000000432859350	0.089700000000000	0.634500000000000
283	0.000000010969257	0.000000363000000	0.089700000000000	0.634500000000000
284	0.000000011089785	0.000000258802544	0.089700000000000	0.634500000000000
285	0.000000011089785	0.000000269273884	0.072437235000000	0.634500000000000
286	0.000000011089785	0.000000269273884	0.089700000000000	0.512390475000000
287	0.000000011089785	0.000000269273884	0.089700000000000	0.634500000000000
288	0.000000011089785	0.000000269273884	0.089700000000000	0.756609525000000
289	0.000000011089785	0.000000269273884	0.106962765000000	0.634500000000000
290	0.000000011089785	0.000000293140650	0.066539579600000	0.634500000000000
291	0.000000011089785	0.000000293140650	0.072437235000000	0.512390475000000
292	0.000000011089785	0.000000293140650	0.072437235000000	0.634500000000000
293	0.000000011089785	0.000000293140650	0.072437235000000	0.756609525000000
294	0.000000011089785	0.000000293140650	0.089700000000000	0.470672946000000
295	0.000000011089785	0.000000293140650	0.089700000000000	0.512390475000000
296	0.000000011089785	0.000000293140650	0.089700000000000	0.634500000000000
297	0.000000011089785	0.000000293140650	0.089700000000000	0.756609525000000
298	0.000000011089785	0.000000293140650	0.089700000000000	0.798327054000000
299	0.000000011089785	0.000000293140650	0.106962765000000	0.512390475000000
300	0.000000011089785	0.000000293140650	0.106962765000000	0.634500000000000
301	0.000000011089785	0.000000293140650	0.106962765000000	0.756609525000000
302	0.000000011089785	0.000000293140650	0.112860420400000	0.634500000000000
303	0.000000011089785	0.000000321862178	0.089700000000000	0.634500000000000
304	0.000000011089785	0.000000363000000	0.063952033600000	0.634500000000000
305	0.000000011089785	0.000000363000000	0.066539579600000	0.512390475000000
306	0.000000011089785	0.000000363000000	0.066539579600000	0.634500000000000
307	0.000000011089785	0.000000363000000	0.066539579600000	0.756609525000000
308	0.000000011089785	0.000000363000000	0.072437235000000	0.470672946000000
309	0.000000011089785	0.000000363000000	0.072437235000000	0.512390475000000
310	0.000000011089785	0.000000363000000	0.072437235000000	0.634500000000000
311	0.000000011089785	0.000000363000000	0.072437235000000	0.756609525000000
312	0.000000011089785	0.000000363000000	0.072437235000000	0.798327054000000
313	0.000000011089785	0.000000363000000	0.079534538200000	0.634500000000000
314	0.000000011089785	0.000000363000000	0.089700000000000	0.452369736000000
315	0.000000011089785	0.000000363000000	0.089700000000000	0.470672946000000
316	0.000000011089785	0.000000363000000	0.089700000000000	0.512390475000000

317	0.000000011089785	0.000000363000000	0.089700000000000	0.562593807000000
318	0.000000011089785	0.000000363000000	0.089700000000000	0.634500000000000
319	0.000000011089785	0.000000363000000	0.089700000000000	0.706406193000000
320	0.000000011089785	0.000000363000000	0.089700000000000	0.756609525000000
321	0.000000011089785	0.000000363000000	0.089700000000000	0.798327054000000
322	0.000000011089785	0.000000363000000	0.089700000000000	0.816630264000000
323	0.000000011089785	0.000000363000000	0.099865461800000	0.634500000000000
324	0.000000011089785	0.000000363000000	0.106962765000000	0.470672946000000
325	0.000000011089785	0.000000363000000	0.106962765000000	0.512390475000000
326	0.000000011089785	0.000000363000000	0.106962765000000	0.634500000000000
327	0.000000011089785	0.000000363000000	0.106962765000000	0.756609525000000
328	0.000000011089785	0.000000363000000	0.106962765000000	0.798327054000000
329	0.000000011089785	0.000000363000000	0.112860420400000	0.512390475000000
330	0.000000011089785	0.000000363000000	0.112860420400000	0.634500000000000
331	0.000000011089785	0.000000363000000	0.112860420400000	0.756609525000000
332	0.000000011089785	0.000000363000000	0.115447966400000	0.634500000000000
333	0.000000011089785	0.000000404137822	0.089700000000000	0.634500000000000
334	0.000000011089785	0.000000432859350	0.066539579600000	0.634500000000000
335	0.000000011089785	0.000000432859350	0.072437235000000	0.512390475000000
336	0.000000011089785	0.000000432859350	0.072437235000000	0.634500000000000
337	0.000000011089785	0.000000432859350	0.072437235000000	0.756609525000000
338	0.000000011089785	0.000000432859350	0.089700000000000	0.470672946000000
339	0.000000011089785	0.000000432859350	0.089700000000000	0.512390475000000
340	0.000000011089785	0.000000432859350	0.089700000000000	0.634500000000000
341	0.000000011089785	0.000000432859350	0.089700000000000	0.756609525000000
342	0.000000011089785	0.000000432859350	0.089700000000000	0.798327054000000
343	0.000000011089785	0.000000432859350	0.106962765000000	0.512390475000000
344	0.000000011089785	0.000000432859350	0.106962765000000	0.634500000000000
345	0.000000011089785	0.000000432859350	0.106962765000000	0.756609525000000
346	0.000000011089785	0.000000432859350	0.112860420400000	0.634500000000000
347	0.000000011089785	0.000000456726116	0.072437235000000	0.634500000000000
348	0.000000011089785	0.000000456726116	0.089700000000000	0.512390475000000
349	0.000000011089785	0.000000456726116	0.089700000000000	0.634500000000000
350	0.000000011089785	0.000000456726116	0.089700000000000	0.756609525000000
351	0.000000011089785	0.000000456726116	0.106962765000000	0.634500000000000
352	0.000000011089785	0.000000467197456	0.089700000000000	0.634500000000000
353	0.000000011701248	0.000000269273884	0.089700000000000	0.634500000000000
354	0.000000011701248	0.000000293140650	0.072437235000000	0.634500000000000
355	0.000000011701248	0.000000293140650	0.089700000000000	0.512390475000000
356	0.000000011701248	0.000000293140650	0.089700000000000	0.634500000000000
357	0.000000011701248	0.000000293140650	0.089700000000000	0.756609525000000
358	0.000000011701248	0.000000293140650	0.106962765000000	0.634500000000000
359	0.000000011701248	0.000000363000000	0.066539579600000	0.634500000000000
360	0.000000011701248	0.000000363000000	0.072437235000000	0.512390475000000
361	0.000000011701248	0.000000363000000	0.072437235000000	0.634500000000000
362	0.000000011701248	0.000000363000000	0.072437235000000	0.756609525000000
363	0.000000011701248	0.000000363000000	0.089700000000000	0.470672946000000
364	0.000000011701248	0.000000363000000	0.089700000000000	0.512390475000000
365	0.000000011701248	0.000000363000000	0.089700000000000	0.634500000000000
366	0.000000011701248	0.000000363000000	0.089700000000000	0.756609525000000
367	0.000000011701248	0.000000363000000	0.089700000000000	0.798327054000000
368	0.000000011701248	0.000000363000000	0.106962765000000	0.512390475000000
369	0.000000011701248	0.000000363000000	0.106962765000000	0.634500000000000
370	0.000000011701248	0.000000363000000	0.106962765000000	0.756609525000000

371	0.000000011701248	0.000000363000000	0.112860420400000	0.634500000000000
372	0.000000011701248	0.000000432859350	0.072437235000000	0.634500000000000
373	0.000000011701248	0.000000432859350	0.089700000000000	0.512390475000000
374	0.000000011701248	0.000000432859350	0.089700000000000	0.634500000000000
375	0.000000011701248	0.000000432859350	0.089700000000000	0.756609525000000
376	0.000000011701248	0.000000432859350	0.106962765000000	0.634500000000000
377	0.000000011701248	0.000000456726116	0.089700000000000	0.634500000000000
378	0.000000011969522	0.000000293140650	0.089700000000000	0.634500000000000
379	0.000000011969522	0.000000363000000	0.072437235000000	0.634500000000000
380	0.000000011969522	0.000000363000000	0.089700000000000	0.512390475000000
381	0.000000011969522	0.000000363000000	0.089700000000000	0.634500000000000
382	0.000000011969522	0.000000363000000	0.089700000000000	0.756609525000000
383	0.000000011969522	0.000000363000000	0.106962765000000	0.634500000000000
384	0.000000011969522	0.000000432859350	0.089700000000000	0.634500000000000
385	0.000000012109158	0.000000363000000	0.089700000000000	0.634500000000000
