# Supplementary material for: Development of an Agent-Based Model (ABM) to Simulate the Immune System and Integration of a Regression Method to Estimate the Key ABM Parameters by Fitting the Experimental Data
Source: PLoS One. 2015 Nov 4;10(11):e0141295. doi: 10.1371/journal.pone.0141295 (PMC4633145; doi:10.1371/journal.pone.0141295)
Supplement: S7 Table — (PDF) [file pone.0141295.s008.pdf]

S7 Table.Output data sets of model 41x9

samples	output data value of ABM with 13 time points												
	1	2	3	4	5	6	7	8	9	10	11	12	13
1	3.24	4.25	5.98	7.02	6.69	6.26	5.83	5.41	5.04	4.67	4.29	3.94	3.59
2	3.24	4.21	5.93	7.02	6.69	6.26	5.84	5.4	5.03	4.66	4.26	3.86	3.48
3	3.24	4.2	5.92	7.02	6.69	6.26	5.84	5.41	5.03	4.65	4.27	3.86	3.49
4	3.24	4.23	5.95	7.02	6.69	6.27	5.84	5.4	5.04	4.66	4.3	3.94	3.59
5	3.24	4.28	6	7.02	6.69	6.26	5.83	5.4	5.02	4.64	4.28	3.88	3.55
6	3.24	4.26	5.97	7.02	6.69	6.26	5.83	5.4	5.03	4.67	4.3	3.93	3.58
7	3.24	4.25	5.98	7.02	6.69	6.26	5.83	5.4	5.02	4.64	4.28	3.87	3.51
8	3.24	4.27	5.99	7.02	6.69	6.26	5.83	5.41	5.04	4.66	4.28	3.9	3.51
9	3.24	4.26	5.97	7.02	6.69	6.26	5.83	5.4	5.03	4.65	4.29	3.91	3.51
10	3.24	3.7	4.73	6.23	6.86	6.43	6	5.57	5.2	4.83	4.47	4.08	3.73
11	3.24	3.76	4.81	6.3	6.85	6.42	5.99	5.56	5.19	4.81	4.44	4.08	3.73
12	3.24	3.75	4.8	6.3	6.85	6.42	5.99	5.56	5.18	4.81	4.42	4.04	3.67
13	3.24	3.7	4.73	6.24	6.86	6.43	6	5.57	5.19	4.82	4.44	4.08	3.67
14	3.24	3.65	4.7	6.2	6.86	6.43	6.01	5.58	5.2	4.82	4.45	4.09	3.74
15	3.24	3.68	4.74	6.25	6.85	6.43	6	5.57	5.2	4.83	4.46	4.09	3.7
16	3.24	3.64	4.67	6.18	6.86	6.44	6.01	5.58	5.2	4.83	4.46	4.08	3.67
17	3.24	3.69	4.74	6.24	6.85	6.43	6	5.57	5.19	4.81	4.44	4.06	3.64
18	3.24	3.72	4.76	6.26	6.86	6.43	6	5.57	5.2	4.83	4.45	4.09	3.73
19	3.24	4.24	5.98	7.07	6.95	6.77	6.59	6.41	6.26	6.1	5.95	5.79	5.64
20	3.24	4.2	5.95	7.07	6.95	6.77	6.59	6.42	6.26	6.1	5.95	5.79	5.64
21	3.24	4.25	5.98	7.07	6.95	6.77	6.59	6.42	6.26	6.11	5.95	5.8	5.65
22	3.24	4.31	6.04	7.07	6.95	6.77	6.59	6.41	6.26	6.1	5.94	5.79	5.63
23	3.24	4.24	5.98	7.07	6.95	6.77	6.6	6.42	6.26	6.11	5.95	5.8	5.64
24	3.24	4.2	5.95	7.07	6.95	6.77	6.6	6.42	6.26	6.1	5.95	5.79	5.64
25	3.24	4.2	5.94	7.07	6.95	6.77	6.6	6.42	6.26	6.11	5.95	5.8	5.64
26	3.24	4.31	6.05	7.07	6.95	6.77	6.59	6.41	6.26	6.1	5.94	5.79	5.63
27	3.24	4.26	5.99	7.07	6.95	6.77	6.59	6.41	6.26	6.1	5.95	5.8	5.64
28	3.24	4.46	6.28	7.27	7.12	6.72	6.3	5.87	5.49	5.12	4.73	4.34	3.95
29	3.24	4.47	6.28	7.27	7.12	6.72	6.3	5.87	5.5	5.13	4.76	4.4	4.03
30	3.24	4.45	6.27	7.27	7.12	6.72	6.3	5.87	5.5	5.13	4.75	4.38	4
31	3.24	4.46	6.28	7.27	7.12	6.72	6.3	5.86	5.49	5.12	4.75	4.36	3.99
32	3.24	4.48	6.29	7.27	7.12	6.72	6.3	5.87	5.5	5.13	4.74	4.34	3.92
33	3.24	4.48	6.3	7.27	7.12	6.72	6.3	5.87	5.5	5.13	4.77	4.39	4.01
34	3.24	4.46	6.28	7.27	7.12	6.72	6.3	5.88	5.51	5.13	4.74	4.35	3.97
35	3.24	4.51	6.32	7.27	7.12	6.72	6.3	5.87	5.49	5.12	4.75	4.38	3.99
36	3.24	4.43	6.25	7.26	7.12	6.72	6.3	5.87	5.49	5.12	4.76	4.4	4.01
37	3.24	4.21	5.95	7.02	6.69	6.26	5.83	5.41	5.03	4.66	4.25	3.88	3.49
38	3.24	4.28	6.02	7.02	6.69	6.26	5.83	5.4	5.03	4.67	4.28	3.89	3.57
39	3.24	4.27	6	7.02	6.69	6.26	5.83	5.41	5.04	4.67	4.31	3.92	3.56
40	3.24	4.23	5.96	7.02	6.69	6.26	5.83	5.4	5.02	4.65	4.28	3.89	3.51
41	3.24	4.17	5.9	7.02	6.69	6.27	5.84	5.41	5.03	4.66	4.29	3.92	3.54
42	3.24	4.19	5.93	7.02	6.69	6.26	5.84	5.41	5.03	4.66	4.3	3.93	3.54
43	3.24	4.22	5.95	7.02	6.69	6.26	5.83	5.4	5.02	4.66	4.28	3.92	3.54
44	3.24	4.31	6.03	7.02	6.69	6.26	5.83	5.4	5.02	4.64	4.25	3.91	3.58

45	3.24	4.22	5.94	7.02	6.69	6.26	5.83	5.41	5.04	4.65	4.3	3.93	3.55
46	3.24	4.01	5.63	6.85	6.49	6.06	5.64	5.21	4.84	4.47	4.1	3.73	3.33
47	3.24	3.96	5.58	6.86	6.49	6.06	5.63	5.2	4.83	4.45	4.07	3.69	3.32
48	3.24	3.92	5.55	6.86	6.49	6.06	5.64	5.21	4.84	4.46	4.09	3.71	3.33
49	3.24	3.96	5.58	6.86	6.49	6.07	5.64	5.21	4.83	4.45	4.06	3.67	3.28
50	3.24	4.02	5.63	6.86	6.49	6.06	5.63	5.21	4.83	4.45	4.09	3.7	3.3
51	3.24	3.97	5.59	6.86	6.49	6.06	5.63	5.21	4.83	4.46	4.1	3.74	3.35
52	3.24	4.03	5.64	6.85	6.49	6.06	5.63	5.2	4.83	4.46	4.07	3.68	3.28
53	3.24	4.02	5.64	6.86	6.49	6.06	5.63	5.2	4.83	4.44	4.07	3.67	3.29
54	3.24	3.93	5.55	6.86	6.49	6.06	5.64	5.21	4.84	4.48	4.1	3.74	3.38
55	3.24	4.29	6	6.97	6.42	5.73	5.04	4.36	3.7	3.09	2.57	1.76	0
56	3.24	4.26	5.95	6.97	6.42	5.73	5.05	4.38	3.74	3.09	2.51	1.91	0
57	3.24	4.22	5.93	6.97	6.42	5.74	5.05	4.35	3.74	3.1	2.57	1.46	0
58	3.24	4.18	5.88	6.98	6.43	5.74	5.06	4.38	3.78	3.17	2.59	2.1	2.01
59	3.24	4.24	5.94	6.97	6.42	5.74	5.06	4.36	3.75	3.23	2.67	2.14	1.67
60	3.24	4.21	5.93	6.97	6.42	5.73	5.04	4.34	3.77	3.26	2.56	1.86	0.3
61	3.24	4.22	5.92	6.98	6.43	5.74	5.06	4.37	3.76	3.07	2.27	2.02	1.46
62	3.24	4.27	5.97	6.97	6.42	5.73	5.04	4.35	3.75	3.11	2.6	2.03	1.7
63	3.24	4.24	5.94	6.97	6.42	5.73	5.04	4.36	3.81	3.16	2.68	2.2	1.32
64	3.24	4.59	6.71	7	6.64	6.21	5.78	5.35	4.97	4.6	4.21	3.81	3.44
65	3.24	4.62	6.73	7	6.64	6.21	5.78	5.35	4.98	4.6	4.24	3.86	3.46
66	3.24	4.62	6.73	7	6.64	6.21	5.78	5.35	4.97	4.6	4.25	3.87	3.49
67	3.24	4.59	6.71	7	6.64	6.21	5.78	5.35	4.98	4.61	4.23	3.81	3.44
68	3.24	4.6	6.71	7	6.64	6.21	5.78	5.36	4.98	4.62	4.25	3.88	3.51
69	3.24	4.63	6.74	7	6.64	6.21	5.78	5.36	4.98	4.59	4.21	3.88	3.53
70	3.24	4.68	6.77	7	6.63	6.2	5.78	5.36	4.98	4.6	4.21	3.84	3.48
71	3.24	4.54	6.67	7	6.64	6.21	5.79	5.36	4.99	4.61	4.22	3.81	3.46
72	3.24	4.61	6.73	7	6.64	6.21	5.78	5.36	4.98	4.61	4.23	3.86	3.52
73	3.24	3.35	3.98	4.97	6.89	6.68	6.26	5.83	5.45	5.07	4.69	4.32	3.94
74	3.24	3.32	4	4.98	6.89	6.68	6.26	5.83	5.46	5.08	4.71	4.33	3.92
75	3.24	3.42	4.12	5.13	6.93	6.66	6.23	5.81	5.43	5.06	4.68	4.33	3.95
76	3.24	3.32	4.02	5.04	6.9	6.67	6.25	5.82	5.45	5.08	4.71	4.34	3.97
77	3.24	3.34	4.05	5.04	6.9	6.67	6.24	5.81	5.44	5.06	4.69	4.31	3.94
78	3.24	3.46	4.17	5.17	6.94	6.65	6.22	5.79	5.42	5.04	4.65	4.28	3.92
79	3.24	3.47	4.16	5.16	6.93	6.65	6.23	5.8	5.42	5.05	4.66	4.29	3.92
80	3.24	3.44	4.14	5.12	6.92	6.66	6.23	5.8	5.42	5.04	4.68	4.31	3.93
81	3.24	3.44	4.1	5.1	6.92	6.67	6.24	5.81	5.43	5.06	4.68	4.32	3.95
82	3.24	3.69	4.78	6.31	7.02	6.84	6.66	6.48	6.33	6.17	6.02	5.86	5.7
83	3.24	3.68	4.73	6.28	7.02	6.84	6.66	6.49	6.33	6.17	6.02	5.86	5.7
84	3.24	3.66	4.73	6.27	7.02	6.84	6.66	6.48	6.33	6.17	6.02	5.86	5.7
85	3.24	3.68	4.71	6.26	7.02	6.84	6.66	6.49	6.33	6.17	6.02	5.86	5.7
86	3.24	3.77	4.84	6.37	7.01	6.84	6.66	6.48	6.33	6.17	6.02	5.86	5.71
87	3.24	3.68	4.75	6.28	7.02	6.84	6.66	6.48	6.33	6.17	6.02	5.86	5.71
88	3.24	3.68	4.76	6.29	7.02	6.84	6.66	6.48	6.33	6.17	6.01	5.86	5.71
89	3.24	3.66	4.76	6.3	7.02	6.84	6.66	6.48	6.33	6.17	6.02	5.86	5.71
90	3.24	3.7	4.76	6.3	7.02	6.84	6.66	6.48	6.33	6.17	6.02	5.86	5.71
91	3.24	3.96	5.15	6.78	7.22	6.85	6.42	5.99	5.62	5.25	4.87	4.49	4.12
92	3.24	3.9	5.08	6.73	7.23	6.86	6.43	6	5.63	5.26	4.9	4.52	4.15
93	3.24	3.97	5.14	6.78	7.22	6.85	6.42	6	5.62	5.24	4.87	4.48	4.09
94	3.24	3.95	5.11	6.76	7.22	6.85	6.43	6	5.62	5.25	4.87	4.47	4.11
95	3.24	3.93	5.09	6.74	7.22	6.85	6.43	6	5.63	5.27	4.91	4.52	4.14
96	3.24	3.9	5.09	6.74	7.22	6.85	6.43	6.01	5.63	5.26	4.89	4.53	4.18
97	3.24	4.01	5.19	6.82	7.22	6.84	6.42	6	5.63	5.25	4.88	4.49	4.07
98	3.24	3.97	5.15	6.79	7.22	6.85	6.43	6	5.62	5.24	4.86	4.48	4.07

99	3.24	3.93	5.1	6.75	7.22	6.85	6.43	6	5.62	5.25	4.87	4.47	4.08
100	3.24	3.67	4.72	6.23	6.86	6.43	6	5.57	5.2	4.82	4.44	4.06	3.73
101	3.24	3.6	4.64	6.15	6.87	6.44	6.01	5.58	5.21	4.84	4.46	4.08	3.72
102	3.24	3.67	4.71	6.2	6.86	6.43	6	5.58	5.21	4.83	4.46	4.11	3.73
103	3.24	3.56	4.59	6.11	6.87	6.44	6.01	5.58	5.21	4.84	4.45	4.08	3.66
104	3.24	3.68	4.72	6.23	6.86	6.43	6	5.57	5.2	4.82	4.45	4.09	3.69
105	3.24	3.67	4.74	6.24	6.86	6.43	6	5.57	5.2	4.83	4.46	4.1	3.76
106	3.24	3.58	4.61	6.12	6.87	6.44	6.02	5.59	5.22	4.83	4.46	4.1	3.73
107	3.24	3.64	4.68	6.19	6.86	6.44	6.01	5.58	5.21	4.83	4.46	4.08	3.72
108	3.24	3.67	4.71	6.21	6.86	6.43	6	5.57	5.2	4.81	4.42	4.07	3.73
109	3.24	3.38	4.3	5.63	6.71	6.28	5.85	5.43	5.05	4.68	4.3	3.9	3.53
110	3.24	3.42	4.34	5.69	6.71	6.28	5.85	5.42	5.05	4.67	4.3	3.94	3.58
111	3.24	3.46	4.37	5.69	6.71	6.27	5.85	5.42	5.04	4.66	4.3	3.93	3.53
112	3.24	3.41	4.34	5.67	6.71	6.28	5.85	5.42	5.04	4.67	4.3	3.89	3.54
113	3.24	3.37	4.29	5.63	6.71	6.29	5.86	5.43	5.05	4.67	4.3	3.92	3.55
114	3.24	3.43	4.32	5.67	6.71	6.28	5.85	5.42	5.05	4.66	4.29	3.91	3.54
115	3.24	3.41	4.35	5.69	6.71	6.28	5.85	5.42	5.05	4.68	4.29	3.91	3.52
116	3.24	3.39	4.34	5.69	6.71	6.28	5.85	5.42	5.04	4.65	4.28	3.91	3.52
117	3.24	3.44	4.39	5.74	6.7	6.27	5.84	5.41	5.03	4.66	4.29	3.92	3.55
118	3.24	3.65	4.69	6.15	6.71	6.02	5.33	4.64	4.05	3.44	2.95	2.24	1.69
119	3.24	3.67	4.69	6.16	6.71	6.02	5.34	4.64	4.05	3.46	2.71	2.35	1.75
120	3.24	3.62	4.66	6.13	6.71	6.03	5.35	4.67	4.06	3.47	2.87	2.01	1.46
121	3.24	3.65	4.7	6.16	6.71	6.02	5.33	4.63	4.03	3.39	2.82	2.32	1.6
122	3.24	3.66	4.67	6.15	6.71	6.02	5.33	4.64	4.07	3.5	2.75	1.9	1.68
123	3.24	3.69	4.72	6.18	6.7	6.02	5.32	4.61	3.99	3.38	2.72	2.03	0
124	3.24	3.7	4.73	6.2	6.7	6.02	5.33	4.64	4.01	3.45	2.94	2.31	1.98
125	3.24	3.69	4.73	6.19	6.7	6.02	5.33	4.65	4.06	3.47	3.01	2.42	2.12
126	3.24	3.72	4.74	6.2	6.7	6.01	5.32	4.64	4.05	3.44	2.84	2.23	1.85
127	3.24	4.25	6	7.09	7.04	6.94	6.85	6.75	6.67	6.59	6.51	6.43	6.34
128	3.24	4.27	6.03	7.09	7.04	6.94	6.85	6.75	6.67	6.59	6.51	6.42	6.34
129	3.24	4.29	6.02	7.09	7.04	6.94	6.85	6.75	6.67	6.59	6.51	6.42	6.34
130	3.24	4.23	5.98	7.09	7.04	6.94	6.85	6.75	6.67	6.59	6.51	6.42	6.34
131	3.24	4.34	6.07	7.09	7.04	6.94	6.85	6.75	6.67	6.59	6.51	6.42	6.34
132	3.24	4.26	6.01	7.09	7.04	6.94	6.85	6.76	6.67	6.59	6.51	6.42	6.34
133	3.24	4.24	5.99	7.09	7.04	6.94	6.85	6.76	6.67	6.59	6.51	6.42	6.34
134	3.24	4.25	5.99	7.09	7.04	6.94	6.85	6.75	6.67	6.59	6.51	6.43	6.34
135	3.24	4.24	5.98	7.09	7.04	6.94	6.85	6.75	6.67	6.59	6.51	6.42	6.34
136	3.24	4.51	6.34	7.32	7.34	7.18	7	6.82	6.67	6.51	6.36	6.2	6.04
137	3.24	4.5	6.33	7.31	7.34	7.18	7	6.82	6.67	6.51	6.35	6.2	6.05
138	3.24	4.51	6.34	7.32	7.34	7.18	7	6.82	6.66	6.51	6.35	6.2	6.04
139	3.24	4.52	6.36	7.32	7.34	7.18	7	6.82	6.66	6.51	6.35	6.2	6.04
140	3.24	4.47	6.31	7.31	7.34	7.18	7	6.82	6.66	6.51	6.35	6.2	6.04
141	3.24	4.51	6.35	7.32	7.34	7.18	7	6.82	6.67	6.51	6.35	6.2	6.04
142	3.24	4.49	6.32	7.31	7.34	7.18	7	6.82	6.67	6.51	6.35	6.2	6.04
143	3.24	4.47	6.29	7.31	7.34	7.18	7	6.82	6.67	6.51	6.35	6.2	6.04
144	3.24	4.51	6.35	7.32	7.34	7.18	7	6.82	6.66	6.51	6.36	6.2	6.05
145	3.24	4.26	6	7.07	6.95	6.77	6.59	6.42	6.26	6.1	5.95	5.79	5.64
146	3.24	4.25	5.99	7.07	6.95	6.77	6.59	6.42	6.26	6.1	5.94	5.79	5.63
147	3.24	4.19	5.94	7.07	6.95	6.77	6.59	6.42	6.26	6.11	5.95	5.79	5.64
148	3.24	4.27	6.01	7.07	6.95	6.77	6.59	6.41	6.26	6.1	5.95	5.79	5.64
149	3.24	4.25	6	7.07	6.95	6.77	6.59	6.41	6.26	6.1	5.95	5.79	5.64
150	3.24	4.21	5.94	7.07	6.95	6.77	6.6	6.42	6.26	6.11	5.95	5.8	5.64
151	3.24	4.28	6.03	7.07	6.95	6.77	6.59	6.42	6.26	6.11	5.95	5.79	5.64
152	3.24	4.22	5.97	7.07	6.95	6.77	6.59	6.41	6.26	6.1	5.94	5.79	5.63

153	3.24	4.24	5.97	7.07	6.95	6.77	6.59	6.41	6.26	6.1	5.95	5.79	5.63
154	3.24	4	5.65	6.9	6.75	6.57	6.39	6.22	6.06	5.91	5.75	5.6	5.45
155	3.24	3.93	5.56	6.9	6.75	6.58	6.4	6.22	6.06	5.91	5.75	5.6	5.44
156	3.24	3.94	5.58	6.9	6.75	6.58	6.4	6.22	6.06	5.91	5.75	5.6	5.44
157	3.24	3.99	5.64	6.9	6.75	6.57	6.39	6.22	6.06	5.9	5.75	5.59	5.44
158	3.24	3.89	5.53	6.9	6.75	6.58	6.4	6.22	6.07	5.91	5.75	5.6	5.44
159	3.24	3.94	5.57	6.9	6.75	6.57	6.4	6.22	6.07	5.91	5.76	5.6	5.45
160	3.24	3.95	5.61	6.9	6.75	6.58	6.4	6.22	6.06	5.91	5.75	5.59	5.44
161	3.24	3.95	5.6	6.9	6.75	6.57	6.4	6.22	6.06	5.91	5.75	5.59	5.43
162	3.24	4.02	5.66	6.9	6.75	6.57	6.4	6.22	6.06	5.91	5.75	5.59	5.44
163	3.24	4.52	6.37	7.37	7.41	7.12	6.76	6.36	6	5.62	5.24	4.88	4.51
164	3.24	4.54	6.4	7.37	7.4	7.12	6.75	6.35	5.99	5.63	5.27	4.9	4.53
165	3.24	4.54	6.39	7.37	7.41	7.12	6.75	6.36	5.99	5.63	5.26	4.88	4.49
166	3.24	4.56	6.42	7.37	7.4	7.12	6.75	6.35	5.99	5.62	5.26	4.89	4.52
167	3.24	4.55	6.4	7.37	7.4	7.12	6.75	6.36	5.99	5.63	5.26	4.89	4.5
168	3.24	4.59	6.44	7.37	7.4	7.12	6.75	6.35	5.99	5.62	5.25	4.87	4.5
169	3.24	4.58	6.42	7.37	7.4	7.12	6.75	6.36	6	5.63	5.27	4.9	4.54
170	3.24	4.53	6.38	7.37	7.41	7.12	6.75	6.35	5.99	5.62	5.24	4.86	4.49
171	3.24	4.56	6.42	7.37	7.4	7.12	6.75	6.35	5.99	5.62	5.24	4.87	4.5
172	3.24	4.43	6.26	7.26	7.12	6.72	6.29	5.87	5.5	5.13	4.75	4.38	4.02
173	3.24	4.48	6.3	7.27	7.12	6.72	6.3	5.87	5.49	5.13	4.76	4.38	3.98
174	3.24	4.46	6.28	7.27	7.12	6.72	6.29	5.87	5.49	5.11	4.74	4.39	4.03
175	3.24	4.5	6.31	7.27	7.12	6.72	6.3	5.87	5.5	5.12	4.74	4.37	3.97
176	3.24	4.47	6.28	7.27	7.12	6.72	6.3	5.88	5.5	5.12	4.74	4.36	4.01
177	3.24	4.5	6.32	7.27	7.12	6.72	6.3	5.87	5.49	5.12	4.75	4.36	3.97
178	3.24	4.47	6.29	7.27	7.12	6.72	6.29	5.86	5.49	5.11	4.74	4.36	3.96
179	3.24	4.41	6.23	7.26	7.13	6.73	6.3	5.88	5.51	5.13	4.74	4.37	3.99
180	3.24	4.48	6.31	7.27	7.12	6.72	6.3	5.87	5.49	5.11	4.73	4.36	4
181	3.24	4.24	5.96	7.02	6.69	6.26	5.83	5.4	5.02	4.65	4.28	3.9	3.52
182	3.24	4.33	6.05	7.02	6.68	6.26	5.83	5.4	5.03	4.66	4.28	3.91	3.52
183	3.24	4.25	5.97	7.02	6.69	6.26	5.83	5.41	5.03	4.66	4.29	3.93	3.51
184	3.24	4.23	5.95	7.02	6.69	6.26	5.83	5.41	5.02	4.65	4.29	3.94	3.56
185	3.24	4.25	5.98	7.02	6.69	6.26	5.83	5.4	5.03	4.64	4.28	3.9	3.51
186	3.24	4.28	6.01	7.02	6.69	6.26	5.83	5.41	5.03	4.65	4.28	3.88	3.49
187	3.24	4.26	5.98	7.02	6.69	6.26	5.83	5.4	5.02	4.64	4.24	3.87	3.45
188	3.24	4.23	5.96	7.02	6.69	6.26	5.83	5.4	5.02	4.65	4.29	3.92	3.5
189	3.24	4.24	5.96	7.02	6.69	6.26	5.83	5.4	5.03	4.65	4.27	3.9	3.53
190	3.24	3.98	5.61	6.86	6.49	6.06	5.63	5.2	4.82	4.46	4.1	3.72	3.37
191	3.24	3.88	5.51	6.86	6.5	6.07	5.64	5.22	4.84	4.47	4.07	3.68	3.28
192	3.24	4.02	5.64	6.85	6.49	6.06	5.63	5.21	4.83	4.47	4.08	3.71	3.39
193	3.24	4.03	5.65	6.85	6.49	6.06	5.63	5.2	4.82	4.44	4.06	3.69	3.31
194	3.24	3.92	5.55	6.86	6.49	6.06	5.64	5.21	4.83	4.46	4.09	3.72	3.34
195	3.24	4	5.62	6.85	6.49	6.06	5.63	5.2	4.83	4.44	4.06	3.66	3.26
196	3.24	3.93	5.57	6.86	6.49	6.06	5.64	5.2	4.82	4.45	4.07	3.7	3.33
197	3.24	3.9	5.53	6.86	6.5	6.07	5.64	5.2	4.83	4.44	4.07	3.7	3.35
198	3.24	3.94	5.54	6.86	6.5	6.07	5.64	5.21	4.83	4.46	4.08	3.7	3.33
199	3.24	3.83	5.42	6.82	6.45	6.02	5.59	5.17	4.79	4.42	4.04	3.62	3.26
200	3.24	3.87	5.46	6.81	6.45	6.02	5.59	5.16	4.79	4.41	4.04	3.66	3.29
201	3.24	3.83	5.42	6.82	6.45	6.02	5.59	5.16	4.79	4.42	4.04	3.67	3.29
202	3.24	3.8	5.38	6.82	6.45	6.02	5.59	5.16	4.79	4.42	4.05	3.67	3.34
203	3.24	3.9	5.47	6.81	6.44	6.01	5.58	5.16	4.78	4.4	4.04	3.68	3.28
204	3.24	3.82	5.39	6.82	6.45	6.02	5.59	5.16	4.78	4.4	4.04	3.69	3.31
205	3.24	3.87	5.46	6.81	6.45	6.02	5.59	5.16	4.78	4.4	4.02	3.67	3.32
206	3.24	3.96	5.56	6.81	6.44	6.01	5.58	5.15	4.77	4.38	4.01	3.61	3.21

207	3.24	3.96	5.55	6.81	6.44	6.01	5.58	5.15	4.77	4.39	4	3.61	3.25
208	3.24	4.49	6.29	7.22	6.91	6.28	5.6	4.92	4.3	3.66	3	2.49	1.68
209	3.24	4.45	6.26	7.22	6.91	6.28	5.6	4.91	4.33	3.72	3.19	2.69	1.99
210	3.24	4.48	6.29	7.22	6.91	6.28	5.59	4.89	4.31	3.71	3.04	2.04	0.9
211	3.24	4.5	6.31	7.22	6.91	6.28	5.6	4.9	4.28	3.71	3.09	2.45	1.45
212	3.24	4.45	6.25	7.22	6.92	6.28	5.61	4.92	4.33	3.71	3.1	2.63	1.59
213	3.24	4.44	6.24	7.22	6.92	6.28	5.6	4.92	4.32	3.75	3.17	2.54	1.91
214	3.24	4.47	6.27	7.22	6.91	6.28	5.6	4.91	4.29	3.68	2.99	2.29	1.86
215	3.24	4.45	6.26	7.22	6.91	6.28	5.6	4.91	4.3	3.76	3.15	2.65	2.25
216	3.24	4.5	6.3	7.22	6.91	6.28	5.59	4.91	4.34	3.78	3.21	2.62	2.16
217	3.24	4.19	5.88	6.98	6.43	5.74	5.06	4.36	3.75	3.15	2.58	2.05	1.43
218	3.24	4.23	5.94	6.97	6.42	5.74	5.05	4.36	3.75	3.15	2.58	2.05	1.43
219	3.24	4.24	5.94	6.97	6.42	5.73	5.05	4.36	3.78	3.24	2.57	2.05	1.23
220	3.24	4.23	5.94	6.97	6.42	5.74	5.05	4.36	3.73	3.05	2.44	1.77	0.85
221	3.24	4.2	5.89	6.98	6.43	5.74	5.05	4.35	3.74	3.14	2.54	2.12	1.4
222	3.24	4.22	5.92	6.98	6.43	5.74	5.06	4.36	3.75	3.22	2.56	1.85	1.41
223	3.24	4.23	5.94	6.97	6.42	5.74	5.05	4.35	3.72	3.11	2.42	1.81	1.32
224	3.24	4.17	5.88	6.98	6.43	5.74	5.06	4.38	3.77	3.17	2.43	1.78	1.38
225	3.24	4.28	5.97	6.97	6.42	5.73	5.03	4.35	3.74	3.14	2.54	2.04	0
226	3.24	3.96	5.56	6.81	6.22	5.53	4.85	4.15	3.51	2.96	2.1	1.48	0
227	3.24	3.93	5.54	6.82	6.23	5.54	4.86	4.17	3.55	2.89	2.31	1.65	1.2
228	3.24	3.96	5.56	6.82	6.22	5.53	4.84	4.17	3.57	2.94	2.36	1.64	1.26
229	3.24	3.87	5.47	6.82	6.23	5.55	4.86	4.18	3.55	2.94	2.34	1.28	0
230	3.24	3.95	5.55	6.82	6.22	5.54	4.84	4.15	3.58	2.94	2.33	1.72	1.18
231	3.24	3.94	5.54	6.82	6.23	5.54	4.84	4.16	3.58	2.96	2.48	1.92	1.69
232	3.24	3.99	5.59	6.81	6.22	5.54	4.85	4.15	3.58	3.03	2.33	1.79	0
233	3.24	3.97	5.55	6.82	6.22	5.54	4.84	4.14	3.53	2.88	2.2	1.51	0
234	3.24	3.96	5.57	6.81	6.22	5.53	4.85	4.16	3.55	2.98	2.4	1.72	1.51
235	3.24	4.25	5.94	6.96	6.33	5.55	4.79	4.01	3.29	2.43	2.01	1.65	0
236	3.24	4.3	5.99	6.95	6.33	5.55	4.77	3.98	3.27	2.62	2.11	1.9	1.34
237	3.24	4.22	5.92	6.96	6.33	5.55	4.77	3.98	3.29	2.71	1.76	0	0
238	3.24	4.24	5.93	6.96	6.33	5.55	4.78	3.99	3.25	2.3	0	0	0
239	3.24	4.24	5.93	6.96	6.33	5.55	4.78	3.97	3.24	2.67	2.27	1.68	1.36
240	3.24	4.2	5.89	6.96	6.34	5.56	4.77	3.97	3.3	2.63	0.95	0	0
241	3.24	4.18	5.88	6.96	6.34	5.56	4.78	3.99	3.29	2.53	1.96	1.34	0
242	3.24	4.22	5.92	6.96	6.33	5.56	4.77	4.02	3.42	2.84	2.29	1.43	0
243	3.24	4.25	5.94	6.96	6.33	5.55	4.77	4.02	3.38	2.72	1.83	0.3	0
244	3.24	4.61	6.73	7.07	6.93	6.75	6.57	6.4	6.24	6.08	5.93	5.77	5.61
245	3.24	4.65	6.77	7.07	6.93	6.75	6.57	6.39	6.24	6.08	5.93	5.77	5.61
246	3.24	4.67	6.77	7.07	6.93	6.75	6.57	6.4	6.24	6.09	5.93	5.78	5.63
247	3.24	4.68	6.79	7.07	6.93	6.75	6.57	6.39	6.24	6.08	5.92	5.77	5.62
248	3.24	4.63	6.75	7.07	6.93	6.75	6.57	6.39	6.24	6.08	5.93	5.77	5.61
249	3.24	4.62	6.75	7.07	6.93	6.75	6.57	6.39	6.24	6.08	5.92	5.77	5.61
250	3.24	4.62	6.74	7.07	6.93	6.75	6.57	6.39	6.24	6.08	5.93	5.77	5.61
251	3.24	4.65	6.77	7.07	6.93	6.75	6.57	6.39	6.24	6.08	5.93	5.77	5.62
252	3.24	4.58	6.71	7.07	6.93	6.75	6.57	6.4	6.24	6.08	5.93	5.77	5.62
253	3.24	4.83	6.92	7.29	7.09	6.68	6.25	5.82	5.44	5.06	4.7	4.3	3.9
254	3.24	4.82	6.92	7.29	7.09	6.68	6.26	5.82	5.45	5.07	4.69	4.28	3.89
255	3.24	4.83	6.92	7.29	7.08	6.68	6.25	5.82	5.45	5.08	4.71	4.34	3.95
256	3.24	4.85	6.92	7.29	7.08	6.68	6.25	5.82	5.45	5.07	4.72	4.34	3.95
257	3.24	4.87	6.93	7.29	7.08	6.68	6.25	5.82	5.45	5.07	4.71	4.35	3.97
258	3.24	4.81	6.91	7.29	7.09	6.68	6.25	5.83	5.45	5.08	4.7	4.33	3.97
259	3.24	4.87	6.93	7.29	7.08	6.68	6.25	5.83	5.45	5.08	4.72	4.36	4
260	3.24	4.82	6.92	7.29	7.09	6.68	6.26	5.83	5.46	5.09	4.71	4.31	3.9

261	3.24	4.87	6.93	7.29	7.08	6.68	6.25	5.82	5.45	5.08	4.7	4.3	3.93
262	3.24	4.65	6.75	7	6.64	6.21	5.78	5.35	4.98	4.6	4.22	3.87	3.48
263	3.24	4.58	6.7	7	6.64	6.21	5.78	5.35	4.97	4.6	4.23	3.87	3.51
264	3.24	4.62	6.73	7	6.64	6.21	5.78	5.35	4.98	4.59	4.23	3.84	3.46
265	3.24	4.68	6.77	7	6.63	6.21	5.78	5.35	4.98	4.61	4.26	3.87	3.38
266	3.24	4.62	6.73	7	6.64	6.21	5.78	5.34	4.96	4.59	4.24	3.9	3.5
267	3.24	4.61	6.72	7	6.64	6.21	5.78	5.35	4.97	4.61	4.2	3.82	3.41
268	3.24	4.63	6.73	7	6.64	6.21	5.79	5.35	4.98	4.6	4.23	3.88	3.45
269	3.24	4.63	6.74	7	6.64	6.21	5.78	5.35	4.97	4.6	4.24	3.86	3.48
270	3.24	4.62	6.72	7	6.64	6.21	5.78	5.35	4.97	4.6	4.22	3.84	3.46
271	3.24	4.35	6.42	6.8	6.43	6	5.57	5.14	4.77	4.39	4.01	3.66	3.27
272	3.24	4.35	6.41	6.8	6.43	6	5.57	5.15	4.77	4.39	4.03	3.66	3.29
273	3.24	4.32	6.38	6.81	6.43	6	5.57	5.14	4.76	4.38	4.01	3.64	3.24
274	3.24	4.37	6.44	6.8	6.43	6	5.57	5.14	4.76	4.39	4.01	3.63	3.23
275	3.24	4.41	6.47	6.8	6.43	6	5.57	5.15	4.77	4.41	4.05	3.68	3.3
276	3.24	4.33	6.4	6.8	6.43	6	5.57	5.15	4.77	4.41	4.04	3.69	3.31
277	3.24	4.35	6.42	6.8	6.43	6	5.57	5.15	4.77	4.4	4.01	3.63	3.2
278	3.24	4.34	6.41	6.8	6.43	6	5.57	5.15	4.77	4.39	4.01	3.66	3.31
279	3.24	4.32	6.39	6.81	6.43	6	5.57	5.14	4.77	4.39	4.02	3.67	3.19
280	3.24	4.67	6.75	6.92	6.33	5.64	4.96	4.26	3.68	3.07	2.4	1.81	0
281	3.24	4.61	6.7	6.92	6.34	5.65	4.97	4.27	3.63	2.99	2.53	2	1.86
282	3.24	4.56	6.67	6.93	6.34	5.66	4.96	4.27	3.65	3.05	2.44	2.01	1.4
283	3.24	4.62	6.71	6.92	6.34	5.65	4.95	4.26	3.68	3.13	2.54	2.15	1.63
284	3.24	4.68	6.76	6.92	6.33	5.65	4.96	4.27	3.62	3.01	2.3	0	0
285	3.24	4.65	6.73	6.92	6.34	5.65	4.96	4.27	3.66	3.06	2.37	1.46	1
286	3.24	4.68	6.76	6.92	6.33	5.65	4.96	4.28	3.65	2.97	2.41	1.46	1.23
287	3.24	4.58	6.69	6.92	6.34	5.65	4.96	4.29	3.69	3.09	2.44	2.04	1.63
288	3.24	4.61	6.71	6.92	6.34	5.65	4.96	4.28	3.65	3.05	2.37	1.9	0
289	3.24	4.74	6.86	6.99	6.63	6.2	5.77	5.34	4.96	4.59	4.24	3.89	3.53
290	3.24	4.74	6.86	6.99	6.63	6.2	5.77	5.34	4.96	4.58	4.21	3.84	3.47
291	3.24	4.74	6.86	6.99	6.63	6.2	5.77	5.34	4.96	4.56	4.18	3.81	3.42
292	3.24	4.69	6.85	6.99	6.63	6.2	5.77	5.34	4.95	4.56	4.18	3.81	3.45
293	3.24	4.71	6.85	6.99	6.63	6.2	5.77	5.35	4.97	4.6	4.23	3.85	3.53
294	3.24	4.73	6.86	6.99	6.63	6.2	5.77	5.34	4.97	4.59	4.21	3.84	3.45
295	3.24	4.76	6.86	6.99	6.62	6.19	5.76	5.34	4.96	4.59	4.22	3.87	3.54
296	3.24	4.73	6.86	6.99	6.63	6.2	5.77	5.34	4.97	4.59	4.22	3.83	3.48
297	3.24	4.74	6.86	6.99	6.63	6.2	5.77	5.35	4.98	4.61	4.24	3.83	3.48
298	3.24	3.76	4.8	6.3	6.85	6.42	5.99	5.56	5.19	4.82	4.44	4.07	3.66
299	3.24	3.71	4.76	6.26	6.85	6.43	6	5.57	5.19	4.82	4.45	4.06	3.69
300	3.24	3.64	4.68	6.18	6.86	6.43	6	5.57	5.19	4.81	4.44	4.05	3.67
301	3.24	3.7	4.74	6.24	6.86	6.43	6	5.57	5.19	4.82	4.43	4.06	3.66
302	3.24	3.71	4.74	6.26	6.85	6.43	6	5.57	5.19	4.79	4.4	4	3.56
303	3.24	3.76	4.8	6.3	6.85	6.42	6	5.57	5.19	4.82	4.41	4.02	3.7
304	3.24	3.63	4.67	6.17	6.86	6.44	6.01	5.58	5.2	4.84	4.49	4.12	3.73
305	3.24	3.69	4.73	6.23	6.86	6.43	6	5.57	5.2	4.83	4.47	4.07	3.72
306	3.24	3.65	4.69	6.2	6.86	6.43	6	5.57	5.2	4.82	4.46	4.1	3.76
307	3.24	4.25	6	7.07	6.95	6.77	6.59	6.42	6.26	6.1	5.95	5.79	5.64
308	3.24	4.23	5.97	7.07	6.95	6.77	6.59	6.42	6.26	6.1	5.95	5.79	5.64
309	3.24	4.18	5.93	7.07	6.95	6.77	6.6	6.42	6.26	6.1	5.95	5.79	5.63
310	3.24	4.33	6.06	7.07	6.95	6.77	6.59	6.41	6.26	6.1	5.94	5.79	5.63
311	3.24	4.24	5.99	7.07	6.95	6.77	6.59	6.41	6.26	6.1	5.94	5.79	5.63
312	3.24	4.26	6.01	7.07	6.95	6.77	6.59	6.42	6.26	6.1	5.95	5.79	5.64
313	3.24	4.23	5.98	7.07	6.95	6.77	6.59	6.42	6.26	6.1	5.95	5.79	5.64
314	3.24	4.22	5.97	7.07	6.95	6.77	6.6	6.42	6.26	6.1	5.95	5.79	5.64

315	3.24	4.3	6.03	7.07	6.95	6.77	6.59	6.41	6.26	6.1	5.94	5.79	5.64
316	3.24	4.5	6.32	7.27	7.12	6.72	6.29	5.86	5.49	5.12	4.73	4.34	3.93
317	3.24	4.52	6.34	7.27	7.12	6.72	6.29	5.86	5.49	5.12	4.76	4.41	4.03
318	3.24	4.48	6.3	7.27	7.12	6.72	6.3	5.87	5.49	5.12	4.74	4.39	4.01
319	3.24	4.5	6.32	7.27	7.12	6.72	6.3	5.87	5.49	5.12	4.74	4.35	3.92
320	3.24	4.46	6.28	7.27	7.12	6.72	6.29	5.87	5.5	5.12	4.76	4.38	4.02
321	3.24	4.45	6.28	7.27	7.12	6.72	6.3	5.87	5.5	5.11	4.74	4.38	4
322	3.24	4.44	6.26	7.27	7.12	6.72	6.3	5.87	5.5	5.13	4.74	4.38	4
323	3.24	4.5	6.31	7.27	7.12	6.72	6.29	5.87	5.49	5.12	4.74	4.36	3.95
324	3.24	4.48	6.3	7.27	7.12	6.72	6.3	5.87	5.49	5.11	4.73	4.36	4.02
325	3.24	4.33	6.05	7.02	6.68	6.26	5.82	5.4	5.01	4.64	4.27	3.88	3.49
326	3.24	4.23	5.96	7.02	6.69	6.26	5.84	5.41	5.03	4.66	4.26	3.86	3.5
327	3.24	4.23	5.95	7.02	6.69	6.26	5.83	5.4	5.03	4.66	4.28	3.9	3.5
328	3.24	4.26	5.98	7.02	6.69	6.26	5.83	5.4	5.03	4.65	4.26	3.89	3.57
329	3.24	4.25	5.97	7.02	6.69	6.26	5.84	5.4	5.03	4.65	4.27	3.88	3.49
330	3.24	4.27	5.99	7.02	6.69	6.26	5.83	5.4	5.02	4.63	4.26	3.89	3.55
331	3.24	4.22	5.95	7.02	6.69	6.26	5.84	5.4	5.03	4.66	4.28	3.87	3.47
332	3.24	4.24	5.97	7.02	6.69	6.26	5.83	5.41	5.03	4.65	4.28	3.92	3.54
333	3.24	4.22	5.93	7.02	6.69	6.26	5.84	5.41	5.04	4.66	4.28	3.9	3.53
334	3.24	3.98	5.61	6.86	6.49	6.06	5.63	5.2	4.82	4.44	4.07	3.7	3.34
335	3.24	3.94	5.55	6.86	6.49	6.06	5.64	5.21	4.84	4.45	4.08	3.68	3.26
336	3.24	3.97	5.6	6.86	6.49	6.06	5.63	5.2	4.83	4.47	4.1	3.71	3.34
337	3.24	3.93	5.55	6.86	6.49	6.07	5.64	5.21	4.83	4.46	4.06	3.68	3.29
338	3.24	3.92	5.55	6.86	6.49	6.06	5.64	5.21	4.84	4.45	4.06	3.67	3.32
339	3.24	3.9	5.54	6.86	6.49	6.07	5.64	5.21	4.84	4.46	4.07	3.71	3.33
340	3.24	4.02	5.63	6.85	6.49	6.06	5.63	5.2	4.83	4.45	4.08	3.72	3.37
341	3.24	3.94	5.58	6.86	6.49	6.06	5.64	5.21	4.83	4.45	4.08	3.69	3.28
342	3.24	3.93	5.56	6.86	6.49	6.07	5.64	5.21	4.83	4.45	4.08	3.71	3.33
343	3.24	4.26	5.95	6.97	6.42	5.74	5.06	4.36	3.76	3.14	2.45	1.32	0
344	3.24	4.26	5.97	6.97	6.42	5.73	5.04	4.36	3.76	3.18	2.69	2.17	0
345	3.24	4.21	5.91	6.98	6.43	5.74	5.06	4.38	3.78	3.13	2.59	1.95	1.62
346	3.24	4.21	5.91	6.98	6.43	5.74	5.04	4.34	3.74	3.14	2.58	1.79	0
347	3.24	4.22	5.93	6.97	6.42	5.73	5.04	4.37	3.78	3.2	2.59	2.05	1.04
348	3.24	4.25	5.97	6.97	6.42	5.73	5.04	4.35	3.71	3.04	2.47	1.7	0
349	3.24	4.25	5.95	6.97	6.42	5.74	5.05	4.36	3.74	3.08	2.4	1.48	0
350	3.24	4.22	5.93	6.97	6.42	5.74	5.05	4.36	3.73	3.16	2.56	1.52	1.34
351	3.24	4.25	5.95	6.97	6.42	5.73	5.03	4.33	3.72	3.09	2.18	1.52	0.9
352	3.24	4.57	6.69	7	6.64	6.21	5.78	5.35	4.97	4.59	4.22	3.83	3.45
353	3.24	4.61	6.72	7	6.64	6.21	5.78	5.35	4.97	4.59	4.22	3.85	3.47
354	3.24	4.58	6.69	7	6.64	6.21	5.78	5.35	4.98	4.61	4.23	3.87	3.49
355	3.24	4.66	6.75	7	6.64	6.21	5.78	5.35	4.98	4.61	4.26	3.93	3.57
356	3.24	4.61	6.72	7	6.64	6.21	5.78	5.35	4.98	4.59	4.21	3.82	3.5
357	3.24	4.63	6.74	7	6.64	6.21	5.78	5.35	4.98	4.6	4.26	3.88	3.47
358	3.24	4.61	6.72	7	6.64	6.21	5.78	5.35	4.98	4.61	4.23	3.86	3.49
359	3.24	4.64	6.74	7	6.64	6.21	5.78	5.36	4.99	4.62	4.23	3.86	3.48
360	3.24	4.63	6.73	7	6.64	6.21	5.78	5.35	4.97	4.59	4.21	3.85	3.44
361	3.24	4.23	5.94	7.02	6.69	6.26	5.83	5.41	5.02	4.64	4.25	3.88	3.5
362	3.24	4.23	5.97	7.02	6.69	6.26	5.83	5.41	5.04	4.67	4.29	3.93	3.56
363	3.24	4.27	5.98	7.02	6.69	6.26	5.83	5.4	5.02	4.66	4.26	3.88	3.5
364	3.24	4.22	5.94	7.02	6.69	6.27	5.84	5.41	5.04	4.68	4.31	3.9	3.56
365	3.24	4.29	6.01	7.02	6.68	6.25	5.82	5.4	5.02	4.64	4.26	3.86	3.46
366	3.24	4.28	6	7.02	6.69	6.26	5.83	5.4	5.03	4.65	4.28	3.89	3.53
367	3.24	4.25	5.98	7.02	6.69	6.26	5.83	5.4	5.03	4.65	4.29	3.92	3.58
368	3.24	4.25	5.97	7.02	6.69	6.26	5.83	5.4	5.03	4.65	4.28	3.91	3.53

369	3.24	4.31	6.03	7.02	6.69	6.26	5.83	5.4	5.03	4.66	4.28	3.9	3.51
