# Supplementary material for: Development of an Agent-Based Model (ABM) to Simulate the Immune System and Integration of a Regression Method to Estimate the Key ABM Parameters by Fitting the Experimental Data
Source: PLoS One. 2015 Nov 4;10(11):e0141295. doi: 10.1371/journal.pone.0141295 (PMC4633145; doi:10.1371/journal.pone.0141295)
Supplement: S8 Table — (PDF) [file pone.0141295.s009.pdf]

S8 Table.Output data sets of model 137x9

samples	output data value of ABM with 13 time points												
	1	2	3	4	5	6	7	8	9	10	11	12	13
1	3.24	4.27	5.99	7.02	6.69	6.26	5.83	5.4	5.03	4.67	4.3	3.91	3.54
2	3.24	4.28	5.99	7.02	6.69	6.26	5.83	5.41	5.03	4.65	4.28	3.92	3.53
3	3.24	4.26	5.98	7.02	6.69	6.26	5.83	5.4	5.03	4.65	4.3	3.93	3.58
4	3.24	4.25	5.97	7.02	6.69	6.26	5.83	5.4	5.02	4.64	4.23	3.85	3.51
5	3.24	4.27	6	7.02	6.69	6.26	5.83	5.4	5.02	4.65	4.29	3.92	3.58
6	3.24	4.31	6.02	7.02	6.69	6.26	5.83	5.4	5.02	4.65	4.28	3.94	3.55
7	3.24	4.26	5.98	7.02	6.69	6.26	5.83	5.41	5.03	4.66	4.28	3.91	3.55
8	3.24	4.27	5.98	7.02	6.69	6.26	5.83	5.41	5.03	4.64	4.26	3.88	3.48
9	3.24	4.29	6.03	7.02	6.68	6.26	5.83	5.4	5.03	4.64	4.25	3.9	3.49
10	3.24	3.69	4.71	6.23	6.86	6.43	6	5.57	5.19	4.82	4.45	4.09	3.67
11	3.24	3.62	4.68	6.19	6.86	6.43	6	5.57	5.2	4.83	4.45	4.07	3.69
12	3.24	3.68	4.7	6.21	6.86	6.43	6	5.57	5.2	4.82	4.45	4.05	3.69
13	3.24	3.7	4.75	6.25	6.85	6.43	6	5.57	5.19	4.81	4.43	4.05	3.63
14	3.24	3.71	4.77	6.27	6.85	6.43	6	5.57	5.2	4.83	4.44	4.07	3.72
15	3.24	3.7	4.74	6.25	6.86	6.43	6	5.58	5.2	4.82	4.44	4.09	3.72
16	3.24	3.71	4.76	6.27	6.85	6.43	6	5.57	5.2	4.83	4.46	4.06	3.67
17	3.24	3.63	4.67	6.19	6.86	6.43	6	5.57	5.19	4.82	4.45	4.04	3.64
18	3.24	3.66	4.71	6.22	6.86	6.43	6	5.57	5.19	4.83	4.44	4.06	3.69
19	3.24	4.26	6	7.07	6.95	6.77	6.59	6.42	6.26	6.11	5.95	5.8	5.64
20	3.24	4.27	6.02	7.07	6.95	6.77	6.59	6.42	6.26	6.1	5.95	5.8	5.64
21	3.24	4.22	5.97	7.07	6.95	6.77	6.59	6.42	6.26	6.11	5.95	5.8	5.64
22	3.24	4.26	6.01	7.07	6.95	6.77	6.59	6.41	6.26	6.1	5.95	5.79	5.63
23	3.24	4.36	6.1	7.07	6.95	6.77	6.59	6.42	6.26	6.11	5.95	5.79	5.64
24	3.24	4.24	5.98	7.07	6.95	6.77	6.6	6.42	6.26	6.11	5.95	5.79	5.64
25	3.24	4.25	6	7.07	6.95	6.77	6.59	6.42	6.26	6.1	5.95	5.79	5.63
26	3.24	4.21	5.94	7.07	6.95	6.77	6.59	6.42	6.26	6.1	5.95	5.79	5.64
27	3.24	4.25	5.99	7.07	6.95	6.77	6.59	6.41	6.26	6.1	5.95	5.79	5.63
28	3.24	4.51	6.33	7.27	7.12	6.72	6.29	5.86	5.48	5.11	4.75	4.38	3.99
29	3.24	4.46	6.28	7.27	7.12	6.72	6.3	5.87	5.49	5.13	4.76	4.39	3.99
30	3.24	4.48	6.3	7.27	7.12	6.72	6.3	5.87	5.49	5.12	4.76	4.39	4.04
31	3.24	4.5	6.32	7.27	7.12	6.72	6.3	5.87	5.49	5.12	4.75	4.39	4.03
32	3.24	4.48	6.3	7.27	7.12	6.72	6.29	5.86	5.49	5.11	4.74	4.34	3.98
33	3.24	4.5	6.31	7.27	7.12	6.72	6.3	5.87	5.49	5.11	4.74	4.37	3.99
34	3.24	4.48	6.3	7.27	7.12	6.72	6.29	5.86	5.49	5.12	4.75	4.36	4
35	3.24	4.46	6.28	7.27	7.12	6.72	6.3	5.87	5.49	5.11	4.74	4.36	3.97
36	3.24	4.51	6.33	7.27	7.12	6.72	6.29	5.86	5.49	5.12	4.74	4.38	4.02
37	3.24	4.24	5.97	7.02	6.69	6.26	5.84	5.41	5.04	4.68	4.29	3.9	3.5
38	3.24	4.27	5.99	7.02	6.69	6.26	5.83	5.4	5.03	4.64	4.26	3.9	3.51
39	3.24	4.28	6	7.02	6.69	6.26	5.83	5.4	5.02	4.64	4.27	3.89	3.5
40	3.24	4.28	6	7.02	6.69	6.26	5.83	5.41	5.03	4.66	4.28	3.91	3.52
41	3.24	4.22	5.95	7.02	6.69	6.26	5.83	5.4	5.03	4.65	4.26	3.91	3.51
42	3.24	4.27	5.99	7.02	6.69	6.26	5.83	5.41	5.03	4.65	4.27	3.9	3.49
43	3.24	4.26	5.99	7.02	6.69	6.26	5.83	5.4	5.03	4.66	4.28	3.91	3.53
44	3.24	4.21	5.94	7.02	6.69	6.26	5.84	5.41	5.03	4.65	4.29	3.9	3.5
45	3.24	4.29	6.01	7.02	6.69	6.26	5.83	5.41	5.03	4.66	4.28	3.89	3.51

46	3.24	3.95	5.55	6.86	6.49	6.06	5.64	5.21	4.83	4.46	4.07	3.7	3.32
47	3.24	3.97	5.59	6.86	6.49	6.06	5.63	5.2	4.83	4.45	4.1	3.72	3.31
48	3.24	3.94	5.56	6.86	6.49	6.07	5.64	5.21	4.84	4.46	4.1	3.73	3.36
49	3.24	3.91	5.53	6.86	6.49	6.07	5.64	5.21	4.84	4.46	4.08	3.72	3.34
50	3.24	3.97	5.59	6.86	6.49	6.06	5.63	5.2	4.83	4.43	4.08	3.73	3.33
51	3.24	3.93	5.55	6.86	6.49	6.06	5.63	5.21	4.82	4.44	4.05	3.66	3.29
52	3.24	4.02	5.65	6.85	6.49	6.06	5.63	5.2	4.83	4.45	4.07	3.66	3.28
53	3.24	4.02	5.65	6.85	6.49	6.06	5.63	5.2	4.82	4.44	4.06	3.66	3.27
54	3.24	3.98	5.61	6.86	6.49	6.06	5.64	5.21	4.84	4.47	4.09	3.7	3.31
55	3.24	4.21	5.91	6.98	6.43	5.74	5.05	4.37	3.76	3.08	2.41	1.54	0
56	3.24	4.21	5.92	6.98	6.43	5.74	5.04	4.33	3.72	3.14	2.57	2.09	1
57	3.24	4.3	6.01	6.97	6.42	5.73	5.04	4.34	3.72	3.14	2.42	1.72	1.73
58	3.24	4.22	5.92	6.98	6.43	5.74	5.04	4.34	3.74	3.15	2.51	1.94	1.38
59	3.24	4.27	5.97	6.97	6.42	5.73	5.05	4.35	3.76	3.11	2.4	1.97	1.71
60	3.24	4.24	5.94	6.97	6.43	5.74	5.04	4.35	3.78	3.15	2.34	1	0
61	3.24	4.26	5.95	6.97	6.42	5.73	5.04	4.35	3.77	3.24	2.73	2.16	0.9
62	3.24	4.18	5.89	6.98	6.43	5.73	5.04	4.35	3.73	3.09	2.44	1.81	0
63	3.24	4.21	5.91	6.98	6.43	5.74	5.04	4.35	3.73	3.08	2.5	0.95	0
64	3.24	4.64	6.74	7	6.64	6.21	5.78	5.36	4.98	4.61	4.23	3.85	3.51
65	3.24	4.62	6.73	7	6.64	6.21	5.78	5.35	4.97	4.59	4.21	3.86	3.51
66	3.24	4.62	6.73	7	6.64	6.21	5.78	5.35	4.97	4.59	4.21	3.84	3.5
67	3.24	4.65	6.75	7	6.64	6.21	5.78	5.35	4.99	4.61	4.23	3.86	3.51
68	3.24	4.62	6.72	7	6.64	6.21	5.78	5.35	4.97	4.6	4.24	3.86	3.48
69	3.24	4.66	6.76	7	6.64	6.21	5.78	5.36	4.99	4.62	4.25	3.87	3.49
70	3.24	4.6	6.72	7	6.64	6.21	5.78	5.36	4.98	4.59	4.2	3.81	3.44
71	3.24	4.65	6.75	7	6.64	6.21	5.78	5.35	4.98	4.59	4.22	3.88	3.51
72	3.24	4.61	6.72	7	6.64	6.21	5.78	5.35	4.98	4.61	4.23	3.85	3.44
73	3.24	3.36	4.07	5.08	6.91	6.67	6.24	5.81	5.44	5.06	4.69	4.3	3.93
74	3.24	3.37	4.05	5.06	6.91	6.67	6.24	5.81	5.44	5.06	4.68	4.32	3.93
75	3.24	3.37	4.05	5.05	6.91	6.67	6.24	5.82	5.44	5.08	4.7	4.32	3.95
76	3.24	3.44	4.12	5.14	6.93	6.66	6.23	5.8	5.43	5.06	4.69	4.32	3.96
77	3.24	3.39	4.08	5.08	6.91	6.67	6.24	5.81	5.44	5.05	4.69	4.32	3.95
78	3.24	3.45	4.16	5.15	6.93	6.66	6.23	5.8	5.42	5.04	4.66	4.28	3.91
79	3.24	3.39	4.09	5.08	6.91	6.67	6.24	5.81	5.43	5.05	4.68	4.3	3.94
80	3.24	3.39	4.09	5.08	6.92	6.67	6.24	5.81	5.44	5.06	4.7	4.33	3.93
81	3.24	3.34	4.02	5.02	6.9	6.68	6.25	5.82	5.44	5.07	4.7	4.33	3.97
82	3.24	3.67	4.75	6.29	7.02	6.84	6.66	6.48	6.33	6.17	6.02	5.87	5.71
83	3.24	3.67	4.75	6.3	7.02	6.84	6.66	6.48	6.33	6.17	6.02	5.86	5.71
84	3.24	3.78	4.83	6.36	7.01	6.83	6.66	6.48	6.32	6.16	6.01	5.85	5.7
85	3.24	3.7	4.77	6.31	7.02	6.84	6.66	6.48	6.33	6.17	6.01	5.86	5.7
86	3.24	3.68	4.74	6.28	7.02	6.84	6.66	6.48	6.33	6.17	6.02	5.87	5.71
87	3.24	3.71	4.8	6.34	7.01	6.84	6.66	6.48	6.32	6.17	6.01	5.86	5.7
88	3.24	3.74	4.81	6.35	7.01	6.84	6.66	6.48	6.32	6.17	6.01	5.85	5.7
89	3.24	3.66	4.74	6.29	7.02	6.84	6.66	6.48	6.33	6.17	6.01	5.86	5.7
90	3.24	3.68	4.77	6.32	7.02	6.84	6.66	6.48	6.32	6.17	6.01	5.86	5.7
91	3.24	3.99	5.17	6.8	7.22	6.85	6.42	6	5.62	5.24	4.85	4.47	4.1
92	3.24	3.93	5.1	6.75	7.22	6.85	6.43	6	5.63	5.25	4.87	4.5	4.1
93	3.24	3.92	5.09	6.74	7.22	6.85	6.43	6	5.63	5.25	4.85	4.49	4.13
94	3.24	3.94	5.11	6.76	7.22	6.85	6.43	6	5.62	5.25	4.87	4.5	4.12
95	3.24	3.92	5.08	6.73	7.22	6.85	6.43	6	5.62	5.24	4.85	4.46	4.11
96	3.24	3.94	5.12	6.76	7.22	6.85	6.43	6	5.62	5.25	4.88	4.5	4.13
97	3.24	3.93	5.1	6.75	7.22	6.85	6.43	6	5.63	5.26	4.89	4.51	4.15
98	3.24	4.03	5.21	6.82	7.22	6.84	6.42	5.99	5.62	5.24	4.86	4.49	4.12
99	3.24	3.94	5.12	6.76	7.22	6.85	6.43	6	5.63	5.25	4.87	4.48	4.14

100	3.24	3.67	4.7	6.22	6.86	6.43	6	5.57	5.2	4.83	4.46	4.1	3.72
101	3.24	3.67	4.7	6.2	6.86	6.43	6	5.58	5.2	4.81	4.42	4.06	3.71
102	3.24	3.7	4.75	6.25	6.85	6.43	6	5.57	5.19	4.81	4.43	4.06	3.67
103	3.24	3.69	4.71	6.22	6.86	6.43	6	5.57	5.2	4.82	4.44	4.06	3.7
104	3.24	3.7	4.72	6.22	6.86	6.43	6	5.57	5.2	4.83	4.45	4.06	3.68
105	3.24	3.7	4.74	6.25	6.85	6.43	6	5.57	5.2	4.82	4.45	4.1	3.7
106	3.24	3.75	4.8	6.29	6.85	6.42	5.99	5.56	5.19	4.8	4.43	4.04	3.69
107	3.24	3.65	4.69	6.19	6.86	6.44	6.01	5.58	5.21	4.83	4.46	4.07	3.71
108	3.24	3.68	4.72	6.22	6.86	6.43	6	5.57	5.2	4.81	4.44	4.06	3.69
109	3.24	3.36	4.27	5.61	6.72	6.29	5.86	5.43	5.05	4.67	4.31	3.93	3.55
110	3.24	3.35	4.28	5.61	6.72	6.29	5.86	5.43	5.04	4.68	4.31	3.93	3.55
111	3.24	3.3	4.22	5.58	6.72	6.29	5.86	5.44	5.07	4.7	4.33	3.97	3.59
112	3.24	3.47	4.38	5.7	6.7	6.28	5.85	5.42	5.04	4.67	4.31	3.95	3.58
113	3.24	3.37	4.27	5.62	6.71	6.29	5.86	5.43	5.06	4.68	4.31	3.95	3.58
114	3.24	3.41	4.35	5.68	6.71	6.28	5.85	5.43	5.05	4.67	4.29	3.93	3.55
115	3.24	3.46	4.39	5.73	6.7	6.27	5.84	5.41	5.03	4.67	4.32	3.93	3.57
116	3.24	3.4	4.31	5.66	6.71	6.28	5.86	5.43	5.05	4.67	4.31	3.95	3.55
117	3.24	3.5	4.44	5.78	6.69	6.27	5.84	5.41	5.04	4.66	4.29	3.91	3.52
118	3.24	3.65	4.66	6.14	6.71	6.03	5.33	4.64	4.05	3.44	2.89	2.35	1.7
119	3.24	3.68	4.68	6.14	6.71	6.03	5.34	4.65	4.05	3.5	2.97	2.35	1.6
120	3.24	3.69	4.72	6.18	6.7	6.02	5.33	4.64	4.05	3.46	2.89	2.23	1.62
121	3.24	3.72	4.74	6.2	6.7	6.01	5.33	4.62	4.03	3.46	2.72	2.07	1.52
122	3.24	3.58	4.58	6.06	6.73	6.04	5.35	4.67	4.08	3.47	2.92	2.3	1.49
123	3.24	3.74	4.75	6.21	6.7	6.01	5.33	4.64	4.04	3.36	2.73	2.18	1.73
124	3.24	3.71	4.73	6.19	6.7	6.01	5.33	4.64	4.04	3.46	2.83	2.33	1.97
125	3.24	3.73	4.75	6.22	6.7	6.01	5.32	4.65	4.06	3.49	2.91	2.3	1.98
126	3.24	3.69	4.72	6.18	6.7	6.02	5.33	4.65	4.07	3.44	2.83	2.19	1.28
127	3.24	4.26	6.02	7.09	7.04	6.94	6.85	6.75	6.67	6.59	6.51	6.42	6.34
128	3.24	4.25	6	7.09	7.04	6.94	6.85	6.76	6.67	6.59	6.51	6.42	6.34
129	3.24	4.22	5.98	7.09	7.04	6.94	6.85	6.75	6.67	6.59	6.51	6.42	6.34
130	3.24	4.24	5.99	7.09	7.04	6.94	6.85	6.75	6.67	6.59	6.51	6.42	6.34
131	3.24	4.33	6.08	7.09	7.04	6.94	6.85	6.75	6.67	6.59	6.5	6.42	6.34
132	3.24	4.27	6.01	7.09	7.04	6.94	6.85	6.75	6.67	6.59	6.51	6.42	6.34
133	3.24	4.28	6.03	7.09	7.04	6.94	6.85	6.75	6.67	6.59	6.51	6.42	6.34
134	3.24	4.24	5.98	7.09	7.04	6.94	6.85	6.75	6.67	6.59	6.51	6.42	6.34
135	3.24	4.3	6.04	7.09	7.04	6.94	6.85	6.75	6.67	6.59	6.51	6.42	6.34
136	3.24	4.44	6.28	7.31	7.34	7.18	7	6.82	6.67	6.51	6.36	6.2	6.04
137	3.24	4.44	6.28	7.31	7.34	7.18	7	6.82	6.67	6.51	6.36	6.2	6.05
138	3.24	4.5	6.33	7.32	7.34	7.17	7	6.82	6.66	6.51	6.35	6.2	6.04
139	3.24	4.47	6.31	7.31	7.34	7.18	7	6.82	6.66	6.51	6.35	6.2	6.04
140	3.24	4.52	6.34	7.32	7.34	7.18	7	6.82	6.67	6.51	6.35	6.2	6.04
141	3.24	4.47	6.31	7.31	7.34	7.18	7	6.82	6.66	6.51	6.35	6.2	6.04
142	3.24	4.54	6.36	7.32	7.34	7.17	7	6.82	6.66	6.51	6.35	6.2	6.04
143	3.24	4.5	6.33	7.32	7.34	7.17	7	6.82	6.67	6.51	6.35	6.2	6.04
144	3.24	4.51	6.34	7.32	7.34	7.17	7	6.82	6.67	6.51	6.35	6.2	6.04
145	3.24	4.17	5.91	7.07	6.95	6.77	6.6	6.42	6.26	6.1	5.95	5.79	5.64
146	3.24	4.27	6	7.07	6.95	6.77	6.59	6.41	6.26	6.1	5.95	5.79	5.64
147	3.24	4.24	5.97	7.07	6.95	6.77	6.59	6.41	6.26	6.1	5.94	5.79	5.63
148	3.24	4.26	6	7.07	6.95	6.77	6.59	6.42	6.26	6.1	5.95	5.79	5.63
149	3.24	4.3	6.04	7.07	6.95	6.77	6.59	6.41	6.26	6.1	5.94	5.79	5.63
150	3.24	4.29	6.02	7.07	6.95	6.77	6.59	6.42	6.26	6.11	5.95	5.79	5.64
151	3.24	4.26	5.99	7.07	6.95	6.77	6.59	6.41	6.26	6.1	5.95	5.79	5.63
152	3.24	4.28	6.01	7.07	6.95	6.77	6.59	6.42	6.26	6.1	5.95	5.79	5.63
153	3.24	4.29	6.02	7.07	6.95	6.77	6.59	6.41	6.26	6.1	5.95	5.8	5.64

154	3.24	3.98	5.61	6.9	6.75	6.58	6.4	6.22	6.06	5.91	5.75	5.6	5.44
155	3.24	3.93	5.59	6.9	6.75	6.57	6.39	6.22	6.06	5.9	5.75	5.59	5.44
156	3.24	4.02	5.67	6.9	6.75	6.57	6.39	6.22	6.06	5.91	5.75	5.6	5.44
157	3.24	3.95	5.59	6.9	6.75	6.57	6.4	6.22	6.06	5.91	5.75	5.6	5.44
158	3.24	3.98	5.63	6.9	6.75	6.57	6.4	6.22	6.06	5.91	5.75	5.59	5.44
159	3.24	3.95	5.58	6.9	6.75	6.57	6.4	6.22	6.06	5.91	5.75	5.6	5.44
160	3.24	4.05	5.7	6.9	6.75	6.57	6.39	6.21	6.06	5.9	5.75	5.59	5.43
161	3.24	4.02	5.65	6.9	6.75	6.57	6.4	6.22	6.06	5.91	5.75	5.6	5.44
162	3.24	3.97	5.61	6.9	6.75	6.57	6.4	6.22	6.06	5.91	5.75	5.59	5.43
163	3.24	4.52	6.37	7.37	7.41	7.12	6.76	6.36	6	5.63	5.27	4.9	4.53
164	3.24	4.5	6.36	7.37	7.41	7.12	6.75	6.36	6	5.63	5.27	4.89	4.52
165	3.24	4.59	6.44	7.37	7.4	7.12	6.75	6.35	5.99	5.62	5.25	4.87	4.49
166	3.24	4.54	6.39	7.37	7.4	7.12	6.75	6.35	5.99	5.63	5.26	4.89	4.51
167	3.24	4.52	6.38	7.37	7.41	7.12	6.75	6.35	5.99	5.62	5.25	4.89	4.52
168	3.24	4.53	6.38	7.37	7.41	7.12	6.75	6.36	6	5.63	5.25	4.89	4.52
169	3.24	4.55	6.4	7.37	7.4	7.12	6.75	6.35	5.99	5.62	5.25	4.87	4.49
170	3.24	4.54	6.39	7.37	7.4	7.12	6.75	6.35	5.99	5.63	5.26	4.89	4.51
171	3.24	4.54	6.39	7.37	7.4	7.12	6.75	6.35	5.99	5.63	5.26	4.89	4.53
172	3.24	4.47	6.28	7.27	7.12	6.72	6.3	5.87	5.49	5.12	4.73	4.35	3.94
173	3.24	4.5	6.32	7.27	7.12	6.72	6.29	5.87	5.49	5.11	4.74	4.38	4.02
174	3.24	4.49	6.31	7.27	7.12	6.72	6.29	5.87	5.49	5.12	4.75	4.36	3.96
175	3.24	4.48	6.3	7.27	7.12	6.72	6.3	5.87	5.49	5.12	4.76	4.38	4.01
176	3.24	4.51	6.32	7.27	7.12	6.72	6.29	5.86	5.49	5.11	4.71	4.33	3.95
177	3.24	4.47	6.29	7.27	7.12	6.72	6.29	5.86	5.48	5.11	4.72	4.36	3.98
178	3.24	4.44	6.26	7.27	7.12	6.72	6.3	5.87	5.5	5.12	4.74	4.35	3.97
179	3.24	4.45	6.27	7.27	7.12	6.72	6.29	5.87	5.5	5.13	4.76	4.39	4.01
180	3.24	4.49	6.31	7.27	7.12	6.72	6.3	5.87	5.49	5.12	4.75	4.38	4
181	3.24	4.23	5.96	7.02	6.69	6.26	5.83	5.4	5.03	4.65	4.28	3.92	3.52
182	3.24	4.28	6.01	7.02	6.69	6.26	5.83	5.4	5.03	4.67	4.29	3.91	3.52
183	3.24	4.27	5.99	7.02	6.69	6.26	5.83	5.41	5.03	4.67	4.29	3.93	3.54
184	3.24	4.24	5.95	7.02	6.69	6.26	5.83	5.41	5.04	4.66	4.28	3.9	3.51
185	3.24	4.18	5.91	7.02	6.69	6.26	5.83	5.4	5.03	4.65	4.27	3.9	3.54
186	3.24	4.24	5.96	7.02	6.69	6.26	5.83	5.41	5.03	4.65	4.27	3.91	3.53
187	3.24	4.26	5.98	7.02	6.69	6.26	5.83	5.39	5.01	4.63	4.27	3.87	3.48
188	3.24	4.28	6	7.02	6.69	6.26	5.83	5.4	5.02	4.65	4.29	3.92	3.51
189	3.24	4.21	5.94	7.02	6.69	6.26	5.83	5.41	5.03	4.64	4.26	3.87	3.51
190	3.24	3.94	5.56	6.86	6.49	6.06	5.64	5.21	4.83	4.45	4.08	3.69	3.34
191	3.24	4.02	5.65	6.85	6.49	6.06	5.63	5.21	4.83	4.45	4.07	3.72	3.41
192	3.24	3.94	5.58	6.86	6.49	6.07	5.64	5.21	4.82	4.45	4.08	3.74	3.37
193	3.24	3.91	5.54	6.86	6.49	6.07	5.64	5.21	4.85	4.46	4.07	3.72	3.35
194	3.24	3.95	5.57	6.86	6.49	6.06	5.64	5.21	4.83	4.46	4.07	3.7	3.34
195	3.24	3.92	5.54	6.86	6.49	6.07	5.64	5.21	4.84	4.46	4.08	3.72	3.32
196	3.24	3.93	5.55	6.86	6.49	6.07	5.64	5.21	4.85	4.46	4.08	3.71	3.34
197	3.24	4	5.62	6.86	6.49	6.06	5.63	5.21	4.84	4.45	4.08	3.72	3.39
198	3.24	4.02	5.64	6.85	6.49	6.06	5.63	5.2	4.83	4.45	4.07	3.71	3.36
199	3.24	3.92	5.52	6.81	6.44	6.01	5.59	5.16	4.78	4.41	4.04	3.67	3.28
200	3.24	3.87	5.45	6.81	6.45	6.02	5.59	5.16	4.78	4.4	4.03	3.61	3.23
201	3.24	3.8	5.39	6.82	6.45	6.02	5.59	5.17	4.79	4.42	4.06	3.71	3.37
202	3.24	3.87	5.44	6.81	6.45	6.02	5.59	5.17	4.8	4.4	4.01	3.65	3.28
203	3.24	3.9	5.48	6.81	6.44	6.02	5.58	5.15	4.78	4.4	4.02	3.6	3.25
204	3.24	3.93	5.51	6.81	6.44	6.01	5.58	5.15	4.78	4.4	4.04	3.65	3.28
205	3.24	3.81	5.4	6.82	6.45	6.02	5.59	5.17	4.79	4.42	4.05	3.66	3.21
206	3.24	3.91	5.5	6.81	6.44	6.02	5.59	5.17	4.8	4.42	4.05	3.65	3.24
207	3.24	3.88	5.46	6.81	6.45	6.02	5.59	5.17	4.8	4.43	4.06	3.7	3.3

208	3.24	4.52	6.32	7.22	6.91	6.27	5.6	4.91	4.31	3.7	3.09	2.46	1.93
209	3.24	4.49	6.29	7.22	6.91	6.28	5.6	4.92	4.33	3.73	3.1	2.6	1.87
210	3.24	4.48	6.29	7.22	6.91	6.28	5.6	4.93	4.3	3.66	3.05	2.41	1.61
211	3.24	4.45	6.26	7.22	6.91	6.28	5.6	4.92	4.29	3.69	3.11	2.45	1.92
212	3.24	4.47	6.27	7.22	6.91	6.28	5.6	4.92	4.33	3.73	3.11	2.61	2.22
213	3.24	4.5	6.3	7.22	6.91	6.27	5.59	4.9	4.3	3.69	3.01	2.41	1.87
214	3.24	4.45	6.25	7.22	6.91	6.28	5.6	4.91	4.3	3.61	2.93	2.28	1.82
215	3.24	4.47	6.27	7.22	6.91	6.28	5.6	4.9	4.3	3.66	2.9	2.03	0.7
216	3.24	4.48	6.29	7.22	6.91	6.28	5.6	4.92	4.33	3.74	3.15	2.57	1.9
217	3.24	4.23	5.94	6.97	6.42	5.74	5.05	4.37	3.75	3.17	2.53	1.75	1.72
218	3.24	4.23	5.94	6.97	6.42	5.73	5.04	4.34	3.71	3.2	2.55	2.01	1.58
219	3.24	4.28	5.99	6.97	6.42	5.73	5.05	4.35	3.72	3.12	2.51	2.01	1.38
220	3.24	4.2	5.91	6.97	6.43	5.73	5.04	4.35	3.71	3.03	2.47	1.89	0.95
221	3.24	4.17	5.87	6.98	6.43	5.74	5.06	4.38	3.73	3.05	2.45	1.59	0
222	3.24	4.21	5.91	6.98	6.43	5.74	5.04	4.34	3.73	3.14	2.63	2.23	0
223	3.24	4.2	5.89	6.98	6.43	5.74	5.05	4.37	3.75	3.2	2.55	2.02	1.54
224	3.24	4.24	5.96	6.97	6.42	5.73	5.04	4.35	3.71	3.06	2.3	2.02	1.51
225	3.24	4.16	5.88	6.98	6.43	5.74	5.06	4.36	3.75	3.05	2.48	1.7	0.78
226	3.24	4.06	5.65	6.81	6.22	5.53	4.84	4.15	3.5	2.96	2.34	0.7	0
227	3.24	3.91	5.51	6.82	6.23	5.54	4.85	4.18	3.58	2.87	2.28	1.65	0
228	3.24	3.96	5.56	6.82	6.22	5.53	4.84	4.14	3.57	2.91	2.32	1.9	1.68
229	3.24	3.89	5.49	6.82	6.23	5.54	4.85	4.16	3.51	2.89	2.11	1.2	0
230	3.24	3.97	5.57	6.81	6.22	5.54	4.85	4.17	3.58	2.97	2.25	1.51	1.18
231	3.24	3.9	5.51	6.82	6.23	5.54	4.85	4.18	3.59	3.01	2.33	1.52	0
232	3.24	3.95	5.55	6.82	6.23	5.54	4.85	4.16	3.57	2.96	2.27	1.48	1.45
233	3.24	3.92	5.53	6.82	6.23	5.54	4.85	4.15	3.55	2.85	2.16	1.78	0
234	3.24	3.93	5.52	6.82	6.23	5.54	4.85	4.17	3.55	2.93	2.34	1.87	0.3
235	3.24	4.29	5.99	6.95	6.33	5.55	4.77	4	3.21	2.44	2	1.18	0
236	3.24	4.27	5.97	6.96	6.33	5.55	4.77	3.96	3.19	2.54	1.59	0	0
237	3.24	4.28	5.97	6.96	6.33	5.54	4.76	3.97	3.21	2.59	1.6	0	0
238	3.24	4.3	6	6.95	6.32	5.54	4.77	3.98	3.3	2.58	0.85	0	0
239	3.24	4.25	5.94	6.96	6.33	5.55	4.78	3.98	3.33	2.65	2.29	0.3	0
240	3.24	4.22	5.91	6.96	6.34	5.56	4.79	3.99	3.25	2.72	2.09	1.38	0
241	3.24	4.21	5.92	6.96	6.33	5.56	4.79	4.01	3.31	2.73	1.88	1.2	0
242	3.24	4.19	5.89	6.96	6.34	5.56	4.77	4.01	3.36	2.6	1.89	0.6	0
243	3.24	4.25	5.95	6.96	6.33	5.55	4.78	3.96	3.3	2.62	2.06	0.48	0
244	3.24	4.63	6.75	7.07	6.93	6.75	6.57	6.39	6.24	6.08	5.93	5.77	5.62
245	3.24	4.67	6.78	7.07	6.93	6.75	6.57	6.39	6.24	6.08	5.93	5.77	5.62
246	3.24	4.56	6.7	7.07	6.93	6.75	6.57	6.4	6.24	6.08	5.93	5.77	5.62
247	3.24	4.65	6.76	7.07	6.93	6.75	6.57	6.39	6.24	6.08	5.93	5.77	5.62
248	3.24	4.56	6.7	7.07	6.93	6.75	6.58	6.4	6.24	6.08	5.93	5.77	5.62
249	3.24	4.65	6.77	7.07	6.93	6.75	6.57	6.39	6.24	6.08	5.92	5.77	5.61
250	3.24	4.65	6.76	7.07	6.93	6.75	6.57	6.39	6.24	6.08	5.92	5.77	5.61
251	3.24	4.63	6.75	7.07	6.93	6.75	6.57	6.39	6.24	6.08	5.93	5.77	5.61
252	3.24	4.58	6.71	7.07	6.93	6.75	6.57	6.4	6.24	6.08	5.93	5.77	5.62
253	3.24	4.83	6.92	7.29	7.09	6.68	6.25	5.82	5.45	5.06	4.67	4.3	3.89
254	3.24	4.84	6.92	7.29	7.08	6.68	6.25	5.83	5.45	5.09	4.72	4.36	4
255	3.24	4.86	6.93	7.29	7.08	6.68	6.25	5.83	5.46	5.09	4.71	4.34	3.99
256	3.24	4.82	6.91	7.29	7.09	6.68	6.25	5.82	5.45	5.08	4.7	4.33	3.94
257	3.24	4.84	6.92	7.29	7.09	6.68	6.25	5.82	5.45	5.08	4.7	4.33	3.92
258	3.24	4.89	6.93	7.29	7.08	6.68	6.25	5.82	5.45	5.08	4.7	4.33	3.93
259	3.24	4.81	6.91	7.29	7.09	6.68	6.25	5.83	5.45	5.08	4.71	4.36	3.96
260	3.24	4.81	6.91	7.29	7.09	6.68	6.25	5.82	5.45	5.09	4.72	4.35	4.03
261	3.24	4.88	6.93	7.29	7.08	6.68	6.25	5.83	5.45	5.08	4.7	4.34	3.95

262	3.24	4.65	6.75	7	6.64	6.21	5.78	5.36	4.98	4.6	4.22	3.85	3.45
263	3.24	4.6	6.71	7	6.64	6.21	5.78	5.36	4.99	4.62	4.27	3.9	3.56
264	3.24	4.54	6.67	7	6.64	6.21	5.78	5.36	4.98	4.61	4.26	3.86	3.45
265	3.24	4.62	6.72	7	6.64	6.21	5.78	5.35	4.98	4.6	4.25	3.87	3.46
266	3.24	4.65	6.75	7	6.64	6.21	5.78	5.34	4.97	4.58	4.19	3.77	3.38
267	3.24	4.64	6.75	7	6.64	6.21	5.78	5.35	4.98	4.6	4.24	3.85	3.53
268	3.24	4.62	6.73	7	6.64	6.21	5.78	5.34	4.96	4.58	4.2	3.82	3.43
269	3.24	4.61	6.72	7	6.64	6.21	5.78	5.35	4.97	4.6	4.21	3.85	3.49
270	3.24	4.58	6.7	7	6.64	6.21	5.79	5.36	4.99	4.61	4.25	3.89	3.52
271	3.24	4.3	6.37	6.81	6.43	6	5.57	5.15	4.77	4.4	4	3.61	3.24
272	3.24	4.35	6.42	6.8	6.43	6	5.57	5.14	4.76	4.39	4.03	3.66	3.24
273	3.24	4.31	6.37	6.81	6.43	6	5.57	5.15	4.78	4.41	4.04	3.65	3.29
274	3.24	4.4	6.46	6.8	6.43	6	5.57	5.13	4.76	4.38	4.02	3.61	3.17
275	3.24	4.34	6.41	6.8	6.43	6	5.57	5.14	4.77	4.4	4.02	3.65	3.3
276	3.24	4.31	6.38	6.81	6.43	6	5.58	5.15	4.78	4.41	4.04	3.64	3.29
277	3.24	4.29	6.37	6.81	6.43	6	5.58	5.15	4.77	4.38	4.02	3.62	3.2
278	3.24	4.37	6.43	6.8	6.43	6	5.57	5.13	4.76	4.39	4.01	3.64	3.24
279	3.24	4.38	6.44	6.8	6.43	6	5.57	5.14	4.77	4.4	4.02	3.64	3.28
280	3.24	4.63	6.72	6.92	6.34	5.65	4.96	4.27	3.68	3.04	2.41	1.48	0
281	3.24	4.63	6.72	6.92	6.34	5.65	4.97	4.27	3.64	2.96	2.01	1.38	1.28
282	3.24	4.63	6.72	6.92	6.34	5.65	4.97	4.28	3.67	3.1	2.35	1.86	1.65
283	3.24	4.57	6.68	6.93	6.34	5.66	4.97	4.3	3.66	3.03	2.37	1.6	1.28
284	3.24	4.61	6.71	6.92	6.34	5.65	4.96	4.28	3.7	3.11	2.47	1.69	0.78
285	3.24	4.6	6.7	6.92	6.34	5.65	4.96	4.29	3.69	3.12	2.55	2.04	1.18
286	3.24	4.59	6.69	6.92	6.35	5.66	4.96	4.29	3.66	3.08	2.4	1	0
287	3.24	4.68	6.75	6.92	6.34	5.65	4.97	4.29	3.73	3.18	2.48	1.72	1.41
288	3.24	4.58	6.68	6.92	6.34	5.66	4.96	4.28	3.67	3.04	2.47	1.79	0.6
289	3.24	4.72	6.86	6.99	6.63	6.2	5.77	5.34	4.97	4.59	4.21	3.81	3.46
290	3.24	4.77	6.87	6.99	6.62	6.19	5.77	5.34	4.97	4.59	4.21	3.86	3.54
291	3.24	4.74	6.86	6.99	6.63	6.19	5.77	5.34	4.97	4.6	4.22	3.83	3.47
292	3.24	4.76	6.87	6.99	6.62	6.2	5.77	5.34	4.97	4.59	4.23	3.87	3.48
293	3.24	4.73	6.86	6.99	6.63	6.2	5.77	5.34	4.96	4.6	4.24	3.89	3.5
294	3.24	4.71	6.86	6.99	6.63	6.2	5.77	5.34	4.97	4.6	4.21	3.79	3.32
295	3.24	4.8	6.87	6.99	6.62	6.19	5.77	5.34	4.96	4.59	4.22	3.84	3.47
296	3.24	4.7	6.85	6.99	6.63	6.2	5.77	5.34	4.95	4.59	4.22	3.86	3.49
297	3.24	4.74	6.86	6.99	6.63	6.2	5.77	5.34	4.97	4.59	4.22	3.84	3.47
298	3.24	4.3	6.02	7.02	6.68	6.26	5.83	5.4	5.02	4.65	4.28	3.88	3.52
299	3.24	4.23	5.95	7.02	6.69	6.26	5.83	5.4	5.03	4.66	4.3	3.95	3.6
300	3.24	4.22	5.94	7.02	6.69	6.26	5.83	5.41	5.04	4.67	4.31	3.95	3.53
301	3.24	4.18	5.9	7.02	6.69	6.27	5.84	5.41	5.03	4.65	4.28	3.91	3.53
302	3.24	4.21	5.93	7.02	6.69	6.26	5.84	5.41	5.04	4.65	4.28	3.91	3.54
303	3.24	4.25	5.97	7.02	6.69	6.26	5.83	5.41	5.03	4.65	4.27	3.88	3.5
304	3.24	4.24	5.97	7.02	6.69	6.26	5.83	5.4	5.03	4.65	4.27	3.93	3.53
305	3.24	4.31	6.02	7.02	6.69	6.26	5.83	5.4	5.02	4.65	4.28	3.91	3.52
306	3.24	4.21	5.93	7.02	6.69	6.26	5.83	5.39	5.02	4.65	4.27	3.92	3.56
307	3.24	3.17	3.69	4.4	5.95	6.89	6.56	6.14	5.76	5.39	5.01	4.63	4.27
308	3.24	3.15	3.67	4.35	5.91	6.89	6.57	6.15	5.78	5.4	5.03	4.65	4.28
309	3.24	3.26	3.81	4.51	6.03	6.89	6.54	6.12	5.74	5.36	4.99	4.62	4.24
310	3.24	3.18	3.76	4.47	5.99	6.89	6.55	6.13	5.75	5.38	5	4.62	4.25
311	3.24	3.2	3.72	4.39	5.92	6.89	6.57	6.15	5.77	5.4	5.03	4.66	4.32
312	3.24	3.25	3.77	4.46	6	6.89	6.55	6.13	5.75	5.38	5.01	4.64	4.26
313	3.24	3.18	3.67	4.38	5.93	6.89	6.57	6.14	5.77	5.4	5.03	4.65	4.29
314	3.24	3.16	3.62	4.3	5.84	6.89	6.59	6.17	5.79	5.42	5.05	4.68	4.3
315	3.24	3.17	3.72	4.42	5.97	6.89	6.56	6.13	5.76	5.38	5.01	4.63	4.25

316	3.24	3.43	4.16	5.2	7.02	6.93	6.75	6.57	6.42	6.26	6.11	5.95	5.79
317	3.24	3.48	4.21	5.25	7.03	6.92	6.74	6.57	6.41	6.25	6.1	5.94	5.79
318	3.24	3.49	4.24	5.31	7.03	6.92	6.74	6.56	6.41	6.25	6.1	5.94	5.78
319	3.24	3.46	4.19	5.21	7.02	6.93	6.75	6.57	6.41	6.26	6.1	5.94	5.79
320	3.24	3.39	4.11	5.17	7.01	6.93	6.75	6.57	6.42	6.26	6.1	5.94	5.79
321	3.24	3.46	4.2	5.23	7.02	6.92	6.75	6.57	6.41	6.26	6.1	5.95	5.79
322	3.24	3.43	4.18	5.22	7.02	6.93	6.75	6.57	6.42	6.26	6.11	5.95	5.79
323	3.24	3.44	4.17	5.21	7.02	6.93	6.75	6.57	6.41	6.26	6.1	5.95	5.79
324	3.24	3.35	4.07	5.14	7.01	6.93	6.75	6.57	6.42	6.26	6.1	5.95	5.79
325	3.24	3.65	4.48	5.69	7.28	7.01	6.59	6.17	5.79	5.42	5.05	4.68	4.32
326	3.24	3.66	4.48	5.7	7.28	7.01	6.59	6.17	5.79	5.42	5.05	4.68	4.31
327	3.24	3.73	4.55	5.76	7.28	7	6.58	6.16	5.78	5.41	5.03	4.66	4.29
328	3.24	3.66	4.49	5.7	7.28	7.01	6.59	6.17	5.8	5.42	5.05	4.68	4.32
329	3.24	3.64	4.46	5.67	7.28	7.01	6.6	6.17	5.8	5.42	5.06	4.7	4.34
330	3.24	3.61	4.44	5.66	7.28	7.01	6.6	6.17	5.8	5.41	5.04	4.66	4.28
331	3.24	3.7	4.53	5.73	7.28	7	6.59	6.16	5.79	5.42	5.05	4.68	4.3
332	3.24	3.7	4.51	5.71	7.28	7	6.59	6.17	5.79	5.42	5.06	4.7	4.34
333	3.24	3.64	4.48	5.69	7.28	7.01	6.59	6.17	5.79	5.41	5.04	4.67	4.3
334	3.24	3.48	4.17	5.18	6.93	6.65	6.23	5.8	5.42	5.04	4.66	4.28	3.92
335	3.24	3.31	4.02	5.02	6.9	6.68	6.25	5.82	5.45	5.07	4.69	4.31	3.94
336	3.24	3.38	4.11	5.1	6.92	6.66	6.24	5.81	5.43	5.06	4.69	4.3	3.95
337	3.24	3.42	4.12	5.11	6.92	6.66	6.23	5.8	5.43	5.06	4.69	4.29	3.91
338	3.24	3.36	4.03	5.02	6.9	6.68	6.25	5.82	5.44	5.07	4.69	4.31	3.93
339	3.24	3.36	4.07	5.04	6.9	6.68	6.25	5.82	5.44	5.06	4.67	4.29	3.95
340	3.24	3.43	4.1	5.1	6.92	6.67	6.24	5.81	5.44	5.05	4.67	4.3	3.9
341	3.24	3.37	4.09	5.09	6.92	6.67	6.24	5.81	5.44	5.07	4.7	4.31	3.94
342	3.24	3.38	4.05	5.05	6.91	6.67	6.24	5.82	5.44	5.07	4.68	4.3	3.91
343	3.24	3.15	3.73	4.57	6.33	6.6	6.18	5.76	5.38	5.01	4.63	4.26	3.88
344	3.24	3.13	3.77	4.58	6.34	6.6	6.18	5.75	5.38	5	4.63	4.27	3.89
345	3.24	3.22	3.75	4.57	6.31	6.6	6.19	5.76	5.39	5.01	4.65	4.28	3.9
346	3.24	3.13	3.73	4.54	6.29	6.61	6.2	5.77	5.39	5.02	4.64	4.25	3.88
347	3.24	3.11	3.67	4.52	6.28	6.61	6.2	5.77	5.39	5.03	4.65	4.28	3.92
348	3.24	3.09	3.67	4.48	6.25	6.62	6.21	5.78	5.41	5.03	4.67	4.3	3.94
349	3.24	3.11	3.73	4.54	6.29	6.61	6.19	5.77	5.39	5.02	4.65	4.28	3.93
350	3.24	3.08	3.7	4.52	6.28	6.61	6.2	5.76	5.39	5.01	4.65	4.28	3.92
351	3.24	3.17	3.71	4.51	6.27	6.61	6.2	5.77	5.39	5.02	4.65	4.28	3.91
352	3.24	3.44	4.11	5.07	6.83	6.43	5.74	5.05	4.46	3.87	3.26	2.68	2.07
353	3.24	3.35	4.01	4.95	6.79	6.46	5.77	5.08	4.47	3.86	3.26	2.55	2.11
354	3.24	3.43	4.11	5.08	6.83	6.42	5.74	5.04	4.44	3.85	3.31	2.67	2.1
355	3.24	3.45	4.13	5.06	6.83	6.43	5.74	5.06	4.47	3.85	3.2	2.7	2.31
356	3.24	3.33	4.01	4.97	6.79	6.46	5.77	5.08	4.48	3.89	3.34	2.73	2.1
357	3.24	3.41	4.07	5.01	6.81	6.44	5.75	5.07	4.47	3.86	3.28	2.77	2.29
358	3.24	3.36	4.04	5	6.81	6.44	5.76	5.08	4.47	3.81	3.27	2.72	1.89
359	3.24	3.37	4.04	5.01	6.81	6.44	5.75	5.06	4.46	3.86	3.35	2.86	2.3
360	3.24	3.42	4.06	5.02	6.81	6.44	5.76	5.07	4.47	3.88	3.29	2.66	2.06
361	3.24	3.77	4.85	6.4	7.07	6.98	6.88	6.79	6.71	6.62	6.54	6.46	6.37
362	3.24	3.69	4.76	6.32	7.07	6.98	6.88	6.79	6.71	6.62	6.54	6.46	6.38
363	3.24	3.69	4.78	6.34	7.07	6.98	6.88	6.79	6.71	6.62	6.54	6.46	6.37
364	3.24	3.67	4.73	6.28	7.07	6.98	6.88	6.79	6.71	6.63	6.54	6.46	6.38
365	3.24	3.7	4.76	6.32	7.07	6.98	6.88	6.79	6.71	6.62	6.54	6.46	6.38
366	3.24	3.65	4.74	6.3	7.07	6.98	6.88	6.79	6.71	6.62	6.54	6.46	6.38
367	3.24	3.68	4.75	6.31	7.07	6.98	6.88	6.79	6.71	6.62	6.54	6.46	6.38
368	3.24	3.68	4.75	6.3	7.07	6.98	6.88	6.79	6.71	6.62	6.54	6.46	6.38
369	3.24	3.7	4.78	6.33	7.07	6.98	6.88	6.79	6.71	6.62	6.54	6.46	6.38

370	3.24	3.92	5.11	6.79	7.37	7.23	7.05	6.88	6.72	6.57	6.41	6.25	6.09
371	3.24	3.96	5.15	6.82	7.37	7.23	7.05	6.87	6.72	6.56	6.41	6.25	6.09
372	3.24	3.95	5.15	6.81	7.37	7.23	7.05	6.88	6.72	6.56	6.4	6.25	6.09
373	3.24	3.97	5.16	6.83	7.37	7.23	7.05	6.87	6.72	6.56	6.4	6.25	6.09
374	3.24	3.95	5.16	6.82	7.37	7.23	7.05	6.87	6.72	6.56	6.41	6.25	6.1
375	3.24	3.99	5.18	6.83	7.37	7.23	7.05	6.87	6.71	6.56	6.41	6.25	6.09
376	3.24	3.96	5.17	6.83	7.37	7.23	7.05	6.87	6.72	6.56	6.4	6.25	6.09
377	3.24	3.98	5.17	6.83	7.37	7.23	7.05	6.87	6.72	6.56	6.4	6.25	6.09
378	3.24	4	5.19	6.84	7.37	7.23	7.05	6.87	6.72	6.56	6.41	6.25	6.09
379	3.24	3.79	4.86	6.38	7.01	6.83	6.66	6.48	6.32	6.17	6.01	5.86	5.7
380	3.24	3.68	4.75	6.29	7.02	6.84	6.66	6.48	6.33	6.17	6.02	5.86	5.7
381	3.24	3.7	4.79	6.33	7.01	6.84	6.66	6.48	6.33	6.17	6.02	5.86	5.7
382	3.24	3.73	4.79	6.32	7.01	6.84	6.66	6.48	6.33	6.17	6.01	5.86	5.7
383	3.24	3.74	4.8	6.33	7.01	6.84	6.66	6.48	6.32	6.17	6.01	5.86	5.7
384	3.24	3.77	4.84	6.37	7.01	6.84	6.66	6.48	6.32	6.17	6.01	5.86	5.7
385	3.24	3.68	4.74	6.28	7.02	6.84	6.66	6.48	6.33	6.17	6.02	5.86	5.71
386	3.24	3.78	4.84	6.37	7.01	6.84	6.66	6.48	6.32	6.16	6.01	5.85	5.7
387	3.24	3.68	4.75	6.29	7.02	6.84	6.66	6.48	6.33	6.17	6.01	5.86	5.7
388	3.24	3.41	4.38	5.75	6.84	6.66	6.48	6.3	6.15	5.99	5.83	5.67	5.52
389	3.24	3.42	4.38	5.76	6.84	6.66	6.48	6.3	6.15	5.99	5.83	5.68	5.52
390	3.24	3.39	4.34	5.72	6.84	6.66	6.48	6.3	6.15	5.99	5.83	5.68	5.52
391	3.24	3.32	4.26	5.65	6.84	6.66	6.49	6.31	6.15	6	5.84	5.69	5.53
392	3.24	3.47	4.41	5.79	6.83	6.66	6.48	6.3	6.14	5.99	5.83	5.68	5.52
393	3.24	3.39	4.33	5.72	6.84	6.66	6.48	6.3	6.15	5.99	5.84	5.68	5.53
394	3.24	3.4	4.34	5.73	6.84	6.66	6.48	6.3	6.15	5.99	5.84	5.68	5.53
395	3.24	3.46	4.4	5.78	6.84	6.66	6.48	6.3	6.14	5.99	5.83	5.68	5.52
396	3.24	3.28	4.26	5.64	6.84	6.66	6.48	6.31	6.15	5.99	5.84	5.68	5.53
397	3.24	4.06	5.29	6.95	7.45	7.21	6.86	6.47	6.11	5.75	5.37	5	4.63
398	3.24	3.99	5.21	6.9	7.45	7.21	6.86	6.47	6.11	5.75	5.38	5.01	4.63
399	3.24	3.98	5.2	6.89	7.45	7.22	6.86	6.47	6.11	5.75	5.37	5.01	4.64
400	3.24	4.05	5.26	6.93	7.45	7.21	6.86	6.47	6.11	5.75	5.39	5.02	4.64
401	3.24	4.07	5.28	6.95	7.45	7.21	6.86	6.47	6.11	5.75	5.38	5.03	4.66
402	3.24	4.01	5.23	6.91	7.45	7.21	6.86	6.47	6.11	5.74	5.38	5.01	4.64
403	3.24	4.02	5.24	6.92	7.45	7.21	6.86	6.47	6.11	5.75	5.38	5	4.63
404	3.24	4.09	5.31	6.96	7.45	7.21	6.86	6.47	6.11	5.74	5.37	4.99	4.61
405	3.24	4.08	5.31	6.96	7.45	7.21	6.86	6.46	6.11	5.74	5.36	4.99	4.61
406	3.24	3.91	5.09	6.75	7.22	6.85	6.43	6	5.62	5.25	4.88	4.52	4.14
407	3.24	3.92	5.11	6.75	7.22	6.85	6.43	6.01	5.63	5.25	4.86	4.48	4.15
408	3.24	4	5.19	6.82	7.22	6.84	6.42	5.99	5.62	5.24	4.87	4.51	4.14
409	3.24	3.94	5.1	6.75	7.22	6.85	6.43	6	5.63	5.26	4.9	4.53	4.15
410	3.24	3.94	5.13	6.77	7.22	6.85	6.43	6	5.63	5.25	4.88	4.51	4.18
411	3.24	3.99	5.17	6.8	7.22	6.84	6.42	5.99	5.62	5.24	4.87	4.51	4.15
412	3.24	3.95	5.13	6.77	7.22	6.85	6.43	5.99	5.62	5.25	4.88	4.51	4.13
413	3.24	3.92	5.1	6.75	7.22	6.85	6.43	6	5.63	5.25	4.87	4.49	4.12
414	3.24	3.91	5.09	6.74	7.22	6.85	6.43	6	5.63	5.26	4.89	4.51	4.17
415	3.24	3.66	4.69	6.19	6.86	6.43	6	5.57	5.2	4.82	4.45	4.06	3.71
416	3.24	3.66	4.71	6.2	6.86	6.43	6	5.58	5.21	4.84	4.47	4.12	3.73
417	3.24	3.64	4.67	6.18	6.86	6.44	6.01	5.58	5.2	4.83	4.46	4.07	3.73
418	3.24	3.72	4.77	6.27	6.85	6.42	5.99	5.56	5.19	4.81	4.43	4.05	3.69
419	3.24	3.65	4.68	6.19	6.86	6.44	6.01	5.58	5.19	4.81	4.43	4.05	3.69
420	3.24	3.73	4.78	6.27	6.85	6.42	5.99	5.56	5.19	4.81	4.45	4.09	3.71
421	3.24	3.59	4.64	6.16	6.86	6.44	6.01	5.58	5.22	4.84	4.45	4.07	3.72
422	3.24	3.65	4.72	6.22	6.86	6.43	6.01	5.58	5.2	4.83	4.44	4.06	3.68
423	3.24	3.62	4.66	6.17	6.86	6.44	6.01	5.58	5.21	4.82	4.46	4.08	3.72

424	3.24	3.46	4.38	5.72	6.7	6.27	5.84	5.41	5.03	4.65	4.28	3.92	3.55
425	3.24	3.44	4.4	5.73	6.7	6.27	5.84	5.41	5.03	4.66	4.3	3.95	3.59
426	3.24	3.4	4.31	5.66	6.71	6.28	5.85	5.43	5.05	4.68	4.29	3.91	3.51
427	3.24	3.52	4.44	5.77	6.69	6.27	5.84	5.4	5.03	4.64	4.27	3.9	3.52
428	3.24	3.33	4.25	5.59	6.72	6.29	5.86	5.43	5.06	4.67	4.3	3.92	3.56
429	3.24	3.39	4.33	5.66	6.71	6.28	5.85	5.43	5.06	4.68	4.29	3.92	3.57
430	3.24	3.38	4.31	5.64	6.71	6.28	5.85	5.42	5.05	4.69	4.31	3.93	3.55
431	3.24	3.13	4.05	5.4	6.74	6.31	5.89	5.46	5.08	4.72	4.34	3.98	3.63
432	3.24	3.41	4.34	5.66	6.71	6.28	5.85	5.42	5.05	4.67	4.3	3.94	3.58
433	3.24	3.32	4.19	5.48	6.68	6.25	5.82	5.39	5.02	4.64	4.28	3.87	3.51
434	3.24	3.27	4.19	5.48	6.68	6.25	5.82	5.4	5.02	4.65	4.27	3.9	3.53
435	3.24	3.19	4.09	5.36	6.69	6.27	5.84	5.41	5.03	4.65	4.27	3.9	3.53
436	3.24	3.28	4.1	5.38	6.69	6.27	5.84	5.41	5.03	4.66	4.29	3.92	3.52
437	3.24	3.23	4.11	5.39	6.69	6.26	5.83	5.4	5.02	4.64	4.28	3.92	3.53
438	3.24	3.19	4.08	5.37	6.69	6.27	5.84	5.41	5.04	4.66	4.29	3.91	3.55
439	3.24	3.18	4.09	5.38	6.69	6.27	5.84	5.41	5.04	4.66	4.28	3.88	3.51
440	3.24	3.39	4.28	5.56	6.67	6.24	5.81	5.38	5.01	4.63	4.27	3.87	3.55
441	3.24	3.21	4.08	5.37	6.69	6.26	5.83	5.41	5.03	4.65	4.27	3.89	3.53
442	3.24	3.92	5.07	6.69	7.08	6.49	5.83	5.15	4.56	3.94	3.29	2.72	2.2
443	3.24	3.89	5.04	6.67	7.09	6.5	5.83	5.14	4.57	4	3.38	2.73	1.82
444	3.24	3.94	5.09	6.71	7.08	6.49	5.82	5.14	4.55	3.98	3.39	2.87	2.36
445	3.24	3.87	5.03	6.66	7.09	6.5	5.83	5.13	4.52	3.91	3.31	2.65	2
446	3.24	3.96	5.11	6.72	7.08	6.49	5.82	5.14	4.55	4.01	3.46	2.91	2.14
447	3.24	3.85	5.02	6.65	7.09	6.5	5.82	5.14	4.51	3.87	3.3	2.4	1.3
448	3.24	3.88	5.04	6.67	7.09	6.5	5.83	5.14	4.53	3.92	3.36	2.7	1.58
449	3.24	3.9	5.03	6.66	7.09	6.5	5.83	5.14	4.55	3.94	3.38	2.84	1.77
450	3.24	3.95	5.09	6.7	7.08	6.49	5.83	5.15	4.54	3.93	3.25	2.56	2.21
451	3.24	3.6	4.62	6.09	6.72	6.03	5.34	4.66	4.05	3.48	2.84	2.26	1.89
452	3.24	3.72	4.75	6.22	6.7	6.01	5.32	4.63	4.03	3.44	2.87	2.26	1.48
453	3.24	3.65	4.65	6.12	6.71	6.03	5.34	4.65	4.05	3.4	2.74	2.05	1.6
454	3.24	3.68	4.69	6.15	6.71	6.02	5.34	4.63	4.04	3.48	2.96	2.22	1.38
455	3.24	3.65	4.66	6.13	6.71	6.02	5.34	4.63	4.05	3.48	2.88	2.35	0
456	3.24	3.71	4.71	6.18	6.7	6.02	5.33	4.64	4.04	3.48	2.9	2.41	1.57
457	3.24	3.67	4.67	6.15	6.71	6.02	5.33	4.62	4.01	3.39	2.82	2.26	1.34
458	3.24	3.7	4.7	6.16	6.71	6.02	5.33	4.65	4.04	3.37	2.77	2.13	1.48
459	3.24	3.71	4.69	6.16	6.71	6.02	5.34	4.64	4.04	3.38	2.87	2.15	1
460	3.24	3.4	4.3	5.58	6.6	5.91	5.22	4.54	3.96	3.37	2.77	2.32	1.38
461	3.24	3.36	4.25	5.51	6.61	5.93	5.24	4.57	3.96	3.4	2.82	2.24	1.93
462	3.24	3.4	4.27	5.57	6.6	5.91	5.22	4.53	3.92	3.32	2.77	2.26	1.67
463	3.24	3.41	4.29	5.58	6.6	5.91	5.22	4.54	3.93	3.36	2.65	2.09	1.08
464	3.24	3.39	4.26	5.55	6.6	5.92	5.23	4.55	3.97	3.33	2.79	1.96	1.49
465	3.24	3.46	4.35	5.64	6.59	5.9	5.21	4.53	3.94	3.35	2.77	2.26	1.7
466	3.24	3.37	4.26	5.55	6.6	5.91	5.23	4.56	3.95	3.38	2.79	2.07	1.18
467	3.24	3.33	4.2	5.51	6.61	5.93	5.24	4.53	3.94	3.31	2.61	2.04	1.18
468	3.24	3.33	4.25	5.54	6.61	5.92	5.23	4.54	3.93	3.29	2.69	2.15	1.79
469	3.24	3.62	4.64	6.1	6.67	5.89	5.11	4.34	3.67	2.99	2.24	1.64	1.43
470	3.24	3.77	4.79	6.23	6.64	5.86	5.09	4.3	3.62	2.95	2.21	1.43	0.85
471	3.24	3.68	4.69	6.15	6.66	5.88	5.11	4.31	3.64	2.99	2.41	1.93	1.61
472	3.24	3.71	4.72	6.17	6.65	5.87	5.09	4.31	3.63	2.92	2.09	0	0
473	3.24	3.61	4.61	6.07	6.67	5.9	5.12	4.34	3.64	3	2.2	1.3	0
474	3.24	3.69	4.71	6.17	6.65	5.87	5.1	4.32	3.65	2.94	1.93	0	0
475	3.24	3.67	4.67	6.13	6.66	5.88	5.1	4.3	3.6	2.91	2.32	1.43	0
476	3.24	3.75	4.78	6.23	6.64	5.86	5.09	4.31	3.63	2.83	1.73	0	0
477	3.24	3.64	4.68	6.14	6.66	5.88	5.09	4.29	3.66	2.94	2.26	1.7	0

478	3.24	3.92	5.29	6.95	6.76	6.33	5.9	5.48	5.1	4.71	4.33	3.92	3.54
479	3.24	3.95	5.3	6.95	6.76	6.33	5.9	5.47	5.09	4.73	4.37	3.98	3.59
480	3.24	3.94	5.3	6.95	6.76	6.33	5.9	5.47	5.09	4.7	4.31	3.96	3.59
481	3.24	3.96	5.32	6.96	6.76	6.33	5.9	5.47	5.1	4.73	4.37	4	3.61
482	3.24	3.96	5.32	6.96	6.75	6.33	5.9	5.48	5.1	4.72	4.35	3.96	3.6
483	3.24	4	5.36	6.96	6.75	6.33	5.9	5.47	5.1	4.72	4.34	3.99	3.61
484	3.24	3.97	5.35	6.96	6.75	6.32	5.89	5.46	5.09	4.71	4.33	3.95	3.59
485	3.24	3.89	5.23	6.93	6.76	6.33	5.91	5.47	5.1	4.72	4.36	3.98	3.64
486	3.24	4.01	5.38	6.97	6.75	6.32	5.89	5.46	5.08	4.7	4.33	3.94	3.56
487	3.24	4.2	5.95	7.1	7.08	7.02	6.96	6.9	6.85	6.8	6.75	6.7	6.65
488	3.24	4.3	6.05	7.1	7.07	7.02	6.96	6.9	6.85	6.8	6.75	6.7	6.65
489	3.24	4.29	6.03	7.1	7.08	7.02	6.96	6.9	6.85	6.8	6.75	6.7	6.65
490	3.24	4.22	5.97	7.1	7.08	7.02	6.96	6.9	6.85	6.8	6.75	6.7	6.65
491	3.24	4.26	6.01	7.1	7.08	7.02	6.96	6.9	6.85	6.8	6.75	6.7	6.65
492	3.24	4.23	6	7.1	7.08	7.02	6.96	6.9	6.85	6.8	6.75	6.7	6.65
493	3.24	4.26	6.01	7.1	7.08	7.02	6.96	6.9	6.85	6.8	6.75	6.7	6.65
494	3.24	4.29	6.03	7.1	7.08	7.02	6.96	6.9	6.85	6.8	6.75	6.7	6.65
495	3.24	4.23	5.98	7.1	7.08	7.02	6.96	6.9	6.85	6.8	6.75	6.7	6.65
496	3.24	4.49	6.33	7.33	7.42	7.33	7.24	7.14	7.06	6.98	6.9	6.81	6.73
497	3.24	4.53	6.36	7.33	7.42	7.33	7.24	7.14	7.06	6.98	6.9	6.81	6.73
498	3.24	4.5	6.33	7.33	7.42	7.33	7.24	7.14	7.06	6.98	6.9	6.81	6.73
499	3.24	4.52	6.37	7.33	7.42	7.33	7.24	7.14	7.06	6.98	6.89	6.81	6.73
500	3.24	4.49	6.33	7.33	7.42	7.33	7.24	7.14	7.06	6.98	6.89	6.81	6.73
501	3.24	4.49	6.33	7.33	7.42	7.33	7.24	7.14	7.06	6.98	6.9	6.81	6.73
502	3.24	4.49	6.33	7.33	7.42	7.33	7.24	7.14	7.06	6.98	6.9	6.81	6.73
503	3.24	4.5	6.34	7.33	7.42	7.33	7.24	7.14	7.06	6.98	6.9	6.81	6.73
504	3.24	4.48	6.32	7.33	7.42	7.33	7.24	7.14	7.06	6.98	6.9	6.81	6.73
505	3.24	4.24	5.99	7.09	7.04	6.94	6.85	6.75	6.67	6.59	6.51	6.42	6.34
506	3.24	4.21	5.96	7.09	7.04	6.94	6.85	6.75	6.67	6.59	6.51	6.42	6.34
507	3.24	4.24	5.98	7.09	7.04	6.94	6.85	6.75	6.67	6.59	6.51	6.42	6.34
508	3.24	4.25	6	7.09	7.04	6.94	6.85	6.76	6.67	6.59	6.51	6.43	6.34
509	3.24	4.27	6.01	7.09	7.04	6.94	6.85	6.75	6.67	6.59	6.51	6.42	6.34
510	3.24	4.24	5.98	7.09	7.04	6.94	6.85	6.75	6.67	6.59	6.51	6.43	6.34
511	3.24	4.27	6.01	7.09	7.04	6.94	6.85	6.76	6.67	6.59	6.51	6.42	6.34
512	3.24	4.33	6.08	7.09	7.04	6.94	6.85	6.75	6.67	6.59	6.51	6.42	6.34
513	3.24	4.29	6.04	7.09	7.04	6.94	6.85	6.75	6.67	6.59	6.51	6.42	6.34
514	3.24	4.02	5.67	6.92	6.84	6.75	6.65	6.56	6.48	6.39	6.31	6.23	6.14
515	3.24	4.03	5.67	6.92	6.84	6.74	6.65	6.56	6.47	6.39	6.31	6.23	6.14
516	3.24	3.94	5.6	6.92	6.84	6.75	6.65	6.56	6.47	6.39	6.31	6.22	6.14
517	3.24	3.96	5.62	6.92	6.84	6.75	6.65	6.56	6.47	6.39	6.31	6.23	6.14
518	3.24	3.97	5.62	6.92	6.84	6.74	6.65	6.56	6.47	6.39	6.31	6.22	6.14
519	3.24	3.97	5.64	6.92	6.84	6.74	6.65	6.56	6.47	6.39	6.31	6.22	6.14
520	3.24	3.99	5.62	6.92	6.84	6.75	6.65	6.56	6.47	6.39	6.31	6.23	6.14
521	3.24	3.98	5.64	6.92	6.84	6.75	6.65	6.56	6.47	6.39	6.31	6.23	6.14
522	3.24	3.99	5.63	6.92	6.84	6.75	6.65	6.56	6.47	6.39	6.31	6.23	6.14
523	3.24	4.59	6.45	7.42	7.58	7.48	7.33	7.16	7	6.85	6.69	6.54	6.38
524	3.24	4.56	6.42	7.42	7.58	7.48	7.33	7.16	7	6.84	6.69	6.53	6.37
525	3.24	4.55	6.41	7.41	7.58	7.49	7.33	7.16	7	6.85	6.69	6.54	6.39
526	3.24	4.53	6.4	7.41	7.58	7.49	7.33	7.16	7	6.85	6.69	6.54	6.38
527	3.24	4.54	6.41	7.41	7.58	7.49	7.33	7.16	7	6.85	6.69	6.54	6.38
528	3.24	4.58	6.44	7.42	7.58	7.48	7.33	7.16	7	6.85	6.69	6.54	6.38
529	3.24	4.53	6.4	7.41	7.58	7.48	7.33	7.16	7	6.85	6.69	6.54	6.38
530	3.24	4.58	6.44	7.42	7.58	7.48	7.33	7.16	7	6.85	6.69	6.54	6.38
531	3.24	4.56	6.43	7.42	7.58	7.48	7.33	7.16	7	6.85	6.69	6.54	6.38

532	3.24	4.51	6.35	7.32	7.34	7.18	7	6.82	6.66	6.51	6.35	6.2	6.04
533	3.24	4.47	6.3	7.31	7.34	7.18	7	6.82	6.67	6.51	6.35	6.2	6.04
534	3.24	4.56	6.39	7.32	7.34	7.17	7	6.82	6.66	6.51	6.35	6.19	6.04
535	3.24	4.47	6.3	7.31	7.34	7.18	7	6.82	6.67	6.51	6.36	6.2	6.04
536	3.24	4.52	6.35	7.32	7.34	7.18	7	6.82	6.67	6.51	6.36	6.2	6.05
537	3.24	4.48	6.31	7.31	7.34	7.18	7	6.82	6.66	6.51	6.35	6.19	6.04
538	3.24	4.52	6.35	7.32	7.34	7.17	7	6.82	6.66	6.51	6.35	6.2	6.04
539	3.24	4.51	6.34	7.32	7.34	7.18	7	6.82	6.67	6.51	6.36	6.2	6.05
540	3.24	4.46	6.29	7.31	7.34	7.18	7	6.82	6.67	6.51	6.35	6.2	6.04
541	3.24	4.22	5.96	7.07	6.95	6.77	6.59	6.42	6.26	6.11	5.95	5.8	5.64
542	3.24	4.26	6.01	7.07	6.95	6.77	6.59	6.41	6.26	6.1	5.95	5.79	5.63
543	3.24	4.28	6.01	7.07	6.95	6.77	6.59	6.41	6.26	6.1	5.95	5.79	5.64
544	3.24	4.28	6.01	7.07	6.95	6.77	6.59	6.42	6.26	6.11	5.95	5.79	5.64
545	3.24	4.22	5.95	7.07	6.95	6.77	6.59	6.42	6.26	6.1	5.95	5.79	5.64
546	3.24	4.25	5.99	7.07	6.95	6.77	6.59	6.42	6.26	6.11	5.95	5.79	5.63
547	3.24	4.32	6.05	7.07	6.95	6.77	6.59	6.42	6.26	6.1	5.94	5.79	5.63
548	3.24	4.24	5.98	7.07	6.95	6.77	6.59	6.42	6.26	6.1	5.95	5.79	5.64
549	3.24	4.27	6.01	7.07	6.95	6.77	6.59	6.41	6.26	6.1	5.95	5.79	5.63
550	3.24	3.94	5.58	6.9	6.75	6.58	6.4	6.22	6.06	5.91	5.75	5.6	5.44
551	3.24	3.99	5.64	6.9	6.75	6.57	6.4	6.22	6.06	5.91	5.75	5.6	5.44
552	3.24	4.04	5.68	6.9	6.75	6.57	6.39	6.22	6.06	5.91	5.75	5.6	5.44
553	3.24	3.93	5.57	6.9	6.75	6.58	6.4	6.22	6.06	5.91	5.75	5.6	5.44
554	3.24	4.05	5.69	6.9	6.75	6.57	6.39	6.22	6.06	5.91	5.75	5.6	5.44
555	3.24	3.93	5.58	6.9	6.75	6.57	6.4	6.22	6.06	5.9	5.75	5.6	5.44
556	3.24	4.02	5.65	6.9	6.75	6.57	6.4	6.22	6.06	5.91	5.75	5.6	5.44
557	3.24	4.01	5.64	6.9	6.75	6.57	6.4	6.22	6.06	5.91	5.75	5.6	5.44
558	3.24	3.94	5.59	6.9	6.75	6.58	6.4	6.22	6.07	5.91	5.76	5.6	5.45
559	3.24	3.88	5.48	6.85	6.7	6.52	6.35	6.17	6.01	5.86	5.7	5.55	5.39
560	3.24	3.91	5.51	6.85	6.7	6.52	6.35	6.17	6.01	5.86	5.7	5.55	5.39
561	3.24	3.84	5.44	6.85	6.7	6.53	6.35	6.17	6.01	5.86	5.7	5.54	5.38
562	3.24	3.98	5.59	6.85	6.7	6.52	6.34	6.16	6.01	5.86	5.7	5.54	5.39
563	3.24	3.79	5.4	6.85	6.71	6.53	6.35	6.17	6.01	5.86	5.71	5.55	5.39
564	3.24	3.87	5.49	6.85	6.7	6.52	6.35	6.17	6.01	5.86	5.7	5.55	5.4
565	3.24	3.88	5.49	6.85	6.7	6.52	6.34	6.17	6.01	5.86	5.7	5.54	5.39
566	3.24	3.77	5.39	6.85	6.71	6.53	6.35	6.17	6.02	5.86	5.71	5.55	5.39
567	3.24	3.88	5.49	6.85	6.7	6.52	6.35	6.17	6.01	5.86	5.7	5.55	5.39
568	3.24	4.32	6.05	7.05	6.84	6.56	6.28	6	5.76	5.52	5.27	5.03	4.79
569	3.24	4.25	5.99	7.05	6.84	6.56	6.28	6	5.75	5.51	5.26	5.03	4.78
570	3.24	4.31	6.04	7.05	6.84	6.56	6.28	6	5.76	5.51	5.27	5.04	4.79
571	3.24	4.24	5.97	7.05	6.84	6.56	6.28	6	5.76	5.51	5.27	5.02	4.77
572	3.24	4.28	6.01	7.05	6.84	6.56	6.28	6.01	5.76	5.52	5.27	5.03	4.78
573	3.24	4.21	5.94	7.05	6.84	6.56	6.28	6	5.76	5.51	5.27	5.03	4.78
574	3.24	4.24	5.99	7.05	6.84	6.56	6.28	6	5.76	5.52	5.27	5.02	4.78
575	3.24	4.28	6.02	7.05	6.84	6.56	6.28	6	5.75	5.51	5.27	5.02	4.78
576	3.24	4.27	6.01	7.05	6.84	6.56	6.28	6	5.76	5.52	5.28	5.03	4.79
577	3.24	4.59	6.46	7.42	7.56	7.39	7.12	6.81	6.52	6.21	5.9	5.58	5.25
578	3.24	4.59	6.45	7.42	7.56	7.39	7.12	6.81	6.52	6.22	5.91	5.58	5.26
579	3.24	4.59	6.46	7.42	7.56	7.39	7.12	6.81	6.52	6.22	5.91	5.59	5.26
580	3.24	4.55	6.42	7.42	7.56	7.39	7.12	6.81	6.52	6.22	5.91	5.59	5.26
581	3.24	4.54	6.41	7.41	7.56	7.39	7.12	6.81	6.52	6.21	5.9	5.58	5.26
582	3.24	4.53	6.4	7.41	7.56	7.39	7.12	6.82	6.53	6.22	5.91	5.59	5.27
583	3.24	4.55	6.42	7.41	7.56	7.39	7.12	6.81	6.52	6.22	5.91	5.59	5.26
584	3.24	4.54	6.41	7.41	7.56	7.39	7.12	6.81	6.52	6.22	5.9	5.59	5.26
585	3.24	4.6	6.46	7.42	7.56	7.39	7.12	6.81	6.52	6.22	5.91	5.59	5.27

586	3.24	4.59	6.43	7.37	7.4	7.12	6.75	6.35	5.99	5.63	5.26	4.89	4.52
587	3.24	4.56	6.41	7.37	7.4	7.12	6.75	6.35	5.99	5.61	5.24	4.87	4.49
588	3.24	4.52	6.38	7.37	7.4	7.12	6.75	6.36	6	5.63	5.25	4.88	4.51
589	3.24	4.56	6.41	7.37	7.4	7.12	6.75	6.36	5.99	5.63	5.26	4.9	4.52
590	3.24	4.56	6.41	7.37	7.4	7.12	6.75	6.36	5.99	5.63	5.27	4.91	4.52
591	3.24	4.53	6.38	7.37	7.4	7.12	6.75	6.35	5.99	5.62	5.25	4.88	4.51
592	3.24	4.5	6.36	7.37	7.4	7.12	6.75	6.35	5.99	5.62	5.25	4.88	4.51
593	3.24	4.53	6.39	7.37	7.41	7.12	6.75	6.35	5.99	5.62	5.24	4.86	4.48
594	3.24	4.58	6.43	7.37	7.4	7.12	6.75	6.35	5.98	5.62	5.25	4.87	4.51
595	3.24	4.43	6.25	7.26	7.12	6.72	6.3	5.87	5.5	5.13	4.75	4.39	4.02
596	3.24	4.45	6.27	7.27	7.12	6.72	6.3	5.87	5.5	5.13	4.75	4.36	3.99
597	3.24	4.49	6.31	7.27	7.12	6.72	6.29	5.86	5.49	5.1	4.73	4.35	3.97
598	3.24	4.49	6.3	7.27	7.12	6.72	6.29	5.87	5.49	5.12	4.75	4.34	3.91
599	3.24	4.47	6.29	7.27	7.12	6.72	6.3	5.87	5.5	5.11	4.73	4.35	3.99
600	3.24	4.53	6.34	7.27	7.12	6.72	6.3	5.87	5.5	5.12	4.74	4.36	3.97
601	3.24	4.48	6.29	7.27	7.12	6.72	6.3	5.87	5.5	5.13	4.76	4.38	4.02
602	3.24	4.51	6.33	7.27	7.12	6.72	6.3	5.87	5.49	5.12	4.74	4.34	3.96
603	3.24	4.46	6.29	7.27	7.12	6.72	6.3	5.87	5.5	5.12	4.75	4.39	4
604	3.24	4.42	6.19	7.16	6.89	6.46	6.03	5.6	5.23	4.85	4.48	4.1	3.68
605	3.24	4.38	6.15	7.16	6.89	6.47	6.04	5.61	5.24	4.87	4.5	4.1	3.7
606	3.24	4.38	6.16	7.16	6.89	6.47	6.04	5.61	5.24	4.86	4.48	4.07	3.68
607	3.24	4.34	6.13	7.15	6.89	6.47	6.04	5.62	5.24	4.86	4.49	4.1	3.72
608	3.24	4.38	6.16	7.16	6.89	6.47	6.04	5.61	5.23	4.86	4.49	4.11	3.74
609	3.24	4.43	6.21	7.16	6.89	6.46	6.03	5.6	5.23	4.86	4.5	4.15	3.81
610	3.24	4.34	6.12	7.15	6.9	6.47	6.04	5.61	5.24	4.86	4.49	4.11	3.67
611	3.24	4.41	6.18	7.16	6.89	6.47	6.04	5.61	5.24	4.86	4.49	4.15	3.77
612	3.24	4.38	6.16	7.16	6.89	6.47	6.04	5.61	5.24	4.88	4.51	4.13	3.72
613	3.24	4.26	5.98	7.02	6.69	6.26	5.83	5.41	5.04	4.68	4.29	3.93	3.51
614	3.24	4.29	6	7.02	6.69	6.26	5.83	5.4	5.03	4.66	4.3	3.92	3.52
615	3.24	4.31	6.02	7.02	6.69	6.26	5.83	5.4	5.01	4.65	4.26	3.86	3.51
616	3.24	4.25	5.97	7.02	6.69	6.26	5.83	5.41	5.03	4.65	4.26	3.9	3.57
617	3.24	4.27	5.99	7.02	6.69	6.26	5.83	5.4	5.02	4.64	4.27	3.9	3.52
618	3.24	4.22	5.95	7.02	6.69	6.26	5.83	5.4	5.03	4.66	4.26	3.89	3.51
619	3.24	4.26	5.98	7.02	6.69	6.26	5.83	5.41	5.02	4.64	4.26	3.88	3.53
620	3.24	4.28	6	7.02	6.69	6.26	5.83	5.4	5.03	4.67	4.3	3.93	3.56
621	3.24	4.25	5.97	7.02	6.69	6.26	5.83	5.4	5.03	4.65	4.28	3.9	3.51
622	3.24	4.08	5.74	6.92	6.56	6.13	5.7	5.26	4.88	4.51	4.15	3.76	3.42
623	3.24	4.03	5.71	6.92	6.56	6.13	5.71	5.28	4.9	4.53	4.17	3.77	3.34
624	3.24	4.07	5.74	6.92	6.56	6.13	5.71	5.28	4.9	4.53	4.16	3.75	3.44
625	3.24	4.07	5.73	6.92	6.56	6.13	5.7	5.27	4.9	4.53	4.14	3.77	3.42
626	3.24	4.06	5.71	6.92	6.56	6.13	5.7	5.28	4.9	4.53	4.15	3.78	3.39
627	3.24	4.17	5.83	6.91	6.55	6.13	5.7	5.28	4.9	4.51	4.15	3.78	3.4
628	3.24	4.14	5.8	6.91	6.55	6.12	5.69	5.27	4.89	4.52	4.15	3.8	3.41
629	3.24	4.04	5.71	6.92	6.56	6.13	5.7	5.27	4.91	4.52	4.14	3.77	3.45
630	3.24	4.11	5.77	6.92	6.56	6.13	5.7	5.28	4.9	4.52	4.14	3.75	3.41
631	3.24	3.95	5.59	6.86	6.49	6.06	5.63	5.2	4.83	4.46	4.08	3.66	3.3
632	3.24	3.96	5.56	6.86	6.49	6.07	5.64	5.22	4.84	4.47	4.1	3.69	3.3
633	3.24	3.97	5.6	6.86	6.49	6.06	5.63	5.21	4.82	4.44	4.08	3.72	3.32
634	3.24	3.98	5.61	6.86	6.49	6.06	5.63	5.2	4.82	4.44	4.07	3.7	3.37
635	3.24	3.98	5.6	6.86	6.49	6.06	5.63	5.2	4.82	4.45	4.07	3.72	3.3
636	3.24	3.9	5.53	6.86	6.49	6.06	5.64	5.21	4.83	4.45	4.07	3.67	3.25
637	3.24	3.99	5.63	6.86	6.49	6.06	5.64	5.2	4.82	4.45	4.06	3.69	3.28
638	3.24	3.91	5.53	6.86	6.49	6.06	5.63	5.2	4.83	4.44	4.08	3.7	3.35
639	3.24	3.96	5.58	6.86	6.49	6.06	5.64	5.21	4.84	4.48	4.11	3.73	3.38

640	3.24	3.97	5.54	6.81	6.44	6.01	5.58	5.15	4.77	4.4	4.04	3.64	3.24
641	3.24	3.94	5.52	6.81	6.44	6.01	5.58	5.15	4.78	4.4	4.03	3.66	3.25
642	3.24	3.87	5.44	6.81	6.45	6.02	5.59	5.16	4.79	4.42	4.04	3.68	3.32
643	3.24	3.87	5.44	6.81	6.45	6.02	5.59	5.16	4.79	4.42	4.04	3.63	3.24
644	3.24	3.79	5.38	6.82	6.45	6.02	5.59	5.17	4.79	4.42	4.05	3.65	3.29
645	3.24	3.7	5.3	6.82	6.46	6.03	5.6	5.17	4.8	4.42	4.06	3.68	3.27
646	3.24	3.77	5.38	6.82	6.45	6.02	5.59	5.17	4.8	4.41	4.05	3.63	3.22
647	3.24	3.81	5.39	6.82	6.45	6.02	5.59	5.16	4.79	4.41	4.07	3.68	3.34
648	3.24	3.89	5.47	6.81	6.44	6.02	5.59	5.16	4.79	4.41	4.02	3.66	3.27
649	3.24	3.77	5.34	6.8	6.43	6	5.57	5.14	4.77	4.39	4	3.6	3.26
650	3.24	3.67	5.25	6.8	6.44	6.01	5.58	5.16	4.78	4.4	4.05	3.67	3.28
651	3.24	3.71	5.29	6.8	6.44	6	5.58	5.16	4.78	4.4	3.99	3.61	3.26
652	3.24	3.78	5.36	6.8	6.43	6	5.57	5.14	4.76	4.39	4.02	3.65	3.25
653	3.24	3.78	5.34	6.8	6.43	6	5.57	5.14	4.77	4.4	4.02	3.65	3.22
654	3.24	3.85	5.43	6.79	6.43	6	5.56	5.14	4.76	4.39	4.03	3.68	3.31
655	3.24	3.8	5.38	6.8	6.43	6	5.57	5.14	4.77	4.4	4.03	3.66	3.26
656	3.24	3.78	5.36	6.8	6.43	6	5.57	5.15	4.77	4.39	4.01	3.64	3.26
657	3.24	3.79	5.37	6.8	6.43	6	5.58	5.14	4.77	4.39	4.01	3.63	3.26
658	3.24	4.25	5.95	6.99	6.53	5.95	5.37	4.8	4.28	3.79	3.26	2.77	2.26
659	3.24	4.22	5.92	6.99	6.54	5.96	5.38	4.8	4.28	3.77	3.29	2.94	2.34
660	3.24	4.16	5.87	7	6.54	5.96	5.38	4.78	4.26	3.67	3.14	2.66	2.11
661	3.24	4.2	5.91	7	6.54	5.96	5.38	4.79	4.28	3.77	3.2	2.61	2.31
662	3.24	4.27	5.98	6.99	6.53	5.95	5.37	4.79	4.27	3.78	3.28	2.72	2.18
663	3.24	4.22	5.92	7	6.54	5.96	5.37	4.8	4.3	3.79	3.27	2.75	2.28
664	3.24	4.27	5.98	6.99	6.53	5.95	5.36	4.79	4.27	3.76	3.17	2.61	2.01
665	3.24	4.23	5.94	6.99	6.53	5.95	5.37	4.79	4.26	3.76	3.22	2.71	2.26
666	3.24	4.24	5.95	6.99	6.53	5.95	5.37	4.79	4.29	3.77	3.29	2.65	1.84
667	3.24	4.54	6.38	7.32	7.24	6.8	6.27	5.69	5.16	4.62	4.06	3.49	2.84
668	3.24	4.57	6.4	7.33	7.24	6.8	6.27	5.69	5.17	4.63	4.1	3.58	2.99
669	3.24	4.52	6.36	7.32	7.24	6.8	6.27	5.69	5.17	4.63	4.1	3.53	2.93
670	3.24	4.52	6.36	7.32	7.24	6.8	6.27	5.69	5.16	4.62	4.07	3.51	2.89
671	3.24	4.52	6.36	7.32	7.24	6.8	6.27	5.69	5.17	4.64	4.09	3.52	2.92
672	3.24	4.55	6.38	7.32	7.24	6.8	6.27	5.69	5.17	4.64	4.09	3.53	3.02
673	3.24	4.53	6.37	7.32	7.24	6.8	6.27	5.69	5.16	4.62	4.08	3.54	2.97
674	3.24	4.54	6.38	7.32	7.24	6.8	6.27	5.69	5.17	4.63	4.06	3.46	2.81
675	3.24	4.53	6.36	7.32	7.24	6.8	6.27	5.7	5.18	4.63	4.07	3.52	2.95
676	3.24	4.5	6.29	7.22	6.91	6.27	5.59	4.9	4.31	3.72	3.13	2.6	2.21
677	3.24	4.47	6.27	7.22	6.91	6.28	5.6	4.92	4.34	3.74	3.12	2.47	1.67
678	3.24	4.52	6.32	7.22	6.91	6.28	5.6	4.93	4.33	3.74	3.16	2.52	1.89
679	3.24	4.46	6.26	7.22	6.91	6.28	5.61	4.92	4.33	3.74	3.13	2.56	2.17
680	3.24	4.47	6.27	7.22	6.91	6.28	5.61	4.92	4.31	3.66	3.07	2.56	1.74
681	3.24	4.46	6.25	7.22	6.91	6.28	5.61	4.94	4.35	3.71	3.04	2.29	1.91
682	3.24	4.47	6.28	7.22	6.91	6.28	5.6	4.91	4.3	3.71	3.02	2.39	1.95
683	3.24	4.43	6.23	7.22	6.92	6.28	5.6	4.91	4.31	3.73	3.19	2.55	1.49
684	3.24	4.4	6.21	7.22	6.92	6.28	5.6	4.93	4.3	3.67	3.14	2.65	2.16
685	3.24	4.25	5.94	6.97	6.42	5.74	5.04	4.37	3.77	3.15	2.5	1.66	0
686	3.24	4.18	5.88	6.98	6.43	5.74	5.05	4.37	3.78	3.14	2.49	1.86	1.28
687	3.24	4.28	5.98	6.97	6.42	5.74	5.05	4.37	3.76	3.19	2.59	2	0.95
688	3.24	4.18	5.9	6.98	6.43	5.74	5.05	4.36	3.78	3.19	2.56	1.72	0.78
689	3.24	4.25	5.96	6.97	6.42	5.73	5.04	4.35	3.71	3.02	2.49	1.94	1.34
690	3.24	4.19	5.91	6.98	6.43	5.73	5.05	4.37	3.75	3.14	2.6	2.18	1.48
691	3.24	4.26	5.96	6.97	6.42	5.74	5.05	4.35	3.78	3.18	2.55	1.8	0
692	3.24	4.27	5.97	6.97	6.42	5.73	5.04	4.34	3.73	3.22	2.56	1.94	1.43
693	3.24	4.26	5.96	6.97	6.42	5.74	5.05	4.35	3.73	3.14	2.55	1.98	0.7

694	3.24	3.95	5.55	6.82	6.23	5.54	4.85	4.15	3.52	2.93	2.16	1.46	0
695	3.24	3.94	5.55	6.82	6.23	5.53	4.86	4.19	3.6	2.95	2.28	1.49	0.3
696	3.24	3.98	5.59	6.81	6.22	5.53	4.84	4.17	3.58	2.96	2.35	1.49	1.15
697	3.24	3.96	5.56	6.82	6.22	5.54	4.85	4.16	3.52	2.9	2.21	0.3	0
698	3.24	3.94	5.54	6.82	6.23	5.54	4.85	4.17	3.51	2.92	2.08	1.86	1.08
699	3.24	3.99	5.59	6.81	6.22	5.53	4.85	4.17	3.57	3.02	2.51	2.01	0
700	3.24	3.98	5.58	6.81	6.22	5.53	4.85	4.16	3.57	2.94	2.46	1.98	1.2
701	3.24	3.96	5.56	6.82	6.23	5.54	4.85	4.17	3.56	2.87	2.15	1.45	1.11
702	3.24	3.94	5.54	6.82	6.23	5.54	4.85	4.15	3.56	2.92	2.42	2.04	1.74
703	3.24	3.87	5.45	6.77	6.18	5.49	4.8	4.11	3.57	3.02	2.54	1.72	1.34
704	3.24	3.86	5.42	6.78	6.18	5.49	4.8	4.12	3.51	2.85	2.39	1.84	1.23
705	3.24	3.73	5.29	6.79	6.2	5.51	4.82	4.13	3.53	2.91	2.31	1.75	0
706	3.24	3.73	5.3	6.79	6.2	5.51	4.82	4.12	3.51	2.97	2.29	1.48	0
707	3.24	3.79	5.35	6.78	6.19	5.5	4.81	4.12	3.52	2.92	2.36	1.85	1.48
708	3.24	3.83	5.4	6.78	6.19	5.49	4.8	4.09	3.48	2.81	2.29	1.15	0
709	3.24	3.82	5.41	6.78	6.19	5.5	4.81	4.13	3.53	2.96	2.17	1.18	0
710	3.24	3.93	5.47	6.77	6.18	5.49	4.79	4.13	3.53	2.97	2.34	1.99	0
711	3.24	3.82	5.39	6.78	6.19	5.5	4.81	4.12	3.52	2.86	2.2	1.86	1.11
712	3.24	4.45	6.26	7.2	6.84	6.13	5.37	4.61	3.94	3.28	2.68	1.97	1.52
713	3.24	4.5	6.29	7.2	6.84	6.13	5.36	4.59	3.88	3.19	2.53	1.91	1.32
714	3.24	4.47	6.28	7.2	6.84	6.13	5.37	4.61	3.93	3.24	2.59	1.99	0.3
715	3.24	4.47	6.26	7.2	6.84	6.14	5.38	4.6	3.91	3.2	2.33	1.36	0.3
716	3.24	4.51	6.3	7.2	6.84	6.13	5.36	4.57	3.86	3.08	2.44	1.72	0.48
717	3.24	4.49	6.29	7.2	6.84	6.13	5.37	4.56	3.85	3.13	2.4	1.81	1.76
718	3.24	4.45	6.25	7.2	6.85	6.13	5.37	4.58	3.89	3.11	2.15	0.78	0
719	3.24	4.44	6.24	7.2	6.85	6.14	5.38	4.6	3.91	3.16	2.33	1.4	0
720	3.24	4.42	6.22	7.2	6.85	6.14	5.37	4.6	3.91	3.28	2.63	1.72	0.7
721	3.24	4.26	5.96	6.96	6.33	5.55	4.77	4	3.34	2.58	1.28	0	0
722	3.24	4.21	5.91	6.96	6.33	5.55	4.79	4	3.3	2.43	1.6	0.3	0
723	3.24	4.21	5.9	6.96	6.34	5.56	4.78	4.01	3.34	2.6	1.75	1.54	0
724	3.24	4.2	5.89	6.96	6.33	5.56	4.78	3.99	3.32	2.69	2.18	1.7	0.3
725	3.24	4.25	5.95	6.96	6.33	5.55	4.78	3.97	3.29	2.42	1.88	1.4	0
726	3.24	4.23	5.91	6.96	6.33	5.55	4.77	3.98	3.35	2.54	2.01	1.62	1.11
727	3.24	4.17	5.86	6.96	6.34	5.56	4.78	4.04	3.35	2.55	2	0.85	0
728	3.24	4.28	5.98	6.96	6.33	5.55	4.79	4.03	3.4	2.73	2.22	1.59	1.34
729	3.24	4.24	5.93	6.96	6.33	5.56	4.78	4	3.3	2.62	1.99	1.28	0
730	3.24	3.93	5.53	6.8	6.13	5.35	4.57	3.78	3.13	2.42	1.95	1.26	0
731	3.24	3.98	5.55	6.8	6.13	5.36	4.57	3.77	3.18	2.33	1.76	1.08	0
732	3.24	3.99	5.57	6.8	6.13	5.34	4.56	3.77	3.06	2.44	1.81	1.15	0
733	3.24	3.97	5.56	6.8	6.13	5.35	4.57	3.76	3.08	2.34	1.15	1.26	0
734	3.24	3.98	5.58	6.8	6.13	5.35	4.58	3.85	3.17	2.54	1.99	1.52	0
735	3.24	3.89	5.48	6.81	6.14	5.36	4.57	3.77	3.09	2.39	1.56	0	0
736	3.24	3.93	5.53	6.8	6.14	5.35	4.58	3.79	3.07	2.29	1.49	1.18	0
737	3.24	3.95	5.53	6.8	6.13	5.34	4.57	3.82	3.2	2.68	2.16	1.52	0.6
738	3.24	3.93	5.52	6.8	6.14	5.36	4.58	3.8	3.08	2.41	1.3	1.2	0
739	3.24	4.3	5.99	6.95	6.29	5.47	4.65	3.83	3.1	2.17	0.3	0	0
740	3.24	4.27	5.96	6.95	6.29	5.47	4.66	3.84	3.15	2.46	1.84	0	0
741	3.24	4.31	5.99	6.95	6.29	5.47	4.65	3.85	3.13	2.32	1.38	1.36	0.9
742	3.24	4.3	5.98	6.95	6.29	5.47	4.66	3.81	3.13	2.4	1.45	1.32	0
743	3.24	4.23	5.93	6.95	6.29	5.47	4.65	3.84	3.12	2.44	1.65	1.38	0
744	3.24	4.18	5.89	6.95	6.29	5.48	4.65	3.83	3.05	2.11	1.4	0	0
745	3.24	4.21	5.9	6.95	6.29	5.47	4.63	3.84	3.13	2.41	0	0	0
746	3.24	4.18	5.87	6.95	6.3	5.48	4.67	3.85	3.1	2.31	1.84	0	0
747	3.24	4.26	5.95	6.95	6.29	5.47	4.65	3.84	3.21	2.54	1.82	0.6	0

748	3.24	4.52	6.5	7.01	6.65	6.22	5.79	5.36	4.98	4.61	4.22	3.83	3.49
749	3.24	4.44	6.42	7.01	6.66	6.23	5.8	5.36	5	4.62	4.26	3.89	3.59
750	3.24	4.46	6.44	7.01	6.65	6.23	5.8	5.38	5	4.63	4.27	3.9	3.51
751	3.24	4.51	6.48	7.01	6.65	6.22	5.79	5.36	4.99	4.62	4.24	3.87	3.49
752	3.24	4.46	6.45	7.01	6.65	6.23	5.8	5.37	4.99	4.63	4.25	3.86	3.5
753	3.24	4.51	6.48	7.01	6.65	6.22	5.79	5.36	4.99	4.62	4.25	3.84	3.49
754	3.24	4.45	6.44	7.01	6.65	6.23	5.8	5.37	5	4.62	4.22	3.83	3.46
755	3.24	4.51	6.49	7.01	6.65	6.22	5.79	5.37	4.99	4.63	4.24	3.9	3.51
756	3.24	4.41	6.4	7.01	6.66	6.23	5.8	5.36	4.99	4.62	4.25	3.89	3.56
757	3.24	4.63	6.75	7.1	7.03	6.93	6.84	6.74	6.66	6.58	6.49	6.41	6.33
758	3.24	4.63	6.75	7.1	7.03	6.93	6.84	6.74	6.66	6.58	6.49	6.41	6.33
759	3.24	4.65	6.77	7.1	7.03	6.93	6.84	6.74	6.66	6.58	6.5	6.41	6.33
760	3.24	4.66	6.77	7.1	7.03	6.93	6.84	6.74	6.66	6.58	6.49	6.41	6.33
761	3.24	4.65	6.77	7.1	7.03	6.93	6.84	6.74	6.66	6.58	6.49	6.41	6.33
762	3.24	4.66	6.78	7.1	7.03	6.93	6.84	6.74	6.66	6.58	6.5	6.41	6.33
763	3.24	4.63	6.76	7.1	7.03	6.93	6.84	6.74	6.66	6.58	6.5	6.41	6.33
764	3.24	4.65	6.77	7.1	7.03	6.93	6.84	6.74	6.66	6.58	6.49	6.41	6.33
765	3.24	4.61	6.74	7.1	7.03	6.93	6.84	6.74	6.66	6.58	6.49	6.41	6.33
766	3.24	4.84	6.93	7.35	7.33	7.16	6.98	6.8	6.65	6.49	6.34	6.18	6.03
767	3.24	4.85	6.93	7.35	7.33	7.16	6.98	6.8	6.65	6.49	6.33	6.18	6.02
768	3.24	4.88	6.94	7.35	7.33	7.16	6.98	6.8	6.65	6.49	6.33	6.18	6.02
769	3.24	4.88	6.94	7.35	7.33	7.16	6.98	6.8	6.65	6.49	6.34	6.18	6.02
770	3.24	4.85	6.93	7.35	7.33	7.16	6.98	6.8	6.65	6.49	6.33	6.18	6.02
771	3.24	4.85	6.93	7.35	7.33	7.16	6.98	6.8	6.65	6.49	6.34	6.18	6.03
772	3.24	4.82	6.92	7.35	7.33	7.16	6.98	6.8	6.65	6.49	6.34	6.18	6.03
773	3.24	4.84	6.93	7.35	7.33	7.16	6.98	6.8	6.65	6.49	6.33	6.18	6.02
774	3.24	4.8	6.92	7.35	7.33	7.16	6.98	6.8	6.65	6.49	6.34	6.18	6.03
775	3.24	4.68	6.79	7.07	6.93	6.75	6.57	6.39	6.24	6.08	5.93	5.77	5.61
776	3.24	4.64	6.76	7.07	6.93	6.75	6.57	6.39	6.24	6.08	5.93	5.78	5.62
777	3.24	4.67	6.78	7.07	6.93	6.75	6.57	6.39	6.24	6.08	5.92	5.77	5.61
778	3.24	4.65	6.76	7.07	6.93	6.75	6.57	6.39	6.24	6.08	5.93	5.77	5.62
779	3.24	4.62	6.74	7.07	6.93	6.75	6.57	6.4	6.24	6.08	5.93	5.77	5.61
780	3.24	4.64	6.76	7.07	6.93	6.75	6.57	6.39	6.24	6.08	5.93	5.77	5.62
781	3.24	4.64	6.76	7.07	6.93	6.75	6.57	6.4	6.24	6.08	5.93	5.77	5.61
782	3.24	4.61	6.73	7.07	6.93	6.75	6.57	6.39	6.24	6.08	5.93	5.77	5.62
783	3.24	4.65	6.76	7.07	6.93	6.75	6.57	6.39	6.24	6.08	5.93	5.77	5.61
784	3.24	4.39	6.47	6.88	6.73	6.55	6.37	6.19	6.04	5.88	5.73	5.57	5.41
785	3.24	4.43	6.49	6.88	6.73	6.55	6.37	6.19	6.03	5.88	5.72	5.56	5.4
786	3.24	4.29	6.39	6.88	6.73	6.55	6.37	6.19	6.04	5.88	5.73	5.57	5.42
787	3.24	4.29	6.37	6.88	6.73	6.55	6.37	6.2	6.04	5.88	5.73	5.57	5.42
788	3.24	4.35	6.44	6.88	6.73	6.55	6.37	6.19	6.04	5.88	5.73	5.57	5.41
789	3.24	4.25	6.34	6.88	6.73	6.55	6.37	6.19	6.04	5.88	5.73	5.57	5.42
790	3.24	4.42	6.5	6.88	6.73	6.55	6.37	6.19	6.04	5.88	5.73	5.57	5.41
791	3.24	4.37	6.46	6.88	6.73	6.55	6.37	6.19	6.04	5.88	5.72	5.57	5.41
792	3.24	4.27	6.37	6.88	6.73	6.55	6.37	6.19	6.04	5.88	5.73	5.57	5.42
793	3.24	4.91	6.97	7.41	7.38	7.09	6.71	6.31	5.95	5.58	5.21	4.83	4.48
794	3.24	4.9	6.96	7.41	7.38	7.09	6.72	6.32	5.96	5.59	5.22	4.85	4.49
795	3.24	4.89	6.96	7.41	7.38	7.09	6.72	6.32	5.96	5.59	5.22	4.84	4.45
796	3.24	4.89	6.96	7.41	7.38	7.09	6.71	6.31	5.95	5.58	5.21	4.85	4.48
797	3.24	4.9	6.96	7.41	7.38	7.09	6.71	6.31	5.95	5.58	5.21	4.83	4.46
798	3.24	4.91	6.96	7.41	7.38	7.09	6.72	6.31	5.95	5.58	5.2	4.82	4.44
799	3.24	4.88	6.95	7.41	7.38	7.09	6.72	6.32	5.95	5.59	5.21	4.84	4.46
800	3.24	4.93	6.97	7.42	7.38	7.09	6.71	6.31	5.95	5.58	5.21	4.83	4.46
801	3.24	4.92	6.97	7.42	7.38	7.09	6.71	6.31	5.95	5.58	5.21	4.84	4.48

802	3.24	4.82	6.92	7.29	7.09	6.68	6.25	5.83	5.45	5.08	4.7	4.31	3.92
803	3.24	4.79	6.91	7.29	7.09	6.68	6.25	5.83	5.46	5.08	4.71	4.33	3.91
804	3.24	4.78	6.9	7.29	7.09	6.68	6.26	5.82	5.44	5.07	4.71	4.33	3.96
805	3.24	4.82	6.91	7.29	7.09	6.68	6.25	5.82	5.44	5.07	4.7	4.32	3.93
806	3.24	4.85	6.92	7.29	7.08	6.68	6.25	5.82	5.45	5.08	4.7	4.33	3.98
807	3.24	4.82	6.91	7.29	7.08	6.68	6.25	5.83	5.45	5.07	4.69	4.31	3.95
808	3.24	4.83	6.92	7.29	7.09	6.68	6.25	5.82	5.44	5.06	4.68	4.3	3.92
809	3.24	4.86	6.93	7.29	7.08	6.68	6.25	5.82	5.44	5.05	4.68	4.28	3.84
810	3.24	4.88	6.93	7.29	7.08	6.68	6.24	5.81	5.44	5.06	4.68	4.31	3.9
811	3.24	4.63	6.74	7	6.64	6.21	5.78	5.35	4.98	4.61	4.24	3.86	3.42
812	3.24	4.62	6.74	7	6.64	6.21	5.78	5.35	4.98	4.59	4.22	3.86	3.47
813	3.24	4.64	6.75	7	6.64	6.21	5.78	5.35	4.97	4.6	4.23	3.84	3.46
814	3.24	4.62	6.73	7	6.64	6.21	5.78	5.35	4.98	4.61	4.23	3.85	3.51
815	3.24	4.65	6.75	7	6.64	6.21	5.78	5.35	4.98	4.6	4.22	3.85	3.53
816	3.24	4.65	6.75	7	6.64	6.21	5.78	5.35	4.99	4.63	4.26	3.87	3.54
817	3.24	4.63	6.73	7	6.64	6.21	5.78	5.35	4.98	4.59	4.23	3.81	3.46
818	3.24	4.63	6.73	7	6.64	6.21	5.78	5.35	4.98	4.61	4.22	3.83	3.45
819	3.24	4.57	6.69	7	6.64	6.21	5.78	5.35	4.98	4.6	4.22	3.85	3.52
820	3.24	4.38	6.44	6.8	6.43	6	5.57	5.14	4.76	4.41	4.01	3.6	3.22
821	3.24	4.41	6.47	6.8	6.43	6	5.57	5.14	4.77	4.39	4.01	3.63	3.23
822	3.24	4.36	6.43	6.8	6.43	6	5.57	5.14	4.77	4.39	4.02	3.63	3.22
823	3.24	4.35	6.43	6.8	6.43	6	5.57	5.14	4.76	4.39	4.01	3.61	3.18
824	3.24	4.43	6.49	6.8	6.42	6	5.57	5.14	4.77	4.38	3.99	3.59	3.17
825	3.24	4.36	6.42	6.8	6.43	6	5.57	5.14	4.77	4.39	4.01	3.63	3.29
826	3.24	4.37	6.44	6.8	6.43	6	5.57	5.14	4.77	4.39	4.03	3.67	3.28
827	3.24	4.37	6.43	6.8	6.43	6	5.57	5.14	4.77	4.38	4	3.61	3.23
828	3.24	4.41	6.47	6.8	6.43	6	5.57	5.15	4.77	4.4	4.02	3.67	3.3
829	3.24	4.2	6.24	6.76	6.38	5.96	5.52	5.09	4.71	4.34	3.98	3.61	3.21
830	3.24	4.22	6.28	6.76	6.38	5.95	5.52	5.09	4.72	4.34	3.96	3.63	3.26
831	3.24	4.23	6.29	6.76	6.38	5.95	5.53	5.09	4.73	4.35	3.96	3.57	3.21
832	3.24	4.31	6.35	6.75	6.38	5.95	5.52	5.09	4.72	4.36	3.98	3.6	3.23
833	3.24	4.24	6.3	6.76	6.38	5.95	5.53	5.09	4.72	4.36	3.97	3.59	3.21
834	3.24	4.25	6.3	6.76	6.38	5.95	5.53	5.1	4.73	4.34	3.95	3.54	3.21
835	3.24	4.12	6.17	6.76	6.39	5.96	5.53	5.11	4.73	4.36	4	3.61	3.21
836	3.24	4.23	6.28	6.76	6.38	5.95	5.53	5.1	4.73	4.36	3.95	3.56	3.15
837	3.24	4.27	6.32	6.75	6.38	5.95	5.52	5.1	4.73	4.36	3.97	3.59	3.2
838	3.24	4.79	6.9	7.23	6.85	6.21	5.52	4.84	4.26	3.66	3.03	2.52	1.97
839	3.24	4.85	6.91	7.23	6.85	6.21	5.53	4.84	4.23	3.71	3.15	2.6	2
840	3.24	4.84	6.91	7.23	6.85	6.21	5.52	4.82	4.21	3.66	3.08	2.62	2.17
841	3.24	4.87	6.92	7.23	6.85	6.21	5.53	4.84	4.24	3.65	3.07	2.26	2.09
842	3.24	4.83	6.91	7.23	6.85	6.21	5.53	4.84	4.23	3.65	2.97	2.36	1.48
843	3.24	4.78	6.9	7.23	6.85	6.21	5.53	4.84	4.25	3.64	3.02	2.46	2.07
844	3.24	4.85	6.91	7.23	6.85	6.21	5.53	4.85	4.23	3.56	2.88	2.36	1.43
845	3.24	4.83	6.91	7.23	6.85	6.21	5.53	4.83	4.22	3.61	2.99	2.34	1.51
846	3.24	4.85	6.91	7.23	6.85	6.21	5.53	4.83	4.23	3.68	3.16	2.69	2.4
847	3.24	4.65	6.73	6.92	6.34	5.65	4.96	4.28	3.66	3.03	2.35	1.76	0
848	3.24	4.61	6.7	6.92	6.34	5.64	4.96	4.28	3.7	3.14	2.48	1.78	0
849	3.24	4.65	6.74	6.92	6.34	5.65	4.98	4.29	3.69	3.1	2.55	1.94	0
850	3.24	4.6	6.71	6.92	6.34	5.65	4.96	4.26	3.65	3.07	2.47	1.95	1.36
851	3.24	4.62	6.72	6.92	6.34	5.65	4.96	4.26	3.62	3.03	2.54	1.86	1
852	3.24	4.62	6.72	6.92	6.34	5.65	4.97	4.27	3.66	3.09	2.48	1.76	1.48
853	3.24	4.66	6.75	6.92	6.33	5.64	4.95	4.28	3.68	3.03	2.39	1.45	1.08
854	3.24	4.58	6.68	6.93	6.34	5.65	4.96	4.29	3.66	3.05	2.37	1.89	1.34
855	3.24	4.58	6.69	6.92	6.34	5.65	4.95	4.25	3.67	3.06	2.5	2.05	1.51

856	3.24	4.38	6.43	6.72	6.12	5.43	4.75	4.06	3.47	2.93	2.31	1.15	0
857	3.24	4.38	6.43	6.72	6.12	5.43	4.75	4.06	3.45	2.93	2.2	1.45	0
858	3.24	4.33	6.37	6.73	6.13	5.44	4.75	4.07	3.46	2.74	2.14	1.56	0.3
859	3.24	4.36	6.4	6.72	6.12	5.44	4.75	4.04	3.44	2.84	2.23	1.34	1.15
860	3.24	4.3	6.36	6.73	6.12	5.43	4.74	4.04	3.44	2.82	2.31	1.8	1.18
861	3.24	4.37	6.42	6.72	6.12	5.44	4.75	4.04	3.42	2.76	2.15	1.71	1.08
862	3.24	4.42	6.46	6.72	6.12	5.43	4.74	4.04	3.41	2.84	2.13	1.28	0
863	3.24	4.4	6.45	6.72	6.12	5.43	4.75	4.06	3.47	2.91	2.42	1.89	1.11
864	3.24	4.31	6.36	6.73	6.13	5.44	4.76	4.08	3.49	2.82	2.25	1.87	1.48
865	3.24	4.62	6.71	6.9	6.23	5.45	4.68	3.92	3.28	2.64	1.18	0	0
866	3.24	4.56	6.66	6.9	6.24	5.47	4.68	3.87	3.14	2.25	0.3	0	0
867	3.24	4.63	6.72	6.9	6.24	5.46	4.67	3.9	3.26	2.64	1.84	0	0
868	3.24	4.58	6.68	6.9	6.24	5.46	4.7	3.92	3.27	2.72	1.77	1.36	1.49
869	3.24	4.61	6.7	6.9	6.24	5.46	4.68	3.88	3.21	2.57	2.06	1.72	1.36
870	3.24	4.65	6.73	6.89	6.23	5.45	4.69	3.88	3.14	2.35	1.87	1.34	0
871	3.24	4.62	6.71	6.9	6.24	5.45	4.67	3.92	3.25	2.63	2.03	1.65	1.32
872	3.24	4.59	6.69	6.9	6.24	5.46	4.68	3.92	3.22	2.63	1.79	0	0
873	3.24	4.62	6.71	6.9	6.24	5.46	4.67	3.91	3.24	2.54	1.62	0	0
874	3.24	4.71	6.87	7.07	6.92	6.75	6.57	6.39	6.23	6.08	5.92	5.76	5.61
875	3.24	4.72	6.86	7.07	6.92	6.74	6.57	6.39	6.23	6.08	5.92	5.77	5.61
876	3.24	4.66	6.85	7.07	6.92	6.75	6.57	6.39	6.23	6.08	5.92	5.77	5.61
877	3.24	4.75	6.87	7.07	6.92	6.75	6.57	6.39	6.23	6.08	5.92	5.76	5.61
878	3.24	4.74	6.87	7.07	6.92	6.75	6.57	6.39	6.23	6.08	5.92	5.77	5.61
879	3.24	4.77	6.88	7.07	6.92	6.75	6.57	6.39	6.23	6.08	5.92	5.77	5.61
880	3.24	4.75	6.87	7.07	6.92	6.75	6.57	6.39	6.23	6.08	5.92	5.77	5.61
881	3.24	4.78	6.88	7.07	6.92	6.74	6.57	6.39	6.23	6.08	5.92	5.76	5.61
882	3.24	4.7	6.86	7.07	6.92	6.75	6.57	6.39	6.23	6.08	5.92	5.77	5.61
883	3.24	4.93	6.98	7.29	7.08	6.67	6.24	5.81	5.43	5.06	4.7	4.33	3.94
884	3.24	4.94	6.98	7.29	7.08	6.67	6.25	5.82	5.45	5.07	4.68	4.31	3.94
885	3.24	4.96	6.99	7.29	7.08	6.67	6.24	5.82	5.44	5.06	4.69	4.33	3.97
886	3.24	4.94	6.98	7.29	7.08	6.67	6.24	5.82	5.44	5.06	4.69	4.3	3.92
887	3.24	4.95	6.98	7.29	7.08	6.67	6.24	5.81	5.44	5.07	4.7	4.33	3.98
888	3.24	4.93	6.98	7.29	7.08	6.67	6.24	5.82	5.44	5.05	4.68	4.31	3.92
889	3.24	4.91	6.97	7.29	7.08	6.67	6.24	5.82	5.45	5.08	4.7	4.34	3.96
890	3.24	4.9	6.97	7.29	7.08	6.67	6.24	5.82	5.44	5.07	4.7	4.32	3.91
891	3.24	4.92	6.97	7.29	7.08	6.67	6.25	5.82	5.44	5.07	4.7	4.33	3.99
892	3.24	4.73	6.86	6.99	6.63	6.2	5.77	5.34	4.97	4.59	4.22	3.82	3.43
893	3.24	4.69	6.85	6.99	6.63	6.2	5.77	5.34	4.97	4.58	4.2	3.83	3.46
894	3.24	4.71	6.85	6.99	6.63	6.2	5.77	5.34	4.96	4.6	4.2	3.8	3.44
895	3.24	4.72	6.86	6.99	6.63	6.2	5.77	5.34	4.96	4.57	4.21	3.81	3.42
896	3.24	4.81	6.87	6.99	6.62	6.19	5.77	5.34	4.97	4.61	4.23	3.85	3.52
897	3.24	4.74	6.86	6.99	6.63	6.2	5.77	5.34	4.98	4.6	4.22	3.85	3.44
898	3.24	4.76	6.86	6.99	6.63	6.19	5.77	5.34	4.96	4.58	4.19	3.81	3.45
899	3.24	4.71	6.86	6.99	6.63	6.2	5.77	5.35	4.98	4.6	4.21	3.82	3.43
900	3.24	4.78	6.87	6.99	6.62	6.19	5.76	5.34	4.95	4.57	4.19	3.79	3.41
901	3.24	4.41	6.58	6.8	6.42	5.99	5.57	5.13	4.77	4.38	4.03	3.67	3.32
902	3.24	4.53	6.68	6.79	6.42	5.99	5.56	5.14	4.77	4.39	4.02	3.67	3.25
903	3.24	4.48	6.65	6.79	6.42	5.99	5.56	5.13	4.75	4.38	4.01	3.61	3.21
904	3.24	4.47	6.64	6.79	6.42	5.99	5.56	5.13	4.75	4.39	4	3.62	3.23
905	3.24	4.42	6.6	6.8	6.42	5.99	5.57	5.14	4.76	4.39	4.01	3.64	3.28
906	3.24	4.53	6.68	6.79	6.42	5.99	5.56	5.13	4.75	4.38	4.01	3.65	3.34
907	3.24	4.52	6.68	6.79	6.42	5.99	5.56	5.13	4.76	4.38	4.01	3.66	3.32
908	3.24	4.41	6.6	6.8	6.42	5.99	5.57	5.13	4.75	4.38	4.01	3.6	3.18
909	3.24	4.47	6.63	6.79	6.42	5.99	5.56	5.12	4.74	4.38	4.01	3.63	3.23

910	3.24	4.74	6.85	6.91	6.32	5.63	4.93	4.21	3.62	2.88	2.2	0.85	0
911	3.24	4.74	6.85	6.91	6.32	5.63	4.93	4.25	3.68	3.05	2.44	1.75	0
912	3.24	4.76	6.85	6.91	6.32	5.63	4.94	4.24	3.65	3.03	2.34	0	0
913	3.24	4.75	6.85	6.9	6.32	5.63	4.95	4.26	3.6	2.96	2.28	1.23	0
914	3.24	4.69	6.84	6.91	6.32	5.64	4.95	4.28	3.7	3.04	2.34	1.72	1.45
915	3.24	4.75	6.85	6.91	6.32	5.63	4.95	4.26	3.65	2.99	2.49	1.99	1.85
916	3.24	4.74	6.85	6.91	6.32	5.63	4.93	4.25	3.65	3.09	2.53	1.83	1.34
917	3.24	4.72	6.85	6.91	6.32	5.63	4.94	4.26	3.66	3.05	2.48	1.23	0
918	3.24	4.7	6.84	6.91	6.32	5.64	4.95	4.28	3.69	3.08	2.58	1.79	0
919	3.24	4.75	6.87	6.99	6.62	6.2	5.77	5.34	4.97	4.6	4.22	3.85	3.5
920	3.24	4.77	6.87	6.99	6.62	6.2	5.77	5.33	4.96	4.59	4.2	3.83	3.4
921	3.24	4.75	6.87	6.99	6.62	6.2	5.77	5.34	4.97	4.6	4.23	3.86	3.48
922	3.24	4.74	6.87	6.99	6.62	6.2	5.77	5.34	4.97	4.59	4.22	3.84	3.45
923	3.24	4.78	6.88	6.99	6.62	6.19	5.76	5.34	4.95	4.57	4.2	3.85	3.48
924	3.24	4.79	6.88	6.99	6.62	6.19	5.76	5.33	4.95	4.57	4.19	3.81	3.45
925	3.24	4.8	6.88	6.99	6.62	6.19	5.77	5.35	4.97	4.59	4.22	3.85	3.45
926	3.24	4.78	6.88	6.99	6.62	6.19	5.76	5.34	4.96	4.58	4.21	3.84	3.45
927	3.24	4.76	6.87	6.99	6.62	6.2	5.77	5.34	4.97	4.59	4.22	3.82	3.43
928	3.24	4.24	5.97	7.02	6.69	6.26	5.83	5.4	5.03	4.66	4.3	3.94	3.54
929	3.24	4.26	5.99	7.02	6.69	6.26	5.83	5.41	5.04	4.65	4.28	3.91	3.49
930	3.24	4.25	5.98	7.02	6.69	6.26	5.84	5.41	5.03	4.66	4.29	3.93	3.52
931	3.24	4.19	5.91	7.02	6.69	6.26	5.84	5.41	5.04	4.67	4.28	3.91	3.53
932	3.24	4.27	5.98	7.02	6.69	6.26	5.83	5.4	5.02	4.65	4.28	3.91	3.57
933	3.24	4.27	6	7.02	6.69	6.26	5.83	5.4	5.03	4.67	4.31	3.93	3.55
934	3.24	4.25	5.97	7.02	6.69	6.26	5.84	5.41	5.02	4.66	4.29	3.88	3.52
935	3.24	4.23	5.95	7.02	6.69	6.26	5.84	5.41	5.04	4.67	4.29	3.95	3.58
936	3.24	4.26	5.98	7.02	6.69	6.26	5.83	5.4	5.03	4.65	4.28	3.89	3.49
937	3.24	3.38	4.06	5.09	6.92	6.67	6.24	5.81	5.43	5.06	4.69	4.31	3.94
938	3.24	3.36	4.02	5.03	6.9	6.68	6.25	5.82	5.45	5.07	4.7	4.31	3.94
939	3.24	3.45	4.16	5.15	6.93	6.66	6.23	5.8	5.43	5.05	4.68	4.33	3.96
940	3.24	3.44	4.12	5.12	6.92	6.66	6.23	5.81	5.43	5.06	4.68	4.28	3.91
941	3.24	3.42	4.12	5.13	6.92	6.66	6.23	5.8	5.43	5.06	4.67	4.29	3.89
942	3.24	3.41	4.14	5.14	6.93	6.66	6.23	5.8	5.43	5.04	4.68	4.31	3.96
943	3.24	3.36	4.08	5.11	6.92	6.66	6.23	5.81	5.44	5.06	4.69	4.33	3.96
944	3.24	3.44	4.1	5.1	6.92	6.66	6.24	5.81	5.43	5.06	4.69	4.33	3.97
945	3.24	3.49	4.22	5.21	6.94	6.65	6.22	5.79	5.42	5.04	4.67	4.29	3.93
946	3.24	3.66	4.73	6.28	7.02	6.84	6.66	6.48	6.33	6.17	6.02	5.86	5.71
947	3.24	3.69	4.78	6.32	7.01	6.84	6.66	6.48	6.33	6.17	6.01	5.86	5.7
948	3.24	3.69	4.76	6.3	7.02	6.84	6.66	6.48	6.33	6.17	6.02	5.86	5.7
949	3.24	3.61	4.72	6.26	7.02	6.84	6.66	6.48	6.33	6.17	6.02	5.86	5.71
950	3.24	3.77	4.86	6.39	7.01	6.83	6.66	6.48	6.32	6.17	6.01	5.85	5.7
951	3.24	3.67	4.73	6.28	7.02	6.84	6.66	6.48	6.33	6.17	6.02	5.86	5.71
952	3.24	3.77	4.81	6.34	7.01	6.84	6.66	6.48	6.33	6.17	6.01	5.86	5.7
953	3.24	3.69	4.77	6.32	7.01	6.84	6.66	6.48	6.32	6.17	6.01	5.86	5.7
954	3.24	3.68	4.75	6.3	7.02	6.84	6.66	6.48	6.33	6.17	6.01	5.86	5.71
955	3.24	3.94	5.1	6.75	7.22	6.85	6.43	6	5.62	5.25	4.87	4.49	4.06
956	3.24	3.96	5.14	6.78	7.22	6.85	6.42	5.99	5.62	5.25	4.87	4.5	4.12
957	3.24	3.94	5.11	6.76	7.22	6.85	6.43	6	5.62	5.25	4.88	4.51	4.15
958	3.24	3.99	5.16	6.79	7.22	6.85	6.42	6	5.62	5.26	4.89	4.52	4.15
959	3.24	3.99	5.17	6.8	7.22	6.84	6.42	5.99	5.62	5.24	4.86	4.49	4.09
960	3.24	3.91	5.09	6.75	7.22	6.85	6.43	6	5.62	5.25	4.87	4.5	4.11
961	3.24	3.94	5.12	6.77	7.22	6.85	6.42	5.99	5.61	5.23	4.86	4.48	4.06
962	3.24	3.93	5.12	6.76	7.22	6.85	6.43	6.01	5.63	5.25	4.88	4.51	4.15
963	3.24	3.95	5.14	6.78	7.22	6.85	6.43	6	5.62	5.25	4.88	4.54	4.15

964	3.24	3.67	4.72	6.22	6.86	6.43	6	5.57	5.2	4.83	4.45	4.1	3.75
965	3.24	3.71	4.78	6.28	6.85	6.42	6	5.57	5.19	4.81	4.42	4.05	3.69
966	3.24	3.63	4.66	6.17	6.86	6.44	6.01	5.58	5.2	4.84	4.45	4.08	3.71
967	3.24	3.69	4.72	6.23	6.86	6.43	6	5.58	5.2	4.83	4.45	4.08	3.71
968	3.24	3.61	4.65	6.17	6.86	6.44	6.01	5.58	5.2	4.82	4.45	4.08	3.69
969	3.24	3.7	4.74	6.24	6.85	6.43	6	5.57	5.19	4.82	4.44	4.07	3.66
970	3.24	3.65	4.72	6.23	6.86	6.43	6	5.58	5.21	4.82	4.45	4.08	3.69
971	3.24	3.62	4.68	6.19	6.86	6.43	6	5.57	5.2	4.81	4.44	4.06	3.7
972	3.24	3.69	4.69	6.2	6.86	6.43	6	5.57	5.2	4.82	4.44	4.07	3.66
973	3.24	3.36	4.28	5.61	6.71	6.29	5.86	5.43	5.06	4.7	4.33	3.95	3.58
974	3.24	3.46	4.39	5.72	6.7	6.27	5.84	5.42	5.04	4.66	4.29	3.89	3.51
975	3.24	3.42	4.34	5.69	6.7	6.28	5.85	5.42	5.04	4.66	4.28	3.92	3.54
976	3.24	3.42	4.35	5.69	6.7	6.28	5.85	5.42	5.05	4.68	4.29	3.91	3.53
977	3.24	3.35	4.29	5.63	6.71	6.28	5.85	5.42	5.05	4.68	4.29	3.92	3.57
978	3.24	3.39	4.34	5.69	6.71	6.28	5.85	5.42	5.04	4.66	4.28	3.92	3.55
979	3.24	3.44	4.37	5.7	6.7	6.27	5.84	5.42	5.04	4.66	4.27	3.87	3.48
980	3.24	3.4	4.33	5.66	6.71	6.28	5.85	5.43	5.05	4.68	4.31	3.95	3.59
981	3.24	3.48	4.4	5.75	6.7	6.27	5.84	5.42	5.04	4.66	4.27	3.9	3.53
982	3.24	3.72	4.74	6.2	6.7	6.02	5.33	4.66	4.04	3.42	2.77	2.2	1.59
983	3.24	3.66	4.66	6.13	6.71	6.03	5.34	4.64	4.03	3.41	2.75	1.88	0
984	3.24	3.67	4.68	6.15	6.71	6.02	5.33	4.65	4.03	3.38	2.88	2.18	1.56
985	3.24	3.69	4.73	6.18	6.7	6.01	5.32	4.64	4.04	3.47	2.95	2.53	2.02
986	3.24	3.65	4.68	6.15	6.71	6.02	5.34	4.65	4.09	3.43	2.67	2.08	1.38
987	3.24	3.68	4.69	6.16	6.71	6.02	5.34	4.66	4.03	3.43	2.93	2.4	1.2
988	3.24	3.64	4.67	6.14	6.71	6.03	5.33	4.63	4.01	3.37	2.86	2.23	1.86
989	3.24	3.66	4.67	6.14	6.71	6.02	5.33	4.64	4.04	3.42	2.74	2.26	1.83
990	3.24	3.71	4.75	6.21	6.7	6.01	5.32	4.63	4.03	3.4	2.76	2.13	1.3
991	3.24	4.2	5.95	7.09	7.04	6.94	6.85	6.76	6.67	6.59	6.51	6.42	6.34
992	3.24	4.3	6.04	7.09	7.04	6.94	6.85	6.75	6.67	6.59	6.51	6.43	6.34
993	3.24	4.25	5.99	7.09	7.04	6.94	6.85	6.75	6.67	6.59	6.51	6.42	6.34
994	3.24	4.3	6.04	7.09	7.04	6.94	6.85	6.75	6.67	6.59	6.51	6.42	6.34
995	3.24	4.24	5.98	7.09	7.04	6.94	6.85	6.75	6.67	6.59	6.51	6.42	6.34
996	3.24	4.23	5.98	7.09	7.04	6.94	6.85	6.75	6.67	6.59	6.51	6.42	6.34
997	3.24	4.28	6.04	7.09	7.04	6.94	6.85	6.75	6.67	6.59	6.51	6.42	6.34
998	3.24	4.25	5.99	7.09	7.04	6.94	6.85	6.75	6.67	6.59	6.51	6.42	6.34
999	3.24	4.24	5.98	7.09	7.04	6.94	6.85	6.76	6.67	6.59	6.51	6.43	6.34
1000	3.24	4.47	6.31	7.31	7.34	7.18	7	6.82	6.66	6.51	6.35	6.19	6.04
1001	3.24	4.47	6.3	7.31	7.34	7.18	7	6.82	6.67	6.51	6.35	6.2	6.04
1002	3.24	4.47	6.31	7.31	7.34	7.18	7	6.82	6.66	6.51	6.35	6.2	6.04
1003	3.24	4.44	6.28	7.31	7.34	7.18	7	6.82	6.67	6.51	6.36	6.2	6.04
1004	3.24	4.48	6.31	7.31	7.34	7.18	7	6.82	6.67	6.51	6.35	6.2	6.04
1005	3.24	4.45	6.28	7.31	7.34	7.18	7	6.82	6.67	6.51	6.35	6.2	6.05
1006	3.24	4.53	6.37	7.32	7.34	7.17	7	6.82	6.66	6.51	6.35	6.2	6.04
1007	3.24	4.42	6.25	7.31	7.34	7.18	7	6.82	6.66	6.51	6.36	6.2	6.04
1008	3.24	4.46	6.29	7.31	7.34	7.18	7	6.82	6.67	6.51	6.36	6.2	6.04
1009	3.24	4.22	5.96	7.07	6.95	6.77	6.59	6.42	6.26	6.11	5.95	5.79	5.64
1010	3.24	4.29	6.02	7.07	6.95	6.77	6.59	6.41	6.26	6.1	5.95	5.79	5.64
1011	3.24	4.33	6.07	7.07	6.95	6.77	6.59	6.41	6.26	6.1	5.94	5.79	5.63
1012	3.24	4.21	5.95	7.07	6.95	6.77	6.6	6.42	6.26	6.11	5.95	5.8	5.64
1013	3.24	4.23	5.98	7.07	6.95	6.77	6.59	6.41	6.26	6.1	5.95	5.79	5.64
1014	3.24	4.26	6	7.07	6.95	6.77	6.59	6.42	6.26	6.11	5.95	5.79	5.64
1015	3.24	4.26	6	7.07	6.95	6.77	6.59	6.41	6.26	6.1	5.95	5.79	5.64
1016	3.24	4.29	6.02	7.07	6.95	6.77	6.59	6.42	6.26	6.1	5.95	5.79	5.63
1017	3.24	4.15	5.89	7.07	6.95	6.77	6.59	6.42	6.26	6.1	5.95	5.79	5.63

1018	3.24	3.96	5.62	6.9	6.75	6.57	6.4	6.22	6.06	5.91	5.75	5.6	5.44
1019	3.24	3.92	5.55	6.9	6.75	6.58	6.4	6.22	6.06	5.91	5.75	5.6	5.44
1020	3.24	3.98	5.62	6.9	6.75	6.57	6.4	6.22	6.06	5.91	5.75	5.6	5.44
1021	3.24	3.97	5.61	6.9	6.75	6.57	6.4	6.22	6.06	5.91	5.75	5.6	5.45
1022	3.24	3.97	5.62	6.9	6.75	6.57	6.4	6.22	6.06	5.91	5.75	5.59	5.44
1023	3.24	3.89	5.53	6.9	6.75	6.58	6.4	6.22	6.06	5.91	5.75	5.6	5.44
1024	3.24	4.01	5.63	6.9	6.75	6.57	6.39	6.22	6.06	5.91	5.75	5.6	5.44
1025	3.24	3.94	5.59	6.9	6.75	6.57	6.4	6.22	6.06	5.9	5.75	5.6	5.44
1026	3.24	4.01	5.66	6.9	6.75	6.57	6.39	6.22	6.06	5.9	5.75	5.59	5.44
1027	3.24	4.56	6.41	7.37	7.4	7.12	6.75	6.35	5.99	5.62	5.25	4.88	4.52
1028	3.24	4.53	6.38	7.37	7.41	7.12	6.75	6.35	5.99	5.62	5.25	4.87	4.48
1029	3.24	4.58	6.43	7.37	7.4	7.12	6.75	6.36	6	5.63	5.26	4.89	4.51
1030	3.24	4.55	6.4	7.37	7.4	7.12	6.75	6.35	5.99	5.62	5.25	4.89	4.53
1031	3.24	4.57	6.42	7.37	7.4	7.12	6.75	6.35	5.99	5.62	5.23	4.85	4.48
1032	3.24	4.54	6.39	7.37	7.4	7.12	6.75	6.36	6	5.63	5.26	4.88	4.51
1033	3.24	4.55	6.4	7.37	7.4	7.12	6.75	6.35	5.99	5.62	5.25	4.87	4.49
1034	3.24	4.57	6.42	7.37	7.4	7.12	6.75	6.36	5.99	5.63	5.26	4.88	4.51
1035	3.24	4.58	6.43	7.37	7.4	7.12	6.75	6.35	5.99	5.62	5.25	4.88	4.49
1036	3.24	4.52	6.33	7.27	7.12	6.72	6.3	5.86	5.49	5.12	4.74	4.36	4
1037	3.24	4.42	6.24	7.26	7.13	6.72	6.3	5.87	5.49	5.11	4.72	4.34	3.96
1038	3.24	4.5	6.32	7.27	7.12	6.72	6.3	5.87	5.5	5.12	4.75	4.36	3.97
1039	3.24	4.42	6.25	7.26	7.12	6.72	6.3	5.87	5.49	5.11	4.73	4.34	3.91
1040	3.24	4.5	6.32	7.27	7.12	6.72	6.3	5.87	5.5	5.1	4.73	4.37	4.01
1041	3.24	4.48	6.3	7.27	7.12	6.72	6.3	5.87	5.49	5.11	4.75	4.37	4
1042	3.24	4.45	6.27	7.27	7.12	6.72	6.3	5.86	5.48	5.11	4.74	4.37	4.02
1043	3.24	4.45	6.27	7.27	7.12	6.72	6.3	5.86	5.49	5.1	4.73	4.33	3.94
1044	3.24	4.45	6.27	7.27	7.12	6.72	6.3	5.87	5.49	5.11	4.73	4.36	4
1045	3.24	4.2	5.93	7.02	6.69	6.26	5.83	5.4	5.02	4.65	4.27	3.9	3.49
1046	3.24	4.21	5.94	7.02	6.69	6.26	5.84	5.41	5.03	4.65	4.27	3.91	3.6
1047	3.24	4.25	5.96	7.02	6.69	6.26	5.83	5.41	5.02	4.65	4.3	3.93	3.56
1048	3.24	4.25	5.97	7.02	6.69	6.26	5.83	5.41	5.03	4.65	4.25	3.89	3.51
1049	3.24	4.24	5.97	7.02	6.69	6.26	5.83	5.41	5.03	4.66	4.28	3.9	3.52
1050	3.24	4.28	6.01	7.02	6.69	6.26	5.83	5.4	5.03	4.66	4.28	3.93	3.5
1051	3.24	4.3	6.02	7.02	6.69	6.26	5.83	5.39	5.02	4.64	4.25	3.86	3.47
1052	3.24	4.26	5.99	7.02	6.69	6.26	5.83	5.4	5.04	4.65	4.29	3.91	3.51
1053	3.24	4.22	5.94	7.02	6.69	6.26	5.84	5.41	5.03	4.66	4.3	3.94	3.53
1054	3.24	3.95	5.56	6.86	6.49	6.07	5.64	5.21	4.83	4.45	4.08	3.67	3.31
1055	3.24	3.99	5.61	6.86	6.49	6.06	5.63	5.2	4.83	4.45	4.09	3.73	3.34
1056	3.24	3.87	5.51	6.86	6.5	6.07	5.64	5.21	4.83	4.45	4.08	3.69	3.25
1057	3.24	3.97	5.59	6.86	6.49	6.06	5.63	5.2	4.83	4.45	4.05	3.65	3.33
1058	3.24	3.97	5.6	6.86	6.49	6.06	5.63	5.21	4.84	4.47	4.08	3.7	3.34
1059	3.24	3.92	5.55	6.86	6.49	6.06	5.64	5.21	4.83	4.45	4.1	3.71	3.31
1060	3.24	3.95	5.57	6.86	6.49	6.06	5.64	5.2	4.82	4.44	4.05	3.68	3.32
1061	3.24	3.88	5.5	6.86	6.5	6.07	5.64	5.2	4.83	4.46	4.1	3.75	3.41
1062	3.24	3.92	5.53	6.86	6.5	6.07	5.64	5.21	4.85	4.47	4.1	3.7	3.37
1063	3.24	3.92	5.51	6.81	6.44	6.01	5.58	5.15	4.78	4.41	4.03	3.63	3.28
1064	3.24	3.92	5.51	6.81	6.44	6.01	5.58	5.15	4.78	4.39	4.03	3.65	3.31
1065	3.24	3.81	5.39	6.82	6.45	6.02	5.59	5.16	4.79	4.42	4.07	3.66	3.28
1066	3.24	3.84	5.43	6.81	6.45	6.02	5.6	5.16	4.79	4.41	4.04	3.64	3.26
1067	3.24	3.86	5.44	6.81	6.45	6.02	5.59	5.16	4.79	4.42	4.03	3.65	3.23
1068	3.24	3.79	5.37	6.82	6.45	6.02	5.59	5.16	4.78	4.4	4.03	3.7	3.34
1069	3.24	3.86	5.44	6.81	6.45	6.02	5.59	5.16	4.79	4.43	4.04	3.65	3.31
1070	3.24	3.73	5.33	6.82	6.45	6.03	5.6	5.17	4.79	4.41	4.03	3.66	3.3
1071	3.24	3.82	5.42	6.82	6.45	6.02	5.59	5.16	4.79	4.41	4.03	3.66	3.29

1072	3.24	4.48	6.29	7.22	6.91	6.28	5.59	4.89	4.28	3.67	3.02	2.19	1.08
1073	3.24	4.49	6.3	7.22	6.91	6.28	5.6	4.91	4.32	3.7	3.12	2.51	1.96
1074	3.24	4.49	6.29	7.22	6.91	6.27	5.59	4.92	4.33	3.69	3.07	2.56	1.75
1075	3.24	4.45	6.25	7.22	6.91	6.28	5.6	4.92	4.32	3.72	3.09	2.33	1.89
1076	3.24	4.52	6.33	7.22	6.91	6.27	5.59	4.89	4.3	3.69	3.13	2.38	2.08
1077	3.24	4.42	6.22	7.22	6.92	6.28	5.61	4.92	4.3	3.7	3.14	2.51	1.95
1078	3.24	4.45	6.26	7.22	6.91	6.28	5.6	4.91	4.3	3.7	3.11	2.56	2.11
1079	3.24	4.46	6.27	7.22	6.91	6.28	5.59	4.89	4.29	3.64	3.02	2.43	1.83
1080	3.24	4.46	6.26	7.22	6.91	6.28	5.6	4.91	4.31	3.73	3.16	2.64	1.98
1081	3.24	4.16	5.86	6.98	6.43	5.75	5.07	4.37	3.81	3.23	2.67	2.05	1.58
1082	3.24	4.25	5.95	6.97	6.42	5.74	5.04	4.35	3.74	3.2	2.51	1.68	0.3
1083	3.24	4.22	5.92	6.97	6.43	5.74	5.05	4.34	3.77	3.14	2.72	2.07	1.95
1084	3.24	4.26	5.96	6.97	6.42	5.73	5.05	4.36	3.74	3.17	2.56	2.01	0
1085	3.24	4.27	5.97	6.97	6.42	5.73	5.04	4.33	3.7	3.08	2.47	2.04	0
1086	3.24	4.25	5.95	6.97	6.42	5.74	5.04	4.34	3.8	3.22	2.7	2.18	1.41
1087	3.24	4.26	5.97	6.97	6.42	5.73	5.04	4.35	3.76	3.14	2.33	1.66	1.32
1088	3.24	4.26	5.97	6.97	6.42	5.74	5.05	4.36	3.78	3.13	2.6	1.83	1.4
1089	3.24	4.26	5.96	6.97	6.42	5.74	5.04	4.35	3.71	3.14	2.64	1.78	0
1090	3.24	4.1	5.7	6.8	6.21	5.52	4.85	4.13	3.53	2.86	2.25	1.2	0
1091	3.24	3.92	5.51	6.82	6.23	5.54	4.84	4.17	3.54	2.97	2.4	1.96	1.48
1092	3.24	3.9	5.51	6.82	6.23	5.54	4.86	4.18	3.58	2.98	2.36	1.88	1.26
1093	3.24	3.96	5.57	6.82	6.22	5.54	4.85	4.16	3.55	2.98	2.4	1.91	1.2
1094	3.24	3.92	5.53	6.82	6.23	5.54	4.86	4.18	3.54	2.88	2.35	1.2	0
1095	3.24	4.04	5.63	6.81	6.22	5.53	4.84	4.17	3.49	2.95	2.34	1.58	0.3
1096	3.24	3.93	5.53	6.82	6.23	5.54	4.85	4.17	3.57	2.96	2.31	1.23	0
1097	3.24	3.97	5.58	6.81	6.22	5.53	4.85	4.15	3.56	2.98	2.43	1.79	1.04
1098	3.24	4.02	5.62	6.81	6.22	5.53	4.84	4.14	3.56	2.98	2.47	1.62	1.15
1099	3.24	4.25	5.95	6.96	6.33	5.55	4.76	3.99	3.28	2.63	2.01	0.3	0
1100	3.24	4.18	5.88	6.96	6.34	5.56	4.78	3.98	3.34	2.69	1.85	0	0
1101	3.24	4.25	5.95	6.96	6.33	5.55	4.76	3.98	3.33	2.62	2.02	0.3	0
1102	3.24	4.22	5.93	6.96	6.33	5.56	4.78	4	3.34	2.59	1.23	0	0
1103	3.24	4.24	5.94	6.96	6.33	5.55	4.77	3.99	3.37	2.78	2.07	0	0
1104	3.24	4.26	5.95	6.96	6.33	5.55	4.78	4	3.24	2.1	1.41	0	0
1105	3.24	4.26	5.95	6.96	6.33	5.56	4.79	3.94	3.33	2.66	1.74	0	0
1106	3.24	4.22	5.92	6.96	6.33	5.55	4.78	4	3.3	2.55	1.7	1.04	0
1107	3.24	4.27	5.96	6.96	6.33	5.55	4.76	3.97	3.3	2.74	1.9	1.6	0
1108	3.24	4.61	6.74	7.07	6.93	6.75	6.57	6.39	6.24	6.08	5.93	5.77	5.61
1109	3.24	4.65	6.76	7.07	6.93	6.75	6.57	6.39	6.24	6.08	5.93	5.77	5.62
1110	3.24	4.62	6.74	7.07	6.93	6.75	6.57	6.39	6.24	6.08	5.93	5.77	5.62
1111	3.24	4.68	6.79	7.07	6.93	6.75	6.57	6.39	6.24	6.08	5.93	5.77	5.61
1112	3.24	4.66	6.77	7.07	6.93	6.75	6.57	6.39	6.24	6.08	5.93	5.77	5.62
1113	3.24	4.69	6.8	7.07	6.93	6.75	6.57	6.39	6.24	6.08	5.92	5.76	5.61
1114	3.24	4.65	6.77	7.07	6.93	6.75	6.57	6.39	6.24	6.08	5.92	5.77	5.61
1115	3.24	4.65	6.76	7.07	6.93	6.75	6.57	6.4	6.24	6.08	5.93	5.77	5.62
1116	3.24	4.65	6.77	7.07	6.93	6.75	6.57	6.39	6.24	6.08	5.93	5.77	5.62
1117	3.24	4.83	6.92	7.29	7.09	6.68	6.25	5.82	5.46	5.09	4.71	4.34	3.93
1118	3.24	4.85	6.92	7.29	7.08	6.67	6.25	5.82	5.45	5.07	4.7	4.35	4
1119	3.24	4.83	6.92	7.29	7.09	6.68	6.25	5.83	5.45	5.08	4.69	4.3	3.89
1120	3.24	4.84	6.92	7.29	7.09	6.68	6.25	5.82	5.44	5.07	4.69	4.32	3.9
1121	3.24	4.83	6.92	7.29	7.08	6.68	6.25	5.83	5.45	5.07	4.69	4.33	3.95
1122	3.24	4.86	6.92	7.29	7.09	6.68	6.25	5.83	5.45	5.07	4.7	4.34	3.96
1123	3.24	4.83	6.92	7.29	7.09	6.68	6.25	5.82	5.45	5.07	4.71	4.34	3.97
1124	3.24	4.87	6.93	7.29	7.09	6.68	6.25	5.83	5.46	5.09	4.7	4.33	3.99
1125	3.24	4.87	6.93	7.29	7.08	6.68	6.25	5.82	5.45	5.08	4.69	4.3	3.95

1126	3.24	4.67	6.77	7	6.64	6.21	5.78	5.35	4.97	4.6	4.23	3.85	3.5
1127	3.24	4.64	6.74	7	6.64	6.21	5.78	5.34	4.97	4.59	4.22	3.85	3.51
1128	3.24	4.65	6.75	7	6.64	6.21	5.78	5.35	4.97	4.6	4.21	3.78	3.42
1129	3.24	4.61	6.72	7	6.64	6.21	5.77	5.35	4.97	4.6	4.21	3.81	3.49
1130	3.24	4.59	6.71	7	6.64	6.21	5.78	5.35	4.98	4.61	4.23	3.89	3.53
1131	3.24	4.58	6.7	7	6.64	6.21	5.79	5.36	4.98	4.62	4.25	3.83	3.45
1132	3.24	4.58	6.71	7	6.64	6.21	5.78	5.35	4.97	4.59	4.21	3.85	3.51
1133	3.24	4.65	6.74	7	6.64	6.21	5.78	5.35	4.98	4.6	4.21	3.83	3.48
1134	3.24	4.63	6.74	7	6.64	6.21	5.78	5.35	4.98	4.6	4.22	3.83	3.53
1135	3.24	4.37	6.44	6.8	6.43	6	5.57	5.14	4.78	4.4	4.02	3.61	3.21
1136	3.24	4.4	6.46	6.8	6.43	6	5.57	5.14	4.78	4.41	4.04	3.66	3.23
1137	3.24	4.44	6.5	6.8	6.42	5.99	5.57	5.14	4.77	4.38	4	3.61	3.29
1138	3.24	4.37	6.45	6.8	6.43	6	5.57	5.15	4.78	4.41	4.05	3.67	3.3
1139	3.24	4.41	6.47	6.8	6.43	6	5.57	5.14	4.76	4.38	4.03	3.66	3.32
1140	3.24	4.33	6.4	6.8	6.43	6	5.58	5.15	4.78	4.4	4.01	3.62	3.25
1141	3.24	4.32	6.39	6.8	6.43	6	5.57	5.15	4.77	4.4	4.03	3.68	3.31
1142	3.24	4.37	6.43	6.8	6.43	6	5.57	5.15	4.77	4.39	4.02	3.63	3.24
1143	3.24	4.36	6.43	6.8	6.43	6	5.57	5.14	4.77	4.4	4	3.63	3.21
1144	3.24	4.69	6.77	6.92	6.33	5.64	4.96	4.28	3.71	3.03	2.32	1.52	0
1145	3.24	4.64	6.72	6.92	6.34	5.65	4.96	4.27	3.67	3.12	2.59	2.12	1.62
1146	3.24	4.58	6.69	6.93	6.34	5.65	4.97	4.26	3.66	3.08	2.4	1.92	1.46
1147	3.24	4.59	6.69	6.92	6.34	5.66	4.98	4.29	3.72	3.14	2.71	2.07	1.36
1148	3.24	4.6	6.7	6.92	6.34	5.65	4.96	4.27	3.65	3.01	2.5	2.1	1.7
1149	3.24	4.54	6.65	6.93	6.35	5.65	4.97	4.26	3.62	3.01	2.54	1.99	1.46
1150	3.24	4.62	6.72	6.92	6.34	5.65	4.97	4.29	3.66	3.04	2.48	1.84	1.45
1151	3.24	4.57	6.68	6.93	6.34	5.65	4.96	4.27	3.7	3.12	2.52	1.99	0.48
1152	3.24	4.61	6.72	6.92	6.34	5.65	4.96	4.26	3.64	3.08	2.43	2.06	1.43
1153	3.24	4.66	6.85	6.99	6.63	6.2	5.77	5.34	4.95	4.58	4.2	3.83	3.39
1154	3.24	4.71	6.86	6.99	6.63	6.2	5.77	5.34	4.98	4.61	4.23	3.87	3.51
1155	3.24	4.72	6.86	6.99	6.63	6.2	5.77	5.34	4.96	4.58	4.22	3.83	3.5
1156	3.24	4.76	6.86	6.99	6.63	6.19	5.77	5.34	4.96	4.59	4.21	3.85	3.49
1157	3.24	4.73	6.86	6.99	6.63	6.2	5.77	5.34	4.96	4.6	4.23	3.87	3.44
1158	3.24	4.75	6.86	6.99	6.63	6.2	5.77	5.34	4.96	4.59	4.2	3.81	3.49
1159	3.24	4.73	6.86	6.99	6.63	6.2	5.77	5.34	4.95	4.57	4.2	3.82	3.41
1160	3.24	4.73	6.86	6.99	6.63	6.2	5.77	5.35	4.97	4.58	4.21	3.84	3.46
1161	3.24	4.75	6.86	6.99	6.63	6.2	5.77	5.34	4.96	4.59	4.23	3.84	3.46
1162	3.24	3.71	4.77	6.27	6.85	6.43	6	5.57	5.2	4.82	4.45	4.09	3.71
1163	3.24	3.72	4.75	6.26	6.85	6.43	6	5.56	5.2	4.82	4.44	4.1	3.74
1164	3.24	3.66	4.68	6.18	6.86	6.43	6	5.57	5.21	4.83	4.43	4.05	3.71
1165	3.24	3.73	4.78	6.28	6.85	6.42	6	5.57	5.19	4.82	4.45	4.08	3.72
1166	3.24	3.69	4.72	6.24	6.86	6.43	6	5.57	5.2	4.82	4.44	4.08	3.69
1167	3.24	3.72	4.77	6.27	6.85	6.43	6	5.57	5.2	4.82	4.45	4.08	3.7
1168	3.24	3.61	4.65	6.16	6.86	6.44	6.01	5.58	5.19	4.82	4.44	4.05	3.65
1169	3.24	3.71	4.72	6.22	6.86	6.43	6	5.57	5.19	4.82	4.45	4.06	3.69
1170	3.24	3.77	4.83	6.32	6.85	6.42	5.99	5.56	5.19	4.81	4.45	4.07	3.68
1171	3.24	4.22	5.95	7.07	6.95	6.77	6.59	6.42	6.26	6.11	5.95	5.79	5.64
1172	3.24	4.24	5.98	7.07	6.95	6.77	6.59	6.42	6.26	6.1	5.95	5.79	5.64
1173	3.24	4.29	6.02	7.07	6.95	6.77	6.59	6.42	6.26	6.1	5.95	5.79	5.63
1174	3.24	4.28	6.03	7.07	6.95	6.77	6.59	6.41	6.26	6.1	5.95	5.79	5.64
1175	3.24	4.22	5.96	7.07	6.95	6.77	6.6	6.42	6.26	6.11	5.95	5.79	5.64
1176	3.24	4.24	5.98	7.07	6.95	6.77	6.59	6.41	6.26	6.1	5.94	5.79	5.64
1177	3.24	4.3	6.04	7.07	6.95	6.77	6.59	6.41	6.26	6.1	5.95	5.79	5.64
1178	3.24	4.2	5.94	7.07	6.95	6.77	6.6	6.42	6.26	6.11	5.95	5.8	5.64
1179	3.24	4.17	5.92	7.07	6.95	6.77	6.6	6.42	6.26	6.11	5.95	5.79	5.64

1180	3.24	4.43	6.26	7.26	7.12	6.72	6.3	5.88	5.5	5.12	4.75	4.37	3.99
1181	3.24	4.45	6.27	7.27	7.12	6.72	6.3	5.87	5.49	5.11	4.74	4.36	3.97
1182	3.24	4.46	6.29	7.27	7.12	6.72	6.29	5.87	5.5	5.12	4.75	4.36	4.02
1183	3.24	4.52	6.33	7.27	7.12	6.72	6.29	5.86	5.49	5.11	4.72	4.33	3.95
1184	3.24	4.45	6.27	7.27	7.12	6.72	6.29	5.87	5.49	5.12	4.76	4.38	3.97
1185	3.24	4.5	6.32	7.27	7.12	6.72	6.29	5.86	5.48	5.1	4.73	4.38	4
1186	3.24	4.48	6.3	7.27	7.12	6.72	6.3	5.87	5.49	5.12	4.74	4.36	3.99
1187	3.24	4.46	6.29	7.27	7.12	6.72	6.3	5.86	5.49	5.12	4.75	4.39	4
1188	3.24	4.49	6.31	7.27	7.12	6.72	6.29	5.86	5.49	5.12	4.74	4.35	3.95
1189	3.24	4.31	6.02	7.02	6.69	6.26	5.83	5.4	5.03	4.65	4.26	3.89	3.48
1190	3.24	4.21	5.93	7.02	6.69	6.26	5.83	5.41	5.03	4.67	4.3	3.93	3.56
1191	3.24	4.21	5.93	7.02	6.69	6.26	5.84	5.41	5.04	4.66	4.28	3.9	3.51
1192	3.24	4.19	5.91	7.02	6.69	6.26	5.84	5.41	5.04	4.66	4.29	3.91	3.53
1193	3.24	4.15	5.87	7.02	6.69	6.27	5.84	5.41	5.04	4.67	4.3	3.91	3.52
1194	3.24	4.23	5.96	7.02	6.69	6.26	5.83	5.4	5.03	4.66	4.29	3.93	3.57
1195	3.24	4.16	5.88	7.02	6.69	6.27	5.84	5.41	5.03	4.66	4.3	3.89	3.5
1196	3.24	4.28	6.01	7.02	6.69	6.26	5.83	5.4	5.01	4.64	4.28	3.93	3.57
1197	3.24	4.24	5.97	7.02	6.69	6.26	5.83	5.41	5.03	4.66	4.27	3.9	3.51
1198	3.24	4.01	5.64	6.85	6.49	6.06	5.64	5.21	4.84	4.47	4.11	3.71	3.34
1199	3.24	3.98	5.61	6.86	6.49	6.06	5.64	5.2	4.84	4.46	4.09	3.7	3.3
1200	3.24	3.95	5.57	6.86	6.49	6.06	5.64	5.21	4.83	4.44	4.05	3.65	3.24
1201	3.24	3.88	5.48	6.86	6.5	6.07	5.64	5.21	4.84	4.47	4.08	3.72	3.33
1202	3.24	3.99	5.61	6.86	6.49	6.06	5.63	5.21	4.83	4.45	4.09	3.71	3.32
1203	3.24	3.93	5.55	6.86	6.49	6.07	5.64	5.2	4.83	4.45	4.08	3.69	3.32
1204	3.24	3.89	5.51	6.86	6.5	6.07	5.64	5.21	4.84	4.47	4.1	3.73	3.32
1205	3.24	4.05	5.66	6.85	6.48	6.06	5.63	5.2	4.82	4.43	4.04	3.67	3.32
1206	3.24	3.92	5.53	6.86	6.49	6.06	5.63	5.2	4.82	4.43	4.05	3.69	3.29
1207	3.24	4.27	5.98	6.97	6.42	5.73	5.03	4.35	3.75	3.11	2.49	0.7	0
1208	3.24	4.25	5.95	6.97	6.42	5.73	5.04	4.33	3.64	2.96	2.24	2.08	1.69
1209	3.24	4.26	5.97	6.97	6.42	5.73	5.05	4.36	3.78	3.14	2.48	1.89	1.65
1210	3.24	4.2	5.91	6.98	6.43	5.74	5.06	4.39	3.78	3.18	2.51	1.65	1.6
1211	3.24	4.23	5.94	6.98	6.42	5.74	5.05	4.35	3.77	3.19	2.6	2.05	1.71
1212	3.24	4.26	5.97	6.97	6.42	5.73	5.05	4.36	3.81	3.31	2.72	2.04	1.58
1213	3.24	4.26	5.96	6.97	6.42	5.73	5.05	4.35	3.79	3.16	2.5	2.1	1.57
1214	3.24	4.23	5.93	6.97	6.42	5.73	5.04	4.36	3.78	3.19	2.67	1.95	1.45
1215	3.24	4.28	5.98	6.97	6.42	5.73	5.05	4.33	3.73	3.19	2.59	1.87	0
1216	3.24	4.64	6.75	7	6.64	6.21	5.78	5.35	4.98	4.6	4.21	3.81	3.46
1217	3.24	4.61	6.72	7	6.64	6.21	5.78	5.36	4.97	4.6	4.2	3.82	3.44
1218	3.24	4.68	6.77	7	6.63	6.21	5.78	5.35	4.98	4.62	4.27	3.89	3.56
1219	3.24	4.62	6.73	7	6.64	6.21	5.78	5.36	4.98	4.62	4.23	3.86	3.45
1220	3.24	4.62	6.73	7	6.64	6.21	5.78	5.35	4.97	4.58	4.2	3.85	3.48
1221	3.24	4.67	6.77	7	6.64	6.21	5.78	5.35	4.98	4.6	4.22	3.85	3.47
1222	3.24	4.58	6.7	7	6.64	6.21	5.78	5.35	4.98	4.61	4.26	3.91	3.53
1223	3.24	4.62	6.73	7	6.64	6.21	5.78	5.35	4.98	4.59	4.22	3.86	3.52
1224	3.24	4.6	6.71	7	6.64	6.21	5.78	5.35	4.98	4.61	4.24	3.83	3.43
1225	3.24	4.21	5.94	7.02	6.69	6.26	5.84	5.41	5.03	4.65	4.28	3.93	3.55
1226	3.24	4.19	5.91	7.02	6.69	6.26	5.84	5.41	5.03	4.65	4.29	3.91	3.53
1227	3.24	4.26	5.97	7.02	6.69	6.26	5.83	5.41	5.03	4.66	4.28	3.92	3.55
1228	3.24	4.22	5.95	7.02	6.69	6.26	5.84	5.41	5.04	4.67	4.29	3.89	3.52
1229	3.24	4.24	5.95	7.02	6.69	6.26	5.83	5.4	5.03	4.66	4.29	3.9	3.53
1230	3.24	4.3	6.03	7.02	6.69	6.26	5.83	5.4	5.04	4.66	4.28	3.89	3.5
1231	3.24	4.28	5.98	7.02	6.69	6.26	5.83	5.4	5.03	4.66	4.29	3.91	3.54
1232	3.24	4.2	5.93	7.02	6.69	6.26	5.84	5.4	5.03	4.64	4.26	3.89	3.52
1233	3.24	4.21	5.95	7.02	6.69	6.26	5.83	5.41	5.05	4.67	4.28	3.93	3.58
