# Supplementary material for: Development of an Agent-Based Model (ABM) to Simulate the Immune System and Integration of a Regression Method to Estimate the Key ABM Parameters by Fitting the Experimental Data
Source: PLoS One. 2015 Nov 4;10(11):e0141295. doi: 10.1371/journal.pone.0141295 (PMC4633145; doi:10.1371/journal.pone.0141295)
Supplement: S9 Table — (PDF) [file pone.0141295.s010.pdf]

S9 Table. Output data sets of model 385x6

samples	output data value of ABM with 13 time points												
	1	2	3	4	5	6	7	8	9	10	11	12	13
1	3.24	4.29	6.02	7.02	6.69	6.26	5.83	5.4	5.02	4.64	4.26	3.89	3.53
2	3.24	4.33	6.05	7.02	6.68	6.26	5.83	5.41	5.04	4.66	4.27	3.91	3.53
3	3.24	4.26	5.99	7.02	6.69	6.26	5.83	5.4	5.04	4.67	4.28	3.93	3.52
4	3.24	4.23	5.95	7.02	6.69	6.26	5.83	5.41	5.02	4.65	4.28	3.9	3.51
5	3.24	4.24	5.97	7.02	6.69	6.26	5.83	5.41	5.03	4.65	4.27	3.89	3.5
6	3.24	4.31	6.03	7.02	6.69	6.26	5.83	5.4	5.02	4.66	4.31	3.93	3.6
7	3.24	3.72	4.74	6.24	6.86	6.43	6	5.57	5.2	4.82	4.45	4.07	3.7
8	3.24	3.77	4.83	6.33	6.85	6.42	5.99	5.56	5.18	4.81	4.44	4.08	3.68
9	3.24	3.7	4.73	6.24	6.86	6.43	6	5.57	5.21	4.83	4.45	4.07	3.69
10	3.24	3.69	4.72	6.23	6.86	6.43	6	5.57	5.2	4.84	4.47	4.11	3.76
11	3.24	3.65	4.69	6.2	6.86	6.43	6	5.57	5.19	4.81	4.42	4.04	3.7
12	3.24	3.7	4.75	6.25	6.86	6.43	6	5.57	5.19	4.82	4.45	4.06	3.67
13	3.24	4.29	6.02	7.07	6.95	6.77	6.59	6.41	6.26	6.1	5.95	5.79	5.63
14	3.24	4.23	5.97	7.07	6.95	6.77	6.59	6.42	6.26	6.1	5.95	5.79	5.64
15	3.24	4.24	5.98	7.07	6.95	6.77	6.59	6.42	6.26	6.11	5.95	5.8	5.64
16	3.24	4.25	6	7.07	6.95	6.77	6.59	6.41	6.26	6.1	5.95	5.79	5.63
17	3.24	4.31	6.05	7.07	6.95	6.77	6.59	6.41	6.26	6.1	5.94	5.79	5.63
18	3.24	4.3	6.04	7.07	6.95	6.77	6.59	6.41	6.26	6.1	5.95	5.79	5.63
19	3.24	4.49	6.31	7.27	7.12	6.72	6.3	5.87	5.5	5.12	4.75	4.41	4.05
20	3.24	4.46	6.27	7.27	7.12	6.72	6.29	5.86	5.49	5.11	4.72	4.34	3.97
21	3.24	4.5	6.32	7.27	7.12	6.72	6.3	5.87	5.5	5.12	4.74	4.39	4.03
22	3.24	4.48	6.29	7.27	7.12	6.72	6.29	5.86	5.49	5.11	4.73	4.35	3.98
23	3.24	4.49	6.31	7.27	7.12	6.72	6.3	5.87	5.5	5.12	4.75	4.37	3.99
24	3.24	4.53	6.35	7.27	7.12	6.72	6.29	5.86	5.48	5.11	4.74	4.34	3.97
25	3.24	4.2	5.92	7.02	6.69	6.26	5.83	5.41	5.04	4.65	4.26	3.87	3.54
26	3.24	4.29	6.01	7.02	6.68	6.25	5.83	5.4	5.02	4.65	4.29	3.92	3.5
27	3.24	4.22	5.94	7.02	6.69	6.26	5.83	5.4	5.03	4.66	4.29	3.92	3.56
28	3.24	4.24	5.95	7.02	6.69	6.26	5.83	5.41	5.04	4.67	4.29	3.88	3.53
29	3.24	4.29	6.01	7.02	6.69	6.26	5.84	5.41	5.02	4.65	4.27	3.87	3.48
30	3.24	4.3	6.03	7.02	6.69	6.26	5.83	5.4	5.02	4.64	4.27	3.91	3.54
31	3.24	3.91	5.54	6.86	6.49	6.06	5.64	5.21	4.84	4.46	4.09	3.7	3.29
32	3.24	3.91	5.55	6.86	6.49	6.06	5.64	5.2	4.83	4.45	4.06	3.72	3.35
33	3.24	3.89	5.51	6.86	6.5	6.06	5.64	5.21	4.84	4.47	4.1	3.72	3.32
34	3.24	3.94	5.55	6.86	6.49	6.06	5.64	5.21	4.84	4.46	4.09	3.73	3.37
35	3.24	3.97	5.59	6.86	6.49	6.06	5.63	5.21	4.83	4.45	4.08	3.68	3.32
36	3.24	3.99	5.61	6.86	6.49	6.06	5.63	5.2	4.82	4.44	4.07	3.68	3.33
37	3.24	4.23	5.94	6.97	6.42	5.74	5.05	4.34	3.76	3.17	2.54	2.13	1.26
38	3.24	4.17	5.87	6.98	6.43	5.74	5.05	4.37	3.78	3.18	2.57	1.64	0
39	3.24	4.27	5.97	6.97	6.42	5.73	5.04	4.36	3.72	3.12	2.58	2.1	0.6
40	3.24	4.29	6	6.97	6.42	5.73	5.05	4.39	3.77	3.24	2.45	1.36	1.32
41	3.24	4.26	5.96	6.97	6.42	5.74	5.05	4.33	3.74	3.17	2.53	1.83	0
42	3.24	4.17	5.87	6.98	6.43	5.74	5.05	4.34	3.74	3.06	2.36	1.75	1.41
43	3.24	4.66	6.75	7	6.64	6.21	5.78	5.35	4.97	4.6	4.21	3.81	3.46
44	3.24	4.63	6.73	7	6.64	6.21	5.78	5.35	4.97	4.6	4.23	3.88	3.52
45	3.24	4.63	6.73	7	6.64	6.21	5.78	5.35	4.97	4.6	4.25	3.86	3.49

46	3.24	4.61	6.72	7	6.64	6.21	5.78	5.35	4.98	4.61	4.24	3.86	3.53
47	3.24	4.66	6.76	7	6.64	6.21	5.78	5.35	4.97	4.62	4.23	3.85	3.5
48	3.24	4.67	6.76	7	6.64	6.21	5.78	5.35	4.98	4.61	4.23	3.83	3.41
49	3.24	3.53	4.26	5.26	6.95	6.64	6.21	5.78	5.41	5.04	4.66	4.3	3.94
50	3.24	3.32	4.01	4.99	6.89	6.68	6.25	5.82	5.44	5.07	4.69	4.31	3.93
51	3.24	3.41	4.1	5.09	6.92	6.67	6.24	5.81	5.44	5.06	4.68	4.31	3.92
52	3.24	3.44	4.15	5.14	6.93	6.66	6.23	5.8	5.43	5.05	4.68	4.31	3.93
53	3.24	3.39	4.11	5.1	6.92	6.66	6.23	5.81	5.44	5.07	4.7	4.34	3.95
54	3.24	3.29	3.98	4.96	6.88	6.69	6.26	5.83	5.46	5.08	4.69	4.31	3.93
55	3.24	3.69	4.74	6.29	7.02	6.84	6.66	6.48	6.33	6.17	6.02	5.86	5.71
56	3.24	3.71	4.77	6.31	7.02	6.84	6.66	6.48	6.33	6.17	6.01	5.86	5.7
57	3.24	3.67	4.71	6.26	7.02	6.84	6.66	6.48	6.33	6.17	6.01	5.86	5.7
58	3.24	3.63	4.7	6.25	7.02	6.84	6.66	6.49	6.33	6.17	6.02	5.87	5.71
59	3.24	3.77	4.86	6.38	7.01	6.83	6.66	6.48	6.32	6.17	6.01	5.85	5.7
60	3.24	3.68	4.74	6.29	7.02	6.84	6.66	6.48	6.33	6.17	6.01	5.86	5.7
61	3.24	3.96	5.1	6.75	7.22	6.85	6.43	6	5.62	5.24	4.86	4.47	4.11
62	3.24	3.86	5.03	6.69	7.23	6.86	6.44	6.01	5.64	5.26	4.87	4.51	4.11
63	3.24	3.97	5.15	6.79	7.22	6.85	6.42	6	5.62	5.24	4.88	4.5	4.15
64	3.24	3.96	5.14	6.78	7.22	6.85	6.42	6	5.62	5.24	4.86	4.49	4.14
65	3.24	3.94	5.11	6.75	7.22	6.85	6.43	6	5.62	5.25	4.87	4.51	4.15
66	3.24	3.89	5.07	6.73	7.23	6.86	6.43	6	5.63	5.25	4.88	4.51	4.12
67	3.24	3.64	4.69	6.2	6.86	6.43	6.01	5.58	5.2	4.83	4.47	4.1	3.76
68	3.24	3.68	4.71	6.22	6.86	6.43	6	5.57	5.2	4.82	4.46	4.08	3.71
69	3.24	3.65	4.66	6.18	6.86	6.44	6.01	5.58	5.2	4.82	4.45	4.07	3.69
70	3.24	3.7	4.74	6.23	6.86	6.43	6	5.57	5.19	4.81	4.42	4.06	3.71
71	3.24	3.68	4.74	6.24	6.86	6.43	6	5.57	5.2	4.83	4.45	4.06	3.71
72	3.24	3.69	4.7	6.21	6.86	6.43	6	5.58	5.21	4.83	4.44	4.07	3.73
73	3.24	3.42	4.34	5.67	6.71	6.28	5.85	5.42	5.05	4.67	4.27	3.89	3.5
74	3.24	3.33	4.29	5.64	6.71	6.28	5.86	5.43	5.05	4.67	4.3	3.91	3.5
75	3.24	3.41	4.34	5.67	6.71	6.28	5.85	5.42	5.05	4.68	4.31	3.9	3.54
76	3.24	3.37	4.27	5.62	6.71	6.29	5.85	5.43	5.05	4.67	4.3	3.94	3.57
77	3.24	3.31	4.2	5.54	6.72	6.3	5.87	5.44	5.06	4.69	4.32	3.95	3.51
78	3.24	3.31	4.24	5.58	6.72	6.29	5.86	5.44	5.06	4.68	4.29	3.92	3.48
79	3.24	3.58	4.6	6.08	6.72	6.04	5.35	4.68	4.1	3.46	2.87	2.25	1.48
80	3.24	3.57	4.59	6.08	6.72	6.04	5.35	4.66	4.06	3.48	2.84	2.33	1.49
81	3.24	3.62	4.67	6.13	6.71	6.03	5.34	4.65	4.06	3.44	2.84	2.21	0.9
82	3.24	3.69	4.72	6.18	6.7	6.02	5.32	4.65	4.02	3.36	2.72	2.19	1.41
83	3.24	3.71	4.71	6.18	6.7	6.02	5.33	4.66	4.04	3.5	2.76	2.08	1.38
84	3.24	3.67	4.68	6.16	6.71	6.02	5.33	4.63	4.05	3.44	2.75	2.17	1.83
85	3.24	4.32	6.07	7.09	7.04	6.94	6.85	6.75	6.67	6.59	6.51	6.42	6.34
86	3.24	4.25	6	7.09	7.04	6.94	6.85	6.76	6.67	6.59	6.51	6.43	6.34
87	3.24	4.21	5.95	7.09	7.04	6.94	6.85	6.75	6.67	6.59	6.51	6.42	6.34
88	3.24	4.24	5.98	7.09	7.04	6.94	6.85	6.75	6.67	6.59	6.51	6.42	6.34
89	3.24	4.26	6.01	7.09	7.04	6.94	6.85	6.75	6.67	6.59	6.51	6.42	6.34
90	3.24	4.25	6	7.09	7.04	6.94	6.85	6.75	6.67	6.59	6.5	6.42	6.34
91	3.24	4.49	6.33	7.31	7.34	7.18	7	6.82	6.67	6.51	6.35	6.2	6.04
92	3.24	4.46	6.29	7.31	7.34	7.18	7	6.82	6.67	6.51	6.35	6.2	6.04
93	3.24	4.51	6.34	7.32	7.34	7.18	7	6.82	6.66	6.51	6.35	6.2	6.04
94	3.24	4.5	6.33	7.32	7.34	7.18	7	6.82	6.67	6.51	6.35	6.2	6.04
95	3.24	4.52	6.35	7.32	7.34	7.17	7	6.82	6.66	6.51	6.35	6.2	6.04
96	3.24	4.49	6.32	7.31	7.34	7.18	7	6.82	6.66	6.51	6.35	6.2	6.04
97	3.24	4.19	5.94	7.07	6.95	6.77	6.59	6.42	6.26	6.1	5.95	5.79	5.63
98	3.24	4.23	5.98	7.07	6.95	6.77	6.59	6.42	6.26	6.11	5.95	5.8	5.64
99	3.24	4.3	6.04	7.07	6.95	6.77	6.59	6.41	6.26	6.1	5.95	5.79	5.63

100	3.24	4.24	5.98	7.07	6.95	6.77	6.59	6.42	6.26	6.11	5.95	5.8	5.64
101	3.24	4.2	5.95	7.07	6.95	6.77	6.6	6.42	6.26	6.11	5.95	5.79	5.64
102	3.24	4.25	5.99	7.07	6.95	6.77	6.59	6.42	6.26	6.1	5.95	5.79	5.64
103	3.24	3.95	5.58	6.9	6.75	6.57	6.4	6.22	6.06	5.91	5.75	5.6	5.44
104	3.24	3.98	5.63	6.9	6.75	6.57	6.4	6.22	6.06	5.91	5.75	5.59	5.44
105	3.24	4.04	5.67	6.9	6.75	6.57	6.4	6.22	6.06	5.91	5.75	5.6	5.44
106	3.24	3.97	5.62	6.9	6.75	6.57	6.4	6.22	6.06	5.91	5.75	5.59	5.44
107	3.24	3.94	5.58	6.9	6.75	6.57	6.4	6.22	6.06	5.91	5.75	5.59	5.44
108	3.24	3.96	5.62	6.9	6.75	6.57	6.4	6.22	6.06	5.91	5.75	5.6	5.44
109	3.24	4.56	6.41	7.37	7.4	7.12	6.75	6.35	5.99	5.63	5.26	4.88	4.51
110	3.24	4.56	6.41	7.37	7.4	7.12	6.75	6.35	5.99	5.62	5.25	4.88	4.48
111	3.24	4.55	6.4	7.37	7.4	7.12	6.75	6.36	6	5.63	5.27	4.9	4.54
112	3.24	4.54	6.39	7.37	7.4	7.12	6.75	6.35	5.99	5.62	5.25	4.89	4.52
113	3.24	4.54	6.4	7.37	7.4	7.12	6.75	6.36	6	5.64	5.27	4.91	4.54
114	3.24	4.48	6.33	7.36	7.41	7.12	6.76	6.36	6	5.63	5.26	4.88	4.49
115	3.24	4.47	6.3	7.27	7.12	6.72	6.29	5.87	5.5	5.12	4.75	4.38	4.03
116	3.24	4.45	6.27	7.27	7.12	6.72	6.3	5.87	5.49	5.12	4.75	4.36	3.99
117	3.24	4.46	6.28	7.27	7.12	6.72	6.29	5.86	5.49	5.11	4.75	4.4	4.05
118	3.24	4.5	6.31	7.27	7.12	6.72	6.3	5.87	5.49	5.12	4.74	4.36	3.96
119	3.24	4.48	6.29	7.27	7.12	6.72	6.3	5.87	5.49	5.12	4.75	4.37	4.01
120	3.24	4.45	6.27	7.27	7.12	6.72	6.3	5.87	5.49	5.11	4.74	4.38	4.04
121	3.24	4.2	5.92	7.02	6.69	6.27	5.84	5.41	5.03	4.66	4.29	3.92	3.51
122	3.24	4.21	5.93	7.02	6.69	6.26	5.83	5.41	5.03	4.65	4.27	3.9	3.56
123	3.24	4.23	5.95	7.02	6.69	6.26	5.84	5.41	5.04	4.66	4.3	3.92	3.55
124	3.24	4.28	6.01	7.02	6.69	6.26	5.84	5.4	5.03	4.66	4.3	3.94	3.55
125	3.24	4.18	5.91	7.02	6.69	6.26	5.83	5.41	5.03	4.66	4.28	3.88	3.52
126	3.24	4.31	6.03	7.02	6.68	6.26	5.83	5.4	5.04	4.66	4.29	3.89	3.55
127	3.24	3.95	5.57	6.86	6.49	6.06	5.63	5.2	4.83	4.45	4.06	3.7	3.31
128	3.24	3.94	5.57	6.86	6.49	6.06	5.63	5.2	4.83	4.47	4.08	3.68	3.29
129	3.24	3.82	5.44	6.86	6.5	6.07	5.64	5.22	4.84	4.47	4.11	3.72	3.34
130	3.24	3.98	5.61	6.86	6.49	6.06	5.63	5.21	4.83	4.45	4.09	3.73	3.34
131	3.24	3.89	5.49	6.86	6.5	6.07	5.64	5.22	4.84	4.48	4.11	3.75	3.4
132	3.24	3.94	5.56	6.86	6.49	6.06	5.64	5.21	4.83	4.45	4.09	3.71	3.35
133	3.24	3.78	5.37	6.82	6.45	6.03	5.6	5.18	4.79	4.43	4.05	3.68	3.33
134	3.24	3.86	5.45	6.81	6.45	6.02	5.59	5.16	4.79	4.42	4.04	3.66	3.3
135	3.24	3.83	5.43	6.81	6.45	6.02	5.59	5.16	4.78	4.41	4.03	3.65	3.28
136	3.24	3.86	5.46	6.81	6.45	6.02	5.59	5.17	4.79	4.41	4.04	3.64	3.24
137	3.24	3.85	5.44	6.81	6.45	6.02	5.59	5.16	4.78	4.4	4.02	3.62	3.24
138	3.24	3.86	5.41	6.82	6.45	6.02	5.59	5.16	4.78	4.39	4.01	3.63	3.27
139	3.24	4.46	6.27	7.22	6.91	6.28	5.6	4.9	4.28	3.67	3.06	2.47	2.09
140	3.24	4.44	6.24	7.22	6.92	6.28	5.61	4.91	4.31	3.71	3.12	2.64	1.85
141	3.24	4.41	6.22	7.22	6.92	6.28	5.6	4.91	4.32	3.71	3.09	2.12	0.95
142	3.24	4.47	6.27	7.22	6.91	6.28	5.6	4.9	4.3	3.74	3.17	2.52	2.13
143	3.24	4.45	6.25	7.22	6.91	6.28	5.59	4.91	4.3	3.7	3.1	2.58	1.53
144	3.24	4.51	6.31	7.22	6.91	6.28	5.6	4.91	4.3	3.66	2.98	2.05	0.85
145	3.24	4.19	5.9	6.98	6.43	5.74	5.05	4.37	3.78	3.2	2.69	2.12	1.34
146	3.24	4.25	5.96	6.97	6.42	5.74	5.05	4.35	3.74	3.15	2.48	2	0
147	3.24	4.23	5.94	6.97	6.42	5.74	5.05	4.36	3.79	3.2	2.65	2.1	1.56
148	3.24	4.27	5.97	6.97	6.42	5.73	5.04	4.38	3.78	3.14	2.46	1.67	1.34
149	3.24	4.26	5.96	6.97	6.42	5.74	5.05	4.37	3.78	3.19	2.67	2.07	1.76
150	3.24	4.21	5.92	6.97	6.42	5.74	5.05	4.36	3.77	3.18	2.57	1.61	0
151	3.24	3.95	5.56	6.82	6.22	5.54	4.84	4.15	3.49	2.8	2.34	1.85	1.15
152	3.24	3.97	5.58	6.81	6.22	5.54	4.84	4.16	3.53	2.91	2.35	1.91	1.46
153	3.24	3.92	5.52	6.82	6.23	5.54	4.86	4.17	3.53	2.88	2.22	1.95	1.68

154	3.24	3.91	5.52	6.82	6.23	5.54	4.86	4.18	3.61	3.01	2.36	1.46	0.7
155	3.24	3.94	5.55	6.82	6.23	5.54	4.86	4.2	3.58	2.9	2.29	1.85	1.3
156	3.24	3.99	5.59	6.81	6.22	5.53	4.85	4.16	3.53	2.92	2.17	1.84	0
157	3.24	4.26	5.95	6.96	6.33	5.55	4.78	4.01	3.36	2.72	2.29	1.41	0
158	3.24	4.27	5.97	6.96	6.33	5.55	4.77	3.97	3.3	2.73	2.2	1.53	1.38
159	3.24	4.22	5.93	6.96	6.33	5.55	4.77	3.97	3.33	2.72	1.97	1.71	0.48
160	3.24	4.26	5.96	6.96	6.33	5.55	4.77	3.98	3.31	2.64	2.31	1.43	0
161	3.24	4.19	5.9	6.96	6.34	5.56	4.77	3.98	3.25	2.51	2.2	1.36	0
162	3.24	4.22	5.91	6.96	6.34	5.55	4.78	4.03	3.34	2.74	2.18	0	0
163	3.24	4.67	6.78	7.07	6.93	6.75	6.57	6.39	6.24	6.08	5.93	5.77	5.61
164	3.24	4.68	6.78	7.07	6.93	6.75	6.57	6.39	6.24	6.09	5.93	5.78	5.62
165	3.24	4.52	6.67	7.07	6.93	6.75	6.58	6.4	6.24	6.09	5.93	5.78	5.62
166	3.24	4.65	6.77	7.07	6.93	6.75	6.57	6.39	6.24	6.08	5.92	5.77	5.61
167	3.24	4.63	6.75	7.07	6.93	6.75	6.57	6.39	6.24	6.08	5.92	5.77	5.61
168	3.24	4.71	6.8	7.07	6.93	6.75	6.57	6.39	6.23	6.08	5.92	5.77	5.61
169	3.24	4.83	6.92	7.29	7.09	6.68	6.25	5.82	5.44	5.06	4.7	4.36	3.96
170	3.24	4.81	6.91	7.29	7.09	6.68	6.25	5.82	5.45	5.08	4.71	4.37	3.99
171	3.24	4.81	6.91	7.29	7.09	6.68	6.25	5.82	5.44	5.06	4.69	4.32	3.94
172	3.24	4.82	6.91	7.29	7.09	6.68	6.26	5.83	5.46	5.09	4.72	4.32	3.93
173	3.24	4.82	6.92	7.29	7.09	6.68	6.25	5.83	5.45	5.07	4.71	4.33	3.95
174	3.24	4.85	6.92	7.29	7.08	6.68	6.25	5.82	5.46	5.08	4.7	4.34	3.97
175	3.24	4.6	6.71	7	6.64	6.21	5.78	5.35	4.98	4.6	4.23	3.86	3.51
176	3.24	4.61	6.72	7	6.64	6.21	5.79	5.36	4.99	4.62	4.25	3.87	3.54
177	3.24	4.61	6.72	7	6.64	6.21	5.78	5.36	4.98	4.61	4.22	3.85	3.46
178	3.24	4.6	6.71	7	6.64	6.21	5.78	5.35	4.98	4.6	4.23	3.84	3.41
179	3.24	4.68	6.77	7	6.64	6.21	5.77	5.35	4.97	4.6	4.2	3.83	3.48
180	3.24	4.64	6.74	7	6.64	6.21	5.78	5.36	4.98	4.6	4.22	3.86	3.46
181	3.24	4.3	6.38	6.81	6.43	6	5.57	5.14	4.77	4.39	4.01	3.65	3.32
182	3.24	4.31	6.38	6.81	6.43	6	5.57	5.14	4.77	4.38	4.03	3.66	3.26
183	3.24	4.25	6.33	6.81	6.44	6.01	5.58	5.15	4.77	4.4	4.01	3.64	3.27
184	3.24	4.38	6.44	6.8	6.43	6	5.57	5.15	4.77	4.41	4.03	3.65	3.3
185	3.24	4.3	6.37	6.81	6.43	6	5.58	5.14	4.76	4.37	4.02	3.64	3.28
186	3.24	4.28	6.36	6.81	6.43	6	5.58	5.14	4.78	4.41	4.03	3.66	3.29
187	3.24	4.61	6.7	6.92	6.34	5.64	4.95	4.26	3.7	3.13	2.42	1.79	0.48
188	3.24	4.59	6.69	6.93	6.34	5.66	4.96	4.27	3.67	3.15	2.51	1.84	1.4
189	3.24	4.63	6.72	6.92	6.34	5.65	4.97	4.26	3.69	3.12	2.43	2.2	1.9
190	3.24	4.63	6.72	6.92	6.34	5.64	4.95	4.25	3.65	3.02	2.44	1.56	0
191	3.24	4.6	6.7	6.92	6.34	5.66	4.97	4.28	3.68	3.07	2.52	1.62	0
192	3.24	4.63	6.73	6.92	6.34	5.65	4.97	4.3	3.7	3.08	2.54	1.92	1.77
193	3.24	4.71	6.85	6.99	6.63	6.2	5.77	5.34	4.96	4.58	4.2	3.83	3.45
194	3.24	4.7	6.85	6.99	6.63	6.2	5.77	5.34	4.96	4.6	4.22	3.84	3.49
195	3.24	4.74	6.86	6.99	6.63	6.19	5.76	5.33	4.95	4.57	4.2	3.84	3.48
196	3.24	4.73	6.86	6.99	6.63	6.2	5.77	5.34	4.96	4.58	4.21	3.84	3.43
197	3.24	4.74	6.86	6.99	6.63	6.2	5.77	5.34	4.97	4.6	4.22	3.85	3.51
198	3.24	4.76	6.86	6.99	6.62	6.19	5.77	5.34	4.96	4.6	4.22	3.86	3.48
199	3.24	3.19	3.7	4.35	5.92	6.89	6.57	6.15	5.77	5.4	5.02	4.65	4.28
200	3.24	3.29	3.78	4.48	6	6.89	6.55	6.12	5.75	5.37	5.01	4.63	4.26
201	3.24	3.27	3.82	4.51	6.03	6.89	6.54	6.12	5.75	5.37	4.99	4.62	4.26
202	3.24	3.28	3.79	4.52	6.04	6.89	6.54	6.12	5.74	5.37	4.99	4.62	4.23
203	3.24	3.24	3.77	4.44	5.98	6.89	6.55	6.13	5.76	5.38	5.01	4.63	4.28
204	3.24	3.13	3.68	4.37	5.92	6.89	6.57	6.15	5.77	5.4	5.02	4.65	4.27
205	3.24	3.26	3.99	5.04	6.99	6.94	6.76	6.58	6.42	6.27	6.11	5.96	5.8
206	3.24	3.47	4.16	5.21	7.02	6.93	6.75	6.57	6.41	6.26	6.1	5.94	5.79
207	3.24	3.33	4.05	5.09	7	6.93	6.76	6.58	6.42	6.27	6.11	5.95	5.8

208	3.24	3.48	4.2	5.24	7.03	6.92	6.75	6.57	6.41	6.26	6.1	5.95	5.79
209	3.24	3.46	4.21	5.26	7.03	6.92	6.74	6.57	6.41	6.25	6.1	5.95	5.79
210	3.24	3.36	4.12	5.17	7.01	6.93	6.75	6.57	6.42	6.26	6.11	5.95	5.79
211	3.24	3.72	4.54	5.75	7.28	7	6.59	6.16	5.79	5.41	5.04	4.67	4.28
212	3.24	3.65	4.47	5.67	7.28	7.01	6.6	6.17	5.79	5.41	5.03	4.64	4.25
213	3.24	3.65	4.48	5.7	7.28	7.01	6.59	6.17	5.79	5.42	5.06	4.68	4.31
214	3.24	3.65	4.45	5.66	7.28	7.01	6.6	6.17	5.79	5.41	5.03	4.66	4.29
215	3.24	3.64	4.45	5.65	7.28	7.01	6.6	6.18	5.8	5.43	5.06	4.67	4.3
216	3.24	3.69	4.53	5.73	7.28	7	6.59	6.16	5.78	5.41	5.04	4.66	4.29
217	3.24	3.4	4.08	5.08	6.91	6.67	6.24	5.81	5.45	5.08	4.7	4.32	3.92
218	3.24	3.33	4	5.01	6.9	6.68	6.25	5.82	5.45	5.08	4.72	4.34	3.96
219	3.24	3.36	4.06	5.06	6.91	6.67	6.25	5.82	5.43	5.07	4.69	4.31	3.91
220	3.24	3.38	4.06	5.06	6.91	6.67	6.24	5.81	5.43	5.06	4.69	4.32	3.94
221	3.24	3.42	4.1	5.09	6.92	6.67	6.24	5.81	5.43	5.06	4.7	4.32	3.94
222	3.24	3.37	4.02	5.01	6.9	6.68	6.25	5.82	5.45	5.08	4.69	4.31	3.95
223	3.24	3.16	3.74	4.51	6.26	6.62	6.2	5.77	5.4	5.02	4.64	4.27	3.86
224	3.24	2.99	3.62	4.45	6.24	6.62	6.21	5.78	5.41	5.03	4.65	4.28	3.9
225	3.24	3.09	3.63	4.49	6.27	6.61	6.2	5.77	5.39	5.02	4.65	4.27	3.9
226	3.24	3.06	3.68	4.53	6.28	6.61	6.19	5.77	5.4	5.02	4.66	4.27	3.9
227	3.24	3.1	3.67	4.47	6.25	6.62	6.2	5.78	5.4	5.03	4.65	4.28	3.88
228	3.24	3.15	3.81	4.63	6.36	6.59	6.17	5.75	5.37	5	4.62	4.26	3.9
229	3.24	3.32	4.01	4.95	6.79	6.46	5.77	5.07	4.47	3.85	3.23	2.79	2.26
230	3.24	3.34	4	4.96	6.79	6.46	5.78	5.09	4.49	3.9	3.29	2.71	2.25
231	3.24	3.35	4	4.96	6.79	6.46	5.78	5.11	4.52	3.91	3.35	2.74	2
232	3.24	3.36	4.01	4.97	6.8	6.45	5.77	5.07	4.47	3.88	3.18	2.56	2.01
233	3.24	3.39	4.04	5	6.81	6.44	5.76	5.07	4.47	3.88	3.26	2.55	1.84
234	3.24	3.36	4.06	5	6.81	6.44	5.76	5.08	4.48	3.86	3.28	2.63	2
235	3.24	3.73	4.79	6.34	7.07	6.98	6.88	6.79	6.71	6.62	6.54	6.46	6.38
236	3.24	3.72	4.81	6.36	7.07	6.98	6.88	6.79	6.7	6.62	6.54	6.46	6.37
237	3.24	3.67	4.76	6.32	7.07	6.98	6.88	6.79	6.71	6.62	6.54	6.46	6.38
238	3.24	3.64	4.7	6.26	7.07	6.98	6.89	6.79	6.71	6.62	6.54	6.46	6.38
239	3.24	3.61	4.69	6.25	7.07	6.98	6.89	6.79	6.71	6.63	6.54	6.46	6.38
240	3.24	3.68	4.77	6.32	7.07	6.98	6.88	6.79	6.71	6.62	6.54	6.46	6.38
241	3.24	3.94	5.13	6.8	7.37	7.23	7.05	6.87	6.72	6.56	6.41	6.25	6.1
242	3.24	3.95	5.15	6.82	7.37	7.23	7.05	6.87	6.72	6.56	6.4	6.25	6.09
243	3.24	3.99	5.18	6.84	7.37	7.23	7.05	6.87	6.72	6.56	6.4	6.25	6.09
244	3.24	3.96	5.15	6.81	7.37	7.23	7.05	6.87	6.72	6.56	6.41	6.25	6.09
245	3.24	3.9	5.09	6.77	7.37	7.23	7.05	6.88	6.72	6.56	6.41	6.25	6.1
246	3.24	3.94	5.12	6.79	7.37	7.23	7.05	6.88	6.72	6.56	6.41	6.25	6.1
247	3.24	3.71	4.78	6.31	7.02	6.84	6.66	6.48	6.32	6.17	6.01	5.86	5.7
248	3.24	3.7	4.78	6.33	7.01	6.84	6.66	6.48	6.32	6.17	6.01	5.86	5.7
249	3.24	3.69	4.76	6.3	7.02	6.84	6.66	6.48	6.33	6.17	6.01	5.86	5.7
250	3.24	3.72	4.78	6.32	7.02	6.84	6.66	6.48	6.33	6.17	6.01	5.86	5.71
251	3.24	3.67	4.75	6.29	7.02	6.84	6.66	6.48	6.33	6.17	6.02	5.86	5.7
252	3.24	3.61	4.64	6.19	7.02	6.84	6.66	6.49	6.33	6.17	6.02	5.86	5.7
253	3.24	3.38	4.29	5.68	6.84	6.66	6.48	6.31	6.15	6	5.84	5.68	5.53
254	3.24	3.45	4.42	5.8	6.83	6.66	6.48	6.3	6.15	5.99	5.83	5.68	5.52
255	3.24	3.29	4.26	5.64	6.84	6.66	6.49	6.31	6.15	6	5.84	5.69	5.53
256	3.24	3.44	4.36	5.73	6.84	6.66	6.48	6.3	6.15	5.99	5.84	5.68	5.52
257	3.24	3.52	4.48	5.87	6.83	6.65	6.47	6.29	6.14	5.98	5.83	5.67	5.51
258	3.24	3.4	4.36	5.75	6.84	6.66	6.48	6.3	6.15	5.99	5.83	5.68	5.52
259	3.24	3.95	5.17	6.87	7.45	7.22	6.87	6.48	6.12	5.75	5.38	5	4.62
260	3.24	3.98	5.21	6.9	7.45	7.21	6.86	6.47	6.11	5.74	5.38	5.01	4.64
261	3.24	3.98	5.2	6.89	7.45	7.22	6.86	6.47	6.11	5.75	5.39	5.02	4.65

262	3.24	4.03	5.26	6.93	7.45	7.21	6.86	6.47	6.11	5.75	5.38	5.01	4.62
263	3.24	4.07	5.28	6.95	7.45	7.21	6.86	6.47	6.11	5.75	5.39	5.02	4.67
264	3.24	4.06	5.27	6.94	7.45	7.21	6.86	6.47	6.11	5.74	5.36	4.99	4.65
265	3.24	3.93	5.1	6.75	7.22	6.85	6.43	6	5.63	5.26	4.89	4.52	4.16
266	3.24	3.91	5.08	6.73	7.22	6.85	6.43	6.01	5.64	5.26	4.89	4.5	4.11
267	3.24	3.9	5.08	6.73	7.22	6.85	6.43	6.01	5.63	5.24	4.87	4.48	4.09
268	3.24	3.97	5.16	6.8	7.22	6.85	6.42	6	5.62	5.25	4.86	4.48	4.1
269	3.24	3.93	5.1	6.75	7.22	6.85	6.43	6	5.63	5.27	4.89	4.52	4.13
270	3.24	3.9	5.08	6.73	7.23	6.86	6.43	6.01	5.63	5.25	4.88	4.51	4.14
271	3.24	3.71	4.75	6.25	6.86	6.43	6	5.57	5.19	4.83	4.45	4.09	3.69
272	3.24	3.55	4.62	6.14	6.87	6.44	6.01	5.58	5.2	4.83	4.45	4.1	3.75
273	3.24	3.64	4.67	6.19	6.86	6.43	6	5.57	5.2	4.83	4.46	4.1	3.74
274	3.24	3.81	4.84	6.33	6.85	6.42	5.99	5.56	5.18	4.81	4.45	4.06	3.69
275	3.24	3.68	4.72	6.23	6.86	6.43	6	5.58	5.2	4.83	4.46	4.11	3.75
276	3.24	3.58	4.61	6.13	6.87	6.44	6.01	5.59	5.21	4.84	4.46	4.08	3.68
277	3.24	3.44	4.37	5.7	6.7	6.28	5.85	5.42	5.05	4.68	4.3	3.94	3.54
278	3.24	3.26	4.19	5.54	6.72	6.3	5.87	5.44	5.06	4.69	4.3	3.93	3.56
279	3.24	3.41	4.3	5.64	6.71	6.28	5.86	5.42	5.04	4.68	4.32	3.93	3.54
280	3.24	3.48	4.4	5.73	6.7	6.27	5.84	5.41	5.04	4.67	4.28	3.89	3.51
281	3.24	3.35	4.22	5.58	6.72	6.29	5.86	5.43	5.05	4.68	4.31	3.9	3.55
282	3.24	3.39	4.32	5.66	6.71	6.28	5.85	5.42	5.05	4.67	4.3	3.92	3.54
283	3.24	3.19	4.05	5.33	6.7	6.27	5.84	5.42	5.04	4.67	4.3	3.93	3.52
284	3.24	3.41	4.29	5.57	6.67	6.24	5.81	5.38	5.01	4.64	4.25	3.88	3.53
285	3.24	3.31	4.21	5.5	6.68	6.25	5.82	5.39	5.02	4.64	4.28	3.92	3.54
286	3.24	3.43	4.28	5.54	6.67	6.25	5.82	5.39	5.02	4.65	4.27	3.91	3.57
287	3.24	3.15	4.05	5.35	6.7	6.27	5.84	5.41	5.04	4.66	4.29	3.94	3.55
288	3.24	3.31	4.22	5.51	6.68	6.25	5.82	5.39	5.02	4.65	4.28	3.93	3.54
289	3.24	3.95	5.1	6.72	7.08	6.49	5.82	5.14	4.55	3.99	3.37	2.58	1.88
290	3.24	3.91	5.05	6.68	7.08	6.49	5.82	5.15	4.54	3.91	3.37	2.66	1.97
291	3.24	3.93	5.1	6.72	7.08	6.49	5.81	5.12	4.51	3.89	3.31	2.71	1.93
292	3.24	3.91	5.05	6.67	7.08	6.49	5.83	5.15	4.56	3.93	3.26	2.51	1.68
293	3.24	3.92	5.07	6.7	7.08	6.49	5.82	5.14	4.53	3.9	3.28	2.75	2.36
294	3.24	3.93	5.1	6.72	7.08	6.49	5.81	5.12	4.51	3.89	3.29	2.68	1.95
295	3.24	3.72	4.73	6.21	6.7	6.01	5.32	4.63	4.03	3.48	2.98	2.38	1.89
296	3.24	3.68	4.69	6.16	6.71	6.02	5.33	4.64	4.05	3.46	2.99	2.42	0
297	3.24	3.64	4.64	6.11	6.72	6.03	5.34	4.67	4.08	3.44	2.86	2.38	1.75
298	3.24	3.65	4.68	6.14	6.71	6.03	5.34	4.65	4.07	3.41	2.75	2.14	0
299	3.24	3.65	4.65	6.11	6.72	6.03	5.35	4.65	4.05	3.44	2.87	2.39	1.84
300	3.24	3.67	4.68	6.15	6.71	6.02	5.34	4.67	4.08	3.49	2.9	2.41	2.03
301	3.24	3.41	4.3	5.59	6.6	5.91	5.22	4.52	3.9	3.28	2.68	2.01	1.49
302	3.24	3.51	4.39	5.67	6.58	5.89	5.2	4.51	3.91	3.31	2.72	1.95	1.65
303	3.24	3.33	4.24	5.52	6.61	5.92	5.24	4.54	3.94	3.33	2.7	2.37	1.64
304	3.24	3.39	4.28	5.57	6.6	5.91	5.22	4.53	3.93	3.35	2.74	1.94	1.15
305	3.24	3.28	4.13	5.42	6.63	5.94	5.25	4.57	3.99	3.41	2.72	2.15	1.51
306	3.24	3.38	4.3	5.59	6.6	5.91	5.22	4.53	3.91	3.33	2.67	2	1.79
307	3.24	3.61	4.62	6.08	6.67	5.89	5.11	4.34	3.7	3	2.44	1.94	1.38
308	3.24	3.6	4.58	6.03	6.68	5.91	5.13	4.35	3.74	2.98	2.3	1.81	0.9
309	3.24	3.66	4.68	6.12	6.66	5.89	5.1	4.32	3.67	2.99	2.36	1.83	0
310	3.24	3.66	4.69	6.14	6.66	5.88	5.09	4.31	3.67	3.04	2.43	1.85	0
311	3.24	3.61	4.64	6.1	6.67	5.89	5.11	4.33	3.6	2.93	2.16	1.69	0
312	3.24	3.66	4.65	6.12	6.66	5.89	5.11	4.33	3.66	3.03	2.33	1.89	1.26
313	3.24	3.88	5.24	6.94	6.76	6.33	5.9	5.48	5.1	4.74	4.36	3.98	3.58
314	3.24	3.94	5.32	6.96	6.75	6.33	5.9	5.47	5.09	4.72	4.35	4	3.6
315	3.24	3.98	5.34	6.96	6.75	6.33	5.9	5.47	5.09	4.72	4.36	3.99	3.63

316	3.24	3.9	5.27	6.94	6.76	6.33	5.9	5.47	5.1	4.72	4.34	3.96	3.6
317	3.24	4.01	5.37	6.97	6.75	6.32	5.89	5.47	5.09	4.71	4.32	3.93	3.57
318	3.24	3.92	5.29	6.95	6.76	6.33	5.9	5.47	5.1	4.72	4.34	3.97	3.59
319	3.24	4.26	6.01	7.1	7.08	7.02	6.96	6.9	6.85	6.8	6.75	6.7	6.65
320	3.24	4.28	6.02	7.1	7.08	7.02	6.96	6.9	6.85	6.8	6.75	6.7	6.65
321	3.24	4.23	5.98	7.1	7.08	7.02	6.96	6.9	6.85	6.8	6.75	6.7	6.65
322	3.24	4.24	5.99	7.1	7.08	7.02	6.96	6.9	6.85	6.8	6.75	6.7	6.65
323	3.24	4.27	6.01	7.1	7.08	7.02	6.96	6.9	6.85	6.8	6.75	6.7	6.65
324	3.24	4.26	6.01	7.1	7.08	7.02	6.96	6.9	6.85	6.8	6.75	6.7	6.65
325	3.24	4.46	6.3	7.33	7.42	7.33	7.24	7.14	7.06	6.98	6.9	6.81	6.73
326	3.24	4.52	6.35	7.33	7.42	7.33	7.24	7.14	7.06	6.98	6.9	6.81	6.73
327	3.24	4.43	6.27	7.33	7.42	7.33	7.24	7.14	7.06	6.98	6.9	6.81	6.73
328	3.24	4.47	6.32	7.33	7.42	7.33	7.24	7.14	7.06	6.98	6.9	6.81	6.73
329	3.24	4.5	6.34	7.33	7.42	7.33	7.24	7.14	7.06	6.98	6.9	6.81	6.73
330	3.24	4.5	6.34	7.33	7.42	7.33	7.24	7.14	7.06	6.98	6.9	6.81	6.73
331	3.24	4.28	6.02	7.09	7.04	6.94	6.85	6.76	6.67	6.59	6.51	6.42	6.34
332	3.24	4.3	6.04	7.09	7.04	6.94	6.85	6.75	6.67	6.59	6.5	6.42	6.34
333	3.24	4.23	5.98	7.09	7.04	6.94	6.85	6.75	6.67	6.59	6.51	6.42	6.34
334	3.24	4.28	6.03	7.09	7.04	6.94	6.85	6.75	6.67	6.59	6.51	6.42	6.34
335	3.24	4.3	6.04	7.09	7.04	6.94	6.85	6.75	6.67	6.59	6.51	6.42	6.34
336	3.24	4.28	6.02	7.09	7.04	6.94	6.85	6.75	6.67	6.59	6.51	6.42	6.34
337	3.24	3.99	5.63	6.92	6.84	6.75	6.65	6.56	6.47	6.39	6.31	6.23	6.14
338	3.24	3.96	5.61	6.92	6.84	6.75	6.65	6.56	6.48	6.39	6.31	6.23	6.15
339	3.24	4.01	5.67	6.92	6.84	6.74	6.65	6.56	6.47	6.39	6.31	6.23	6.14
340	3.24	3.93	5.59	6.92	6.84	6.75	6.65	6.56	6.48	6.39	6.31	6.23	6.15
341	3.24	3.92	5.58	6.92	6.84	6.75	6.65	6.56	6.47	6.39	6.31	6.23	6.14
342	3.24	3.95	5.59	6.92	6.84	6.75	6.65	6.56	6.47	6.39	6.31	6.23	6.14
343	3.24	4.55	6.42	7.42	7.58	7.49	7.33	7.16	7	6.85	6.69	6.54	6.38
344	3.24	4.5	6.37	7.41	7.58	7.49	7.33	7.16	7	6.85	6.69	6.54	6.38
345	3.24	4.53	6.4	7.41	7.58	7.48	7.33	7.16	7	6.85	6.69	6.54	6.38
346	3.24	4.56	6.43	7.42	7.58	7.48	7.33	7.15	7	6.84	6.69	6.53	6.38
347	3.24	4.54	6.41	7.41	7.58	7.49	7.33	7.16	7	6.85	6.69	6.54	6.38
348	3.24	4.53	6.39	7.41	7.58	7.49	7.33	7.16	7	6.85	6.69	6.54	6.38
349	3.24	4.48	6.32	7.31	7.34	7.17	7	6.82	6.66	6.51	6.35	6.19	6.04
350	3.24	4.47	6.31	7.31	7.34	7.18	7	6.82	6.67	6.51	6.35	6.2	6.04
351	3.24	4.47	6.31	7.31	7.34	7.18	7	6.82	6.66	6.51	6.35	6.2	6.04
352	3.24	4.52	6.35	7.32	7.34	7.17	7	6.82	6.66	6.51	6.35	6.2	6.04
353	3.24	4.51	6.34	7.32	7.34	7.18	7	6.82	6.66	6.51	6.35	6.19	6.04
354	3.24	4.5	6.34	7.32	7.34	7.18	7	6.82	6.67	6.51	6.36	6.2	6.04
355	3.24	4.22	5.96	7.07	6.95	6.77	6.59	6.42	6.26	6.11	5.95	5.8	5.64
356	3.24	4.24	5.98	7.07	6.95	6.77	6.59	6.42	6.26	6.11	5.95	5.79	5.63
357	3.24	4.22	5.95	7.07	6.95	6.77	6.6	6.42	6.26	6.11	5.95	5.79	5.64
358	3.24	4.26	5.99	7.07	6.95	6.77	6.59	6.41	6.26	6.11	5.95	5.79	5.64
359	3.24	4.26	6	7.07	6.95	6.77	6.59	6.41	6.26	6.1	5.95	5.79	5.64
360	3.24	4.26	6	7.07	6.95	6.77	6.59	6.42	6.26	6.1	5.95	5.79	5.64
361	3.24	4.01	5.65	6.9	6.75	6.57	6.4	6.22	6.06	5.91	5.75	5.6	5.44
362	3.24	4	5.65	6.9	6.75	6.57	6.4	6.22	6.06	5.91	5.75	5.59	5.44
363	3.24	4.02	5.66	6.9	6.75	6.57	6.4	6.22	6.06	5.91	5.75	5.6	5.44
364	3.24	3.94	5.59	6.9	6.75	6.57	6.4	6.22	6.06	5.91	5.75	5.6	5.44
365	3.24	3.96	5.59	6.9	6.75	6.57	6.39	6.22	6.06	5.91	5.75	5.6	5.44
366	3.24	4.03	5.68	6.9	6.75	6.57	6.4	6.22	6.06	5.91	5.75	5.6	5.44
367	3.24	3.84	5.46	6.85	6.7	6.52	6.35	6.17	6.01	5.86	5.7	5.54	5.38
368	3.24	3.95	5.55	6.85	6.7	6.52	6.35	6.17	6.01	5.86	5.7	5.55	5.39
369	3.24	4.02	5.61	6.85	6.7	6.52	6.34	6.17	6.01	5.85	5.7	5.54	5.38

370	3.24	3.86	5.47	6.85	6.7	6.53	6.35	6.17	6.01	5.86	5.7	5.54	5.39
371	3.24	3.86	5.47	6.85	6.7	6.52	6.35	6.17	6.01	5.85	5.7	5.54	5.38
372	3.24	3.94	5.53	6.85	6.7	6.52	6.35	6.17	6.01	5.85	5.7	5.54	5.38
373	3.24	4.24	5.96	7.05	6.84	6.56	6.28	6	5.76	5.51	5.26	5.02	4.77
374	3.24	4.24	5.97	7.05	6.84	6.56	6.28	6	5.76	5.52	5.27	5.02	4.78
375	3.24	4.27	6	7.05	6.84	6.56	6.28	6	5.76	5.51	5.27	5.03	4.77
376	3.24	4.27	5.99	7.05	6.84	6.56	6.28	6	5.76	5.51	5.27	5.03	4.78
377	3.24	4.28	6.01	7.05	6.84	6.56	6.28	6	5.76	5.51	5.26	5.02	4.78
378	3.24	4.23	5.97	7.05	6.84	6.56	6.28	6	5.76	5.51	5.27	5.03	4.78
379	3.24	4.56	6.42	7.42	7.56	7.39	7.12	6.81	6.52	6.22	5.91	5.59	5.27
380	3.24	4.61	6.47	7.42	7.56	7.39	7.12	6.81	6.52	6.21	5.9	5.58	5.25
381	3.24	4.58	6.44	7.42	7.56	7.39	7.12	6.81	6.52	6.22	5.91	5.59	5.27
382	3.24	4.58	6.44	7.42	7.56	7.39	7.12	6.81	6.52	6.22	5.91	5.59	5.26
383	3.24	4.62	6.47	7.42	7.56	7.39	7.12	6.81	6.52	6.21	5.9	5.58	5.25
384	3.24	4.55	6.42	7.41	7.56	7.39	7.12	6.81	6.52	6.22	5.91	5.59	5.27
385	3.24	4.49	6.35	7.37	7.41	7.12	6.75	6.35	5.99	5.62	5.24	4.87	4.49
386	3.24	4.57	6.42	7.37	7.4	7.12	6.75	6.35	5.99	5.62	5.25	4.88	4.49
387	3.24	4.53	6.37	7.37	7.4	7.12	6.75	6.36	6	5.63	5.25	4.88	4.5
388	3.24	4.55	6.39	7.37	7.4	7.12	6.75	6.35	5.99	5.62	5.25	4.88	4.51
389	3.24	4.52	6.37	7.37	7.41	7.12	6.75	6.35	5.99	5.62	5.25	4.88	4.49
390	3.24	4.54	6.39	7.37	7.4	7.12	6.75	6.35	5.99	5.62	5.25	4.88	4.49
391	3.24	4.5	6.32	7.27	7.12	6.72	6.3	5.87	5.5	5.13	4.75	4.38	3.97
392	3.24	4.48	6.29	7.27	7.12	6.72	6.3	5.87	5.5	5.12	4.74	4.37	4.01
393	3.24	4.48	6.31	7.27	7.12	6.72	6.29	5.87	5.49	5.12	4.74	4.37	4.01
394	3.24	4.5	6.32	7.27	7.12	6.72	6.3	5.87	5.5	5.13	4.75	4.35	3.98
395	3.24	4.48	6.29	7.27	7.12	6.72	6.29	5.86	5.49	5.12	4.72	4.34	3.96
396	3.24	4.54	6.35	7.27	7.12	6.72	6.29	5.86	5.49	5.11	4.73	4.37	3.96
397	3.24	4.41	6.18	7.16	6.89	6.46	6.04	5.61	5.23	4.85	4.47	4.11	3.76
398	3.24	4.43	6.22	7.16	6.89	6.46	6.03	5.6	5.23	4.86	4.48	4.13	3.75
399	3.24	4.37	6.15	7.16	6.89	6.47	6.04	5.61	5.24	4.87	4.5	4.14	3.76
400	3.24	4.43	6.2	7.16	6.89	6.46	6.03	5.6	5.23	4.85	4.45	4.11	3.76
401	3.24	4.33	6.11	7.15	6.9	6.47	6.04	5.61	5.24	4.86	4.51	4.13	3.75
402	3.24	4.32	6.11	7.15	6.9	6.47	6.04	5.62	5.25	4.87	4.5	4.13	3.76
403	3.24	4.19	5.9	7.02	6.69	6.26	5.83	5.4	5.03	4.65	4.27	3.89	3.47
404	3.24	4.23	5.97	7.02	6.69	6.26	5.83	5.41	5.03	4.66	4.29	3.89	3.52
405	3.24	4.25	5.99	7.02	6.69	6.26	5.83	5.4	5.02	4.64	4.24	3.85	3.49
406	3.24	4.24	5.96	7.02	6.69	6.26	5.83	5.41	5.03	4.66	4.29	3.92	3.53
407	3.24	4.24	5.96	7.02	6.69	6.26	5.83	5.4	5.03	4.65	4.28	3.88	3.51
408	3.24	4.28	6	7.02	6.69	6.26	5.83	5.41	5.04	4.66	4.3	3.92	3.54
409	3.24	4.07	5.72	6.92	6.56	6.13	5.7	5.28	4.9	4.53	4.15	3.76	3.41
410	3.24	4.1	5.76	6.92	6.56	6.13	5.7	5.28	4.9	4.52	4.14	3.77	3.39
411	3.24	4.14	5.8	6.91	6.55	6.13	5.7	5.27	4.9	4.53	4.15	3.76	3.41
412	3.24	4.09	5.75	6.92	6.56	6.13	5.7	5.27	4.88	4.5	4.13	3.75	3.35
413	3.24	4.13	5.8	6.92	6.55	6.13	5.7	5.27	4.9	4.53	4.17	3.79	3.41
414	3.24	4.07	5.72	6.92	6.56	6.13	5.7	5.28	4.9	4.53	4.16	3.77	3.36
415	3.24	3.87	5.5	6.86	6.5	6.07	5.64	5.22	4.84	4.45	4.08	3.71	3.31
416	3.24	4.01	5.65	6.85	6.49	6.06	5.63	5.2	4.83	4.46	4.09	3.72	3.34
417	3.24	4.05	5.66	6.85	6.49	6.06	5.63	5.21	4.83	4.46	4.09	3.74	3.34
418	3.24	3.98	5.59	6.86	6.49	6.06	5.63	5.2	4.83	4.45	4.08	3.72	3.36
419	3.24	4.01	5.62	6.86	6.49	6.06	5.63	5.21	4.83	4.47	4.1	3.75	3.38
420	3.24	4.05	5.68	6.85	6.49	6.06	5.63	5.21	4.83	4.46	4.08	3.68	3.31
421	3.24	3.84	5.43	6.82	6.45	6.02	5.59	5.17	4.78	4.41	4.04	3.65	3.29
422	3.24	3.81	5.39	6.82	6.45	6.02	5.6	5.17	4.79	4.4	4.03	3.64	3.25
423	3.24	3.88	5.47	6.81	6.45	6.02	5.59	5.16	4.79	4.41	4.05	3.68	3.29

424	3.24	3.92	5.5	6.81	6.44	6.02	5.59	5.16	4.78	4.42	4.02	3.65	3.28
425	3.24	3.82	5.41	6.82	6.45	6.02	5.59	5.17	4.79	4.41	4.06	3.65	3.23
426	3.24	3.86	5.45	6.81	6.45	6.02	5.59	5.16	4.79	4.41	4.03	3.63	3.22
427	3.24	3.76	5.35	6.8	6.43	6	5.57	5.14	4.77	4.4	4.03	3.65	3.29
428	3.24	3.86	5.44	6.8	6.43	6	5.57	5.14	4.76	4.39	4.03	3.7	3.29
429	3.24	3.73	5.32	6.8	6.43	6	5.57	5.14	4.77	4.4	4.03	3.66	3.27
430	3.24	3.64	5.21	6.81	6.44	6.01	5.58	5.16	4.78	4.41	4.04	3.65	3.3
431	3.24	3.8	5.38	6.8	6.43	6	5.57	5.15	4.77	4.41	4.02	3.66	3.28
432	3.24	3.72	5.3	6.8	6.43	6.01	5.58	5.15	4.77	4.4	4.03	3.65	3.31
433	3.24	4.2	5.91	7	6.54	5.95	5.37	4.79	4.3	3.78	3.33	2.85	2.28
434	3.24	4.24	5.95	6.99	6.53	5.95	5.38	4.79	4.27	3.78	3.22	2.79	2.27
435	3.24	4.24	5.95	6.99	6.53	5.95	5.37	4.8	4.28	3.73	3.21	2.67	2.2
436	3.24	4.25	5.97	6.99	6.53	5.95	5.37	4.8	4.27	3.73	3.25	2.7	2.02
437	3.24	4.22	5.94	6.99	6.54	5.96	5.37	4.79	4.28	3.77	3.23	2.61	1.49
438	3.24	4.26	5.99	6.99	6.53	5.95	5.37	4.78	4.29	3.77	3.29	2.85	2.31
439	3.24	4.55	6.38	7.32	7.24	6.8	6.26	5.68	5.16	4.62	4.07	3.56	3.09
440	3.24	4.58	6.41	7.33	7.24	6.8	6.27	5.69	5.17	4.65	4.12	3.57	3.05
441	3.24	4.51	6.36	7.32	7.24	6.8	6.27	5.69	5.16	4.63	4.07	3.48	2.88
442	3.24	4.55	6.39	7.32	7.24	6.8	6.27	5.69	5.17	4.64	4.08	3.5	2.93
443	3.24	4.56	6.4	7.33	7.24	6.8	6.27	5.69	5.17	4.63	4.08	3.5	2.89
444	3.24	4.59	6.42	7.33	7.24	6.8	6.26	5.69	5.17	4.64	4.1	3.54	2.93
445	3.24	4.48	6.29	7.22	6.91	6.28	5.6	4.91	4.31	3.74	3.1	2.61	1.89
446	3.24	4.5	6.3	7.22	6.91	6.28	5.6	4.92	4.31	3.76	3.14	2.46	1.99
447	3.24	4.48	6.28	7.22	6.91	6.28	5.6	4.91	4.31	3.68	3.17	2.75	2
448	3.24	4.47	6.27	7.22	6.91	6.28	5.61	4.92	4.32	3.73	3.18	2.31	1.08
449	3.24	4.46	6.26	7.22	6.91	6.28	5.6	4.92	4.3	3.67	3	2.35	1.38
450	3.24	4.48	6.28	7.22	6.91	6.28	5.6	4.92	4.32	3.7	3.09	2.39	1.85
451	3.24	4.31	6.01	6.97	6.42	5.73	5.04	4.35	3.74	3.21	2.36	0	0
452	3.24	4.26	5.97	6.97	6.42	5.73	5.04	4.35	3.75	3.1	2.64	2.1	1.57
453	3.24	4.25	5.95	6.97	6.42	5.74	5.05	4.37	3.79	3.17	2.61	2.11	1.46
454	3.24	4.28	5.98	6.97	6.42	5.73	5.04	4.34	3.77	3.24	2.56	1.56	1.34
455	3.24	4.23	5.94	6.97	6.43	5.73	5.04	4.36	3.74	3.13	2.54	1.76	0
456	3.24	4.26	5.96	6.97	6.42	5.74	5.04	4.37	3.78	3.12	2.53	1.86	1.63
457	3.24	4.06	5.66	6.81	6.21	5.52	4.84	4.15	3.53	2.92	2.32	1.84	1.46
458	3.24	3.88	5.49	6.82	6.23	5.55	4.85	4.17	3.56	3.06	2.46	1.92	1.53
459	3.24	4.02	5.62	6.81	6.22	5.53	4.84	4.16	3.54	2.94	2.13	1.4	1.45
460	3.24	3.95	5.55	6.82	6.23	5.54	4.84	4.19	3.58	2.91	2.37	1.83	0
461	3.24	3.87	5.48	6.82	6.23	5.54	4.85	4.17	3.57	2.94	2.43	1.81	1.52
462	3.24	3.96	5.56	6.82	6.23	5.54	4.85	4.18	3.55	3	2.34	1.76	1.38
463	3.24	3.85	5.4	6.78	6.19	5.5	4.81	4.14	3.56	2.96	2.41	1.98	0
464	3.24	3.76	5.33	6.78	6.19	5.51	4.83	4.16	3.52	2.96	2.44	2.1	1.4
465	3.24	3.86	5.41	6.78	6.19	5.5	4.8	4.13	3.53	2.92	2.41	1.41	1.08
466	3.24	3.81	5.4	6.78	6.19	5.5	4.81	4.11	3.52	2.86	2.21	1.41	0
467	3.24	3.82	5.38	6.78	6.19	5.5	4.81	4.13	3.53	2.98	2.4	1.96	1.46
468	3.24	3.91	5.48	6.77	6.18	5.49	4.8	4.12	3.51	2.87	2.31	1.45	0
469	3.24	4.45	6.25	7.2	6.84	6.13	5.38	4.6	3.91	3.18	2.41	1.92	1.34
470	3.24	4.47	6.26	7.2	6.85	6.13	5.37	4.59	3.89	3.26	2.65	1.98	1.26
471	3.24	4.47	6.27	7.2	6.84	6.14	5.37	4.57	3.88	3.25	2.74	1.87	0.48
472	3.24	4.46	6.26	7.2	6.84	6.14	5.37	4.6	3.92	3.18	2.41	1.72	0.85
473	3.24	4.52	6.31	7.2	6.84	6.13	5.36	4.58	3.9	3.2	2.49	1.98	0.7
474	3.24	4.46	6.27	7.2	6.85	6.14	5.38	4.62	3.9	3.25	2.43	1.74	0
475	3.24	4.28	5.96	6.96	6.33	5.55	4.78	3.97	3.29	2.51	1.34	0.7	0
476	3.24	4.19	5.89	6.96	6.34	5.55	4.77	4.01	3.36	2.71	2.21	1.73	1.32
477	3.24	4.16	5.86	6.96	6.34	5.55	4.77	3.99	3.29	2.58	1.82	1.46	1.36

478	3.24	4.23	5.92	6.96	6.33	5.56	4.78	4.02	3.37	2.66	2.11	1.68	0
479	3.24	4.22	5.93	6.96	6.33	5.56	4.77	4	3.31	2.61	2.09	1.72	0
480	3.24	4.2	5.9	6.96	6.33	5.55	4.78	3.99	3.26	2.59	1.76	1.43	0
481	3.24	3.88	5.47	6.81	6.14	5.36	4.59	3.82	3.17	2.41	1.82	1.49	0
482	3.24	3.93	5.52	6.8	6.13	5.35	4.58	3.82	3.11	2.33	1.53	0	0
483	3.24	3.97	5.57	6.8	6.13	5.35	4.57	3.78	3.01	2.11	1.26	0	0
484	3.24	3.96	5.55	6.8	6.13	5.35	4.57	3.79	3.1	2.05	0	0	0
485	3.24	3.95	5.56	6.8	6.13	5.35	4.59	3.82	3.05	2.05	1.18	0	0
486	3.24	4	5.6	6.8	6.12	5.34	4.58	3.8	3.15	2.53	1.9	1.72	0
487	3.24	4.22	5.91	6.95	6.29	5.47	4.65	3.84	3.23	2.59	1.88	1.57	0
488	3.24	4.19	5.89	6.95	6.3	5.48	4.67	3.82	3.12	2.18	1.46	1.34	0
489	3.24	4.22	5.92	6.95	6.29	5.48	4.64	3.78	3.14	2.33	1.41	0	0
490	3.24	4.18	5.88	6.95	6.3	5.48	4.64	3.82	3.11	2.33	1.72	0	0
491	3.24	4.22	5.92	6.95	6.29	5.47	4.65	3.83	3.08	2.22	1.26	0	0
492	3.24	4.25	5.94	6.95	6.29	5.47	4.65	3.81	3.13	2.47	1.77	0	0
493	3.24	4.52	6.49	7.01	6.65	6.22	5.79	5.36	4.98	4.61	4.26	3.88	3.49
494	3.24	4.51	6.49	7.01	6.65	6.22	5.79	5.36	4.99	4.62	4.24	3.86	3.49
495	3.24	4.45	6.43	7.01	6.66	6.23	5.8	5.37	4.99	4.61	4.23	3.86	3.49
496	3.24	4.5	6.49	7.01	6.65	6.22	5.79	5.37	5	4.64	4.27	3.89	3.52
497	3.24	4.44	6.43	7.01	6.65	6.23	5.79	5.37	5	4.63	4.26	3.89	3.49
498	3.24	4.45	6.44	7.01	6.65	6.22	5.8	5.37	4.99	4.61	4.24	3.89	3.52
499	3.24	4.66	6.77	7.1	7.03	6.93	6.84	6.74	6.66	6.58	6.5	6.41	6.33
500	3.24	4.65	6.77	7.1	7.03	6.93	6.84	6.74	6.66	6.58	6.49	6.41	6.33
501	3.24	4.67	6.78	7.1	7.03	6.93	6.84	6.74	6.66	6.58	6.49	6.41	6.33
502	3.24	4.65	6.77	7.1	7.03	6.93	6.84	6.74	6.66	6.58	6.49	6.41	6.33
503	3.24	4.63	6.76	7.1	7.03	6.93	6.84	6.74	6.66	6.58	6.5	6.41	6.33
504	3.24	4.64	6.76	7.1	7.03	6.93	6.84	6.74	6.66	6.58	6.5	6.41	6.33
505	3.24	4.86	6.94	7.35	7.33	7.16	6.98	6.8	6.65	6.49	6.33	6.18	6.02
506	3.24	4.85	6.93	7.35	7.33	7.16	6.98	6.8	6.65	6.49	6.33	6.18	6.02
507	3.24	4.87	6.94	7.35	7.33	7.16	6.98	6.8	6.65	6.49	6.34	6.18	6.03
508	3.24	4.8	6.92	7.35	7.33	7.16	6.98	6.8	6.65	6.49	6.34	6.18	6.03
509	3.24	4.79	6.92	7.35	7.33	7.16	6.98	6.8	6.65	6.49	6.34	6.18	6.02
510	3.24	4.82	6.92	7.35	7.33	7.16	6.98	6.8	6.65	6.49	6.34	6.18	6.03
511	3.24	4.64	6.76	7.07	6.93	6.75	6.57	6.39	6.24	6.08	5.93	5.77	5.61
512	3.24	4.58	6.71	7.07	6.93	6.75	6.57	6.4	6.24	6.08	5.93	5.77	5.62
513	3.24	4.6	6.73	7.07	6.93	6.75	6.57	6.39	6.24	6.08	5.93	5.77	5.62
514	3.24	4.68	6.79	7.07	6.93	6.75	6.57	6.39	6.24	6.08	5.92	5.77	5.61
515	3.24	4.64	6.76	7.07	6.93	6.75	6.57	6.39	6.24	6.08	5.93	5.77	5.62
516	3.24	4.63	6.75	7.07	6.93	6.75	6.57	6.39	6.24	6.08	5.92	5.77	5.61
517	3.24	4.43	6.51	6.88	6.73	6.55	6.37	6.19	6.04	5.88	5.72	5.57	5.42
518	3.24	4.5	6.56	6.88	6.72	6.55	6.37	6.19	6.03	5.88	5.72	5.57	5.41
519	3.24	4.35	6.43	6.88	6.73	6.55	6.37	6.19	6.04	5.88	5.72	5.57	5.41
520	3.24	4.38	6.46	6.88	6.73	6.55	6.37	6.19	6.03	5.88	5.72	5.57	5.41
521	3.24	4.39	6.48	6.88	6.73	6.55	6.37	6.19	6.04	5.88	5.72	5.57	5.41
522	3.24	4.38	6.46	6.88	6.73	6.55	6.37	6.19	6.03	5.88	5.73	5.57	5.41
523	3.24	4.9	6.96	7.41	7.38	7.09	6.72	6.31	5.95	5.59	5.22	4.85	4.47
524	3.24	4.95	6.98	7.42	7.38	7.08	6.71	6.31	5.95	5.58	5.21	4.84	4.45
525	3.24	4.9	6.96	7.41	7.38	7.09	6.71	6.31	5.95	5.58	5.21	4.84	4.47
526	3.24	4.87	6.95	7.41	7.39	7.09	6.72	6.32	5.96	5.6	5.22	4.85	4.49
527	3.24	4.89	6.96	7.41	7.38	7.09	6.71	6.31	5.95	5.59	5.22	4.86	4.48
528	3.24	4.9	6.96	7.41	7.38	7.09	6.72	6.32	5.95	5.58	5.21	4.82	4.43
529	3.24	4.78	6.9	7.29	7.09	6.68	6.26	5.83	5.45	5.07	4.68	4.32	3.96
530	3.24	4.85	6.92	7.29	7.08	6.68	6.25	5.83	5.45	5.07	4.7	4.34	3.96
531	3.24	4.8	6.91	7.29	7.09	6.68	6.26	5.83	5.46	5.09	4.72	4.35	3.96

532	3.24	4.84	6.92	7.29	7.09	6.68	6.25	5.83	5.46	5.09	4.71	4.34	3.97
533	3.24	4.85	6.92	7.29	7.09	6.68	6.25	5.82	5.44	5.07	4.71	4.32	3.94
534	3.24	4.82	6.92	7.29	7.09	6.68	6.25	5.82	5.44	5.06	4.69	4.33	3.93
535	3.24	4.61	6.72	7	6.64	6.21	5.78	5.35	4.97	4.6	4.23	3.86	3.51
536	3.24	4.64	6.75	7	6.64	6.21	5.78	5.35	4.97	4.59	4.22	3.87	3.53
537	3.24	4.6	6.71	7	6.64	6.21	5.78	5.35	4.98	4.6	4.23	3.86	3.45
538	3.24	4.69	6.78	7	6.64	6.21	5.78	5.36	4.99	4.62	4.24	3.86	3.51
539	3.24	4.63	6.74	7	6.64	6.21	5.78	5.35	4.97	4.59	4.2	3.85	3.46
540	3.24	4.63	6.73	7	6.64	6.21	5.78	5.35	4.98	4.6	4.22	3.85	3.51
541	3.24	4.38	6.44	6.8	6.43	6	5.57	5.14	4.76	4.4	4.02	3.63	3.23
542	3.24	4.39	6.45	6.8	6.43	6	5.57	5.14	4.76	4.39	4.03	3.67	3.28
543	3.24	4.32	6.39	6.8	6.43	6	5.58	5.14	4.77	4.4	4.04	3.7	3.33
544	3.24	4.4	6.46	6.8	6.43	6	5.57	5.14	4.77	4.39	3.98	3.61	3.21
545	3.24	4.31	6.38	6.81	6.43	6	5.57	5.14	4.77	4.4	4.04	3.67	3.33
546	3.24	4.38	6.44	6.8	6.43	6	5.57	5.13	4.76	4.39	4.02	3.64	3.29
547	3.24	4.27	6.31	6.75	6.38	5.95	5.52	5.1	4.72	4.35	3.97	3.56	3.15
548	3.24	4.21	6.27	6.76	6.38	5.96	5.53	5.1	4.73	4.36	3.98	3.63	3.27
549	3.24	4.27	6.32	6.75	6.38	5.95	5.52	5.1	4.72	4.35	4	3.62	3.21
550	3.24	4.27	6.31	6.75	6.38	5.95	5.52	5.09	4.71	4.34	3.95	3.56	3.19
551	3.24	4.29	6.33	6.75	6.38	5.95	5.52	5.09	4.71	4.35	3.95	3.58	3.23
552	3.24	4.25	6.3	6.76	6.38	5.95	5.52	5.08	4.71	4.36	4.01	3.6	3.2
553	3.24	4.84	6.91	7.23	6.85	6.21	5.53	4.83	4.22	3.62	3.06	2.5	1.69
554	3.24	4.86	6.92	7.23	6.85	6.21	5.53	4.86	4.27	3.71	3.18	2.56	1.26
555	3.24	4.84	6.91	7.23	6.85	6.21	5.53	4.84	4.26	3.69	3.1	2.62	1.89
556	3.24	4.81	6.9	7.23	6.85	6.21	5.53	4.85	4.25	3.62	2.98	2.36	1.76
557	3.24	4.84	6.91	7.23	6.85	6.21	5.53	4.84	4.23	3.62	3.06	2.53	1.69
558	3.24	4.82	6.9	7.23	6.85	6.21	5.53	4.85	4.27	3.66	3.07	2.44	1.56
559	3.24	4.57	6.68	6.93	6.35	5.66	4.98	4.32	3.68	3.15	2.51	1.75	1.43
560	3.24	4.63	6.72	6.92	6.34	5.65	4.95	4.26	3.7	3.12	2.56	1.71	0.78
561	3.24	4.58	6.68	6.93	6.34	5.66	4.97	4.29	3.66	2.98	2.4	1.32	0
562	3.24	4.71	6.78	6.92	6.33	5.64	4.96	4.26	3.65	3.11	2.59	1.94	0.48
563	3.24	4.64	6.73	6.92	6.34	5.65	4.97	4.29	3.7	3.01	2.39	1.97	0.3
564	3.24	4.61	6.71	6.92	6.34	5.65	4.98	4.32	3.76	3.19	2.57	1.65	0
565	3.24	4.37	6.42	6.72	6.12	5.43	4.74	4.03	3.41	2.93	2.32	1.65	0
566	3.24	4.31	6.36	6.73	6.13	5.44	4.74	4.06	3.49	2.87	2.21	2.05	1.15
567	3.24	4.38	6.42	6.72	6.12	5.43	4.75	4.05	3.44	2.85	2.21	1.99	1.4
568	3.24	4.39	6.43	6.72	6.12	5.43	4.74	4.06	3.48	2.87	2.23	1.4	0
569	3.24	4.37	6.42	6.72	6.12	5.43	4.74	4.03	3.42	2.89	2.29	1.79	0
570	3.24	4.31	6.37	6.73	6.13	5.43	4.74	4.06	3.46	2.82	2.23	1.52	0.78
571	3.24	4.6	6.69	6.9	6.24	5.46	4.69	3.93	3.24	2.58	2.09	1.4	0
572	3.24	4.7	6.77	6.89	6.23	5.45	4.66	3.9	3.27	2.67	1.67	0	0
573	3.24	4.61	6.7	6.9	6.24	5.46	4.68	3.9	3.23	2.39	1.51	1.38	0.85
574	3.24	4.64	6.72	6.89	6.23	5.45	4.68	3.9	3.27	2.62	2.08	1.83	1.08
575	3.24	4.69	6.76	6.89	6.23	5.45	4.67	3.91	3.2	2.66	2	1.3	0.3
576	3.24	4.61	6.7	6.9	6.24	5.46	4.67	3.93	3.26	2.54	1.99	1.41	0
577	3.24	4.75	6.87	7.07	6.92	6.75	6.57	6.39	6.23	6.08	5.92	5.77	5.61
578	3.24	4.79	6.88	7.07	6.92	6.75	6.57	6.39	6.24	6.08	5.92	5.77	5.61
579	3.24	4.74	6.87	7.07	6.92	6.75	6.57	6.39	6.24	6.08	5.92	5.77	5.61
580	3.24	4.69	6.86	7.07	6.92	6.75	6.57	6.39	6.24	6.08	5.92	5.77	5.61
581	3.24	4.78	6.88	7.07	6.92	6.74	6.57	6.39	6.23	6.08	5.92	5.77	5.61
582	3.24	4.75	6.87	7.07	6.92	6.75	6.57	6.39	6.23	6.08	5.92	5.77	5.61
583	3.24	4.96	6.99	7.29	7.08	6.67	6.24	5.81	5.44	5.06	4.68	4.28	3.91
584	3.24	5.01	7	7.29	7.07	6.67	6.24	5.81	5.44	5.07	4.71	4.34	3.96
585	3.24	4.96	6.99	7.29	7.08	6.67	6.24	5.81	5.44	5.05	4.66	4.3	3.91

586	3.24	4.95	6.98	7.29	7.08	6.67	6.24	5.81	5.44	5.07	4.68	4.32	3.94
587	3.24	4.89	6.97	7.29	7.08	6.67	6.25	5.82	5.45	5.07	4.69	4.33	3.98
588	3.24	4.93	6.98	7.29	7.08	6.67	6.24	5.82	5.44	5.07	4.7	4.33	3.98
589	3.24	4.75	6.86	6.99	6.63	6.2	5.77	5.34	4.97	4.59	4.22	3.81	3.45
590	3.24	4.74	6.86	6.99	6.63	6.2	5.77	5.34	4.95	4.57	4.21	3.85	3.48
591	3.24	4.76	6.87	6.99	6.63	6.2	5.77	5.34	4.98	4.6	4.22	3.84	3.5
592	3.24	4.76	6.86	6.99	6.62	6.2	5.77	5.34	4.96	4.58	4.22	3.81	3.45
593	3.24	4.72	6.86	6.99	6.63	6.2	5.77	5.34	4.96	4.6	4.22	3.82	3.43
594	3.24	4.71	6.85	6.99	6.63	6.2	5.77	5.34	4.96	4.57	4.2	3.84	3.5
595	3.24	4.46	6.62	6.79	6.42	5.99	5.56	5.14	4.77	4.39	4.01	3.63	3.26
596	3.24	4.52	6.67	6.79	6.42	5.99	5.56	5.13	4.76	4.39	4.03	3.62	3.24
597	3.24	4.5	6.65	6.79	6.42	5.99	5.56	5.12	4.75	4.39	4.02	3.65	3.28
598	3.24	4.45	6.62	6.79	6.42	5.99	5.56	5.13	4.75	4.38	4	3.63	3.22
599	3.24	4.46	6.63	6.79	6.42	5.99	5.57	5.14	4.76	4.39	4.03	3.69	3.29
600	3.24	4.44	6.62	6.79	6.42	5.99	5.56	5.13	4.76	4.39	4.02	3.65	3.27
601	3.24	4.74	6.85	6.91	6.32	5.63	4.93	4.24	3.62	2.95	2.27	1.85	1.41
602	3.24	4.7	6.85	6.91	6.32	5.63	4.94	4.24	3.58	2.93	2.4	1.69	0
603	3.24	4.73	6.85	6.91	6.32	5.63	4.94	4.24	3.64	3.06	2.38	1.89	1.23
604	3.24	4.66	6.84	6.91	6.32	5.63	4.94	4.26	3.65	3.11	2.62	1.97	1.26
605	3.24	4.69	6.84	6.91	6.32	5.63	4.95	4.28	3.66	3.12	2.48	1.81	0
606	3.24	4.73	6.85	6.91	6.32	5.64	4.95	4.26	3.65	3	2.36	1.91	0
607	3.24	4.78	6.88	6.99	6.62	6.19	5.77	5.33	4.95	4.58	4.19	3.82	3.45
608	3.24	4.76	6.87	6.99	6.62	6.2	5.77	5.34	4.98	4.61	4.23	3.87	3.48
609	3.24	4.84	6.89	6.99	6.62	6.19	5.77	5.33	4.96	4.59	4.21	3.8	3.42
610	3.24	4.79	6.88	6.99	6.62	6.2	5.77	5.34	4.98	4.6	4.19	3.79	3.36
611	3.24	4.73	6.87	6.99	6.63	6.2	5.76	5.33	4.96	4.59	4.22	3.86	3.49
612	3.24	4.75	6.87	6.99	6.62	6.2	5.77	5.34	4.96	4.59	4.21	3.82	3.45
613	3.24	4.46	6.62	6.79	6.42	5.99	5.56	5.14	4.77	4.39	4.01	3.63	3.26
614	3.24	4.52	6.67	6.79	6.42	5.99	5.56	5.13	4.76	4.39	4.03	3.62	3.24
615	3.24	4.5	6.65	6.79	6.42	5.99	5.56	5.12	4.75	4.39	4.02	3.65	3.28
616	3.24	4.45	6.62	6.79	6.42	5.99	5.56	5.13	4.75	4.38	4	3.63	3.22
617	3.24	4.46	6.63	6.79	6.42	5.99	5.57	5.14	4.76	4.39	4.03	3.69	3.29
618	3.24	4.44	6.62	6.79	6.42	5.99	5.56	5.13	4.76	4.39	4.02	3.65	3.27
619	3.24	3.66	4.7	6.21	6.86	6.43	6	5.58	5.21	4.84	4.47	4.08	3.72
620	3.24	3.63	4.67	6.18	6.86	6.44	6.01	5.58	5.21	4.82	4.44	4.05	3.68
621	3.24	3.68	4.72	6.23	6.86	6.43	6	5.57	5.2	4.82	4.45	4.06	3.65
622	3.24	3.65	4.68	6.18	6.86	6.44	6.01	5.58	5.21	4.83	4.44	4.07	3.65
623	3.24	3.7	4.73	6.24	6.85	6.43	6	5.57	5.19	4.81	4.45	4.07	3.71
624	3.24	3.69	4.74	6.25	6.86	6.43	6	5.57	5.2	4.82	4.44	4.08	3.72
625	3.24	4.25	5.99	7.07	6.95	6.77	6.59	6.42	6.26	6.1	5.95	5.79	5.64
626	3.24	4.3	6.04	7.07	6.95	6.77	6.59	6.41	6.26	6.1	5.95	5.79	5.64
627	3.24	4.3	6.04	7.07	6.95	6.77	6.59	6.42	6.26	6.1	5.95	5.8	5.64
628	3.24	4.29	6.02	7.07	6.95	6.77	6.59	6.41	6.26	6.1	5.95	5.79	5.63
629	3.24	4.23	5.98	7.07	6.95	6.77	6.59	6.42	6.26	6.11	5.95	5.79	5.64
630	3.24	4.21	5.94	7.07	6.95	6.77	6.59	6.42	6.26	6.11	5.95	5.79	5.63
631	3.24	4.45	6.28	7.27	7.12	6.72	6.29	5.86	5.49	5.12	4.74	4.36	3.96
632	3.24	4.5	6.32	7.27	7.12	6.72	6.29	5.86	5.48	5.11	4.73	4.36	4
633	3.24	4.44	6.26	7.27	7.12	6.72	6.3	5.87	5.5	5.13	4.75	4.38	4.02
634	3.24	4.48	6.3	7.27	7.12	6.72	6.29	5.86	5.49	5.1	4.72	4.37	3.98
635	3.24	4.49	6.3	7.27	7.12	6.72	6.3	5.87	5.51	5.14	4.75	4.36	3.98
636	3.24	4.5	6.32	7.27	7.12	6.72	6.29	5.86	5.48	5.11	4.74	4.37	4
637	3.24	4.22	5.93	7.02	6.69	6.26	5.83	5.41	5.03	4.67	4.3	3.9	3.56
638	3.24	4.25	5.97	7.02	6.69	6.26	5.84	5.4	5.03	4.67	4.28	3.9	3.5
639	3.24	4.31	6.03	7.02	6.68	6.25	5.83	5.39	5.02	4.63	4.25	3.91	3.53

640	3.24	4.18	5.91	7.02	6.69	6.26	5.84	5.41	5.03	4.65	4.27	3.9	3.54
641	3.24	4.21	5.93	7.02	6.69	6.26	5.83	5.4	5.03	4.66	4.28	3.93	3.55
642	3.24	4.24	5.96	7.02	6.69	6.26	5.83	5.4	5.02	4.65	4.28	3.87	3.52
643	3.24	4.11	5.74	6.85	6.48	6.05	5.63	5.2	4.83	4.45	4.07	3.68	3.34
644	3.24	3.98	5.62	6.86	6.49	6.06	5.63	5.21	4.84	4.46	4.07	3.67	3.31
645	3.24	3.96	5.58	6.86	6.49	6.06	5.64	5.21	4.84	4.47	4.08	3.72	3.33
646	3.24	3.98	5.62	6.86	6.49	6.06	5.63	5.2	4.83	4.46	4.07	3.67	3.26
647	3.24	4.04	5.67	6.85	6.49	6.06	5.63	5.19	4.81	4.44	4.06	3.7	3.32
648	3.24	3.93	5.53	6.86	6.5	6.07	5.64	5.21	4.84	4.45	4.08	3.72	3.34
649	3.24	4.31	6.01	6.97	6.42	5.73	5.04	4.33	3.74	3.15	2.37	0.7	0
650	3.24	4.13	5.84	6.98	6.43	5.75	5.06	4.37	3.78	3.18	2.51	1.96	1.46
651	3.24	4.22	5.93	6.97	6.42	5.73	5.05	4.36	3.73	3.18	2.52	2.13	0.3
652	3.24	4.24	5.93	6.97	6.43	5.74	5.05	4.37	3.74	3.11	2.5	2.16	1.71
653	3.24	4.26	5.97	6.97	6.42	5.73	5.04	4.34	3.72	3.15	2.49	1.94	0
654	3.24	4.25	5.95	6.97	6.42	5.74	5.04	4.34	3.73	3.2	2.62	2.21	1.58
655	3.24	4.62	6.73	7	6.64	6.21	5.78	5.36	4.98	4.61	4.24	3.86	3.51
656	3.24	4.62	6.73	7	6.64	6.21	5.78	5.35	4.98	4.59	4.22	3.83	3.45
657	3.24	4.59	6.71	7	6.64	6.21	5.79	5.36	4.97	4.61	4.23	3.83	3.46
658	3.24	4.66	6.76	7	6.63	6.21	5.78	5.35	4.98	4.6	4.21	3.83	3.43
659	3.24	4.62	6.73	7	6.64	6.21	5.78	5.36	4.99	4.61	4.23	3.85	3.47
660	3.24	4.6	6.71	7	6.64	6.21	5.79	5.36	4.98	4.6	4.21	3.81	3.39
661	3.24	3.13	3.57	4.1	5.19	6.34	6.8	6.53	6.19	5.82	5.46	5.1	4.74
662	3.24	3.11	3.5	4.02	5.13	6.28	6.8	6.55	6.2	5.84	5.48	5.12	4.75
663	3.24	3.1	3.48	4.03	5.14	6.29	6.8	6.54	6.2	5.84	5.49	5.12	4.76
664	3.24	3.22	3.57	4.05	5.17	6.32	6.8	6.54	6.19	5.83	5.46	5.1	4.72
665	3.24	3.16	3.57	4.08	5.2	6.33	6.8	6.53	6.18	5.82	5.46	5.09	4.73
666	3.24	3.21	3.61	4.13	5.23	6.36	6.8	6.52	6.17	5.81	5.45	5.08	4.72
667	3.24	3.32	3.89	4.61	6.26	7.02	6.85	6.68	6.52	6.36	6.21	6.05	5.9
668	3.24	3.15	3.72	4.49	6.16	7.02	6.86	6.69	6.53	6.37	6.22	6.06	5.91
669	3.24	3.19	3.71	4.46	6.11	7.02	6.87	6.69	6.54	6.38	6.23	6.07	5.92
670	3.24	3.29	3.81	4.59	6.24	7.02	6.86	6.68	6.52	6.37	6.21	6.06	5.9
671	3.24	3.2	3.72	4.46	6.11	7.02	6.87	6.69	6.54	6.38	6.23	6.07	5.91
672	3.24	3.34	3.9	4.64	6.28	7.01	6.85	6.67	6.52	6.36	6.21	6.05	5.9
673	3.24	3.58	4.2	5.09	6.97	7.16	6.77	6.35	5.98	5.61	5.23	4.86	4.49
674	3.24	3.57	4.2	5.11	6.98	7.16	6.77	6.35	5.97	5.6	5.23	4.84	4.48
675	3.24	3.53	4.15	5.05	6.94	7.16	6.78	6.35	5.98	5.61	5.24	4.86	4.46
676	3.24	3.49	4.1	5	6.91	7.17	6.79	6.37	6	5.62	5.24	4.87	4.5
677	3.24	3.58	4.19	5.1	6.98	7.16	6.77	6.35	5.98	5.6	5.22	4.84	4.45
678	3.24	3.49	4.12	5.03	6.93	7.17	6.78	6.36	5.99	5.62	5.26	4.89	4.49
679	3.24	3.24	3.74	4.44	5.97	6.89	6.56	6.13	5.76	5.38	5.01	4.64	4.26
680	3.24	3.22	3.78	4.47	6.01	6.89	6.55	6.12	5.75	5.37	4.99	4.6	4.23
681	3.24	3.23	3.77	4.47	6	6.89	6.55	6.12	5.75	5.37	5	4.62	4.26
682	3.24	3.25	3.77	4.48	6.01	6.89	6.55	6.12	5.75	5.38	5	4.61	4.24
683	3.24	3.23	3.79	4.48	6.01	6.89	6.55	6.12	5.75	5.38	5.01	4.63	4.26
684	3.24	3.27	3.76	4.44	5.97	6.89	6.56	6.14	5.76	5.39	5.02	4.64	4.27
685	3.24	2.93	3.32	3.88	5.05	6.23	6.6	6.28	5.94	5.58	5.22	4.87	4.51
686	3.24	3.02	3.43	3.94	5.12	6.29	6.58	6.26	5.91	5.55	5.19	4.83	4.48
687	3.24	2.87	3.35	3.94	5.12	6.3	6.58	6.26	5.91	5.55	5.18	4.83	4.47
688	3.24	3.02	3.43	3.96	5.13	6.3	6.58	6.26	5.91	5.55	5.19	4.82	4.46
689	3.24	2.96	3.45	4.05	5.23	6.35	6.57	6.23	5.88	5.53	5.17	4.8	4.43
690	3.24	3.05	3.48	4.07	5.24	6.37	6.57	6.23	5.88	5.52	5.16	4.8	4.45
691	3.24	3.25	3.78	4.43	5.83	6.77	6.33	5.69	5.1	4.5	3.92	3.35	2.61
692	3.24	3.28	3.71	4.36	5.79	6.77	6.35	5.7	5.12	4.54	3.97	3.37	2.84
693	3.24	3.12	3.57	4.21	5.63	6.75	6.41	5.77	5.2	4.62	4.03	3.4	2.84

694	3.24	3.24	3.72	4.39	5.8	6.77	6.35	5.7	5.12	4.53	3.97	3.41	2.77
695	3.24	3.2	3.7	4.33	5.75	6.77	6.36	5.71	5.13	4.55	3.96	3.39	2.88
696	3.24	3.22	3.71	4.38	5.79	6.77	6.35	5.71	5.12	4.54	3.96	3.38	2.67
697	3.24	3.4	4.14	5.21	7.05	7.02	6.93	6.84	6.75	6.67	6.59	6.51	6.42
698	3.24	3.41	4.14	5.22	7.05	7.02	6.93	6.83	6.75	6.67	6.59	6.5	6.42
699	3.24	3.33	4.06	5.12	7.03	7.03	6.93	6.84	6.75	6.67	6.59	6.51	6.42
700	3.24	3.48	4.22	5.27	7.06	7.02	6.93	6.83	6.75	6.67	6.59	6.5	6.42
701	3.24	3.36	4.08	5.15	7.04	7.03	6.93	6.84	6.75	6.67	6.59	6.51	6.42
702	3.24	3.32	4.09	5.15	7.04	7.03	6.93	6.84	6.75	6.67	6.59	6.5	6.42
703	3.24	3.72	4.55	5.8	7.37	7.29	7.11	6.94	6.78	6.63	6.47	6.32	6.16
704	3.24	3.68	4.54	5.79	7.37	7.29	7.12	6.94	6.78	6.63	6.47	6.31	6.16
705	3.24	3.64	4.47	5.72	7.36	7.29	7.12	6.94	6.78	6.63	6.47	6.32	6.16
706	3.24	3.66	4.53	5.79	7.37	7.29	7.11	6.94	6.78	6.62	6.47	6.31	6.16
707	3.24	3.72	4.55	5.8	7.37	7.29	7.11	6.93	6.78	6.62	6.47	6.31	6.15
708	3.24	3.71	4.57	5.82	7.37	7.29	7.11	6.94	6.78	6.62	6.47	6.31	6.16
709	3.24	3.38	4.13	5.17	7.01	6.93	6.75	6.57	6.42	6.26	6.1	5.95	5.79
710	3.24	3.32	4.03	5.09	7	6.93	6.75	6.58	6.42	6.26	6.11	5.95	5.79
711	3.24	3.46	4.18	5.23	7.02	6.92	6.75	6.57	6.41	6.25	6.1	5.94	5.78
712	3.24	3.42	4.16	5.2	7.02	6.93	6.75	6.57	6.41	6.26	6.1	5.95	5.79
713	3.24	3.38	4.11	5.16	7.01	6.93	6.75	6.57	6.42	6.26	6.11	5.95	5.79
714	3.24	3.37	4.09	5.15	7.01	6.93	6.75	6.57	6.42	6.26	6.11	5.95	5.8
715	3.24	3.05	3.61	4.49	6.38	6.79	6.61	6.44	6.28	6.13	5.97	5.81	5.66
716	3.24	3.1	3.73	4.6	6.47	6.78	6.61	6.43	6.27	6.12	5.96	5.8	5.65
717	3.24	3.02	3.61	4.5	6.39	6.79	6.61	6.44	6.28	6.12	5.97	5.81	5.65
718	3.24	3.09	3.66	4.54	6.42	6.79	6.61	6.43	6.28	6.12	5.96	5.81	5.65
719	3.24	3.08	3.73	4.59	6.46	6.78	6.61	6.43	6.27	6.12	5.96	5.8	5.65
720	3.24	3.14	3.74	4.64	6.49	6.78	6.6	6.42	6.27	6.11	5.96	5.8	5.65
721	3.24	3.67	4.55	5.83	7.44	7.32	7	6.62	6.26	5.9	5.53	5.15	4.77
722	3.24	3.83	4.71	5.99	7.44	7.31	6.98	6.6	6.24	5.88	5.51	5.14	4.77
723	3.24	3.76	4.63	5.92	7.44	7.31	6.99	6.6	6.25	5.89	5.52	5.14	4.77
724	3.24	3.75	4.61	5.9	7.44	7.31	6.99	6.61	6.25	5.89	5.52	5.15	4.78
725	3.24	3.77	4.65	5.93	7.44	7.31	6.99	6.61	6.26	5.89	5.53	5.16	4.79
726	3.24	3.71	4.59	5.87	7.44	7.32	6.99	6.61	6.26	5.9	5.53	5.16	4.78
727	3.24	3.64	4.47	5.67	7.28	7.01	6.6	6.17	5.8	5.42	5.05	4.67	4.32
728	3.24	3.68	4.53	5.74	7.28	7	6.59	6.16	5.79	5.41	5.04	4.65	4.27
729	3.24	3.67	4.49	5.7	7.28	7.01	6.59	6.17	5.79	5.41	5.04	4.65	4.27
730	3.24	3.71	4.54	5.73	7.28	7	6.59	6.16	5.78	5.4	5.02	4.65	4.27
731	3.24	3.68	4.51	5.72	7.28	7	6.59	6.17	5.79	5.41	5.04	4.67	4.3
732	3.24	3.67	4.48	5.69	7.28	7.01	6.59	6.17	5.8	5.43	5.06	4.68	4.31
733	3.24	3.36	4.01	5.01	6.89	6.68	6.25	5.82	5.45	5.07	4.68	4.29	3.9
734	3.24	3.41	4.07	5.05	6.91	6.67	6.25	5.82	5.44	5.05	4.67	4.31	3.94
735	3.24	3.49	4.23	5.23	6.94	6.64	6.22	5.79	5.41	5.03	4.64	4.25	3.87
736	3.24	3.48	4.15	5.16	6.93	6.66	6.23	5.8	5.43	5.05	4.67	4.3	3.92
737	3.24	3.47	4.16	5.14	6.93	6.66	6.23	5.8	5.42	5.04	4.68	4.31	3.91
738	3.24	3.47	4.14	5.13	6.93	6.66	6.23	5.8	5.43	5.06	4.68	4.31	3.91
739	3.24	3.24	3.81	4.64	6.36	6.59	6.17	5.75	5.37	5	4.63	4.25	3.85
740	3.24	3.1	3.71	4.56	6.32	6.6	6.19	5.77	5.39	5.02	4.64	4.25	3.89
741	3.24	3.04	3.64	4.44	6.22	6.63	6.21	5.78	5.41	5.04	4.66	4.27	3.92
742	3.24	3.05	3.64	4.48	6.26	6.62	6.2	5.78	5.41	5.04	4.66	4.28	3.91
743	3.24	3.14	3.74	4.55	6.3	6.61	6.19	5.76	5.39	5.02	4.65	4.29	3.94
744	3.24	3.15	3.71	4.54	6.31	6.6	6.19	5.76	5.39	5.01	4.63	4.26	3.89
745	3.24	2.91	3.51	4.28	5.95	6.62	6.23	5.81	5.44	5.06	4.69	4.32	3.94
746	3.24	3.13	3.7	4.48	6.14	6.58	6.18	5.76	5.38	5.01	4.63	4.26	3.88
747	3.24	2.98	3.55	4.32	5.98	6.61	6.22	5.8	5.42	5.05	4.68	4.3	3.92

748	3.24	3.01	3.58	4.31	6	6.61	6.22	5.79	5.42	5.05	4.68	4.3	3.93
749	3.24	3.08	3.67	4.43	6.1	6.59	6.19	5.77	5.4	5.03	4.66	4.28	3.88
750	3.24	3.07	3.63	4.41	6.07	6.6	6.2	5.77	5.4	5.03	4.66	4.29	3.91
751	3.24	3.71	4.5	5.68	7.2	6.74	6.09	5.41	4.83	4.24	3.63	2.9	2.47
752	3.24	3.71	4.49	5.66	7.2	6.75	6.09	5.42	4.83	4.25	3.64	3.03	2.52
753	3.24	3.65	4.46	5.62	7.2	6.75	6.1	5.41	4.81	4.23	3.62	2.92	2.26
754	3.24	3.66	4.48	5.65	7.2	6.75	6.09	5.41	4.84	4.24	3.66	3.01	2.2
755	3.24	3.68	4.49	5.67	7.2	6.74	6.09	5.41	4.81	4.22	3.63	3.07	2.21
756	3.24	3.67	4.48	5.64	7.2	6.75	6.1	5.42	4.82	4.23	3.63	3.05	2.41
757	3.24	3.39	4.04	4.98	6.8	6.45	5.77	5.09	4.48	3.87	3.3	2.64	1.89
758	3.24	3.38	4.05	4.98	6.8	6.45	5.77	5.08	4.47	3.87	3.35	2.65	1.95
759	3.24	3.29	3.95	4.92	6.77	6.47	5.78	5.09	4.5	3.88	3.24	2.73	2.1
760	3.24	3.39	4.06	5.02	6.82	6.44	5.75	5.07	4.47	3.86	3.22	2.43	1.53
761	3.24	3.51	4.19	5.14	6.85	6.41	5.72	5.04	4.45	3.83	3.24	2.6	2.2
762	3.24	3.34	4.01	4.96	6.8	6.45	5.77	5.08	4.48	3.87	3.27	2.71	2.11
763	3.24	3.24	3.74	4.51	6.14	6.48	5.85	5.19	4.61	4.02	3.46	2.84	2.32
764	3.24	3.09	3.7	4.41	6.06	6.5	5.88	5.22	4.64	4.04	3.47	2.83	2.21
765	3.24	2.99	3.53	4.28	5.94	6.54	5.93	5.26	4.67	4.09	3.53	2.95	2.41
766	3.24	3.14	3.76	4.5	6.14	6.48	5.85	5.19	4.62	4.01	3.44	2.84	2.21
767	3.24	3.12	3.71	4.49	6.13	6.48	5.85	5.19	4.6	4.01	3.44	2.91	2.24
768	3.24	2.82	3.4	4.2	5.89	6.55	5.95	5.28	4.71	4.13	3.53	2.95	2.43
769	3.24	3.43	4.13	5.07	6.8	6.35	5.58	4.79	4.1	3.4	2.75	2.14	0
770	3.24	3.41	4.07	5	6.78	6.37	5.6	4.81	4.15	3.48	2.86	2.25	1.83
771	3.24	3.34	4	4.93	6.75	6.39	5.61	4.84	4.15	3.49	2.93	1.92	1.41
772	3.24	3.27	3.92	4.86	6.72	6.41	5.65	4.87	4.18	3.53	2.88	2.17	0.7
773	3.24	3.47	4.14	5.07	6.8	6.35	5.57	4.8	4.12	3.48	2.77	2.07	1.43
774	3.24	3.42	4.1	5.04	6.8	6.36	5.58	4.8	4.12	3.42	2.79	1.74	1.4
775	3.24	3.69	4.75	6.3	7.1	7.04	6.98	6.92	6.87	6.82	6.77	6.72	6.67
776	3.24	3.66	4.73	6.3	7.1	7.04	6.98	6.92	6.87	6.82	6.77	6.72	6.67
777	3.24	3.66	4.78	6.34	7.1	7.04	6.98	6.92	6.87	6.82	6.77	6.72	6.67
778	3.24	3.68	4.76	6.32	7.1	7.04	6.98	6.92	6.87	6.82	6.77	6.72	6.67
779	3.24	3.66	4.73	6.3	7.1	7.04	6.98	6.92	6.87	6.82	6.77	6.72	6.67
780	3.24	3.7	4.79	6.35	7.1	7.04	6.98	6.92	6.87	6.82	6.77	6.72	6.67
781	3.24	3.95	5.16	6.84	7.43	7.36	7.27	7.17	7.09	7.01	6.92	6.84	6.76
782	3.24	3.91	5.11	6.79	7.43	7.36	7.27	7.17	7.09	7.01	6.92	6.84	6.76
783	3.24	3.94	5.14	6.82	7.43	7.36	7.27	7.17	7.09	7.01	6.92	6.84	6.76
784	3.24	4.03	5.24	6.89	7.43	7.36	7.26	7.17	7.09	7	6.92	6.84	6.76
785	3.24	3.93	5.14	6.83	7.43	7.36	7.27	7.17	7.09	7.01	6.92	6.84	6.76
786	3.24	3.98	5.2	6.86	7.43	7.36	7.27	7.17	7.09	7.01	6.92	6.84	6.76
787	3.24	3.59	4.67	6.23	7.07	6.98	6.89	6.79	6.71	6.63	6.54	6.46	6.38
788	3.24	3.73	4.81	6.36	7.07	6.98	6.88	6.79	6.71	6.62	6.54	6.46	6.38
789	3.24	3.69	4.75	6.3	7.07	6.98	6.88	6.79	6.71	6.63	6.54	6.46	6.38
790	3.24	3.7	4.77	6.32	7.07	6.98	6.88	6.79	6.71	6.62	6.54	6.46	6.38
791	3.24	3.76	4.84	6.39	7.07	6.98	6.88	6.79	6.71	6.62	6.54	6.46	6.38
792	3.24	3.74	4.79	6.34	7.07	6.98	6.88	6.79	6.71	6.62	6.54	6.46	6.38
793	3.24	3.47	4.45	5.84	6.88	6.79	6.69	6.6	6.51	6.43	6.35	6.27	6.18
794	3.24	3.39	4.39	5.79	6.88	6.79	6.69	6.6	6.52	6.44	6.35	6.27	6.19
795	3.24	3.35	4.3	5.7	6.88	6.79	6.7	6.6	6.52	6.44	6.36	6.27	6.19
796	3.24	3.4	4.38	5.78	6.88	6.79	6.69	6.6	6.52	6.43	6.35	6.27	6.19
797	3.24	3.48	4.41	5.8	6.88	6.79	6.69	6.6	6.52	6.44	6.35	6.27	6.19
798	3.24	3.42	4.37	5.78	6.88	6.79	6.69	6.6	6.52	6.44	6.35	6.27	6.19
799	3.24	4.07	5.31	6.99	7.58	7.52	7.37	7.2	7.05	6.89	6.74	6.58	6.42
800	3.24	4.01	5.24	6.95	7.58	7.52	7.38	7.21	7.05	6.9	6.74	6.59	6.43
801	3.24	4.03	5.28	6.98	7.58	7.52	7.37	7.2	7.05	6.9	6.74	6.58	6.43

802	3.24	4.02	5.26	6.96	7.58	7.52	7.37	7.2	7.05	6.9	6.74	6.59	6.43
803	3.24	4.05	5.3	6.99	7.58	7.52	7.37	7.2	7.05	6.89	6.74	6.58	6.43
804	3.24	3.99	5.22	6.94	7.58	7.52	7.38	7.21	7.05	6.9	6.75	6.59	6.44
805	3.24	3.93	5.12	6.79	7.37	7.23	7.05	6.87	6.72	6.56	6.4	6.25	6.09
806	3.24	3.94	5.15	6.82	7.37	7.23	7.05	6.87	6.72	6.56	6.41	6.25	6.09
807	3.24	3.92	5.13	6.8	7.37	7.23	7.05	6.87	6.72	6.56	6.41	6.25	6.1
808	3.24	3.96	5.15	6.81	7.37	7.23	7.05	6.87	6.72	6.56	6.41	6.25	6.1
809	3.24	3.91	5.11	6.78	7.37	7.23	7.05	6.87	6.72	6.56	6.41	6.26	6.1
810	3.24	4	5.18	6.84	7.37	7.23	7.05	6.87	6.72	6.56	6.4	6.25	6.09
811	3.24	3.78	4.87	6.4	7.01	6.83	6.66	6.48	6.32	6.17	6.01	5.86	5.7
812	3.24	3.71	4.75	6.29	7.02	6.84	6.66	6.48	6.32	6.17	6.01	5.85	5.7
813	3.24	3.76	4.83	6.36	7.01	6.84	6.66	6.48	6.32	6.17	6.01	5.86	5.7
814	3.24	3.74	4.82	6.36	7.01	6.83	6.66	6.48	6.32	6.16	6.01	5.85	5.7
815	3.24	3.69	4.76	6.29	7.02	6.84	6.66	6.48	6.33	6.17	6.01	5.86	5.71
816	3.24	3.71	4.77	6.3	7.02	6.84	6.66	6.48	6.32	6.17	6.01	5.85	5.7
817	3.24	3.37	4.33	5.72	6.84	6.66	6.48	6.3	6.15	5.99	5.83	5.68	5.52
818	3.24	3.51	4.45	5.83	6.83	6.65	6.48	6.3	6.14	5.99	5.83	5.67	5.52
819	3.24	3.37	4.34	5.72	6.84	6.66	6.48	6.3	6.15	5.99	5.83	5.68	5.52
820	3.24	3.32	4.29	5.68	6.84	6.66	6.49	6.31	6.15	6	5.84	5.68	5.53
821	3.24	3.36	4.36	5.75	6.84	6.66	6.48	6.3	6.15	5.99	5.83	5.68	5.52
822	3.24	3.33	4.28	5.66	6.84	6.66	6.49	6.31	6.15	6	5.84	5.69	5.53
823	3.24	3.2	4.11	5.43	6.8	6.62	6.45	6.27	6.11	5.96	5.8	5.65	5.49
824	3.24	3.33	4.24	5.57	6.79	6.62	6.44	6.26	6.1	5.95	5.79	5.64	5.48
825	3.24	3.3	4.28	5.61	6.79	6.61	6.44	6.26	6.1	5.95	5.79	5.64	5.48
826	3.24	3.41	4.33	5.66	6.79	6.61	6.43	6.26	6.1	5.94	5.79	5.63	5.47
827	3.24	3.3	4.24	5.58	6.79	6.62	6.44	6.26	6.1	5.95	5.79	5.63	5.48
828	3.24	3.22	4.12	5.46	6.8	6.62	6.44	6.27	6.11	5.96	5.8	5.64	5.49
829	3.24	3.7	4.74	6.26	6.95	6.67	6.39	6.11	5.86	5.62	5.37	5.13	4.88
830	3.24	3.65	4.71	6.23	6.95	6.67	6.39	6.11	5.87	5.62	5.38	5.15	4.9
831	3.24	3.73	4.8	6.32	6.95	6.67	6.39	6.1	5.86	5.61	5.37	5.12	4.88
832	3.24	3.67	4.73	6.26	6.95	6.67	6.39	6.11	5.87	5.62	5.38	5.14	4.89
833	3.24	3.68	4.74	6.27	6.95	6.67	6.39	6.11	5.87	5.62	5.38	5.13	4.88
834	3.24	3.66	4.74	6.26	6.95	6.67	6.39	6.11	5.87	5.62	5.38	5.14	4.9
835	3.24	4.07	5.33	7.01	7.57	7.45	7.2	6.9	6.61	6.32	6.01	5.69	5.36
836	3.24	4.09	5.33	7.01	7.57	7.44	7.2	6.9	6.61	6.32	6.01	5.69	5.36
837	3.24	4.09	5.33	7.01	7.57	7.45	7.2	6.9	6.61	6.31	6	5.69	5.36
838	3.24	4.07	5.3	6.99	7.57	7.45	7.2	6.9	6.61	6.32	6.01	5.69	5.37
839	3.24	4.07	5.31	7	7.57	7.45	7.2	6.9	6.61	6.31	6	5.69	5.36
840	3.24	4.07	5.31	7	7.57	7.45	7.2	6.9	6.62	6.32	6.01	5.69	5.37
841	3.24	4.01	5.23	6.91	7.45	7.21	6.86	6.47	6.11	5.74	5.37	5.01	4.63
842	3.24	4.02	5.24	6.92	7.45	7.21	6.86	6.47	6.11	5.74	5.38	5	4.62
843	3.24	4.03	5.24	6.92	7.45	7.21	6.86	6.47	6.11	5.74	5.37	5	4.63
844	3.24	4	5.22	6.91	7.45	7.21	6.86	6.47	6.11	5.74	5.38	5.01	4.65
845	3.24	4.06	5.27	6.94	7.45	7.21	6.86	6.47	6.11	5.74	5.38	5.01	4.64
846	3.24	3.99	5.22	6.9	7.45	7.22	6.86	6.47	6.11	5.74	5.38	5.01	4.65
847	3.24	3.99	5.16	6.8	7.22	6.85	6.42	6	5.62	5.25	4.87	4.47	4.11
848	3.24	3.98	5.14	6.78	7.22	6.85	6.43	6	5.62	5.25	4.87	4.51	4.15
849	3.24	3.91	5.08	6.74	7.23	6.85	6.43	6	5.63	5.25	4.87	4.49	4.1
850	3.24	3.98	5.16	6.79	7.22	6.85	6.42	6	5.63	5.26	4.87	4.49	4.12
851	3.24	3.91	5.06	6.72	7.23	6.86	6.43	6.01	5.64	5.26	4.88	4.51	4.14
852	3.24	3.94	5.12	6.76	7.22	6.85	6.43	6	5.62	5.25	4.86	4.48	4.06
853	3.24	3.85	4.98	6.57	7.03	6.61	6.18	5.76	5.38	5	4.63	4.26	3.91
854	3.24	3.94	5.06	6.63	7.02	6.6	6.17	5.74	5.37	5	4.63	4.24	3.88
855	3.24	3.81	4.93	6.52	7.03	6.62	6.19	5.76	5.39	5.02	4.64	4.27	3.91

856	3.24	3.89	5	6.58	7.03	6.61	6.18	5.75	5.38	5.01	4.65	4.29	3.9
857	3.24	3.87	4.97	6.56	7.03	6.61	6.18	5.75	5.38	5.01	4.64	4.26	3.91
858	3.24	3.8	4.9	6.5	7.03	6.62	6.19	5.76	5.38	5.01	4.61	4.21	3.8
859	3.24	3.71	4.76	6.25	6.85	6.43	6	5.57	5.19	4.82	4.44	4.08	3.68
860	3.24	3.62	4.66	6.17	6.86	6.44	6.01	5.58	5.2	4.83	4.46	4.08	3.68
861	3.24	3.7	4.75	6.25	6.86	6.43	6	5.57	5.2	4.82	4.45	4.06	3.71
862	3.24	3.66	4.69	6.2	6.86	6.43	6.01	5.58	5.2	4.83	4.45	4.07	3.67
863	3.24	3.68	4.74	6.24	6.85	6.43	6	5.57	5.2	4.82	4.43	4.07	3.72
864	3.24	3.72	4.76	6.26	6.86	6.43	6	5.57	5.2	4.82	4.45	4.08	3.73
865	3.24	3.55	4.53	5.94	6.75	6.32	5.9	5.47	5.1	4.73	4.37	3.98	3.6
866	3.24	3.5	4.47	5.88	6.76	6.33	5.9	5.47	5.09	4.71	4.32	3.93	3.52
867	3.24	3.47	4.45	5.85	6.76	6.33	5.9	5.47	5.1	4.73	4.35	3.96	3.56
868	3.24	3.51	4.47	5.87	6.76	6.33	5.9	5.47	5.1	4.72	4.34	3.98	3.62
869	3.24	3.46	4.43	5.85	6.76	6.33	5.91	5.48	5.1	4.72	4.36	3.99	3.6
870	3.24	3.55	4.54	5.95	6.75	6.32	5.89	5.46	5.09	4.72	4.34	3.97	3.59
871	3.24	3.31	4.23	5.58	6.72	6.29	5.86	5.44	5.06	4.68	4.31	3.95	3.56
872	3.24	3.38	4.31	5.64	6.71	6.28	5.85	5.43	5.06	4.68	4.31	3.93	3.6
873	3.24	3.37	4.3	5.66	6.71	6.28	5.85	5.42	5.06	4.68	4.3	3.93	3.52
874	3.24	3.44	4.37	5.72	6.7	6.27	5.85	5.42	5.04	4.66	4.28	3.92	3.57
875	3.24	3.42	4.36	5.69	6.7	6.28	5.85	5.42	5.05	4.69	4.31	3.92	3.52
876	3.24	3.42	4.35	5.69	6.71	6.28	5.85	5.42	5.05	4.68	4.3	3.94	3.56
877	3.24	3.28	4.19	5.49	6.68	6.25	5.82	5.4	5.02	4.64	4.26	3.89	3.56
878	3.24	3.25	4.14	5.42	6.69	6.26	5.83	5.4	5.02	4.65	4.27	3.9	3.55
879	3.24	3.18	4.09	5.38	6.69	6.27	5.84	5.41	5.03	4.66	4.28	3.91	3.54
880	3.24	3.29	4.2	5.47	6.68	6.25	5.82	5.39	5.02	4.64	4.27	3.89	3.53
881	3.24	3.17	4.07	5.36	6.7	6.27	5.84	5.41	5.03	4.64	4.26	3.88	3.53
882	3.24	3.25	4.12	5.4	6.69	6.26	5.83	5.41	5.03	4.66	4.29	3.92	3.5
883	3.24	3.21	4.03	5.29	6.68	6.26	5.83	5.4	5.03	4.66	4.3	3.94	3.6
884	3.24	3.28	4.18	5.46	6.66	6.24	5.81	5.38	5	4.64	4.27	3.92	3.55
885	3.24	3.2	4.1	5.37	6.67	6.25	5.82	5.39	5.02	4.65	4.28	3.89	3.49
886	3.24	3.36	4.28	5.53	6.65	6.23	5.8	5.37	4.99	4.63	4.26	3.89	3.5
887	3.24	3.23	4.13	5.38	6.67	6.24	5.82	5.38	5.01	4.63	4.25	3.89	3.49
888	3.24	3.16	3.97	5.23	6.69	6.26	5.84	5.41	5.04	4.66	4.28	3.9	3.51
889	3.24	3.69	4.73	6.21	6.76	6.19	5.61	5.02	4.52	4.04	3.57	3.07	2.57
890	3.24	3.68	4.73	6.22	6.76	6.19	5.61	5.02	4.53	4.02	3.54	3.06	2.54
891	3.24	3.69	4.73	6.21	6.76	6.19	5.6	5.02	4.51	3.99	3.48	3.03	2.42
892	3.24	3.61	4.61	6.1	6.78	6.2	5.62	5.05	4.55	4.02	3.56	3.04	2.55
893	3.24	3.63	4.66	6.15	6.77	6.19	5.62	5.04	4.52	4.04	3.52	3.03	2.61
894	3.24	3.72	4.77	6.25	6.76	6.18	5.6	5.02	4.51	3.98	3.45	2.88	2.52
895	3.24	3.98	5.16	6.83	7.33	6.95	6.44	5.87	5.36	4.83	4.29	3.72	3.15
896	3.24	4.04	5.24	6.89	7.32	6.94	6.43	5.86	5.34	4.81	4.27	3.71	3.14
897	3.24	4.01	5.21	6.86	7.32	6.94	6.43	5.87	5.35	4.81	4.26	3.7	3.14
898	3.24	4.04	5.26	6.9	7.32	6.94	6.43	5.86	5.35	4.81	4.24	3.67	3.04
899	3.24	4	5.22	6.87	7.32	6.94	6.43	5.86	5.34	4.82	4.26	3.69	3.08
900	3.24	4.01	5.22	6.87	7.32	6.94	6.43	5.87	5.35	4.81	4.28	3.73	3.18
901	3.24	3.94	5.08	6.7	7.08	6.49	5.82	5.14	4.54	3.93	3.37	2.75	1.7
902	3.24	3.88	5.03	6.66	7.09	6.5	5.83	5.14	4.54	3.92	3.28	2.56	2.18
903	3.24	3.92	5.07	6.69	7.08	6.49	5.82	5.13	4.54	3.98	3.46	2.98	2.37
904	3.24	3.97	5.11	6.72	7.08	6.49	5.82	5.14	4.53	3.93	3.29	2.72	2.03
905	3.24	3.91	5.05	6.68	7.08	6.5	5.82	5.13	4.53	3.91	3.28	2.68	1.7
906	3.24	3.9	5.06	6.68	7.08	6.5	5.83	5.14	4.53	3.93	3.35	2.85	2.04
907	3.24	3.74	4.74	6.2	6.7	6.01	5.33	4.64	4.03	3.41	2.82	2.25	1.62
908	3.24	3.58	4.62	6.1	6.72	6.03	5.34	4.66	4.03	3.45	2.85	2.21	1.36
909	3.24	3.7	4.73	6.19	6.7	6.01	5.33	4.64	4.06	3.45	2.79	2.2	1.46

910	3.24	3.78	4.76	6.22	6.69	6.01	5.32	4.64	4.04	3.44	2.95	2.52	2.05
911	3.24	3.65	4.7	6.16	6.71	6.02	5.34	4.64	4.04	3.37	2.85	2.34	1.58
912	3.24	3.64	4.64	6.12	6.71	6.03	5.34	4.64	4.05	3.42	2.79	2.4	1.54
913	3.24	3.49	4.36	5.66	6.58	5.89	5.2	4.52	3.93	3.29	2.68	2	1.43
914	3.24	3.37	4.26	5.55	6.6	5.92	5.23	4.54	3.94	3.34	2.7	2.01	1.46
915	3.24	3.38	4.27	5.56	6.6	5.91	5.23	4.53	3.94	3.26	2.67	1.9	1.46
916	3.24	3.27	4.15	5.45	6.62	5.94	5.25	4.55	3.94	3.35	2.69	2.15	1.75
917	3.24	3.32	4.21	5.51	6.61	5.92	5.24	4.56	3.97	3.37	2.71	2.16	1.52
918	3.24	3.41	4.27	5.56	6.6	5.91	5.23	4.54	3.93	3.35	2.79	2.03	0.78
919	3.24	3.32	4.19	5.42	6.58	5.9	5.21	4.53	3.93	3.32	2.62	1.81	1.11
920	3.24	3.25	4.07	5.3	6.6	5.92	5.23	4.54	3.96	3.34	2.71	2.15	1.57
921	3.24	3.26	4.1	5.34	6.6	5.92	5.22	4.53	3.91	3.34	2.72	2.05	0.3
922	3.24	3.3	4.11	5.33	6.6	5.92	5.23	4.54	3.95	3.38	2.81	2.16	1.74
923	3.24	3.12	3.94	5.2	6.62	5.94	5.26	4.57	3.96	3.34	2.81	2.26	1.61
924	3.24	3.22	4.13	5.37	6.59	5.91	5.23	4.54	3.95	3.3	2.6	1.98	0.3
925	3.24	3.94	5.09	6.7	7.03	6.37	5.62	4.84	4.13	3.44	2.75	2.41	1.2
926	3.24	3.87	5.03	6.66	7.04	6.38	5.63	4.86	4.14	3.42	2.63	1.67	0.48
927	3.24	3.98	5.11	6.71	7.03	6.37	5.62	4.85	4.19	3.51	2.89	2.44	2.25
928	3.24	3.93	5.07	6.68	7.04	6.37	5.63	4.87	4.21	3.58	2.83	2.27	1.43
929	3.24	3.86	4.97	6.6	7.05	6.39	5.63	4.85	4.16	3.47	2.87	2.21	1.26
930	3.24	3.91	5.06	6.68	7.04	6.37	5.62	4.85	4.19	3.51	2.76	2.32	1.83
931	3.24	3.64	4.64	6.09	6.67	5.89	5.11	4.32	3.62	2.97	2.23	1.51	0.3
932	3.24	3.68	4.68	6.14	6.66	5.88	5.1	4.32	3.67	3.08	2.47	1.88	1.26
933	3.24	3.65	4.65	6.1	6.67	5.89	5.11	4.32	3.62	2.92	2.25	0	0
934	3.24	3.71	4.74	6.19	6.65	5.87	5.1	4.32	3.62	2.97	2.28	1.2	0
935	3.24	3.6	4.61	6.07	6.67	5.9	5.12	4.3	3.68	3.03	2.25	1.67	0
936	3.24	3.68	4.73	6.17	6.65	5.87	5.09	4.32	3.64	2.92	2.34	1.56	0
937	3.24	3.45	4.34	5.62	6.55	5.77	4.99	4.22	3.54	2.84	2.17	1.2	0
938	3.24	3.29	4.17	5.44	6.59	5.82	5.04	4.26	3.55	2.72	2.06	1.68	1.11
939	3.24	3.35	4.22	5.5	6.58	5.8	5.02	4.24	3.55	2.84	2.13	1.46	0
940	3.24	3.31	4.22	5.48	6.58	5.8	5.03	4.25	3.59	2.9	2.4	1.49	1.11
941	3.24	3.39	4.27	5.56	6.56	5.79	5.01	4.24	3.58	2.92	2.13	1.53	0
942	3.24	3.37	4.24	5.51	6.57	5.8	5.02	4.25	3.61	2.88	2.16	1.15	0
943	3.24	3.7	4.71	6.15	6.63	5.81	4.99	4.18	3.46	2.78	1.86	0	0
944	3.24	3.61	4.58	6.03	6.66	5.84	5.02	4.2	3.51	2.83	2.16	1.67	0.3
945	3.24	3.72	4.71	6.16	6.63	5.81	5	4.2	3.52	2.79	2.09	1.32	1.32
946	3.24	3.77	4.79	6.23	6.62	5.79	4.96	4.12	3.44	2.71	1.95	0	0
947	3.24	3.69	4.7	6.14	6.63	5.82	5.01	4.19	3.47	2.84	2.19	1.66	1.36
948	3.24	3.65	4.66	6.12	6.64	5.82	5	4.16	3.41	2.74	2.13	1.4	1.45
949	3.24	3.72	4.82	6.4	6.83	6.41	5.98	5.55	5.17	4.8	4.4	4.03	3.61
950	3.24	3.71	4.8	6.38	6.84	6.41	5.98	5.55	5.18	4.79	4.42	4.03	3.67
951	3.24	3.81	4.91	6.47	6.82	6.4	5.97	5.55	5.17	4.79	4.43	4.03	3.67
952	3.24	3.7	4.82	6.4	6.83	6.41	5.98	5.55	5.18	4.8	4.42	4.02	3.64
953	3.24	3.83	4.93	6.48	6.82	6.4	5.97	5.54	5.17	4.8	4.42	4.05	3.66
954	3.24	3.77	4.85	6.42	6.83	6.4	5.98	5.55	5.18	4.79	4.41	4.04	3.68
955	3.24	3.9	5.29	6.98	6.98	6.8	6.62	6.45	6.29	6.13	5.98	5.82	5.67
956	3.24	3.93	5.32	6.98	6.98	6.8	6.62	6.44	6.29	6.13	5.98	5.82	5.67
957	3.24	3.93	5.34	6.99	6.98	6.8	6.62	6.44	6.29	6.13	5.98	5.82	5.67
958	3.24	4.03	5.42	7	6.97	6.8	6.62	6.44	6.28	6.13	5.97	5.82	5.67
959	3.24	4	5.39	7	6.97	6.8	6.62	6.44	6.28	6.13	5.97	5.82	5.66
960	3.24	3.89	5.28	6.97	6.98	6.8	6.62	6.44	6.29	6.13	5.98	5.82	5.66
961	3.24	4.15	5.64	7.18	7.17	6.78	6.35	5.92	5.55	5.18	4.82	4.44	4.05
962	3.24	4.16	5.66	7.18	7.17	6.77	6.35	5.92	5.54	5.16	4.79	4.43	4.05
963	3.24	4.2	5.67	7.18	7.17	6.77	6.35	5.92	5.54	5.16	4.78	4.4	4

964	3.24	4.25	5.74	7.2	7.16	6.77	6.34	5.91	5.54	5.17	4.79	4.41	4.04
965	3.24	4.23	5.72	7.19	7.16	6.77	6.35	5.92	5.55	5.17	4.78	4.39	4
966	3.24	4.24	5.73	7.19	7.16	6.77	6.34	5.92	5.54	5.16	4.78	4.4	4.03
967	3.24	4.01	5.38	6.97	6.75	6.32	5.89	5.46	5.08	4.7	4.32	3.95	3.57
968	3.24	3.98	5.34	6.96	6.75	6.32	5.89	5.47	5.1	4.72	4.35	3.96	3.62
969	3.24	3.93	5.29	6.95	6.76	6.33	5.9	5.47	5.09	4.71	4.34	3.96	3.57
970	3.24	3.9	5.26	6.94	6.76	6.33	5.9	5.48	5.11	4.73	4.35	3.98	3.61
971	3.24	3.91	5.28	6.95	6.76	6.33	5.9	5.47	5.09	4.72	4.33	3.96	3.55
972	3.24	3.92	5.29	6.95	6.76	6.33	5.9	5.47	5.1	4.72	4.35	3.96	3.53
973	3.24	3.72	4.97	6.65	6.57	6.14	5.71	5.29	4.91	4.56	4.19	3.81	3.43
974	3.24	3.6	4.89	6.6	6.58	6.15	5.72	5.3	4.92	4.55	4.17	3.79	3.44
975	3.24	3.62	4.86	6.58	6.58	6.15	5.72	5.29	4.92	4.54	4.13	3.76	3.41
976	3.24	3.61	4.88	6.59	6.58	6.15	5.71	5.28	4.9	4.54	4.16	3.78	3.42
977	3.24	3.7	4.92	6.62	6.57	6.14	5.72	5.29	4.91	4.54	4.17	3.79	3.41
978	3.24	3.6	4.83	6.56	6.58	6.15	5.72	5.29	4.92	4.54	4.2	3.83	3.45
979	3.24	4	5.34	6.93	6.53	5.84	5.15	4.45	3.85	3.26	2.6	2.13	1.11
980	3.24	3.97	5.32	6.93	6.53	5.84	5.15	4.47	3.85	3.3	2.73	1.95	1.7
981	3.24	3.95	5.3	6.92	6.53	5.84	5.16	4.47	3.84	3.26	2.66	2.11	1.43
982	3.24	3.9	5.25	6.91	6.54	5.85	5.16	4.49	3.88	3.24	2.67	2.13	1.66
983	3.24	3.9	5.22	6.9	6.54	5.85	5.16	4.48	3.88	3.25	2.44	1.86	1.18
984	3.24	3.97	5.31	6.93	6.53	5.84	5.16	4.48	3.86	3.22	2.7	2.16	1.68
985	3.24	4.24	6	7.1	7.1	7.06	7.02	6.98	6.94	6.91	6.88	6.84	6.81
986	3.24	4.26	6.01	7.1	7.1	7.06	7.02	6.98	6.94	6.91	6.87	6.84	6.81
987	3.24	4.23	5.98	7.1	7.1	7.06	7.02	6.98	6.94	6.91	6.87	6.84	6.81
988	3.24	4.31	6.06	7.1	7.09	7.06	7.02	6.98	6.94	6.91	6.87	6.84	6.81
989	3.24	4.29	6.05	7.1	7.1	7.06	7.02	6.98	6.94	6.91	6.87	6.84	6.81
990	3.24	4.27	6.02	7.1	7.1	7.06	7.02	6.98	6.94	6.91	6.88	6.84	6.81
991	3.24	4.49	6.34	7.34	7.45	7.4	7.34	7.29	7.23	7.18	7.13	7.08	7.03
992	3.24	4.49	6.33	7.34	7.45	7.4	7.34	7.28	7.23	7.18	7.13	7.08	7.03
993	3.24	4.49	6.33	7.34	7.45	7.4	7.34	7.28	7.23	7.18	7.13	7.08	7.03
994	3.24	4.46	6.3	7.34	7.45	7.4	7.34	7.28	7.23	7.18	7.13	7.08	7.03
995	3.24	4.49	6.33	7.34	7.45	7.4	7.34	7.29	7.23	7.18	7.13	7.08	7.03
996	3.24	4.45	6.29	7.34	7.45	7.4	7.34	7.29	7.23	7.18	7.13	7.08	7.03
997	3.24	4.24	5.98	7.1	7.08	7.02	6.96	6.9	6.85	6.8	6.75	6.7	6.65
998	3.24	4.28	6.04	7.1	7.08	7.02	6.96	6.9	6.85	6.8	6.75	6.7	6.65
999	3.24	4.29	6.03	7.1	7.08	7.02	6.96	6.9	6.85	6.8	6.75	6.7	6.65
1000	3.24	4.32	6.06	7.1	7.07	7.02	6.96	6.9	6.85	6.8	6.75	6.7	6.65
1001	3.24	4.33	6.08	7.1	7.08	7.02	6.96	6.9	6.85	6.8	6.75	6.7	6.65
1002	3.24	4.36	6.11	7.1	7.08	7.02	6.96	6.9	6.85	6.8	6.75	6.7	6.65
1003	3.24	3.94	5.57	6.92	6.88	6.82	6.76	6.7	6.65	6.6	6.55	6.5	6.45
1004	3.24	3.92	5.58	6.92	6.88	6.82	6.76	6.7	6.65	6.6	6.55	6.5	6.45
1005	3.24	3.91	5.57	6.92	6.88	6.82	6.76	6.7	6.65	6.6	6.55	6.5	6.45
1006	3.24	4.01	5.65	6.92	6.88	6.82	6.76	6.7	6.65	6.6	6.55	6.5	6.45
1007	3.24	3.94	5.59	6.92	6.88	6.82	6.76	6.7	6.65	6.6	6.55	6.5	6.45
1008	3.24	3.97	5.63	6.92	6.88	6.82	6.76	6.7	6.65	6.6	6.55	6.5	6.45
1009	3.24	4.54	6.42	7.43	7.65	7.62	7.54	7.45	7.36	7.28	7.2	7.12	7.03
1010	3.24	4.56	6.43	7.43	7.65	7.62	7.54	7.45	7.37	7.28	7.2	7.12	7.04
1011	3.24	4.55	6.42	7.43	7.65	7.62	7.54	7.45	7.36	7.28	7.2	7.12	7.03
1012	3.24	4.55	6.42	7.43	7.65	7.62	7.54	7.45	7.37	7.28	7.2	7.12	7.04
1013	3.24	4.57	6.44	7.43	7.65	7.62	7.54	7.45	7.36	7.28	7.2	7.12	7.04
1014	3.24	4.57	6.44	7.43	7.65	7.62	7.54	7.45	7.36	7.28	7.2	7.12	7.04
1015	3.24	4.5	6.33	7.33	7.42	7.33	7.24	7.14	7.06	6.98	6.9	6.81	6.73
1016	3.24	4.45	6.29	7.33	7.42	7.33	7.24	7.14	7.06	6.98	6.9	6.81	6.73
1017	3.24	4.46	6.3	7.33	7.42	7.33	7.24	7.14	7.06	6.98	6.9	6.81	6.73

1018	3.24	4.52	6.35	7.33	7.42	7.33	7.24	7.14	7.06	6.98	6.9	6.81	6.73
1019	3.24	4.45	6.29	7.33	7.42	7.33	7.24	7.14	7.06	6.98	6.9	6.82	6.73
1020	3.24	4.46	6.3	7.33	7.42	7.33	7.24	7.14	7.06	6.98	6.9	6.81	6.73
1021	3.24	4.3	6.04	7.09	7.04	6.94	6.85	6.75	6.67	6.59	6.51	6.42	6.34
1022	3.24	4.26	6	7.09	7.04	6.94	6.85	6.75	6.67	6.59	6.51	6.42	6.34
1023	3.24	4.3	6.04	7.09	7.04	6.94	6.85	6.75	6.67	6.59	6.51	6.42	6.34
1024	3.24	4.35	6.08	7.09	7.04	6.94	6.85	6.75	6.67	6.59	6.5	6.42	6.34
1025	3.24	4.26	5.99	7.09	7.04	6.94	6.85	6.75	6.67	6.59	6.51	6.42	6.34
1026	3.24	4.33	6.07	7.09	7.04	6.94	6.85	6.75	6.67	6.59	6.51	6.42	6.34
1027	3.24	3.86	5.52	6.92	6.84	6.75	6.65	6.56	6.48	6.39	6.31	6.23	6.15
1028	3.24	4.05	5.7	6.92	6.84	6.74	6.65	6.55	6.47	6.39	6.31	6.22	6.14
1029	3.24	3.98	5.62	6.92	6.84	6.75	6.65	6.56	6.47	6.39	6.31	6.23	6.14
1030	3.24	3.95	5.62	6.92	6.84	6.74	6.65	6.56	6.47	6.39	6.31	6.22	6.14
1031	3.24	3.92	5.57	6.92	6.84	6.75	6.65	6.56	6.47	6.39	6.31	6.23	6.14
1032	3.24	3.99	5.63	6.92	6.84	6.75	6.65	6.56	6.47	6.39	6.31	6.23	6.14
1033	3.24	3.85	5.46	6.87	6.79	6.7	6.6	6.51	6.42	6.34	6.26	6.17	6.09
1034	3.24	3.85	5.48	6.87	6.79	6.7	6.6	6.51	6.42	6.34	6.26	6.18	6.09
1035	3.24	3.93	5.54	6.87	6.79	6.69	6.6	6.51	6.42	6.34	6.26	6.18	6.09
1036	3.24	3.87	5.47	6.87	6.79	6.7	6.6	6.51	6.43	6.34	6.26	6.18	6.09
1037	3.24	3.78	5.39	6.87	6.79	6.7	6.6	6.51	6.42	6.34	6.26	6.18	6.09
1038	3.24	3.86	5.47	6.87	6.79	6.7	6.6	6.51	6.42	6.34	6.26	6.18	6.09
1039	3.24	4.57	6.45	7.46	7.72	7.69	7.58	7.44	7.3	7.15	7	6.85	6.7
1040	3.24	4.57	6.45	7.46	7.72	7.69	7.58	7.44	7.3	7.15	7.01	6.85	6.7
1041	3.24	4.6	6.48	7.47	7.72	7.69	7.58	7.44	7.3	7.15	7	6.85	6.7
1042	3.24	4.61	6.49	7.47	7.72	7.69	7.58	7.43	7.3	7.15	7	6.85	6.7
1043	3.24	4.61	6.49	7.47	7.72	7.69	7.58	7.44	7.3	7.15	7	6.85	6.69
1044	3.24	4.57	6.45	7.46	7.72	7.69	7.58	7.44	7.3	7.15	7	6.85	6.7
1045	3.24	4.57	6.44	7.42	7.58	7.48	7.33	7.15	7	6.85	6.69	6.53	6.38
1046	3.24	4.58	6.45	7.42	7.58	7.48	7.33	7.16	7	6.85	6.69	6.53	6.38
1047	3.24	4.56	6.42	7.41	7.58	7.48	7.33	7.16	7	6.85	6.69	6.54	6.38
1048	3.24	4.53	6.4	7.41	7.58	7.48	7.33	7.16	7	6.85	6.69	6.54	6.38
1049	3.24	4.55	6.42	7.42	7.58	7.48	7.33	7.16	7	6.85	6.69	6.54	6.38
1050	3.24	4.54	6.4	7.41	7.58	7.49	7.33	7.16	7	6.85	6.69	6.53	6.38
1051	3.24	4.49	6.33	7.31	7.34	7.18	7	6.82	6.66	6.51	6.35	6.19	6.04
1052	3.24	4.43	6.27	7.31	7.34	7.18	7	6.82	6.67	6.51	6.35	6.2	6.04
1053	3.24	4.49	6.33	7.31	7.34	7.18	7	6.82	6.66	6.51	6.35	6.19	6.04
1054	3.24	4.48	6.32	7.31	7.34	7.18	7	6.82	6.67	6.51	6.35	6.2	6.04
1055	3.24	4.49	6.33	7.32	7.34	7.17	7	6.82	6.66	6.51	6.35	6.2	6.04
1056	3.24	4.46	6.3	7.31	7.34	7.18	7	6.82	6.67	6.51	6.35	6.2	6.04
1057	3.24	4.42	6.22	7.21	7.14	6.96	6.78	6.6	6.45	6.29	6.14	5.98	5.83
1058	3.24	4.35	6.15	7.2	7.14	6.96	6.78	6.61	6.45	6.29	6.14	5.98	5.83
1059	3.24	4.4	6.19	7.21	7.14	6.96	6.78	6.6	6.45	6.29	6.13	5.98	5.82
1060	3.24	4.39	6.18	7.21	7.14	6.96	6.78	6.6	6.45	6.29	6.14	5.98	5.83
1061	3.24	4.4	6.19	7.21	7.14	6.96	6.78	6.6	6.45	6.3	6.14	5.98	5.83
1062	3.24	4.39	6.19	7.21	7.14	6.96	6.78	6.6	6.45	6.29	6.14	5.98	5.83
1063	3.24	4.26	6.01	7.07	6.95	6.77	6.59	6.42	6.26	6.11	5.95	5.79	5.64
1064	3.24	4.24	5.99	7.07	6.95	6.77	6.59	6.42	6.26	6.1	5.95	5.79	5.64
1065	3.24	4.25	5.99	7.07	6.95	6.77	6.59	6.42	6.26	6.1	5.95	5.79	5.63
1066	3.24	4.23	5.97	7.07	6.95	6.77	6.59	6.42	6.26	6.1	5.95	5.79	5.63
1067	3.24	4.19	5.93	7.07	6.95	6.77	6.59	6.42	6.26	6.1	5.95	5.79	5.64
1068	3.24	4.25	5.98	7.07	6.95	6.77	6.59	6.42	6.26	6.11	5.95	5.79	5.63
1069	3.24	4.13	5.82	6.96	6.82	6.64	6.46	6.29	6.13	5.97	5.82	5.66	5.5
1070	3.24	4.1	5.78	6.96	6.82	6.64	6.47	6.29	6.13	5.97	5.82	5.66	5.51
1071	3.24	4.09	5.78	6.96	6.82	6.64	6.47	6.29	6.13	5.98	5.82	5.67	5.51

1072	3.24	4.17	5.84	6.96	6.82	6.64	6.46	6.28	6.13	5.97	5.82	5.66	5.5
1073	3.24	4.16	5.84	6.96	6.82	6.64	6.46	6.29	6.13	5.98	5.82	5.67	5.51
1074	3.24	4.15	5.84	6.96	6.82	6.64	6.46	6.28	6.13	5.97	5.82	5.66	5.5
1075	3.24	4.09	5.72	6.9	6.75	6.57	6.39	6.22	6.06	5.9	5.75	5.59	5.44
1076	3.24	3.95	5.57	6.9	6.75	6.57	6.4	6.22	6.06	5.9	5.75	5.59	5.44
1077	3.24	4.01	5.66	6.9	6.75	6.57	6.4	6.22	6.06	5.91	5.75	5.59	5.44
1078	3.24	4.01	5.66	6.9	6.75	6.57	6.4	6.22	6.06	5.91	5.75	5.6	5.44
1079	3.24	3.97	5.62	6.9	6.75	6.57	6.4	6.22	6.06	5.91	5.75	5.6	5.44
1080	3.24	4.07	5.71	6.9	6.75	6.57	6.39	6.22	6.06	5.91	5.75	5.59	5.44
1081	3.24	3.88	5.49	6.85	6.7	6.52	6.35	6.17	6.01	5.86	5.7	5.55	5.39
1082	3.24	3.87	5.47	6.85	6.7	6.53	6.35	6.17	6.01	5.86	5.7	5.55	5.39
1083	3.24	3.89	5.5	6.85	6.7	6.52	6.35	6.17	6.01	5.86	5.7	5.55	5.39
1084	3.24	3.92	5.53	6.85	6.7	6.52	6.35	6.17	6.01	5.86	5.7	5.54	5.39
1085	3.24	3.87	5.47	6.85	6.7	6.52	6.35	6.17	6.01	5.86	5.7	5.55	5.39
1086	3.24	3.93	5.56	6.85	6.7	6.52	6.34	6.17	6.01	5.85	5.7	5.55	5.39
1087	3.24	3.95	5.55	6.83	6.68	6.5	6.32	6.15	5.99	5.83	5.68	5.52	5.37
1088	3.24	3.87	5.44	6.83	6.68	6.5	6.33	6.15	5.99	5.84	5.68	5.53	5.37
1089	3.24	3.8	5.38	6.84	6.68	6.51	6.33	6.15	6	5.84	5.68	5.53	5.37
1090	3.24	3.9	5.49	6.83	6.68	6.5	6.32	6.15	5.99	5.83	5.68	5.52	5.37
1091	3.24	3.79	5.37	6.84	6.68	6.51	6.33	6.15	5.99	5.84	5.68	5.53	5.37
1092	3.24	3.8	5.4	6.84	6.68	6.51	6.33	6.15	6	5.84	5.68	5.53	5.37
1093	3.24	4.22	5.96	7.07	6.93	6.74	6.54	6.35	6.18	6.01	5.84	5.67	5.5
1094	3.24	4.21	5.95	7.07	6.93	6.74	6.54	6.35	6.18	6.01	5.84	5.67	5.5
1095	3.24	4.29	6.02	7.07	6.93	6.74	6.54	6.35	6.18	6.01	5.84	5.66	5.49
1096	3.24	4.3	6.05	7.07	6.93	6.74	6.54	6.35	6.18	6.01	5.84	5.66	5.49
1097	3.24	4.21	5.95	7.07	6.93	6.74	6.55	6.35	6.18	6.01	5.84	5.67	5.5
1098	3.24	4.25	5.99	7.07	6.93	6.74	6.54	6.35	6.18	6.01	5.84	5.67	5.5
1099	3.24	4.54	6.37	7.3	7.25	6.99	6.71	6.43	6.18	5.94	5.69	5.45	5.2
1100	3.24	4.49	6.32	7.29	7.25	6.99	6.71	6.43	6.18	5.94	5.69	5.45	5.2
1101	3.24	4.45	6.27	7.29	7.25	6.99	6.71	6.43	6.19	5.94	5.69	5.45	5.2
1102	3.24	4.49	6.31	7.29	7.25	6.99	6.71	6.43	6.19	5.94	5.69	5.44	5.2
1103	3.24	4.47	6.31	7.29	7.25	6.99	6.71	6.43	6.18	5.94	5.69	5.45	5.19
1104	3.24	4.54	6.36	7.3	7.25	6.99	6.71	6.43	6.18	5.94	5.69	5.45	5.2
1105	3.24	4.27	6.01	7.05	6.84	6.56	6.28	6	5.76	5.51	5.27	5.03	4.78
1106	3.24	4.3	6.03	7.05	6.84	6.56	6.28	6	5.76	5.51	5.27	5.02	4.77
1107	3.24	4.33	6.06	7.05	6.84	6.56	6.28	6	5.76	5.51	5.27	5.02	4.78
1108	3.24	4.27	6.01	7.05	6.84	6.56	6.28	6	5.76	5.51	5.26	5.02	4.78
1109	3.24	4.19	5.93	7.05	6.84	6.57	6.29	6.01	5.76	5.52	5.27	5.02	4.78
1110	3.24	4.21	5.95	7.05	6.84	6.56	6.28	6	5.76	5.51	5.27	5.01	4.75
1111	3.24	4.01	5.65	6.88	6.64	6.36	6.08	5.81	5.56	5.32	5.07	4.83	4.58
1112	3.24	4.01	5.64	6.88	6.64	6.36	6.08	5.81	5.56	5.32	5.07	4.83	4.6
1113	3.24	3.98	5.63	6.88	6.65	6.37	6.09	5.81	5.57	5.32	5.07	4.82	4.58
1114	3.24	3.96	5.6	6.88	6.65	6.36	6.08	5.8	5.56	5.32	5.07	4.82	4.57
1115	3.24	4.01	5.63	6.88	6.64	6.36	6.08	5.8	5.56	5.31	5.07	4.82	4.58
1116	3.24	3.99	5.62	6.88	6.65	6.37	6.09	5.81	5.56	5.31	5.07	4.83	4.58
1117	3.24	4.57	6.44	7.44	7.65	7.55	7.36	7.14	6.93	6.7	6.47	6.24	6
1118	3.24	4.58	6.45	7.44	7.64	7.55	7.37	7.14	6.93	6.71	6.48	6.24	6.01
1119	3.24	4.62	6.5	7.45	7.65	7.55	7.36	7.14	6.93	6.71	6.48	6.24	6
1120	3.24	4.57	6.44	7.44	7.65	7.55	7.37	7.14	6.93	6.71	6.48	6.24	6.01
1121	3.24	4.58	6.45	7.44	7.65	7.55	7.37	7.14	6.93	6.71	6.48	6.24	6
1122	3.24	4.59	6.47	7.44	7.65	7.55	7.36	7.14	6.93	6.71	6.48	6.24	6
1123	3.24	4.6	6.46	7.42	7.56	7.39	7.12	6.81	6.52	6.22	5.91	5.59	5.26
1124	3.24	4.52	6.39	7.41	7.56	7.39	7.12	6.81	6.52	6.22	5.91	5.58	5.26
1125	3.24	4.56	6.43	7.42	7.56	7.39	7.12	6.81	6.52	6.22	5.91	5.59	5.27

1126	3.24	4.59	6.45	7.42	7.56	7.39	7.12	6.81	6.52	6.22	5.91	5.59	5.26
1127	3.24	4.58	6.44	7.42	7.56	7.39	7.12	6.81	6.52	6.22	5.9	5.58	5.26
1128	3.24	4.57	6.44	7.42	7.56	7.39	7.12	6.81	6.52	6.22	5.91	5.59	5.27
1129	3.24	4.51	6.36	7.37	7.41	7.12	6.75	6.36	6	5.63	5.26	4.89	4.51
1130	3.24	4.58	6.44	7.37	7.4	7.12	6.75	6.35	5.99	5.62	5.24	4.85	4.48
1131	3.24	4.59	6.43	7.37	7.4	7.12	6.75	6.35	6	5.63	5.26	4.9	4.53
1132	3.24	4.52	6.37	7.37	7.41	7.12	6.75	6.36	5.99	5.63	5.26	4.88	4.51
1133	3.24	4.55	6.4	7.37	7.4	7.12	6.75	6.35	5.99	5.62	5.25	4.87	4.52
1134	3.24	4.56	6.41	7.37	7.4	7.12	6.75	6.35	5.99	5.62	5.25	4.88	4.49
1135	3.24	4.48	6.31	7.27	7.12	6.72	6.29	5.87	5.5	5.12	4.76	4.39	4.01
1136	3.24	4.49	6.31	7.27	7.12	6.72	6.29	5.87	5.49	5.13	4.76	4.39	4.01
1137	3.24	4.49	6.31	7.27	7.12	6.72	6.29	5.87	5.49	5.11	4.74	4.36	3.95
1138	3.24	4.44	6.26	7.26	7.12	6.72	6.3	5.87	5.49	5.11	4.74	4.37	3.96
1139	3.24	4.46	6.29	7.27	7.12	6.72	6.3	5.87	5.5	5.13	4.78	4.39	4.05
1140	3.24	4.48	6.3	7.27	7.12	6.72	6.29	5.87	5.49	5.11	4.72	4.34	3.96
1141	3.24	4.48	6.29	7.25	7.08	6.67	6.24	5.81	5.43	5.05	4.68	4.29	3.9
1142	3.24	4.48	6.29	7.25	7.08	6.67	6.24	5.81	5.43	5.05	4.68	4.31	3.94
1143	3.24	4.46	6.27	7.25	7.08	6.67	6.25	5.82	5.45	5.07	4.7	4.32	3.94
1144	3.24	4.49	6.29	7.25	7.08	6.67	6.24	5.81	5.43	5.05	4.66	4.28	3.89
1145	3.24	4.51	6.32	7.25	7.08	6.67	6.24	5.81	5.44	5.07	4.71	4.35	3.99
1146	3.24	4.47	6.29	7.25	7.08	6.67	6.24	5.81	5.44	5.06	4.69	4.32	3.98
1147	3.24	4.41	6.18	7.16	6.89	6.46	6.03	5.6	5.23	4.84	4.49	4.14	3.73
1148	3.24	4.41	6.2	7.16	6.89	6.47	6.04	5.61	5.23	4.84	4.47	4.11	3.76
1149	3.24	4.39	6.18	7.16	6.89	6.46	6.03	5.61	5.24	4.86	4.49	4.11	3.74
1150	3.24	4.36	6.14	7.16	6.89	6.47	6.04	5.61	5.24	4.88	4.51	4.09	3.7
1151	3.24	4.41	6.18	7.16	6.89	6.47	6.04	5.61	5.24	4.87	4.49	4.12	3.78
1152	3.24	4.42	6.2	7.16	6.89	6.46	6.03	5.61	5.23	4.86	4.49	4.12	3.71
1153	3.24	4.31	6.04	7.02	6.69	6.26	5.83	5.39	5.02	4.64	4.27	3.87	3.47
1154	3.24	4.28	6.02	7.02	6.68	6.25	5.82	5.4	5.02	4.66	4.3	3.92	3.54
1155	3.24	4.24	5.97	7.02	6.69	6.26	5.83	5.41	5.03	4.65	4.27	3.89	3.51
1156	3.24	4.25	5.98	7.02	6.69	6.26	5.83	5.4	5.02	4.64	4.26	3.88	3.51
1157	3.24	4.26	5.98	7.02	6.69	6.26	5.83	5.4	5.03	4.66	4.27	3.87	3.51
1158	3.24	4.2	5.93	7.02	6.69	6.27	5.84	5.41	5.04	4.67	4.29	3.93	3.56
1159	3.24	4.11	5.77	6.92	6.56	6.13	5.7	5.27	4.89	4.51	4.15	3.74	3.34
1160	3.24	4.06	5.72	6.92	6.56	6.13	5.7	5.27	4.9	4.54	4.17	3.8	3.42
1161	3.24	4.1	5.77	6.92	6.56	6.13	5.7	5.28	4.9	4.53	4.15	3.77	3.31
1162	3.24	4.08	5.75	6.92	6.56	6.13	5.7	5.27	4.91	4.52	4.13	3.74	3.33
1163	3.24	4.09	5.76	6.92	6.56	6.13	5.7	5.28	4.92	4.53	4.15	3.79	3.4
1164	3.24	4.03	5.69	6.92	6.56	6.13	5.7	5.28	4.9	4.53	4.15	3.79	3.36
1165	3.24	3.93	5.54	6.87	6.51	6.08	5.65	5.22	4.84	4.45	4.07	3.69	3.26
1166	3.24	3.97	5.61	6.87	6.5	6.07	5.64	5.21	4.84	4.45	4.07	3.69	3.31
1167	3.24	4.01	5.63	6.87	6.5	6.07	5.64	5.22	4.84	4.47	4.1	3.73	3.37
1168	3.24	3.99	5.63	6.87	6.5	6.07	5.64	5.21	4.84	4.45	4.07	3.68	3.34
1169	3.24	3.99	5.61	6.87	6.5	6.07	5.65	5.22	4.85	4.47	4.12	3.75	3.38
1170	3.24	3.99	5.62	6.87	6.5	6.07	5.64	5.22	4.84	4.46	4.09	3.73	3.38
1171	3.24	3.91	5.54	6.86	6.49	6.07	5.64	5.2	4.82	4.44	4.06	3.69	3.32
1172	3.24	3.92	5.55	6.86	6.49	6.07	5.63	5.2	4.82	4.43	4.03	3.67	3.32
1173	3.24	4	5.62	6.86	6.49	6.06	5.63	5.2	4.81	4.44	4.05	3.68	3.29
1174	3.24	3.95	5.55	6.86	6.49	6.06	5.64	5.21	4.84	4.46	4.06	3.68	3.31
1175	3.24	4.02	5.64	6.85	6.49	6.06	5.64	5.21	4.83	4.44	4.08	3.71	3.36
1176	3.24	4.02	5.64	6.85	6.49	6.06	5.63	5.19	4.82	4.44	4.07	3.69	3.28
1177	3.24	3.75	5.32	6.82	6.45	6.03	5.6	5.17	4.8	4.43	4.04	3.66	3.29
1178	3.24	3.85	5.43	6.81	6.45	6.02	5.59	5.16	4.79	4.4	4.02	3.62	3.25
1179	3.24	3.86	5.44	6.81	6.45	6.02	5.59	5.15	4.78	4.4	4.03	3.67	3.23

1180	3.24	3.91	5.51	6.81	6.44	6.01	5.58	5.16	4.77	4.41	4.05	3.66	3.23
1181	3.24	3.91	5.48	6.81	6.44	6.01	5.58	5.16	4.78	4.41	4.02	3.65	3.3
1182	3.24	3.8	5.39	6.82	6.45	6.02	5.59	5.17	4.79	4.42	4.05	3.68	3.28
1183	3.24	3.85	5.44	6.79	6.42	5.99	5.57	5.13	4.75	4.38	3.99	3.6	3.26
1184	3.24	3.67	5.25	6.8	6.44	6.01	5.58	5.15	4.77	4.39	4.03	3.63	3.29
1185	3.24	3.8	5.38	6.8	6.43	6	5.57	5.14	4.77	4.39	4	3.58	3.16
1186	3.24	3.81	5.39	6.8	6.43	6	5.57	5.14	4.77	4.39	4.04	3.67	3.25
1187	3.24	3.88	5.45	6.79	6.43	6	5.57	5.14	4.76	4.38	4.01	3.65	3.29
1188	3.24	3.78	5.36	6.8	6.43	6	5.57	5.15	4.77	4.39	4	3.62	3.25
1189	3.24	3.86	5.42	6.79	6.42	5.99	5.56	5.13	4.74	4.37	3.99	3.61	3.25
1190	3.24	3.78	5.33	6.79	6.42	5.99	5.56	5.14	4.77	4.4	4.04	3.65	3.29
1191	3.24	3.66	5.21	6.8	6.43	6	5.57	5.15	4.77	4.39	4	3.62	3.21
1192	3.24	3.65	5.21	6.8	6.43	6	5.57	5.15	4.78	4.39	4.01	3.64	3.26
1193	3.24	3.75	5.31	6.79	6.42	5.99	5.56	5.13	4.75	4.38	4.01	3.62	3.25
1194	3.24	3.72	5.27	6.79	6.42	6	5.57	5.14	4.77	4.39	4.01	3.57	3.19
1195	3.24	4.44	6.26	7.24	7	6.46	5.89	5.31	4.8	4.27	3.73	3.1	2.52
1196	3.24	4.5	6.31	7.24	7	6.46	5.88	5.31	4.79	4.28	3.76	3.31	2.89
1197	3.24	4.46	6.26	7.24	7	6.46	5.88	5.3	4.79	4.25	3.73	3.23	2.72
1198	3.24	4.44	6.26	7.24	7	6.46	5.89	5.31	4.8	4.3	3.8	3.29	2.81
1199	3.24	4.43	6.24	7.24	7	6.46	5.89	5.32	4.82	4.31	3.78	3.28	2.92
1200	3.24	4.48	6.29	7.24	7	6.46	5.88	5.29	4.79	4.29	3.83	3.39	2.82
1201	3.24	4.28	5.99	6.99	6.53	5.95	5.37	4.79	4.29	3.79	3.26	2.72	2.13
1202	3.24	4.28	6	6.99	6.53	5.95	5.37	4.8	4.29	3.81	3.35	2.77	2.23
1203	3.24	4.31	6.01	6.99	6.53	5.95	5.37	4.77	4.28	3.72	3.17	2.61	2.4
1204	3.24	4.22	5.93	6.99	6.53	5.95	5.37	4.8	4.26	3.75	3.24	2.79	2.17
1205	3.24	4.28	6	6.99	6.53	5.95	5.36	4.78	4.28	3.73	3.2	2.6	2.2
1206	3.24	4.26	5.97	6.99	6.53	5.96	5.37	4.8	4.29	3.8	3.3	2.69	2.18
1207	3.24	3.97	5.58	6.83	6.33	5.75	5.17	4.58	4.08	3.6	3.08	2.63	2.22
1208	3.24	3.97	5.6	6.83	6.33	5.76	5.18	4.6	4.09	3.57	3.08	2.66	2.22
1209	3.24	3.89	5.49	6.84	6.34	5.76	5.18	4.59	4.09	3.63	3.11	2.56	2.16
1210	3.24	3.96	5.57	6.83	6.33	5.76	5.18	4.6	4.09	3.58	3.13	2.55	2.11
1211	3.24	3.89	5.5	6.84	6.34	5.76	5.18	4.6	4.11	3.59	3.06	2.53	1.51
1212	3.24	3.94	5.55	6.83	6.34	5.75	5.17	4.6	4.07	3.6	3.08	2.62	2.1
1213	3.24	4.22	5.93	6.98	6.44	5.77	5.1	4.42	3.84	3.25	2.72	2.2	1.34
1214	3.24	4.18	5.89	6.98	6.45	5.77	5.11	4.42	3.8	3.16	2.46	1.79	1.38
1215	3.24	4.2	5.91	6.98	6.44	5.78	5.1	4.44	3.84	3.24	2.72	2.11	1.72
1216	3.24	4.22	5.93	6.98	6.44	5.77	5.1	4.43	3.84	3.28	2.58	1.93	0.7
1217	3.24	4.23	5.94	6.98	6.44	5.77	5.1	4.41	3.84	3.22	2.48	1.74	0.3
1218	3.24	4.27	5.97	6.98	6.44	5.76	5.09	4.42	3.83	3.26	2.83	2.26	1.72
1219	3.24	4.59	6.43	7.37	7.41	7.13	6.77	6.38	6.03	5.67	5.3	4.94	4.57
1220	3.24	4.52	6.37	7.37	7.41	7.14	6.78	6.39	6.03	5.67	5.31	4.95	4.58
1221	3.24	4.58	6.43	7.37	7.41	7.13	6.77	6.38	6.03	5.67	5.3	4.94	4.58
1222	3.24	4.58	6.43	7.37	7.41	7.13	6.78	6.38	6.03	5.67	5.3	4.94	4.58
1223	3.24	4.58	6.43	7.37	7.41	7.14	6.77	6.38	6.03	5.67	5.31	4.94	4.58
1224	3.24	4.6	6.45	7.38	7.41	7.13	6.77	6.38	6.03	5.67	5.31	4.94	4.57
1225	3.24	4.57	6.41	7.33	7.24	6.8	6.27	5.7	5.18	4.66	4.13	3.62	3.11
1226	3.24	4.55	6.38	7.32	7.24	6.8	6.27	5.69	5.16	4.62	4.06	3.52	3.02
1227	3.24	4.55	6.39	7.32	7.24	6.8	6.27	5.69	5.15	4.6	4.03	3.45	2.87
1228	3.24	4.51	6.35	7.32	7.24	6.8	6.27	5.69	5.17	4.64	4.11	3.6	3.14
1229	3.24	4.57	6.41	7.33	7.24	6.8	6.26	5.68	5.16	4.63	4.08	3.51	2.9
1230	3.24	4.56	6.4	7.33	7.24	6.8	6.26	5.69	5.16	4.63	4.1	3.56	3.01
1231	3.24	4.46	6.27	7.22	6.91	6.28	5.6	4.91	4.3	3.69	3.07	2.47	1.97
1232	3.24	4.48	6.29	7.22	6.91	6.28	5.6	4.91	4.31	3.7	3.12	2.52	2.08
1233	3.24	4.42	6.23	7.22	6.92	6.28	5.6	4.91	4.32	3.7	3.08	2.29	0.95

1234	3.24	4.48	6.28	7.22	6.91	6.27	5.59	4.9	4.27	3.68	3.15	2.62	2.05
1235	3.24	4.5	6.3	7.22	6.91	6.28	5.6	4.92	4.31	3.69	3.2	2.68	2.28
1236	3.24	4.47	6.27	7.22	6.91	6.28	5.6	4.91	4.32	3.74	3.13	2.54	1.45
1237	3.24	4.37	6.14	7.11	6.65	5.97	5.28	4.59	4	3.42	2.85	2.16	0.48
1238	3.24	4.37	6.13	7.11	6.65	5.96	5.27	4.6	4.01	3.41	2.86	2.09	1.11
1239	3.24	4.38	6.14	7.11	6.65	5.96	5.26	4.58	3.94	3.27	2.6	1.72	0
1240	3.24	4.38	6.13	7.11	6.65	5.96	5.27	4.57	4	3.33	2.55	1.9	0
1241	3.24	4.41	6.17	7.11	6.65	5.97	5.28	4.59	4.01	3.38	2.75	2.18	1.53
1242	3.24	4.43	6.19	7.11	6.64	5.96	5.28	4.59	3.92	3.26	2.64	2.14	0.9
1243	3.24	4.25	5.97	6.97	6.42	5.74	5.05	4.35	3.76	3.17	2.68	2.18	1.69
1244	3.24	4.19	5.9	6.98	6.43	5.74	5.05	4.37	3.77	3.1	2.55	1.94	0.7
1245	3.24	4.15	5.87	6.98	6.43	5.74	5.05	4.37	3.73	3.2	2.57	2.15	1.46
1246	3.24	4.25	5.95	6.97	6.42	5.74	5.05	4.38	3.81	3.16	2.57	1.54	0
1247	3.24	4.23	5.94	6.97	6.42	5.73	5.04	4.36	3.74	3.16	2.56	2.03	1.86
1248	3.24	4.25	5.96	6.97	6.42	5.73	5.04	4.38	3.82	3.27	2.71	2.2	1.53
1249	3.24	4.12	5.75	6.87	6.29	5.6	4.92	4.22	3.61	3.05	2.4	1.72	0
1250	3.24	4.09	5.73	6.87	6.29	5.6	4.91	4.22	3.64	3.07	2.41	2	0
1251	3.24	4.08	5.73	6.87	6.29	5.6	4.92	4.24	3.69	3.04	2.44	1.87	0
1252	3.24	4.07	5.71	6.87	6.29	5.6	4.91	4.23	3.64	3.01	2.25	1.84	1.18
1253	3.24	4.09	5.73	6.87	6.29	5.6	4.92	4.23	3.65	3.04	2.23	1.48	1.28
1254	3.24	4.06	5.7	6.87	6.29	5.6	4.92	4.23	3.64	3.06	2.58	2.08	1.53
1255	3.24	3.91	5.51	6.82	6.23	5.54	4.85	4.17	3.58	2.95	2.14	1.23	0
1256	3.24	3.95	5.54	6.82	6.23	5.54	4.86	4.18	3.56	2.93	2.38	1.53	0
1257	3.24	3.95	5.56	6.82	6.22	5.54	4.85	4.14	3.55	2.92	2.2	1.18	0
1258	3.24	4.07	5.66	6.81	6.22	5.53	4.84	4.15	3.52	2.89	2.17	1.59	0
1259	3.24	3.99	5.6	6.81	6.22	5.53	4.85	4.16	3.55	2.94	2.26	1.76	1.51
1260	3.24	3.92	5.51	6.82	6.23	5.54	4.85	4.18	3.59	3.07	2.49	1.79	1.28
1261	3.24	3.84	5.41	6.78	6.19	5.5	4.81	4.1	3.5	2.92	2.25	1.41	1.11
1262	3.24	3.83	5.39	6.78	6.19	5.5	4.81	4.13	3.52	2.87	2.27	1.62	1.41
1263	3.24	3.89	5.44	6.78	6.18	5.49	4.81	4.12	3.52	2.95	2.34	1.89	1.15
1264	3.24	3.83	5.41	6.78	6.18	5.5	4.81	4.15	3.48	2.95	2.39	1.71	1.38
1265	3.24	3.74	5.31	6.79	6.19	5.51	4.82	4.14	3.55	2.89	2.37	1.23	0
1266	3.24	3.73	5.3	6.79	6.2	5.51	4.81	4.14	3.53	2.92	2.45	1.91	1.18
1267	3.24	3.76	5.31	6.77	6.17	5.48	4.79	4.09	3.5	2.82	2.14	1.6	1.08
1268	3.24	3.81	5.34	6.76	6.17	5.48	4.81	4.13	3.53	2.97	2.31	1.89	1.6
1269	3.24	3.75	5.3	6.77	6.17	5.49	4.79	4.11	3.55	2.92	2.39	1.6	1.08
1270	3.24	3.8	5.35	6.76	6.17	5.48	4.79	4.11	3.47	2.81	2.18	1.75	1.08
1271	3.24	3.84	5.37	6.76	6.17	5.47	4.78	4.08	3.48	2.84	2.16	1.71	1.41
1272	3.24	3.83	5.39	6.76	6.17	5.48	4.78	4.1	3.51	2.93	2.38	1.76	0
1273	3.24	4.52	6.35	7.31	7.19	6.71	6.13	5.52	4.96	4.38	3.8	3.24	2.64
1274	3.24	4.53	6.36	7.31	7.19	6.71	6.13	5.51	4.96	4.39	3.81	3.21	2.6
1275	3.24	4.54	6.37	7.31	7.19	6.7	6.13	5.51	4.96	4.39	3.81	3.23	2.62
1276	3.24	4.54	6.37	7.31	7.19	6.7	6.13	5.51	4.95	4.38	3.8	3.2	2.56
1277	3.24	4.52	6.34	7.31	7.19	6.71	6.13	5.52	4.96	4.38	3.82	3.28	2.66
1278	3.24	4.54	6.37	7.31	7.19	6.71	6.13	5.52	4.96	4.4	3.82	3.22	2.61
1279	3.24	4.5	6.29	7.2	6.84	6.13	5.36	4.58	3.93	3.25	2.56	2.08	1.3
1280	3.24	4.5	6.3	7.2	6.84	6.13	5.37	4.58	3.9	3.11	2.18	1	0
1281	3.24	4.48	6.28	7.2	6.84	6.13	5.36	4.58	3.89	3.19	2.45	1.46	0.48
1282	3.24	4.47	6.27	7.2	6.84	6.13	5.37	4.59	3.9	3.25	2.73	2.1	0.95
1283	3.24	4.44	6.24	7.2	6.84	6.13	5.36	4.58	3.92	3.23	2.53	2.08	1.67
1284	3.24	4.51	6.3	7.2	6.84	6.13	5.37	4.6	3.96	3.35	2.65	1.94	0.78
1285	3.24	4.21	5.91	6.96	6.33	5.55	4.77	3.97	3.3	2.73	1.81	1.4	0
1286	3.24	4.31	6.01	6.95	6.32	5.54	4.76	3.98	3.32	2.5	1.69	0	0
1287	3.24	4.24	5.94	6.96	6.33	5.55	4.75	4	3.32	2.6	1.57	0	0

1288	3.24	4.31	6.01	6.95	6.32	5.54	4.77	4	3.38	2.65	2.04	0.7	0
1289	3.24	4.23	5.93	6.96	6.33	5.55	4.77	3.99	3.25	2.54	1.83	0	0
1290	3.24	4.28	5.98	6.95	6.33	5.55	4.76	3.96	3.19	2.54	1.59	0	0
1291	3.24	4.03	5.62	6.8	6.12	5.34	4.56	3.77	3.14	2.44	1.49	0	0
1292	3.24	3.94	5.54	6.8	6.13	5.36	4.57	3.81	3.1	2.35	1.89	0	0
1293	3.24	3.91	5.5	6.81	6.14	5.37	4.57	3.8	3.1	2.37	1.65	0	0
1294	3.24	3.92	5.52	6.8	6.14	5.37	4.58	3.78	3.09	2.48	1.4	0	0
1295	3.24	4	5.6	6.8	6.13	5.34	4.56	3.77	3.04	2.27	1.41	1.28	0
1296	3.24	3.99	5.59	6.8	6.13	5.35	4.57	3.77	3.07	2.32	1.45	0	0
1297	3.24	3.83	5.4	6.77	6.09	5.32	4.55	3.78	3.1	2.5	1.65	0	0
1298	3.24	3.83	5.39	6.77	6.1	5.32	4.55	3.78	3.1	2.42	1.72	0	0
1299	3.24	3.8	5.35	6.77	6.1	5.33	4.55	3.76	3.06	2.31	1.52	1.18	1.08
1300	3.24	3.89	5.44	6.76	6.09	5.31	4.51	3.76	3.13	2.57	1.94	1.45	1.08
1301	3.24	3.88	5.43	6.76	6.09	5.31	4.52	3.75	3.02	2.4	0.3	0	0
1302	3.24	3.91	5.48	6.76	6.08	5.3	4.54	3.75	3.01	2.33	1.61	0	0
1303	3.24	4.45	6.25	7.2	6.81	6.07	5.27	4.45	3.75	3.04	2.24	1.08	0
1304	3.24	4.47	6.26	7.2	6.82	6.07	5.28	4.44	3.7	3.03	2.04	0.7	0
1305	3.24	4.44	6.24	7.2	6.81	6.07	5.27	4.44	3.68	2.97	2.21	1.15	0
1306	3.24	4.44	6.25	7.2	6.81	6.07	5.27	4.45	3.71	2.77	1.98	1.78	1.3
1307	3.24	4.46	6.25	7.2	6.82	6.07	5.27	4.47	3.73	2.92	1.94	0.85	0
1308	3.24	4.41	6.21	7.2	6.82	6.07	5.26	4.43	3.75	3.01	2.31	1.54	0.48
1309	3.24	4.25	5.94	6.95	6.29	5.47	4.66	3.85	3.15	2.25	1.65	0.48	0
1310	3.24	4.25	5.93	6.95	6.29	5.47	4.64	3.83	3.1	2.36	1.54	0	0
1311	3.24	4.23	5.92	6.95	6.29	5.47	4.65	3.84	3.09	2.41	2.06	0	0
1312	3.24	4.21	5.91	6.95	6.29	5.47	4.65	3.83	3.22	2.41	1.43	1.34	0
1313	3.24	4.24	5.93	6.95	6.29	5.48	4.66	3.83	3.14	2.59	1.89	1.67	1.34
1314	3.24	4.25	5.93	6.95	6.29	5.47	4.64	3.84	3.18	2.44	1.85	0	0
1315	3.24	3.98	5.56	6.79	6.09	5.27	4.46	3.67	2.96	2.3	1.41	0	0
1316	3.24	3.92	5.51	6.8	6.1	5.28	4.45	3.64	2.93	2.07	1.45	0	0
1317	3.24	3.93	5.51	6.8	6.1	5.28	4.45	3.64	2.98	2.31	1.18	1.18	0
1318	3.24	3.95	5.54	6.8	6.09	5.27	4.46	3.66	2.9	2.29	1.73	1.15	1.2
1319	3.24	3.96	5.55	6.8	6.09	5.27	4.44	3.6	2.76	2.12	1.3	0	0
1320	3.24	3.93	5.54	6.8	6.09	5.27	4.45	3.64	2.94	2.17	1.3	0	0
1321	3.24	4.25	5.94	6.95	6.27	5.42	4.58	3.78	2.98	2.29	1.83	0	0
1322	3.24	4.12	5.81	6.95	6.29	5.45	4.6	3.74	3.01	2.17	1.56	0.7	0
1323	3.24	4.21	5.9	6.95	6.27	5.43	4.58	3.75	3	2.06	0	0	0
1324	3.24	4.24	5.93	6.95	6.27	5.43	4.58	3.72	2.98	2.23	1.79	0	0
1325	3.24	4.22	5.9	6.95	6.27	5.43	4.59	3.76	2.97	2.24	1.89	1.71	0
1326	3.24	4.2	5.9	6.95	6.27	5.43	4.6	3.74	3	1.97	1.4	0	0
1327	3.24	4.52	6.51	7.07	6.93	6.76	6.58	6.4	6.24	6.09	5.93	5.78	5.63
1328	3.24	4.51	6.5	7.08	6.93	6.76	6.58	6.4	6.25	6.09	5.93	5.78	5.62
1329	3.24	4.47	6.46	7.08	6.94	6.76	6.58	6.4	6.24	6.09	5.93	5.78	5.62
1330	3.24	4.5	6.5	7.08	6.93	6.76	6.58	6.4	6.24	6.09	5.93	5.78	5.62
1331	3.24	4.52	6.52	7.07	6.93	6.76	6.58	6.4	6.25	6.09	5.93	5.78	5.62
1332	3.24	4.44	6.44	7.08	6.94	6.76	6.58	6.4	6.25	6.09	5.94	5.78	5.63
1333	3.24	4.72	6.76	7.28	7.1	6.69	6.26	5.84	5.46	5.09	4.71	4.32	3.93
1334	3.24	4.75	6.78	7.29	7.1	6.69	6.26	5.84	5.47	5.1	4.72	4.33	3.95
1335	3.24	4.69	6.73	7.28	7.1	6.69	6.27	5.84	5.47	5.1	4.72	4.35	3.96
1336	3.24	4.68	6.73	7.28	7.1	6.7	6.27	5.84	5.46	5.09	4.72	4.33	3.95
1337	3.24	4.7	6.74	7.28	7.1	6.69	6.26	5.84	5.46	5.07	4.69	4.3	3.93
1338	3.24	4.69	6.73	7.28	7.1	6.69	6.27	5.84	5.46	5.08	4.7	4.31	3.9
1339	3.24	4.46	6.44	7.01	6.65	6.22	5.79	5.37	4.98	4.59	4.2	3.81	3.47
1340	3.24	4.49	6.48	7.01	6.65	6.22	5.8	5.37	4.99	4.61	4.25	3.86	3.44
1341	3.24	4.52	6.5	7.01	6.65	6.22	5.8	5.37	5	4.62	4.24	3.85	3.45

1342	3.24	4.53	6.51	7.01	6.65	6.22	5.79	5.36	4.98	4.6	4.23	3.87	3.43
1343	3.24	4.5	6.48	7.01	6.65	6.22	5.8	5.37	5	4.63	4.24	3.85	3.42
1344	3.24	4.48	6.47	7.01	6.65	6.22	5.79	5.36	4.98	4.61	4.26	3.87	3.51
1345	3.24	4.12	6.03	6.83	6.45	6.02	5.6	5.17	4.8	4.43	4.05	3.7	3.27
1346	3.24	4.19	6.1	6.82	6.45	6.02	5.6	5.17	4.78	4.42	4.05	3.67	3.3
1347	3.24	4.29	6.2	6.82	6.44	6.02	5.59	5.16	4.79	4.41	4.03	3.66	3.26
1348	3.24	4.22	6.13	6.82	6.45	6.02	5.6	5.17	4.79	4.41	4.04	3.69	3.3
1349	3.24	4.2	6.12	6.82	6.45	6.02	5.6	5.17	4.79	4.42	4.05	3.67	3.31
1350	3.24	4.19	6.1	6.82	6.45	6.02	5.59	5.16	4.79	4.42	4.02	3.63	3.28
1351	3.24	4.43	6.4	6.94	6.37	5.68	5	4.3	3.72	3.15	2.47	1.89	0
1352	3.24	4.46	6.43	6.94	6.37	5.68	4.98	4.3	3.72	3.18	2.66	1.73	1.36
1353	3.24	4.49	6.46	6.94	6.36	5.67	4.98	4.27	3.68	3.01	2.36	1.95	1.91
1354	3.24	4.49	6.46	6.94	6.36	5.67	4.99	4.3	3.71	3.09	2.5	2.05	0.85
1355	3.24	4.45	6.43	6.94	6.37	5.68	4.99	4.33	3.78	3.09	2.46	1.56	1.3
1356	3.24	4.47	6.44	6.94	6.37	5.68	4.99	4.29	3.68	3.05	2.5	1.92	1.68
1357	3.24	4.6	6.68	7	6.64	6.22	5.79	5.36	4.99	4.6	4.23	3.86	3.48
1358	3.24	4.58	6.67	7	6.64	6.21	5.79	5.36	4.99	4.61	4.25	3.87	3.46
1359	3.24	4.6	6.7	7	6.64	6.21	5.78	5.35	4.98	4.61	4.26	3.87	3.53
1360	3.24	4.58	6.67	7	6.64	6.21	5.78	5.36	4.99	4.61	4.25	3.84	3.44
1361	3.24	4.55	6.66	7	6.64	6.21	5.78	5.35	4.98	4.6	4.22	3.87	3.41
1362	3.24	4.6	6.68	7	6.64	6.21	5.79	5.36	4.98	4.59	4.2	3.82	3.45
1363	3.24	4.69	6.8	7.11	7.07	7.01	6.95	6.89	6.84	6.79	6.74	6.69	6.64
1364	3.24	4.62	6.75	7.11	7.07	7.01	6.95	6.9	6.84	6.79	6.74	6.69	6.64
1365	3.24	4.62	6.75	7.11	7.07	7.01	6.95	6.89	6.84	6.79	6.74	6.69	6.64
1366	3.24	4.59	6.73	7.11	7.07	7.01	6.95	6.89	6.84	6.79	6.74	6.69	6.64
1367	3.24	4.63	6.76	7.11	7.07	7.01	6.95	6.89	6.84	6.79	6.74	6.69	6.64
1368	3.24	4.64	6.77	7.11	7.07	7.01	6.95	6.89	6.84	6.79	6.74	6.69	6.64
1369	3.24	4.86	6.94	7.38	7.41	7.32	7.23	7.13	7.05	6.97	6.89	6.8	6.72
1370	3.24	4.89	6.95	7.38	7.41	7.32	7.23	7.13	7.05	6.97	6.89	6.8	6.72
1371	3.24	4.87	6.94	7.38	7.41	7.32	7.23	7.14	7.05	6.97	6.89	6.81	6.72
1372	3.24	4.82	6.93	7.37	7.41	7.32	7.23	7.13	7.05	6.97	6.89	6.8	6.72
1373	3.24	4.81	6.92	7.37	7.41	7.32	7.23	7.13	7.05	6.97	6.89	6.8	6.72
1374	3.24	4.82	6.93	7.37	7.41	7.32	7.23	7.13	7.05	6.97	6.89	6.81	6.72
1375	3.24	4.6	6.73	7.1	7.03	6.93	6.84	6.74	6.66	6.58	6.5	6.41	6.33
1376	3.24	4.63	6.76	7.1	7.03	6.93	6.84	6.74	6.66	6.58	6.49	6.41	6.33
1377	3.24	4.58	6.71	7.1	7.03	6.93	6.84	6.74	6.66	6.58	6.49	6.41	6.33
1378	3.24	4.61	6.74	7.1	7.03	6.93	6.84	6.74	6.66	6.58	6.49	6.41	6.33
1379	3.24	4.63	6.75	7.1	7.03	6.93	6.84	6.74	6.66	6.58	6.5	6.41	6.33
1380	3.24	4.68	6.79	7.1	7.03	6.93	6.84	6.74	6.66	6.58	6.49	6.41	6.33
1381	3.24	4.3	6.39	6.91	6.83	6.73	6.64	6.55	6.46	6.38	6.3	6.21	6.13
1382	3.24	4.42	6.51	6.91	6.83	6.73	6.64	6.54	6.46	6.38	6.3	6.22	6.13
1383	3.24	4.35	6.44	6.91	6.83	6.73	6.64	6.54	6.46	6.38	6.3	6.21	6.13
1384	3.24	4.32	6.41	6.91	6.83	6.73	6.64	6.54	6.46	6.38	6.3	6.21	6.13
1385	3.24	4.34	6.44	6.91	6.83	6.73	6.64	6.54	6.46	6.38	6.29	6.21	6.13
1386	3.24	4.36	6.44	6.91	6.83	6.73	6.64	6.54	6.46	6.38	6.3	6.22	6.13
1387	3.24	4.94	6.98	7.47	7.58	7.47	7.31	7.14	6.99	6.83	6.68	6.52	6.36
1388	3.24	4.93	6.98	7.47	7.58	7.47	7.31	7.14	6.98	6.83	6.67	6.51	6.36
1389	3.24	4.89	6.97	7.47	7.58	7.47	7.31	7.14	6.99	6.83	6.67	6.52	6.36
1390	3.24	4.88	6.97	7.47	7.58	7.47	7.31	7.14	6.98	6.83	6.67	6.52	6.36
1391	3.24	4.89	6.97	7.47	7.58	7.47	7.31	7.14	6.98	6.83	6.67	6.52	6.36
1392	3.24	4.88	6.96	7.47	7.58	7.47	7.31	7.14	6.99	6.83	6.68	6.52	6.37
1393	3.24	4.85	6.93	7.35	7.33	7.16	6.98	6.8	6.65	6.49	6.34	6.18	6.03
1394	3.24	4.79	6.92	7.35	7.33	7.16	6.98	6.8	6.65	6.49	6.34	6.18	6.03
1395	3.24	4.87	6.94	7.35	7.33	7.16	6.98	6.8	6.65	6.49	6.34	6.18	6.03

1396	3.24	4.82	6.93	7.35	7.33	7.16	6.98	6.8	6.65	6.49	6.34	6.18	6.02
1397	3.24	4.89	6.94	7.35	7.32	7.16	6.98	6.8	6.65	6.49	6.34	6.18	6.02
1398	3.24	4.85	6.93	7.35	7.33	7.16	6.98	6.8	6.65	6.49	6.34	6.18	6.03
1399	3.24	4.65	6.76	7.07	6.93	6.75	6.57	6.39	6.24	6.08	5.93	5.77	5.61
1400	3.24	4.68	6.78	7.07	6.93	6.75	6.57	6.39	6.24	6.08	5.93	5.77	5.62
1401	3.24	4.64	6.76	7.07	6.93	6.75	6.57	6.4	6.24	6.09	5.93	5.77	5.62
1402	3.24	4.61	6.74	7.07	6.93	6.75	6.57	6.39	6.24	6.08	5.92	5.77	5.61
1403	3.24	4.63	6.75	7.07	6.93	6.75	6.57	6.39	6.24	6.08	5.93	5.77	5.62
1404	3.24	4.61	6.74	7.07	6.93	6.75	6.57	6.39	6.24	6.08	5.93	5.77	5.61
1405	3.24	4.41	6.49	6.88	6.73	6.55	6.37	6.19	6.04	5.88	5.72	5.57	5.41
1406	3.24	4.39	6.47	6.88	6.73	6.55	6.37	6.19	6.04	5.88	5.72	5.57	5.41
1407	3.24	4.4	6.48	6.88	6.73	6.55	6.37	6.19	6.04	5.88	5.72	5.57	5.41
1408	3.24	4.29	6.39	6.88	6.73	6.55	6.37	6.19	6.04	5.88	5.73	5.58	5.42
1409	3.24	4.36	6.44	6.88	6.73	6.55	6.37	6.19	6.04	5.88	5.73	5.57	5.42
1410	3.24	4.38	6.46	6.88	6.73	6.55	6.37	6.19	6.04	5.88	5.73	5.57	5.42
1411	3.24	4.23	6.29	6.83	6.68	6.5	6.32	6.14	5.99	5.83	5.68	5.52	5.37
1412	3.24	4.31	6.38	6.83	6.68	6.5	6.32	6.14	5.99	5.83	5.68	5.52	5.36
1413	3.24	4.21	6.27	6.83	6.68	6.5	6.32	6.14	5.99	5.83	5.68	5.52	5.36
1414	3.24	4.35	6.41	6.83	6.67	6.5	6.32	6.14	5.99	5.83	5.67	5.51	5.36
1415	3.24	4.27	6.33	6.83	6.68	6.5	6.32	6.14	5.99	5.83	5.68	5.52	5.36
1416	3.24	4.31	6.37	6.83	6.68	6.5	6.32	6.14	5.99	5.83	5.68	5.52	5.36
1417	3.24	4.66	6.77	7.04	6.81	6.53	6.25	5.97	5.73	5.48	5.24	5	4.75
1418	3.24	4.67	6.77	7.04	6.81	6.53	6.25	5.96	5.72	5.48	5.22	4.98	4.74
1419	3.24	4.62	6.74	7.04	6.81	6.53	6.25	5.97	5.72	5.48	5.24	4.99	4.75
1420	3.24	4.61	6.73	7.04	6.81	6.53	6.25	5.97	5.72	5.47	5.23	4.99	4.75
1421	3.24	4.64	6.76	7.04	6.81	6.53	6.25	5.97	5.72	5.48	5.23	4.99	4.74
1422	3.24	4.6	6.72	7.04	6.81	6.53	6.25	5.97	5.72	5.48	5.24	4.99	4.76
1423	3.24	4.95	6.99	7.47	7.55	7.37	7.1	6.78	6.49	6.19	5.88	5.56	5.23
1424	3.24	4.94	6.99	7.47	7.55	7.36	7.09	6.78	6.49	6.18	5.87	5.55	5.22
1425	3.24	4.93	6.98	7.47	7.55	7.36	7.09	6.78	6.49	6.18	5.87	5.55	5.23
1426	3.24	4.95	6.99	7.47	7.55	7.36	7.09	6.78	6.49	6.18	5.87	5.55	5.23
1427	3.24	4.91	6.98	7.47	7.55	7.36	7.09	6.78	6.49	6.19	5.87	5.55	5.23
1428	3.24	4.95	6.99	7.47	7.55	7.37	7.09	6.78	6.49	6.19	5.88	5.56	5.23
1429	3.24	4.87	6.95	7.41	7.38	7.09	6.72	6.32	5.95	5.58	5.21	4.84	4.45
1430	3.24	4.9	6.96	7.41	7.38	7.09	6.72	6.32	5.96	5.6	5.24	4.88	4.49
1431	3.24	4.92	6.97	7.41	7.38	7.09	6.72	6.31	5.95	5.58	5.2	4.82	4.43
1432	3.24	4.9	6.96	7.41	7.38	7.09	6.71	6.31	5.96	5.59	5.22	4.84	4.46
1433	3.24	4.9	6.96	7.41	7.38	7.09	6.72	6.32	5.95	5.59	5.22	4.86	4.49
1434	3.24	4.87	6.95	7.41	7.38	7.09	6.72	6.31	5.95	5.58	5.21	4.83	4.44
1435	3.24	4.86	6.92	7.29	7.08	6.68	6.25	5.83	5.46	5.08	4.71	4.34	3.98
1436	3.24	4.83	6.92	7.29	7.08	6.68	6.25	5.83	5.45	5.06	4.7	4.33	3.95
1437	3.24	4.87	6.93	7.29	7.08	6.68	6.25	5.82	5.44	5.07	4.69	4.33	3.96
1438	3.24	4.83	6.92	7.29	7.08	6.68	6.25	5.82	5.45	5.08	4.69	4.32	3.97
1439	3.24	4.82	6.91	7.29	7.08	6.68	6.25	5.83	5.45	5.07	4.69	4.31	3.95
1440	3.24	4.82	6.92	7.29	7.09	6.68	6.25	5.82	5.44	5.07	4.69	4.32	3.96
1441	3.24	4.72	6.86	7.16	6.85	6.42	5.99	5.57	5.19	4.81	4.45	4.05	3.66
1442	3.24	4.76	6.88	7.16	6.85	6.42	5.99	5.56	5.18	4.81	4.44	4.04	3.69
1443	3.24	4.75	6.87	7.16	6.85	6.42	5.99	5.57	5.2	4.81	4.41	4.06	3.64
1444	3.24	4.74	6.87	7.16	6.85	6.42	5.99	5.56	5.18	4.8	4.43	4.07	3.68
1445	3.24	4.79	6.88	7.16	6.84	6.42	5.99	5.56	5.19	4.83	4.44	4.06	3.66
1446	3.24	4.74	6.87	7.16	6.85	6.42	5.99	5.56	5.19	4.82	4.46	4.1	3.75
1447	3.24	4.66	6.76	7	6.64	6.21	5.78	5.34	4.97	4.6	4.22	3.86	3.46
1448	3.24	4.66	6.76	7	6.63	6.21	5.77	5.34	4.96	4.57	4.17	3.77	3.38
1449	3.24	4.62	6.73	7	6.64	6.21	5.78	5.36	4.98	4.6	4.22	3.83	3.43

1450	3.24	4.63	6.74	7	6.64	6.21	5.78	5.35	4.97	4.59	4.23	3.87	3.54
1451	3.24	4.63	6.73	7	6.64	6.21	5.78	5.34	4.97	4.6	4.24	3.89	3.51
1452	3.24	4.6	6.72	7	6.64	6.21	5.78	5.35	4.97	4.61	4.24	3.87	3.48
1453	3.24	4.47	6.56	6.87	6.5	6.07	5.64	5.22	4.83	4.46	4.08	3.7	3.32
1454	3.24	4.46	6.55	6.88	6.5	6.07	5.64	5.21	4.84	4.46	4.1	3.72	3.36
1455	3.24	4.49	6.58	6.87	6.5	6.07	5.64	5.21	4.84	4.47	4.07	3.69	3.26
1456	3.24	4.56	6.63	6.87	6.5	6.07	5.64	5.22	4.83	4.47	4.11	3.73	3.35
1457	3.24	4.49	6.57	6.87	6.5	6.07	5.64	5.22	4.84	4.46	4.05	3.67	3.29
1458	3.24	4.47	6.56	6.87	6.5	6.07	5.64	5.22	4.83	4.45	4.07	3.7	3.3
1459	3.24	4.33	6.4	6.8	6.43	6	5.57	5.14	4.77	4.39	4.01	3.62	3.25
1460	3.24	4.35	6.42	6.8	6.43	6	5.58	5.15	4.77	4.4	4.04	3.65	3.26
1461	3.24	4.36	6.42	6.8	6.43	6	5.57	5.14	4.77	4.4	4.04	3.68	3.3
1462	3.24	4.27	6.35	6.81	6.43	6.01	5.57	5.15	4.77	4.41	4.04	3.69	3.3
1463	3.24	4.39	6.45	6.8	6.43	6	5.57	5.14	4.77	4.41	4.04	3.64	3.28
1464	3.24	4.39	6.44	6.8	6.43	6	5.57	5.14	4.77	4.38	4.01	3.63	3.27
1465	3.24	4.21	6.27	6.76	6.38	5.95	5.52	5.1	4.73	4.35	3.98	3.6	3.21
1466	3.24	4.09	6.14	6.77	6.39	5.96	5.54	5.1	4.73	4.35	3.99	3.63	3.24
1467	3.24	4.24	6.29	6.76	6.38	5.95	5.53	5.09	4.72	4.34	3.99	3.63	3.29
1468	3.24	4.28	6.33	6.75	6.38	5.95	5.52	5.09	4.71	4.33	3.97	3.59	3.24
1469	3.24	4.26	6.3	6.76	6.38	5.95	5.52	5.1	4.72	4.35	3.97	3.62	3.28
1470	3.24	4.16	6.22	6.76	6.39	5.96	5.53	5.1	4.72	4.34	3.96	3.55	3.18
1471	3.24	4.17	6.2	6.74	6.36	5.94	5.51	5.08	4.7	4.32	3.95	3.56	3.17
1472	3.24	4.13	6.18	6.74	6.37	5.94	5.51	5.08	4.71	4.33	3.97	3.57	3.25
1473	3.24	4.23	6.26	6.74	6.36	5.93	5.5	5.06	4.68	4.3	3.93	3.56	3.14
1474	3.24	4.19	6.23	6.74	6.36	5.93	5.5	5.07	4.7	4.32	3.95	3.58	3.24
1475	3.24	4.21	6.24	6.74	6.36	5.93	5.51	5.08	4.71	4.33	3.93	3.55	3.17
1476	3.24	4.17	6.22	6.74	6.36	5.93	5.51	5.08	4.7	4.33	3.95	3.58	3.18
1477	3.24	4.6	6.71	6.95	6.46	5.88	5.31	4.71	4.18	3.69	3.13	2.44	2.03
1478	3.24	4.65	6.74	6.95	6.46	5.88	5.3	4.73	4.21	3.74	3.17	2.7	2.39
1479	3.24	4.62	6.72	6.95	6.46	5.88	5.3	4.73	4.21	3.7	3.19	2.44	1.64
1480	3.24	4.61	6.72	6.95	6.46	5.88	5.29	4.7	4.19	3.65	3.13	2.48	1.85
1481	3.24	4.64	6.73	6.95	6.46	5.88	5.3	4.71	4.2	3.69	3.17	2.65	2.18
1482	3.24	4.61	6.71	6.95	6.47	5.89	5.31	4.74	4.25	3.74	3.25	2.85	2.5
1483	3.24	4.86	6.94	7.36	7.2	6.75	6.21	5.63	5.11	4.56	4.02	3.48	2.93
1484	3.24	4.89	6.95	7.36	7.2	6.75	6.22	5.64	5.11	4.55	4	3.43	2.84
1485	3.24	4.92	6.96	7.36	7.2	6.75	6.21	5.63	5.11	4.57	4.03	3.48	2.89
1486	3.24	4.9	6.95	7.36	7.2	6.75	6.21	5.63	5.1	4.55	4.01	3.44	2.87
1487	3.24	4.9	6.95	7.36	7.2	6.75	6.22	5.64	5.11	4.58	4.04	3.5	2.91
1488	3.24	4.89	6.95	7.36	7.2	6.75	6.21	5.64	5.12	4.59	4.06	3.51	2.96
1489	3.24	4.8	6.9	7.23	6.85	6.21	5.53	4.83	4.25	3.65	3.12	2.65	1.87
1490	3.24	4.82	6.91	7.23	6.85	6.21	5.53	4.85	4.25	3.61	2.96	2.03	0.95
1491	3.24	4.81	6.9	7.23	6.85	6.21	5.53	4.84	4.24	3.67	3.01	2.54	1.83
1492	3.24	4.84	6.91	7.23	6.85	6.21	5.53	4.83	4.24	3.61	2.91	2.15	1.4
1493	3.24	4.85	6.91	7.23	6.85	6.21	5.53	4.85	4.27	3.7	3.08	2.49	2.1
1494	3.24	4.82	6.9	7.23	6.85	6.21	5.53	4.85	4.27	3.72	3.19	2.64	1.83
1495	3.24	4.61	6.7	6.92	6.34	5.65	4.96	4.27	3.61	2.97	2.19	2.01	1.41
1496	3.24	4.64	6.73	6.92	6.34	5.65	4.96	4.25	3.65	3.12	2.61	2	1.69
1497	3.24	4.63	6.72	6.92	6.34	5.65	4.96	4.28	3.68	3.1	2.5	1.67	0
1498	3.24	4.62	6.72	6.92	6.34	5.65	4.97	4.31	3.7	3.05	2.39	1.51	0.48
1499	3.24	4.62	6.71	6.92	6.34	5.65	4.96	4.28	3.72	3.13	2.5	1.73	0
1500	3.24	4.61	6.71	6.92	6.34	5.65	4.96	4.27	3.71	3.02	2.53	1.95	1.45
1501	3.24	4.39	6.43	6.72	6.12	5.43	4.75	4.05	3.42	2.76	2.18	1.51	1.15
1502	3.24	4.38	6.42	6.72	6.12	5.43	4.74	4.06	3.46	2.93	2.37	2.06	1.69
1503	3.24	4.39	6.44	6.72	6.12	5.44	4.74	4.07	3.49	2.9	2.32	1.99	1.51

1504	3.24	4.37	6.42	6.72	6.12	5.43	4.75	4.07	3.45	2.78	2.2	1.82	0
1505	3.24	4.38	6.42	6.72	6.12	5.43	4.75	4.08	3.52	2.84	2.17	1.48	1.26
1506	3.24	4.33	6.38	6.73	6.12	5.43	4.76	4.06	3.45	2.84	2.18	1.81	1.23
1507	3.24	4.3	6.31	6.67	6.07	5.39	4.7	4.03	3.41	2.68	2.03	1.45	0
1508	3.24	4.17	6.2	6.69	6.08	5.39	4.71	4.02	3.42	2.74	1.87	1.11	1.2
1509	3.24	4.22	6.26	6.68	6.08	5.39	4.7	4.01	3.39	2.78	2.28	1.43	1.08
1510	3.24	4.24	6.28	6.68	6.08	5.39	4.68	3.99	3.33	2.73	2.22	1.58	1.3
1511	3.24	4.24	6.28	6.68	6.07	5.39	4.7	3.99	3.36	2.78	1.87	1.49	1.15
1512	3.24	4.18	6.22	6.68	6.08	5.39	4.69	4	3.4	2.8	2.19	1.79	1.08
1513	3.24	4.84	6.91	7.21	6.77	6.05	5.28	4.51	3.86	3.21	2.51	1.32	0.78
1514	3.24	4.82	6.9	7.21	6.77	6.05	5.29	4.52	3.87	3.14	2.42	1.81	0.78
1515	3.24	4.83	6.9	7.21	6.77	6.06	5.29	4.52	3.87	3.22	2.59	2.09	1.32
1516	3.24	4.84	6.91	7.21	6.77	6.05	5.3	4.53	3.86	3.13	2.05	0.85	0
1517	3.24	4.8	6.9	7.21	6.78	6.05	5.29	4.49	3.83	3.2	2.37	1.49	0.48
1518	3.24	4.83	6.9	7.21	6.77	6.06	5.3	4.53	3.82	3.17	2.4	1.85	0.78
1519	3.24	4.63	6.71	6.9	6.23	5.45	4.69	3.9	3.22	2.6	1.96	1.36	0
1520	3.24	4.61	6.71	6.9	6.24	5.46	4.68	3.91	3.29	2.6	2.03	0	0
1521	3.24	4.61	6.7	6.9	6.23	5.46	4.7	3.93	3.24	2.49	1.51	0	0
1522	3.24	4.66	6.73	6.89	6.23	5.45	4.67	3.86	3.27	2.56	1.63	1.4	0
1523	3.24	4.6	6.69	6.9	6.24	5.46	4.68	3.88	3.23	2.57	1.84	1.28	0.78
1524	3.24	4.58	6.68	6.9	6.24	5.46	4.67	3.89	3.23	2.68	2.1	1.34	0.6
1525	3.24	4.32	6.37	6.7	6.02	5.24	4.47	3.7	2.97	2.36	1.56	0	0
1526	3.24	4.37	6.42	6.69	6.01	5.23	4.44	3.67	2.97	2.16	0	0	0
1527	3.24	4.27	6.32	6.7	6.02	5.25	4.46	3.7	3.08	2.51	1.61	1.23	0
1528	3.24	4.41	6.45	6.69	6.01	5.23	4.44	3.63	2.95	2.35	1.64	0	0
1529	3.24	4.4	6.44	6.69	6.01	5.23	4.46	3.67	2.94	2.2	1.34	0	0
1530	3.24	4.33	6.37	6.7	6.01	5.24	4.46	3.68	2.97	2.12	1.2	0	0
1531	3.24	4.59	6.69	6.89	6.19	5.38	4.56	3.71	3	2.03	1.32	0	0
1532	3.24	4.61	6.7	6.89	6.19	5.37	4.53	3.7	2.97	2.28	1.15	0	0
1533	3.24	4.56	6.66	6.89	6.19	5.38	4.57	3.78	3.06	2.33	1.2	0	0
1534	3.24	4.59	6.69	6.89	6.2	5.38	4.56	3.69	2.98	2.05	1.4	0	0
1535	3.24	4.63	6.72	6.88	6.19	5.37	4.56	3.73	2.98	2.36	0.3	0	0
1536	3.24	4.59	6.68	6.89	6.19	5.37	4.55	3.74	3.01	2.06	0.6	0	0
1537	3.24	4.77	6.88	7.1	7.02	6.93	6.83	6.74	6.66	6.57	6.49	6.41	6.32
1538	3.24	4.74	6.87	7.1	7.02	6.93	6.83	6.74	6.66	6.57	6.49	6.41	6.33
1539	3.24	4.71	6.87	7.1	7.02	6.93	6.84	6.74	6.66	6.58	6.49	6.41	6.33
1540	3.24	4.79	6.88	7.1	7.02	6.93	6.84	6.74	6.66	6.58	6.49	6.41	6.33
1541	3.24	4.7	6.87	7.1	7.02	6.93	6.84	6.74	6.66	6.58	6.49	6.41	6.33
1542	3.24	4.79	6.89	7.1	7.02	6.93	6.83	6.74	6.66	6.57	6.49	6.41	6.33
1543	3.24	4.95	6.99	7.36	7.32	7.15	6.98	6.8	6.64	6.49	6.33	6.18	6.03
1544	3.24	4.99	7.01	7.36	7.32	7.15	6.98	6.8	6.64	6.49	6.33	6.17	6.01
1545	3.24	4.94	6.99	7.36	7.32	7.15	6.98	6.8	6.64	6.49	6.33	6.17	6.02
1546	3.24	4.96	7	7.36	7.32	7.15	6.98	6.8	6.64	6.49	6.33	6.18	6.02
1547	3.24	4.95	7	7.36	7.32	7.16	6.98	6.8	6.65	6.49	6.33	6.18	6.02
1548	3.24	4.93	6.99	7.36	7.32	7.15	6.98	6.8	6.64	6.49	6.33	6.18	6.02
1549	3.24	4.75	6.87	7.07	6.92	6.75	6.57	6.39	6.23	6.08	5.92	5.76	5.61
1550	3.24	4.78	6.88	7.07	6.92	6.74	6.57	6.39	6.23	6.08	5.92	5.77	5.61
1551	3.24	4.72	6.87	7.07	6.93	6.75	6.57	6.39	6.24	6.08	5.93	5.77	5.62
1552	3.24	4.75	6.87	7.07	6.92	6.75	6.57	6.39	6.23	6.08	5.92	5.76	5.61
1553	3.24	4.71	6.86	7.07	6.92	6.75	6.57	6.39	6.24	6.08	5.93	5.77	5.61
1554	3.24	4.74	6.87	7.07	6.92	6.74	6.57	6.39	6.23	6.08	5.92	5.77	5.61
1555	3.24	4.43	6.62	6.88	6.72	6.54	6.37	6.19	6.03	5.88	5.72	5.57	5.42
1556	3.24	4.47	6.66	6.88	6.72	6.54	6.37	6.19	6.03	5.88	5.72	5.57	5.41
1557	3.24	4.44	6.62	6.88	6.72	6.54	6.37	6.19	6.03	5.87	5.72	5.56	5.41

1558	3.24	4.42	6.62	6.88	6.72	6.55	6.37	6.19	6.03	5.88	5.72	5.57	5.41
1559	3.24	4.46	6.64	6.88	6.72	6.55	6.37	6.19	6.03	5.88	5.72	5.57	5.42
1560	3.24	4.42	6.62	6.88	6.72	6.54	6.37	6.19	6.03	5.87	5.72	5.56	5.41
1561	3.24	4.98	7.02	7.42	7.38	7.08	6.71	6.31	5.95	5.58	5.21	4.84	4.47
1562	3.24	4.98	7.02	7.42	7.38	7.08	6.71	6.31	5.95	5.58	5.21	4.83	4.46
1563	3.24	5.01	7.03	7.42	7.38	7.08	6.7	6.3	5.94	5.57	5.19	4.83	4.46
1564	3.24	4.98	7.02	7.42	7.38	7.08	6.71	6.31	5.94	5.58	5.22	4.84	4.46
1565	3.24	5.02	7.04	7.42	7.38	7.08	6.7	6.3	5.94	5.58	5.2	4.83	4.45
1566	3.24	4.99	7.02	7.42	7.38	7.08	6.71	6.31	5.94	5.57	5.2	4.83	4.46
1567	3.24	4.91	6.97	7.29	7.08	6.67	6.25	5.82	5.45	5.08	4.72	4.33	3.95
1568	3.24	4.93	6.98	7.29	7.08	6.67	6.24	5.81	5.43	5.06	4.69	4.34	3.94
1569	3.24	4.96	6.99	7.29	7.08	6.67	6.24	5.81	5.43	5.06	4.68	4.33	3.94
1570	3.24	4.94	6.98	7.29	7.08	6.67	6.24	5.81	5.44	5.06	4.69	4.33	3.93
1571	3.24	4.92	6.97	7.29	7.08	6.67	6.24	5.82	5.44	5.06	4.7	4.32	3.92
1572	3.24	4.92	6.97	7.29	7.08	6.67	6.24	5.82	5.44	5.08	4.7	4.31	3.92
1573	3.24	4.77	6.87	6.99	6.63	6.2	5.77	5.34	4.97	4.6	4.21	3.82	3.39
1574	3.24	4.72	6.86	6.99	6.63	6.2	5.76	5.33	4.95	4.57	4.16	3.81	3.44
1575	3.24	4.71	6.86	6.99	6.63	6.2	5.77	5.34	4.96	4.59	4.2	3.8	3.38
1576	3.24	4.76	6.86	6.99	6.63	6.2	5.77	5.34	4.96	4.6	4.22	3.83	3.47
1577	3.24	4.73	6.86	6.99	6.63	6.2	5.77	5.34	4.97	4.6	4.21	3.82	3.42
1578	3.24	4.76	6.86	6.99	6.63	6.2	5.77	5.34	4.97	4.6	4.22	3.83	3.48
1579	3.24	4.43	6.61	6.79	6.42	5.99	5.56	5.13	4.76	4.39	4.02	3.64	3.29
1580	3.24	4.42	6.6	6.8	6.42	5.99	5.56	5.13	4.76	4.38	4	3.68	3.28
1581	3.24	4.48	6.64	6.79	6.42	5.99	5.57	5.14	4.77	4.4	4.02	3.63	3.24
1582	3.24	4.52	6.67	6.79	6.42	5.99	5.56	5.14	4.75	4.38	4.02	3.65	3.27
1583	3.24	4.55	6.69	6.79	6.41	5.99	5.56	5.13	4.75	4.37	3.99	3.64	3.26
1584	3.24	4.48	6.64	6.79	6.42	5.99	5.56	5.13	4.75	4.38	3.97	3.61	3.26
1585	3.24	4.33	6.5	6.74	6.37	5.94	5.52	5.09	4.71	4.34	3.97	3.62	3.27
1586	3.24	4.39	6.54	6.74	6.37	5.94	5.51	5.08	4.7	4.33	3.95	3.58	3.29
1587	3.24	4.31	6.48	6.74	6.37	5.94	5.51	5.09	4.72	4.33	3.97	3.59	3.24
1588	3.24	4.37	6.52	6.74	6.37	5.94	5.51	5.08	4.72	4.34	3.98	3.62	3.24
1589	3.24	4.34	6.51	6.74	6.37	5.94	5.51	5.09	4.72	4.36	3.98	3.64	3.22
1590	3.24	4.27	6.44	6.75	6.37	5.94	5.52	5.08	4.71	4.34	3.95	3.56	3.18
1591	3.24	4.95	6.97	7.23	6.84	6.2	5.52	4.83	4.22	3.64	3.06	2.47	1.88
1592	3.24	4.92	6.96	7.23	6.84	6.2	5.53	4.86	4.27	3.64	3	2.37	1.3
1593	3.24	4.91	6.96	7.23	6.84	6.2	5.52	4.84	4.24	3.66	3	2.51	1.98
1594	3.24	4.93	6.96	7.23	6.84	6.19	5.52	4.84	4.24	3.66	3.06	2.54	1.95
1595	3.24	4.9	6.96	7.23	6.84	6.2	5.52	4.82	4.21	3.57	3.02	2.6	2.1
1596	3.24	4.94	6.97	7.23	6.84	6.19	5.51	4.81	4.22	3.64	2.93	1.93	0.9
1597	3.24	4.73	6.85	6.91	6.32	5.63	4.95	4.25	3.68	3.06	2.64	1.85	0
1598	3.24	4.72	6.85	6.91	6.32	5.64	4.94	4.24	3.64	3.09	2.46	1.58	1.36
1599	3.24	4.71	6.85	6.91	6.32	5.63	4.94	4.25	3.66	3.01	2.47	1.65	0.7
1600	3.24	4.73	6.85	6.91	6.32	5.63	4.94	4.27	3.68	3.09	2.24	1.81	1.72
1601	3.24	4.71	6.85	6.91	6.32	5.64	4.93	4.27	3.69	3.18	2.73	2.24	1.71
1602	3.24	4.69	6.84	6.91	6.32	5.64	4.96	4.27	3.67	3.08	2.58	2.19	1.4
1603	3.24	4.4	6.57	6.71	6.11	5.42	4.73	4.03	3.35	2.78	1.91	0.7	0
1604	3.24	4.39	6.56	6.71	6.11	5.43	4.73	4.03	3.42	2.77	2.11	1.45	0.3
1605	3.24	4.46	6.61	6.71	6.11	5.42	4.73	4.02	3.41	2.83	2.37	1.85	1.23
1606	3.24	4.47	6.63	6.71	6.1	5.41	4.71	4.02	3.39	2.79	2.23	1.18	1.08
1607	3.24	4.49	6.64	6.7	6.1	5.41	4.72	4.03	3.4	2.89	2.26	1.67	0
1608	3.24	4.42	6.58	6.71	6.11	5.42	4.74	4.04	3.48	2.78	2.23	1.85	1.11
1609	3.24	4.78	6.85	6.88	6.21	5.43	4.64	3.86	3.21	2.47	1.69	0	0
1610	3.24	4.75	6.85	6.88	6.21	5.43	4.65	3.88	3.15	2.5	1.6	0	0
1611	3.24	4.75	6.85	6.88	6.21	5.44	4.65	3.87	3.25	2.66	1.45	0	0

1612	3.24	4.73	6.85	6.88	6.21	5.44	4.67	3.87	3.24	2.5	2.14	1.52	0
1613	3.24	4.76	6.85	6.88	6.21	5.44	4.65	3.86	3.19	2.65	1.85	0	0
1614	3.24	4.74	6.85	6.88	6.21	5.44	4.66	3.86	3.23	2.46	1.81	1.46	0
1615	3.24	4.8	6.89	7.07	6.92	6.74	6.57	6.39	6.23	6.08	5.92	5.76	5.61
1616	3.24	4.75	6.88	7.07	6.92	6.74	6.57	6.39	6.23	6.08	5.92	5.77	5.61
1617	3.24	4.77	6.88	7.07	6.92	6.74	6.56	6.39	6.23	6.07	5.92	5.76	5.6
1618	3.24	4.77	6.88	7.07	6.92	6.74	6.57	6.39	6.23	6.08	5.92	5.77	5.61
1619	3.24	4.82	6.89	7.07	6.92	6.74	6.57	6.39	6.23	6.08	5.92	5.76	5.61
1620	3.24	4.77	6.88	7.07	6.92	6.74	6.57	6.39	6.23	6.08	5.92	5.76	5.61
1621	3.24	4.96	7	7.29	7.08	6.67	6.24	5.81	5.44	5.07	4.69	4.31	3.97
1622	3.24	4.97	7	7.29	7.07	6.67	6.24	5.82	5.45	5.07	4.68	4.28	3.88
1623	3.24	4.99	7.01	7.29	7.07	6.66	6.24	5.81	5.44	5.08	4.72	4.35	4.01
1624	3.24	4.98	7.01	7.29	7.07	6.66	6.24	5.81	5.43	5.05	4.67	4.26	3.83
1625	3.24	4.99	7.01	7.29	7.07	6.67	6.24	5.81	5.44	5.06	4.69	4.32	3.93
1626	3.24	4.97	7	7.29	7.07	6.66	6.24	5.81	5.43	5.05	4.67	4.3	3.94
1627	3.24	4.76	6.87	6.99	6.62	6.19	5.77	5.34	4.96	4.59	4.2	3.79	3.48
1628	3.24	4.78	6.88	6.99	6.62	6.19	5.76	5.33	4.96	4.59	4.2	3.83	3.44
1629	3.24	4.73	6.87	6.99	6.63	6.2	5.77	5.34	4.96	4.58	4.21	3.85	3.5
1630	3.24	4.79	6.88	6.99	6.62	6.19	5.76	5.34	4.97	4.59	4.21	3.84	3.46
1631	3.24	4.73	6.87	6.99	6.63	6.2	5.77	5.34	4.97	4.59	4.22	3.84	3.46
1632	3.24	4.75	6.87	6.99	6.62	6.19	5.76	5.33	4.96	4.6	4.22	3.83	3.42
1633	3.24	4.45	6.67	6.79	6.42	5.99	5.56	5.13	4.76	4.38	4.01	3.69	3.3
1634	3.24	4.61	6.78	6.78	6.41	5.98	5.55	5.12	4.74	4.34	3.99	3.6	3.25
1635	3.24	4.43	6.65	6.79	6.42	5.99	5.57	5.14	4.76	4.38	3.99	3.61	3.22
1636	3.24	4.54	6.74	6.78	6.41	5.98	5.55	5.13	4.75	4.39	4.01	3.64	3.27
1637	3.24	4.51	6.7	6.79	6.41	5.99	5.55	5.12	4.75	4.37	3.98	3.59	3.17
1638	3.24	4.56	6.75	6.78	6.41	5.98	5.55	5.13	4.75	4.38	4	3.64	3.29
1639	3.24	4.76	6.86	6.9	6.32	5.63	4.94	4.22	3.62	3.1	2.57	1.75	0.48
1640	3.24	4.74	6.86	6.9	6.32	5.63	4.95	4.26	3.66	3.02	2.46	2.01	1.63
1641	3.24	4.79	6.87	6.9	6.31	5.62	4.94	4.25	3.64	3.04	2.48	2.05	1.52
1642	3.24	4.76	6.86	6.9	6.32	5.63	4.94	4.24	3.62	3.02	2.35	1.75	0.3
1643	3.24	4.73	6.86	6.91	6.32	5.64	4.94	4.26	3.64	2.97	2.42	1.76	0
1644	3.24	4.73	6.86	6.9	6.32	5.63	4.94	4.24	3.62	3.01	2.29	1.82	1.46
1645	3.24	4.86	6.9	6.98	6.62	6.19	5.76	5.34	4.96	4.58	4.21	3.85	3.48
1646	3.24	4.76	6.88	6.99	6.62	6.19	5.77	5.33	4.95	4.57	4.2	3.84	3.45
1647	3.24	4.88	6.9	6.98	6.62	6.19	5.76	5.33	4.95	4.57	4.2	3.82	3.45
1648	3.24	4.8	6.88	6.99	6.62	6.19	5.76	5.33	4.95	4.58	4.2	3.85	3.49
1649	3.24	4.83	6.89	6.98	6.62	6.2	5.77	5.34	4.96	4.6	4.23	3.88	3.44
1650	3.24	4.77	6.88	6.99	6.62	6.19	5.76	5.33	4.96	4.58	4.22	3.81	3.45
1651	3.24	3.61	4.62	6.14	6.87	6.44	6.01	5.58	5.2	4.82	4.42	4.04	3.66
1652	3.24	3.68	4.73	6.23	6.86	6.43	6	5.57	5.19	4.82	4.45	4.08	3.68
1653	3.24	3.72	4.78	6.28	6.85	6.42	6	5.57	5.19	4.81	4.44	4.07	3.7
1654	3.24	3.7	4.73	6.24	6.86	6.43	6	5.57	5.19	4.82	4.43	4.04	3.66
1655	3.24	3.64	4.67	6.18	6.86	6.44	6.01	5.58	5.2	4.82	4.44	4.06	3.75
1656	3.24	3.7	4.72	6.22	6.86	6.43	6	5.58	5.2	4.83	4.45	4.08	3.68
1657	3.24	4.28	6	7.07	6.95	6.77	6.59	6.41	6.26	6.1	5.95	5.79	5.64
1658	3.24	4.24	5.98	7.07	6.95	6.77	6.59	6.42	6.26	6.1	5.95	5.79	5.64
1659	3.24	4.3	6.04	7.07	6.95	6.77	6.59	6.42	6.26	6.1	5.95	5.79	5.64
1660	3.24	4.27	6.01	7.07	6.95	6.77	6.59	6.41	6.26	6.1	5.94	5.79	5.63
1661	3.24	4.32	6.05	7.07	6.95	6.77	6.59	6.41	6.26	6.1	5.95	5.79	5.64
1662	3.24	4.27	6.01	7.07	6.95	6.77	6.59	6.41	6.26	6.1	5.95	5.79	5.63
1663	3.24	4.5	6.32	7.27	7.12	6.72	6.3	5.87	5.49	5.11	4.72	4.35	3.97
1664	3.24	4.44	6.26	7.27	7.12	6.72	6.3	5.87	5.49	5.12	4.76	4.37	3.99
1665	3.24	4.53	6.35	7.27	7.12	6.72	6.29	5.86	5.48	5.11	4.74	4.38	4.03

1666	3.24	4.5	6.31	7.27	7.12	6.72	6.29	5.87	5.49	5.12	4.74	4.37	4.01
1667	3.24	4.52	6.33	7.27	7.12	6.72	6.29	5.86	5.49	5.12	4.74	4.39	4.02
1668	3.24	4.51	6.33	7.27	7.12	6.72	6.29	5.86	5.49	5.11	4.74	4.37	3.99
1669	3.24	4.28	5.99	7.02	6.69	6.26	5.83	5.4	5.03	4.65	4.27	3.87	3.51
1670	3.24	4.3	6.02	7.02	6.68	6.26	5.83	5.4	5.02	4.65	4.29	3.91	3.56
1671	3.24	4.21	5.94	7.02	6.69	6.26	5.83	5.41	5.04	4.66	4.28	3.89	3.52
1672	3.24	4.27	6	7.02	6.69	6.26	5.83	5.41	5.04	4.65	4.29	3.92	3.55
1673	3.24	4.28	6	7.02	6.69	6.26	5.83	5.41	5.04	4.66	4.32	3.94	3.56
1674	3.24	4.28	6	7.02	6.69	6.26	5.83	5.4	5.02	4.65	4.27	3.92	3.56
1675	3.24	4.01	5.65	6.85	6.49	6.06	5.62	5.19	4.81	4.44	4.06	3.7	3.34
1676	3.24	3.95	5.58	6.86	6.49	6.06	5.63	5.21	4.84	4.48	4.11	3.73	3.35
1677	3.24	3.88	5.51	6.86	6.5	6.07	5.64	5.2	4.83	4.46	4.1	3.74	3.38
1678	3.24	4.03	5.66	6.85	6.49	6.06	5.63	5.21	4.84	4.47	4.07	3.67	3.29
1679	3.24	4.02	5.65	6.85	6.49	6.06	5.63	5.2	4.82	4.45	4.07	3.7	3.3
1680	3.24	3.93	5.56	6.86	6.49	6.07	5.64	5.21	4.83	4.46	4.08	3.7	3.32
1681	3.24	4.24	5.96	6.97	6.42	5.74	5.05	4.34	3.71	3.09	2.6	1.76	0.85
1682	3.24	4.27	5.98	6.97	6.42	5.73	5.05	4.35	3.76	3.17	2.43	1.83	1.45
1683	3.24	4.23	5.94	6.97	6.43	5.74	5.05	4.36	3.73	3.19	2.52	1.99	1.85
1684	3.24	4.23	5.94	6.97	6.43	5.74	5.05	4.36	3.77	3.16	2.56	1.89	1.28
1685	3.24	4.25	5.96	6.97	6.42	5.73	5.04	4.34	3.74	3.08	2.17	1.6	0
1686	3.24	4.25	5.95	6.97	6.42	5.74	5.04	4.36	3.79	3.22	2.67	2.12	0
1687	3.24	4.61	6.72	7	6.64	6.21	5.78	5.34	4.97	4.6	4.23	3.89	3.49
1688	3.24	4.59	6.71	7	6.64	6.21	5.78	5.36	4.98	4.61	4.24	3.86	3.41
1689	3.24	4.64	6.74	7	6.64	6.21	5.78	5.35	4.98	4.59	4.21	3.84	3.41
1690	3.24	4.67	6.77	7	6.64	6.21	5.78	5.35	4.97	4.59	4.22	3.82	3.45
1691	3.24	4.63	6.74	7	6.64	6.21	5.78	5.35	4.97	4.6	4.23	3.86	3.45
1692	3.24	4.67	6.76	7	6.64	6.21	5.78	5.35	4.97	4.59	4.21	3.84	3.45
1693	3.24	4.27	5.99	7.02	6.69	6.26	5.83	5.41	5.03	4.65	4.26	3.85	3.43
1694	3.24	4.22	5.94	7.02	6.69	6.26	5.84	5.4	5.02	4.66	4.28	3.9	3.58
1695	3.24	4.27	6	7.02	6.69	6.26	5.83	5.41	5.03	4.66	4.28	3.91	3.52
1696	3.24	4.25	5.98	7.02	6.69	6.26	5.83	5.41	5.04	4.65	4.27	3.9	3.52
1697	3.24	4.3	6.03	7.02	6.69	6.26	5.83	5.4	5.03	4.65	4.27	3.89	3.48
1698	3.24	4.23	5.95	7.02	6.69	6.26	5.83	5.41	5.03	4.66	4.27	3.9	3.53
1699	3.24	3.12	3.6	4.29	5.84	6.89	6.59	6.17	5.8	5.42	5.05	4.67	4.3
1700	3.24	3.27	3.8	4.5	6.03	6.89	6.54	6.12	5.75	5.38	5.01	4.63	4.24
1701	3.24	3.22	3.74	4.46	6	6.89	6.55	6.13	5.75	5.38	5	4.64	4.26
1702	3.24	3.2	3.72	4.47	6.01	6.89	6.55	6.12	5.75	5.38	5	4.63	4.26
1703	3.24	3.2	3.74	4.46	6	6.89	6.55	6.13	5.76	5.38	5.01	4.64	4.26
1704	3.24	3.21	3.7	4.39	5.94	6.89	6.57	6.14	5.77	5.39	5.02	4.65	4.27
1705	3.24	3.47	4.21	5.26	7.03	6.92	6.74	6.57	6.41	6.25	6.1	5.94	5.79
1706	3.24	3.37	4.05	5.11	7	6.93	6.76	6.58	6.42	6.26	6.11	5.95	5.79
1707	3.24	3.36	4.11	5.16	7.01	6.93	6.75	6.57	6.42	6.26	6.11	5.95	5.8
1708	3.24	3.41	4.1	5.14	7.01	6.93	6.75	6.57	6.42	6.26	6.11	5.95	5.79
1709	3.24	3.41	4.1	5.18	7.02	6.93	6.75	6.57	6.42	6.26	6.1	5.95	5.79
1710	3.24	3.46	4.2	5.25	7.03	6.92	6.74	6.57	6.41	6.25	6.1	5.95	5.79
1711	3.24	3.7	4.55	5.76	7.28	7	6.58	6.16	5.79	5.42	5.04	4.69	4.34
1712	3.24	3.7	4.52	5.74	7.28	7	6.59	6.16	5.79	5.41	5.03	4.65	4.29
1713	3.24	3.63	4.43	5.63	7.28	7.01	6.6	6.18	5.8	5.43	5.06	4.7	4.31
1714	3.24	3.65	4.48	5.7	7.28	7	6.59	6.16	5.79	5.41	5.03	4.63	4.24
1715	3.24	3.62	4.44	5.64	7.28	7.01	6.6	6.18	5.8	5.42	5.04	4.67	4.29
1716	3.24	3.59	4.4	5.61	7.28	7.02	6.6	6.18	5.8	5.43	5.05	4.66	4.31
1717	3.24	3.37	4.06	5.06	6.91	6.67	6.24	5.82	5.44	5.06	4.68	4.32	3.96
1718	3.24	3.32	4.05	5.05	6.91	6.67	6.24	5.81	5.44	5.06	4.67	4.29	3.92
1719	3.24	3.44	4.15	5.13	6.93	6.66	6.23	5.8	5.43	5.06	4.68	4.29	3.92

1720	3.24	3.4	4.09	5.09	6.92	6.67	6.24	5.81	5.43	5.06	4.69	4.31	3.94
1721	3.24	3.4	4.11	5.1	6.92	6.66	6.24	5.81	5.44	5.06	4.69	4.32	3.96
1722	3.24	3.4	4.12	5.13	6.93	6.66	6.23	5.8	5.43	5.06	4.69	4.31	3.95
1723	3.24	3.03	3.67	4.51	6.27	6.61	6.2	5.77	5.4	5.02	4.65	4.28	3.91
1724	3.24	3.07	3.66	4.48	6.26	6.62	6.2	5.78	5.4	5.03	4.66	4.29	3.89
1725	3.24	2.99	3.62	4.47	6.24	6.62	6.21	5.78	5.41	5.04	4.67	4.3	3.91
1726	3.24	3.21	3.81	4.61	6.34	6.6	6.18	5.75	5.38	5.01	4.64	4.25	3.89
1727	3.24	3.06	3.68	4.52	6.29	6.61	6.19	5.77	5.39	5.02	4.65	4.27	3.92
1728	3.24	3.07	3.67	4.48	6.26	6.62	6.2	5.77	5.4	5.03	4.66	4.27	3.9
1729	3.24	3.48	4.13	5.09	6.83	6.42	5.74	5.05	4.45	3.81	3.18	2.59	1.84
1730	3.24	3.45	4.12	5.07	6.83	6.42	5.74	5.05	4.44	3.81	3.21	2.62	2.13
1731	3.24	3.43	4.08	5.03	6.82	6.43	5.75	5.06	4.45	3.83	3.24	2.67	2.06
1732	3.24	3.37	4.06	5.02	6.81	6.44	5.75	5.07	4.47	3.89	3.29	2.75	1.96
1733	3.24	3.39	4.05	5	6.81	6.44	5.76	5.07	4.46	3.89	3.33	2.78	2.19
1734	3.24	3.36	3.98	4.94	6.79	6.46	5.78	5.09	4.5	3.91	3.28	2.64	2.14
1735	3.24	3.7	4.78	6.34	7.07	6.98	6.88	6.79	6.71	6.62	6.54	6.46	6.38
1736	3.24	3.72	4.79	6.34	7.07	6.98	6.88	6.79	6.7	6.62	6.54	6.46	6.37
1737	3.24	3.71	4.79	6.34	7.07	6.98	6.88	6.79	6.71	6.62	6.54	6.46	6.38
1738	3.24	3.7	4.78	6.33	7.07	6.98	6.88	6.79	6.71	6.62	6.54	6.46	6.37
1739	3.24	3.76	4.83	6.38	7.07	6.98	6.88	6.79	6.71	6.62	6.54	6.46	6.37
1740	3.24	3.68	4.76	6.32	7.07	6.98	6.88	6.79	6.71	6.62	6.54	6.46	6.38
1741	3.24	3.95	5.15	6.81	7.37	7.23	7.05	6.87	6.72	6.56	6.41	6.25	6.09
1742	3.24	3.88	5.07	6.75	7.37	7.23	7.06	6.88	6.72	6.57	6.41	6.26	6.1
1743	3.24	3.95	5.16	6.83	7.37	7.23	7.05	6.87	6.72	6.56	6.41	6.25	6.09
1744	3.24	3.93	5.11	6.78	7.37	7.23	7.05	6.88	6.72	6.56	6.41	6.25	6.09
1745	3.24	3.95	5.15	6.82	7.37	7.23	7.05	6.87	6.72	6.56	6.41	6.25	6.09
1746	3.24	3.98	5.18	6.84	7.37	7.23	7.05	6.87	6.72	6.56	6.41	6.25	6.1
1747	3.24	3.74	4.8	6.35	7.01	6.84	6.66	6.48	6.32	6.17	6.01	5.85	5.7
1748	3.24	3.7	4.77	6.31	7.02	6.84	6.66	6.48	6.33	6.17	6.01	5.86	5.7
1749	3.24	3.7	4.76	6.29	7.02	6.84	6.66	6.48	6.33	6.17	6.02	5.87	5.71
1750	3.24	3.75	4.81	6.35	7.01	6.84	6.66	6.48	6.32	6.17	6.01	5.86	5.7
1751	3.24	3.67	4.75	6.29	7.02	6.84	6.66	6.48	6.33	6.17	6.02	5.86	5.7
1752	3.24	3.71	4.76	6.3	7.02	6.84	6.66	6.48	6.33	6.17	6.02	5.86	5.7
1753	3.24	3.51	4.46	5.84	6.83	6.65	6.48	6.3	6.14	5.99	5.83	5.68	5.52
1754	3.24	3.36	4.33	5.72	6.84	6.66	6.48	6.31	6.15	6	5.84	5.68	5.53
1755	3.24	3.48	4.45	5.83	6.83	6.65	6.48	6.3	6.14	5.99	5.83	5.67	5.52
1756	3.24	3.45	4.37	5.75	6.84	6.66	6.48	6.3	6.15	5.99	5.84	5.68	5.53
1757	3.24	3.46	4.39	5.78	6.84	6.66	6.48	6.3	6.15	5.99	5.83	5.68	5.52
1758	3.24	3.38	4.28	5.68	6.84	6.66	6.48	6.31	6.15	6	5.84	5.68	5.53
1759	3.24	3.98	5.21	6.9	7.45	7.22	6.86	6.47	6.12	5.76	5.39	5.02	4.64
1760	3.24	4.03	5.24	6.92	7.45	7.21	6.86	6.47	6.11	5.75	5.38	5	4.62
1761	3.24	4.07	5.29	6.95	7.45	7.21	6.86	6.46	6.11	5.74	5.37	4.99	4.61
1762	3.24	4.09	5.32	6.97	7.45	7.21	6.85	6.46	6.1	5.74	5.37	5.01	4.66
1763	3.24	4.01	5.23	6.91	7.45	7.21	6.86	6.47	6.11	5.74	5.37	5	4.62
1764	3.24	4.04	5.27	6.94	7.45	7.21	6.86	6.47	6.11	5.75	5.38	5.01	4.64
1765	3.24	3.99	5.17	6.8	7.22	6.85	6.42	5.99	5.62	5.24	4.87	4.5	4.13
1766	3.24	3.94	5.1	6.75	7.22	6.85	6.43	6	5.62	5.25	4.86	4.47	4.1
1767	3.24	3.99	5.17	6.8	7.22	6.85	6.42	5.99	5.62	5.24	4.86	4.48	4.11
1768	3.24	3.9	5.07	6.72	7.23	6.86	6.43	6	5.63	5.26	4.87	4.51	4.15
1769	3.24	3.97	5.15	6.78	7.22	6.85	6.42	5.99	5.62	5.25	4.87	4.51	4.1
1770	3.24	3.95	5.16	6.79	7.22	6.85	6.42	6	5.63	5.26	4.89	4.52	4.16
1771	3.24	3.68	4.73	6.24	6.86	6.43	6	5.58	5.2	4.82	4.46	4.09	3.7
1772	3.24	3.68	4.73	6.23	6.86	6.43	6	5.57	5.19	4.81	4.44	4.06	3.65
1773	3.24	3.65	4.7	6.21	6.86	6.43	6	5.58	5.21	4.83	4.46	4.08	3.72

1774	3.24	3.6	4.65	6.16	6.87	6.44	6.01	5.58	5.21	4.84	4.47	4.08	3.75
1775	3.24	3.68	4.73	6.23	6.86	6.43	6.01	5.58	5.21	4.84	4.46	4.09	3.7
1776	3.24	3.74	4.79	6.29	6.85	6.42	5.99	5.57	5.19	4.8	4.43	4.03	3.66
1777	3.24	3.42	4.32	5.66	6.71	6.28	5.85	5.42	5.05	4.67	4.28	3.91	3.54
1778	3.24	3.42	4.32	5.66	6.71	6.28	5.85	5.42	5.04	4.67	4.31	3.93	3.56
1779	3.24	3.36	4.28	5.62	6.71	6.29	5.86	5.43	5.05	4.68	4.31	3.92	3.56
1780	3.24	3.31	4.2	5.56	6.72	6.29	5.86	5.43	5.06	4.69	4.33	3.98	3.65
1781	3.24	3.46	4.39	5.74	6.7	6.27	5.84	5.42	5.03	4.66	4.28	3.89	3.51
1782	3.24	3.41	4.32	5.65	6.71	6.28	5.85	5.42	5.06	4.68	4.3	3.93	3.56
1783	3.24	3.15	4.01	5.25	6.71	6.28	5.85	5.42	5.05	4.67	4.29	3.92	3.54
1784	3.24	3.33	4.25	5.53	6.67	6.25	5.82	5.39	5.02	4.65	4.27	3.87	3.5
1785	3.24	3.35	4.21	5.49	6.68	6.25	5.82	5.39	5.02	4.65	4.26	3.9	3.55
1786	3.24	3.4	4.28	5.55	6.67	6.24	5.81	5.38	5.01	4.63	4.26	3.88	3.53
1787	3.24	3.24	4.16	5.45	6.68	6.25	5.83	5.4	5.02	4.64	4.26	3.88	3.5
1788	3.24	3.38	4.31	5.59	6.67	6.24	5.81	5.39	5.02	4.64	4.27	3.91	3.55
1789	3.24	3.9	5.04	6.67	7.09	6.5	5.83	5.14	4.54	3.92	3.28	2.72	2.28
1790	3.24	4.01	5.16	6.76	7.07	6.48	5.81	5.11	4.5	3.9	3.27	2.71	1.6
1791	3.24	3.9	5.04	6.67	7.08	6.49	5.82	5.14	4.55	3.98	3.43	2.85	2.2
1792	3.24	3.9	5.07	6.7	7.08	6.49	5.81	5.12	4.52	3.94	3.28	2.56	1.75
1793	3.24	3.93	5.08	6.7	7.08	6.49	5.82	5.14	4.53	3.91	3.26	2.57	2.03
1794	3.24	3.93	5.08	6.7	7.08	6.49	5.82	5.13	4.52	3.96	3.4	2.77	1.87
1795	3.24	3.72	4.74	6.2	6.7	6.02	5.33	4.62	4.02	3.45	2.91	2.3	1.76
1796	3.24	3.67	4.68	6.14	6.71	6.02	5.34	4.64	4.04	3.43	2.78	2.23	1.99
1797	3.24	3.71	4.73	6.2	6.7	6.01	5.32	4.63	4.04	3.5	3	2.28	1.73
1798	3.24	3.71	4.76	6.22	6.7	6.01	5.32	4.63	4.07	3.49	2.94	2.39	1.66
1799	3.24	3.67	4.69	6.16	6.71	6.02	5.34	4.64	4.03	3.42	2.91	2.35	1.08
1800	3.24	3.67	4.69	6.16	6.71	6.02	5.33	4.66	4.05	3.47	2.82	2.39	2.13
1801	3.24	3.42	4.25	5.55	6.6	5.92	5.23	4.54	3.95	3.35	2.86	2.18	1.46
1802	3.24	3.26	4.16	5.46	6.62	5.94	5.25	4.56	3.97	3.36	2.91	2.08	0.48
1803	3.24	3.29	4.22	5.5	6.61	5.93	5.24	4.54	3.96	3.4	2.89	2.22	1.46
1804	3.24	3.33	4.21	5.52	6.61	5.92	5.24	4.54	3.87	3.28	2.63	2.14	1.61
1805	3.24	3.38	4.31	5.6	6.59	5.91	5.22	4.53	3.94	3.36	2.75	2.17	1.78
1806	3.24	3.5	4.37	5.65	6.58	5.9	5.21	4.54	3.92	3.32	2.69	2.14	1.28
1807	3.24	3.72	4.7	6.15	6.66	5.88	5.11	4.34	3.64	2.89	2.3	1.2	0
1808	3.24	3.6	4.61	6.09	6.67	5.89	5.11	4.33	3.67	2.93	2.34	1.6	0
1809	3.24	3.81	4.81	6.25	6.63	5.86	5.07	4.28	3.63	3.04	2.43	1.84	0.9
1810	3.24	3.71	4.7	6.15	6.65	5.88	5.1	4.33	3.67	2.97	2.19	0	0
1811	3.24	3.74	4.74	6.19	6.65	5.87	5.09	4.33	3.61	2.92	2.14	0.6	0
1812	3.24	3.63	4.61	6.07	6.67	5.89	5.13	4.34	3.64	3	2.49	1.98	0.6
1813	3.24	4.03	5.39	6.97	6.75	6.32	5.89	5.47	5.09	4.73	4.36	4	3.66
1814	3.24	3.95	5.31	6.95	6.75	6.33	5.9	5.47	5.09	4.72	4.35	3.97	3.59
1815	3.24	3.93	5.28	6.95	6.76	6.33	5.9	5.48	5.11	4.74	4.36	3.99	3.61
1816	3.24	3.98	5.32	6.96	6.76	6.33	5.9	5.48	5.11	4.74	4.37	3.98	3.62
1817	3.24	3.94	5.31	6.95	6.76	6.33	5.9	5.47	5.08	4.71	4.33	3.95	3.54
1818	3.24	3.91	5.28	6.95	6.76	6.33	5.9	5.47	5.1	4.72	4.34	3.95	3.6
1819	3.24	4.3	6.05	7.1	7.08	7.02	6.96	6.9	6.85	6.8	6.75	6.7	6.65
1820	3.24	4.2	5.95	7.1	7.08	7.02	6.96	6.9	6.85	6.8	6.75	6.7	6.65
1821	3.24	4.24	6	7.1	7.08	7.02	6.96	6.9	6.85	6.8	6.75	6.7	6.65
1822	3.24	4.23	5.99	7.1	7.08	7.02	6.96	6.9	6.85	6.8	6.75	6.7	6.65
1823	3.24	4.3	6.05	7.1	7.07	7.02	6.96	6.9	6.85	6.8	6.75	6.7	6.65
1824	3.24	4.23	5.97	7.1	7.08	7.02	6.96	6.9	6.85	6.8	6.75	6.7	6.65
1825	3.24	4.47	6.32	7.33	7.42	7.33	7.24	7.14	7.06	6.98	6.9	6.81	6.73
1826	3.24	4.49	6.33	7.33	7.42	7.33	7.24	7.14	7.06	6.98	6.9	6.81	6.73
1827	3.24	4.48	6.33	7.33	7.42	7.33	7.24	7.14	7.06	6.98	6.9	6.81	6.73

1828	3.24	4.49	6.33	7.33	7.42	7.33	7.24	7.14	7.06	6.98	6.9	6.81	6.73
1829	3.24	4.5	6.34	7.33	7.42	7.33	7.24	7.14	7.06	6.98	6.9	6.81	6.73
1830	3.24	4.53	6.37	7.33	7.42	7.33	7.24	7.14	7.06	6.98	6.9	6.81	6.73
1831	3.24	4.25	6	7.09	7.04	6.94	6.85	6.75	6.67	6.59	6.51	6.43	6.34
1832	3.24	4.23	5.98	7.09	7.04	6.94	6.85	6.75	6.67	6.59	6.51	6.42	6.34
1833	3.24	4.27	6.03	7.09	7.04	6.94	6.85	6.75	6.67	6.59	6.51	6.42	6.34
1834	3.24	4.26	6.01	7.09	7.04	6.94	6.85	6.75	6.67	6.59	6.51	6.42	6.34
1835	3.24	4.26	6.01	7.09	7.04	6.94	6.85	6.75	6.67	6.59	6.51	6.42	6.34
1836	3.24	4.22	5.98	7.09	7.04	6.94	6.85	6.75	6.67	6.59	6.51	6.43	6.34
1837	3.24	4	5.65	6.92	6.84	6.75	6.65	6.56	6.47	6.39	6.31	6.23	6.15
1838	3.24	3.94	5.58	6.92	6.84	6.75	6.65	6.56	6.48	6.39	6.31	6.23	6.15
1839	3.24	4.06	5.7	6.92	6.84	6.74	6.65	6.56	6.47	6.39	6.31	6.23	6.14
1840	3.24	3.99	5.65	6.92	6.84	6.74	6.65	6.56	6.47	6.39	6.31	6.23	6.14
1841	3.24	3.93	5.56	6.92	6.84	6.75	6.65	6.56	6.47	6.39	6.31	6.23	6.14
1842	3.24	3.96	5.6	6.92	6.84	6.75	6.65	6.56	6.47	6.39	6.31	6.23	6.14
1843	3.24	4.59	6.45	7.42	7.58	7.48	7.33	7.15	7	6.85	6.69	6.53	6.38
1844	3.24	4.55	6.42	7.42	7.58	7.49	7.33	7.16	7	6.85	6.69	6.54	6.38
1845	3.24	4.56	6.43	7.42	7.58	7.48	7.33	7.16	7	6.85	6.69	6.53	6.38
1846	3.24	4.53	6.4	7.41	7.58	7.49	7.33	7.16	7	6.85	6.69	6.54	6.38
1847	3.24	4.58	6.45	7.42	7.58	7.48	7.33	7.15	7	6.85	6.69	6.53	6.38
1848	3.24	4.52	6.39	7.41	7.58	7.49	7.33	7.16	7	6.85	6.69	6.54	6.38
1849	3.24	4.51	6.35	7.32	7.34	7.17	7	6.82	6.66	6.51	6.35	6.2	6.04
1850	3.24	4.46	6.3	7.31	7.34	7.18	7	6.82	6.67	6.51	6.36	6.2	6.05
1851	3.24	4.5	6.34	7.32	7.34	7.18	7	6.82	6.67	6.51	6.35	6.2	6.04
1852	3.24	4.5	6.33	7.31	7.34	7.17	7	6.82	6.66	6.5	6.35	6.19	6.04
1853	3.24	4.52	6.35	7.32	7.34	7.17	7	6.82	6.66	6.51	6.35	6.19	6.04
1854	3.24	4.45	6.3	7.31	7.34	7.18	7	6.82	6.67	6.51	6.35	6.2	6.04
1855	3.24	4.24	5.98	7.07	6.95	6.77	6.59	6.42	6.26	6.1	5.95	5.79	5.64
1856	3.24	4.29	6.02	7.07	6.95	6.77	6.59	6.41	6.26	6.1	5.95	5.79	5.64
1857	3.24	4.28	6.02	7.07	6.95	6.77	6.59	6.42	6.26	6.1	5.95	5.79	5.63
1858	3.24	4.24	5.97	7.07	6.95	6.77	6.59	6.42	6.26	6.11	5.95	5.79	5.64
1859	3.24	4.24	5.98	7.07	6.95	6.77	6.59	6.42	6.26	6.11	5.95	5.79	5.63
1860	3.24	4.25	5.99	7.07	6.95	6.77	6.59	6.42	6.26	6.1	5.95	5.79	5.64
1861	3.24	3.93	5.57	6.9	6.75	6.58	6.4	6.22	6.07	5.91	5.75	5.6	5.44
1862	3.24	3.97	5.6	6.9	6.75	6.57	6.4	6.22	6.06	5.91	5.75	5.59	5.43
1863	3.24	4.03	5.67	6.9	6.75	6.57	6.4	6.22	6.06	5.91	5.75	5.6	5.44
1864	3.24	3.99	5.63	6.9	6.75	6.57	6.39	6.22	6.06	5.9	5.75	5.59	5.43
1865	3.24	3.94	5.58	6.9	6.75	6.58	6.4	6.22	6.06	5.91	5.75	5.6	5.44
1866	3.24	3.97	5.61	6.9	6.75	6.57	6.4	6.22	6.07	5.91	5.76	5.6	5.44
1867	3.24	3.99	5.61	6.85	6.7	6.52	6.34	6.16	6.01	5.85	5.69	5.54	5.38
1868	3.24	3.86	5.48	6.85	6.7	6.52	6.35	6.17	6.01	5.86	5.7	5.55	5.39
1869	3.24	3.95	5.55	6.85	6.7	6.52	6.35	6.17	6.01	5.85	5.7	5.54	5.39
1870	3.24	3.89	5.48	6.85	6.7	6.52	6.35	6.17	6.02	5.86	5.7	5.55	5.39
1871	3.24	3.87	5.47	6.85	6.7	6.52	6.35	6.17	6.02	5.86	5.7	5.55	5.4
1872	3.24	3.7	5.32	6.85	6.71	6.53	6.35	6.17	6.02	5.86	5.7	5.55	5.39
1873	3.24	4.27	6	7.05	6.84	6.56	6.28	6	5.76	5.52	5.27	5.03	4.78
1874	3.24	4.31	6.04	7.05	6.84	6.56	6.28	6	5.76	5.52	5.27	5.02	4.77
1875	3.24	4.27	6.01	7.05	6.84	6.56	6.28	6	5.76	5.51	5.27	5.02	4.77
1876	3.24	4.27	6.01	7.05	6.84	6.56	6.28	6	5.76	5.52	5.27	5.02	4.78
1877	3.24	4.26	6	7.05	6.84	6.56	6.28	6	5.76	5.51	5.27	5.02	4.78
1878	3.24	4.26	5.99	7.05	6.84	6.56	6.28	6	5.76	5.51	5.27	5.02	4.78
1879	3.24	4.59	6.46	7.42	7.56	7.39	7.12	6.81	6.52	6.22	5.9	5.58	5.25
1880	3.24	4.59	6.45	7.42	7.56	7.39	7.12	6.81	6.52	6.22	5.91	5.59	5.27
1881	3.24	4.58	6.45	7.42	7.56	7.39	7.12	6.81	6.52	6.22	5.9	5.58	5.26

1882	3.24	4.6	6.47	7.42	7.56	7.39	7.12	6.81	6.52	6.22	5.91	5.59	5.26
1883	3.24	4.57	6.43	7.42	7.56	7.39	7.12	6.81	6.52	6.22	5.91	5.59	5.26
1884	3.24	4.6	6.47	7.42	7.56	7.39	7.12	6.81	6.52	6.22	5.9	5.59	5.26
1885	3.24	4.57	6.42	7.37	7.4	7.12	6.75	6.35	5.99	5.62	5.25	4.86	4.49
1886	3.24	4.56	6.41	7.37	7.4	7.12	6.75	6.36	6	5.63	5.27	4.9	4.51
1887	3.24	4.58	6.43	7.37	7.4	7.12	6.75	6.35	5.99	5.62	5.25	4.88	4.52
1888	3.24	4.52	6.37	7.37	7.41	7.12	6.75	6.35	5.99	5.62	5.25	4.87	4.48
1889	3.24	4.53	6.38	7.37	7.41	7.12	6.75	6.36	5.99	5.62	5.25	4.87	4.49
1890	3.24	4.57	6.42	7.37	7.4	7.12	6.75	6.35	5.99	5.62	5.25	4.89	4.53
1891	3.24	4.49	6.31	7.27	7.12	6.72	6.29	5.87	5.49	5.11	4.74	4.37	4
1892	3.24	4.51	6.32	7.27	7.12	6.72	6.3	5.87	5.49	5.12	4.75	4.37	3.99
1893	3.24	4.47	6.29	7.27	7.12	6.72	6.3	5.87	5.49	5.11	4.74	4.39	4.05
1894	3.24	4.48	6.29	7.27	7.12	6.72	6.3	5.87	5.49	5.11	4.74	4.37	3.99
1895	3.24	4.51	6.33	7.27	7.12	6.72	6.29	5.86	5.5	5.13	4.76	4.39	4.05
1896	3.24	4.44	6.27	7.27	7.12	6.72	6.3	5.87	5.5	5.12	4.75	4.36	3.97
1897	3.24	4.36	6.14	7.15	6.89	6.47	6.04	5.62	5.25	4.87	4.5	4.15	3.77
1898	3.24	4.36	6.14	7.16	6.89	6.47	6.04	5.6	5.22	4.84	4.44	4.05	3.64
1899	3.24	4.42	6.2	7.16	6.89	6.46	6.04	5.6	5.23	4.86	4.47	4.1	3.69
1900	3.24	4.43	6.21	7.16	6.89	6.46	6.03	5.6	5.22	4.84	4.46	4.07	3.71
1901	3.24	4.36	6.15	7.16	6.89	6.47	6.04	5.61	5.24	4.87	4.49	4.12	3.73
1902	3.24	4.4	6.18	7.16	6.89	6.46	6.03	5.6	5.22	4.85	4.48	4.1	3.71
1903	3.24	4.23	5.96	7.02	6.69	6.26	5.83	5.4	5.03	4.66	4.3	3.95	3.6
1904	3.24	4.26	5.97	7.02	6.69	6.26	5.83	5.4	5.03	4.66	4.28	3.9	3.53
1905	3.24	4.27	5.98	7.02	6.69	6.26	5.83	5.4	5.02	4.64	4.27	3.88	3.53
1906	3.24	4.17	5.89	7.02	6.69	6.27	5.83	5.4	5.03	4.64	4.26	3.87	3.48
1907	3.24	4.22	5.95	7.02	6.69	6.26	5.83	5.4	5.02	4.65	4.28	3.9	3.55
1908	3.24	4.22	5.95	7.02	6.69	6.26	5.84	5.41	5.03	4.65	4.28	3.9	3.52
1909	3.24	4.13	5.79	6.91	6.55	6.12	5.69	5.27	4.89	4.52	4.13	3.76	3.41
1910	3.24	4.12	5.79	6.92	6.56	6.13	5.7	5.27	4.89	4.51	4.14	3.74	3.38
1911	3.24	4.11	5.78	6.92	6.56	6.13	5.7	5.28	4.9	4.53	4.16	3.75	3.37
1912	3.24	4.18	5.84	6.91	6.55	6.12	5.69	5.26	4.89	4.51	4.13	3.73	3.33
1913	3.24	4.13	5.79	6.92	6.56	6.13	5.7	5.28	4.9	4.52	4.15	3.77	3.42
1914	3.24	4.05	5.72	6.92	6.56	6.13	5.7	5.27	4.89	4.52	4.15	3.75	3.37
1915	3.24	3.93	5.55	6.86	6.49	6.07	5.63	5.21	4.84	4.46	4.06	3.68	3.29
1916	3.24	4	5.62	6.86	6.49	6.06	5.64	5.21	4.84	4.46	4.08	3.7	3.35
1917	3.24	3.99	5.61	6.86	6.49	6.06	5.63	5.2	4.83	4.46	4.08	3.67	3.32
1918	3.24	3.94	5.57	6.86	6.49	6.06	5.64	5.21	4.83	4.46	4.09	3.73	3.3
1919	3.24	3.86	5.48	6.86	6.5	6.07	5.64	5.21	4.83	4.44	4.07	3.69	3.31
1920	3.24	4.02	5.64	6.85	6.49	6.06	5.63	5.21	4.83	4.46	4.08	3.7	3.36
1921	3.24	3.86	5.45	6.81	6.45	6.02	5.59	5.16	4.79	4.42	4.05	3.65	3.26
1922	3.24	3.85	5.44	6.81	6.45	6.02	5.59	5.16	4.78	4.41	4.05	3.71	3.35
1923	3.24	3.82	5.4	6.82	6.45	6.02	5.6	5.17	4.8	4.43	4.04	3.7	3.3
1924	3.24	3.87	5.44	6.81	6.45	6.02	5.59	5.16	4.79	4.42	4.05	3.67	3.3
1925	3.24	3.83	5.42	6.82	6.45	6.02	5.59	5.17	4.79	4.4	4.02	3.66	3.29
1926	3.24	3.77	5.35	6.82	6.45	6.03	5.6	5.17	4.79	4.41	4.04	3.68	3.28
1927	3.24	3.68	5.26	6.8	6.44	6.01	5.58	5.15	4.76	4.38	4	3.6	3.22
1928	3.24	3.88	5.44	6.8	6.43	6	5.57	5.15	4.76	4.38	3.99	3.62	3.2
1929	3.24	3.91	5.49	6.79	6.42	5.99	5.56	5.14	4.76	4.38	4	3.62	3.29
1930	3.24	3.77	5.33	6.8	6.43	6	5.58	5.15	4.78	4.4	4.01	3.6	3.19
1931	3.24	3.87	5.42	6.8	6.43	6	5.57	5.14	4.77	4.39	4.03	3.68	3.34
1932	3.24	3.81	5.41	6.8	6.43	6	5.57	5.14	4.76	4.39	4.02	3.62	3.29
1933	3.24	4.27	5.97	6.99	6.53	5.95	5.37	4.78	4.28	3.81	3.29	2.78	2.38
1934	3.24	4.23	5.93	6.99	6.53	5.95	5.38	4.8	4.31	3.8	3.33	2.67	2.1
1935	3.24	4.25	5.96	6.99	6.53	5.95	5.37	4.79	4.29	3.78	3.33	2.77	2.3

1936	3.24	4.27	5.98	6.99	6.53	5.95	5.37	4.8	4.29	3.78	3.27	2.75	2.09
1937	3.24	4.26	5.97	6.99	6.53	5.95	5.37	4.79	4.26	3.72	3.26	2.73	2.2
1938	3.24	4.27	5.97	6.99	6.53	5.95	5.37	4.78	4.27	3.8	3.37	2.84	2.25
1939	3.24	4.58	6.42	7.33	7.24	6.8	6.26	5.69	5.17	4.62	4.07	3.54	2.97
1940	3.24	4.56	6.4	7.33	7.24	6.8	6.27	5.69	5.17	4.63	4.09	3.55	2.96
1941	3.24	4.53	6.37	7.32	7.24	6.8	6.27	5.69	5.17	4.64	4.1	3.52	2.93
1942	3.24	4.54	6.37	7.32	7.24	6.8	6.27	5.69	5.17	4.63	4.1	3.58	3.02
1943	3.24	4.52	6.35	7.32	7.24	6.8	6.27	5.69	5.16	4.63	4.08	3.53	2.99
1944	3.24	4.56	6.4	7.33	7.24	6.8	6.27	5.69	5.16	4.62	4.08	3.54	3
1945	3.24	4.52	6.32	7.22	6.91	6.28	5.6	4.92	4.33	3.7	3.02	2.19	1.28
1946	3.24	4.43	6.24	7.22	6.91	6.28	5.6	4.93	4.33	3.74	3.17	2.31	1.79
1947	3.24	4.44	6.24	7.22	6.92	6.28	5.61	4.94	4.36	3.7	3.05	2.46	1.9
1948	3.24	4.46	6.27	7.22	6.91	6.28	5.6	4.92	4.3	3.71	3.04	2.45	1.49
1949	3.24	4.42	6.23	7.22	6.92	6.29	5.61	4.93	4.33	3.75	3.05	2.23	1.15
1950	3.24	4.49	6.29	7.22	6.91	6.28	5.6	4.91	4.32	3.77	3.25	2.64	2.12
1951	3.24	4.27	5.97	6.97	6.42	5.74	5.05	4.37	3.75	3.21	2.64	1.85	0.6
1952	3.24	4.26	5.96	6.97	6.42	5.73	5.04	4.37	3.79	3.14	2.56	2.04	0
1953	3.24	4.27	5.97	6.97	6.42	5.74	5.04	4.34	3.78	3.13	2.56	1.95	0.7
1954	3.24	4.2	5.9	6.98	6.43	5.73	5.06	4.37	3.73	3.08	2.32	1.71	0
1955	3.24	4.26	5.96	6.97	6.42	5.73	5.04	4.35	3.76	3.18	2.42	1.61	0.85
1956	3.24	4.2	5.9	6.98	6.43	5.74	5.05	4.36	3.77	3.23	2.65	2.11	1.4
1957	3.24	4	5.61	6.81	6.22	5.53	4.84	4.14	3.52	2.92	2.3	1.59	1.23
1958	3.24	3.91	5.52	6.82	6.23	5.54	4.85	4.16	3.57	2.96	2.48	1.82	1.43
1959	3.24	3.99	5.6	6.81	6.22	5.53	4.84	4.15	3.58	2.98	2.45	1.79	1.18
1960	3.24	3.96	5.56	6.82	6.23	5.54	4.84	4.16	3.51	2.89	2.21	1.46	1.26
1961	3.24	3.97	5.57	6.81	6.22	5.53	4.85	4.17	3.56	2.93	2.3	1.58	0
1962	3.24	3.9	5.5	6.82	6.23	5.54	4.85	4.18	3.61	3.03	2.44	1.96	1.77
1963	3.24	3.81	5.38	6.78	6.19	5.5	4.81	4.11	3.54	2.98	2.41	1.81	1.15
1964	3.24	3.8	5.35	6.78	6.19	5.5	4.81	4.12	3.53	2.98	2.35	1.6	1.15
1965	3.24	3.81	5.38	6.78	6.19	5.5	4.82	4.12	3.56	2.95	2.37	1.72	1.04
1966	3.24	3.82	5.38	6.78	6.19	5.5	4.81	4.11	3.52	2.85	2.34	1.46	0
1967	3.24	3.85	5.44	6.78	6.18	5.49	4.8	4.1	3.52	2.87	2.28	1.67	1.36
1968	3.24	3.87	5.44	6.78	6.18	5.49	4.8	4.11	3.48	3	2.38	1.3	0
1969	3.24	4.42	6.22	7.2	6.84	6.13	5.37	4.6	3.93	3.28	2.67	1.93	0.6
1970	3.24	4.5	6.3	7.2	6.84	6.13	5.36	4.57	3.85	3.12	2.35	1.8	0.48
1971	3.24	4.44	6.24	7.2	6.85	6.14	5.38	4.61	3.88	3.09	2.6	2.26	2.03
1972	3.24	4.41	6.21	7.2	6.85	6.14	5.37	4.59	3.88	3.16	2.5	1.28	0
1973	3.24	4.46	6.27	7.2	6.84	6.13	5.37	4.59	3.9	3.21	2.46	2.09	0.7
1974	3.24	4.48	6.28	7.2	6.84	6.13	5.37	4.59	3.93	3.26	2.6	1.96	1.48
1975	3.24	4.21	5.92	6.96	6.34	5.56	4.79	4.01	3.32	2.74	2.25	1.73	0
1976	3.24	4.21	5.9	6.96	6.33	5.55	4.79	4.02	3.38	2.75	2.02	0	0
1977	3.24	4.25	5.94	6.96	6.33	5.56	4.77	3.99	3.33	2.67	2.04	1.38	0
1978	3.24	4.26	5.97	6.96	6.33	5.55	4.77	3.95	3.24	2.48	1.41	0	0
1979	3.24	4.28	5.98	6.96	6.32	5.55	4.77	3.99	3.34	2.73	2.27	1.46	1.4
1980	3.24	4.22	5.93	6.96	6.34	5.56	4.78	4	3.25	2.43	1.92	1.49	0
1981	3.24	3.92	5.5	6.81	6.14	5.35	4.58	3.79	3.08	2.56	1.81	1.53	0
1982	3.24	3.92	5.52	6.8	6.14	5.35	4.59	3.77	3.11	2.38	1.86	0.48	0
1983	3.24	3.95	5.54	6.8	6.13	5.36	4.57	3.76	3.13	2.46	2.09	1.23	0
1984	3.24	3.9	5.49	6.81	6.14	5.36	4.59	3.78	3.16	2.34	1.57	1.26	0
1985	3.24	4.03	5.64	6.79	6.12	5.35	4.58	3.8	3.17	2.45	1.65	1.26	1.08
1986	3.24	3.91	5.5	6.81	6.14	5.36	4.58	3.8	3.15	2.45	1.93	1.54	0
1987	3.24	4.22	5.91	6.95	6.29	5.48	4.66	3.81	3.08	2.33	1.53	0	0
1988	3.24	4.23	5.92	6.95	6.29	5.48	4.65	3.84	3.15	2.49	1.71	0	0
1989	3.24	4.2	5.89	6.95	6.3	5.48	4.66	3.79	3.15	2.33	1.59	1.08	0

1990	3.24	4.25	5.95	6.95	6.29	5.47	4.64	3.81	2.97	2.38	1.66	0	0
1991	3.24	4.19	5.9	6.95	6.29	5.47	4.65	3.84	3.09	2.22	1.41	0	0
1992	3.24	4.21	5.9	6.95	6.29	5.47	4.64	3.8	3.09	2.32	1.3	0	0
1993	3.24	4.49	6.47	7.01	6.65	6.23	5.79	5.36	4.98	4.6	4.24	3.83	3.47
1994	3.24	4.5	6.48	7.01	6.65	6.22	5.79	5.36	4.99	4.61	4.23	3.81	3.41
1995	3.24	4.49	6.47	7.01	6.65	6.22	5.8	5.37	5	4.62	4.26	3.88	3.51
1996	3.24	4.49	6.47	7.01	6.65	6.22	5.79	5.36	4.98	4.61	4.22	3.85	3.41
1997	3.24	4.52	6.5	7.01	6.65	6.22	5.79	5.37	4.98	4.62	4.24	3.84	3.49
1998	3.24	4.5	6.48	7.01	6.65	6.22	5.79	5.37	4.99	4.61	4.23	3.84	3.41
1999	3.24	4.64	6.76	7.1	7.03	6.93	6.84	6.74	6.66	6.58	6.49	6.41	6.33
2000	3.24	4.62	6.75	7.1	7.03	6.93	6.84	6.74	6.66	6.58	6.49	6.41	6.33
2001	3.24	4.62	6.75	7.1	7.03	6.93	6.84	6.74	6.66	6.58	6.49	6.41	6.33
2002	3.24	4.64	6.76	7.1	7.03	6.93	6.84	6.74	6.66	6.58	6.5	6.41	6.33
2003	3.24	4.64	6.76	7.1	7.03	6.93	6.84	6.74	6.66	6.58	6.49	6.41	6.33
2004	3.24	4.66	6.78	7.1	7.03	6.93	6.84	6.74	6.66	6.58	6.49	6.41	6.33
2005	3.24	4.84	6.93	7.35	7.33	7.16	6.98	6.8	6.64	6.49	6.33	6.18	6.02
2006	3.24	4.83	6.93	7.35	7.33	7.16	6.98	6.8	6.65	6.49	6.34	6.18	6.03
2007	3.24	4.88	6.94	7.35	7.33	7.16	6.98	6.8	6.65	6.49	6.33	6.18	6.02
2008	3.24	4.87	6.94	7.35	7.33	7.16	6.98	6.8	6.65	6.5	6.34	6.18	6.03
2009	3.24	4.8	6.92	7.35	7.33	7.16	6.98	6.8	6.65	6.49	6.33	6.18	6.02
2010	3.24	4.83	6.93	7.35	7.33	7.16	6.98	6.8	6.65	6.49	6.34	6.18	6.03
2011	3.24	4.62	6.74	7.07	6.93	6.75	6.57	6.39	6.24	6.08	5.92	5.77	5.62
2012	3.24	4.66	6.77	7.07	6.93	6.75	6.57	6.39	6.24	6.08	5.93	5.78	5.62
2013	3.24	4.68	6.79	7.07	6.93	6.75	6.57	6.39	6.24	6.08	5.92	5.77	5.62
2014	3.24	4.65	6.76	7.07	6.93	6.75	6.57	6.4	6.24	6.08	5.93	5.77	5.62
2015	3.24	4.64	6.77	7.07	6.93	6.75	6.57	6.39	6.24	6.08	5.93	5.77	5.61
2016	3.24	4.61	6.74	7.07	6.93	6.75	6.57	6.4	6.24	6.09	5.93	5.77	5.62
2017	3.24	4.33	6.41	6.88	6.73	6.55	6.37	6.19	6.04	5.88	5.72	5.57	5.41
2018	3.24	4.35	6.42	6.88	6.73	6.55	6.37	6.19	6.04	5.88	5.73	5.57	5.41
2019	3.24	4.36	6.44	6.88	6.73	6.55	6.37	6.19	6.04	5.88	5.73	5.57	5.41
2020	3.24	4.38	6.46	6.88	6.73	6.55	6.37	6.19	6.03	5.88	5.72	5.57	5.41
2021	3.24	4.32	6.41	6.88	6.73	6.55	6.37	6.19	6.04	5.88	5.73	5.57	5.41
2022	3.24	4.29	6.38	6.88	6.73	6.55	6.37	6.19	6.04	5.89	5.73	5.57	5.42
2023	3.24	4.92	6.97	7.41	7.38	7.09	6.71	6.31	5.96	5.59	5.22	4.84	4.47
2024	3.24	4.91	6.96	7.41	7.38	7.09	6.72	6.31	5.95	5.59	5.23	4.86	4.51
2025	3.24	4.9	6.96	7.41	7.38	7.09	6.71	6.31	5.95	5.59	5.22	4.84	4.47
2026	3.24	4.9	6.96	7.41	7.38	7.09	6.71	6.31	5.95	5.58	5.21	4.83	4.44
2027	3.24	4.87	6.95	7.41	7.38	7.09	6.72	6.32	5.96	5.59	5.22	4.85	4.48
2028	3.24	4.89	6.96	7.41	7.38	7.09	6.72	6.32	5.96	5.59	5.21	4.84	4.47
2029	3.24	4.85	6.92	7.29	7.08	6.68	6.25	5.82	5.44	5.07	4.7	4.32	3.95
2030	3.24	4.86	6.92	7.29	7.08	6.68	6.25	5.82	5.44	5.07	4.69	4.3	3.9
2031	3.24	4.85	6.92	7.29	7.08	6.68	6.25	5.83	5.45	5.08	4.72	4.36	3.98
2032	3.24	4.84	6.92	7.29	7.08	6.68	6.25	5.82	5.44	5.07	4.7	4.33	3.95
2033	3.24	4.82	6.92	7.29	7.09	6.68	6.25	5.82	5.45	5.06	4.69	4.35	3.98
2034	3.24	4.83	6.92	7.29	7.08	6.68	6.25	5.82	5.45	5.07	4.68	4.33	3.96
2035	3.24	4.65	6.76	7	6.64	6.21	5.78	5.35	4.98	4.59	4.2	3.82	3.43
2036	3.24	4.57	6.69	7	6.64	6.21	5.79	5.36	4.99	4.62	4.24	3.87	3.49
2037	3.24	4.64	6.74	7	6.64	6.21	5.78	5.35	4.97	4.61	4.23	3.87	3.47
2038	3.24	4.65	6.75	7	6.64	6.21	5.78	5.36	4.98	4.62	4.25	3.91	3.51
2039	3.24	4.63	6.74	7	6.64	6.21	5.79	5.36	4.98	4.6	4.22	3.88	3.5
2040	3.24	4.64	6.74	7	6.64	6.21	5.78	5.35	4.98	4.6	4.21	3.84	3.47
2041	3.24	4.33	6.4	6.8	6.43	6	5.57	5.14	4.76	4.41	4.03	3.64	3.27
2042	3.24	4.29	6.36	6.81	6.43	6.01	5.58	5.15	4.78	4.39	4	3.62	3.21
2043	3.24	4.34	6.41	6.8	6.43	6	5.57	5.15	4.77	4.39	4.01	3.61	3.22

2044	3.24	4.3	6.38	6.81	6.43	6	5.58	5.15	4.77	4.39	4.03	3.64	3.23
2045	3.24	4.42	6.48	6.8	6.43	6	5.57	5.14	4.77	4.4	4.02	3.62	3.22
2046	3.24	4.29	6.37	6.81	6.43	6.01	5.58	5.15	4.76	4.37	3.98	3.64	3.25
2047	3.24	4.25	6.31	6.76	6.38	5.95	5.52	5.1	4.73	4.37	3.99	3.59	3.23
2048	3.24	4.2	6.26	6.76	6.38	5.96	5.53	5.1	4.72	4.35	3.97	3.59	3.17
2049	3.24	4.17	6.22	6.76	6.39	5.96	5.52	5.1	4.73	4.36	4.01	3.62	3.28
2050	3.24	4.2	6.25	6.76	6.38	5.95	5.53	5.1	4.72	4.36	3.97	3.59	3.23
2051	3.24	4.19	6.25	6.76	6.38	5.96	5.53	5.1	4.73	4.35	3.97	3.61	3.23
2052	3.24	4.27	6.32	6.75	6.38	5.95	5.52	5.09	4.71	4.32	3.94	3.55	3.08
2053	3.24	4.85	6.91	7.23	6.85	6.21	5.53	4.84	4.24	3.64	3.03	2.48	2.09
2054	3.24	4.86	6.92	7.23	6.85	6.21	5.53	4.85	4.25	3.65	3.02	2.31	1.69
2055	3.24	4.82	6.91	7.23	6.85	6.21	5.53	4.84	4.21	3.64	3.08	2.38	1.4
2056	3.24	4.82	6.91	7.23	6.85	6.21	5.54	4.86	4.24	3.57	3	2.28	1.72
2057	3.24	4.89	6.93	7.23	6.85	6.2	5.52	4.82	4.2	3.65	3.02	2.46	1.26
2058	3.24	4.85	6.91	7.23	6.85	6.21	5.53	4.86	4.23	3.6	2.97	2.3	1.95
2059	3.24	4.61	6.7	6.92	6.34	5.66	4.97	4.29	3.69	3.11	2.55	2.01	0
2060	3.24	4.56	6.66	6.93	6.34	5.66	4.96	4.26	3.66	3.03	2.22	1.72	0.9
2061	3.24	4.63	6.72	6.92	6.34	5.65	4.96	4.28	3.71	3.14	2.57	2.18	1.98
2062	3.24	4.64	6.73	6.92	6.34	5.65	4.96	4.26	3.67	2.97	2.1	1.75	0.85
2063	3.24	4.69	6.77	6.92	6.33	5.64	4.96	4.29	3.69	3.12	2.35	1.71	0
2064	3.24	4.62	6.72	6.92	6.34	5.65	4.96	4.24	3.64	3.07	2.52	1.83	1.53
2065	3.24	4.36	6.4	6.72	6.12	5.43	4.74	4.02	3.45	2.85	2.14	1.64	0.85
2066	3.24	4.34	6.39	6.72	6.12	5.44	4.74	4.05	3.46	2.91	2.25	1.26	1.11
2067	3.24	4.34	6.39	6.73	6.13	5.44	4.74	4.04	3.39	2.8	2.19	1.56	0
2068	3.24	4.3	6.35	6.73	6.13	5.44	4.75	4.07	3.47	2.85	2.19	1.26	0
2069	3.24	4.38	6.43	6.72	6.12	5.43	4.75	4.05	3.44	2.92	2.34	1.53	0.6
2070	3.24	4.31	6.37	6.73	6.13	5.43	4.75	4.08	3.49	2.85	2.29	1.57	1.23
2071	3.24	4.69	6.76	6.89	6.23	5.44	4.66	3.89	3.19	2.57	1.77	1.67	0
2072	3.24	4.59	6.69	6.9	6.24	5.46	4.67	3.91	3.26	2.52	2.05	1.58	0
2073	3.24	4.66	6.74	6.89	6.23	5.45	4.66	3.89	3.23	2.46	1.86	1.36	0
2074	3.24	4.62	6.71	6.9	6.23	5.46	4.67	3.89	3.19	2.46	1.75	1.67	0.6
2075	3.24	4.64	6.73	6.89	6.23	5.45	4.66	3.87	3.17	2.4	1.9	1.38	0
2076	3.24	4.55	6.65	6.9	6.24	5.45	4.68	3.92	3.21	2.51	2.1	0.3	0
2077	3.24	4.74	6.87	7.07	6.92	6.75	6.57	6.39	6.23	6.08	5.92	5.77	5.61
2078	3.24	4.71	6.86	7.07	6.92	6.75	6.57	6.39	6.23	6.08	5.92	5.76	5.61
2079	3.24	4.72	6.87	7.07	6.92	6.75	6.57	6.39	6.24	6.08	5.92	5.77	5.61
2080	3.24	4.77	6.88	7.07	6.92	6.75	6.57	6.39	6.23	6.08	5.92	5.76	5.61
2081	3.24	4.74	6.87	7.07	6.92	6.75	6.57	6.39	6.24	6.08	5.93	5.77	5.61
2082	3.24	4.73	6.87	7.07	6.92	6.75	6.57	6.39	6.24	6.08	5.92	5.76	5.61
2083	3.24	4.94	6.98	7.29	7.08	6.67	6.24	5.81	5.44	5.06	4.7	4.33	3.93
2084	3.24	4.94	6.98	7.29	7.08	6.67	6.24	5.81	5.44	5.07	4.69	4.34	3.94
2085	3.24	4.97	6.99	7.29	7.08	6.67	6.24	5.81	5.44	5.07	4.69	4.31	3.9
2086	3.24	4.93	6.98	7.29	7.08	6.67	6.25	5.81	5.43	5.04	4.66	4.29	3.92
2087	3.24	4.92	6.97	7.29	7.08	6.67	6.25	5.82	5.45	5.07	4.69	4.31	3.98
2088	3.24	4.93	6.98	7.29	7.08	6.67	6.24	5.81	5.43	5.05	4.66	4.28	3.92
2089	3.24	4.73	6.86	6.99	6.63	6.2	5.77	5.35	4.96	4.59	4.22	3.87	3.45
2090	3.24	4.68	6.85	6.99	6.63	6.2	5.77	5.35	4.97	4.59	4.24	3.87	3.49
2091	3.24	4.7	6.85	6.99	6.63	6.2	5.77	5.34	4.96	4.57	4.21	3.82	3.44
2092	3.24	4.74	6.86	6.99	6.63	6.2	5.77	5.34	4.98	4.6	4.23	3.87	3.46
2093	3.24	4.68	6.85	6.99	6.63	6.2	5.77	5.34	4.97	4.58	4.23	3.86	3.51
2094	3.24	4.75	6.86	6.99	6.62	6.2	5.77	5.35	4.96	4.59	4.2	3.81	3.41
2095	3.24	4.47	6.63	6.79	6.42	5.99	5.56	5.13	4.76	4.38	3.99	3.63	3.28
2096	3.24	4.48	6.64	6.79	6.42	5.99	5.56	5.14	4.77	4.39	4.01	3.61	3.19
2097	3.24	4.55	6.7	6.79	6.41	5.98	5.56	5.13	4.76	4.39	4	3.6	3.29

2098	3.24	4.45	6.62	6.79	6.42	5.99	5.56	5.13	4.75	4.38	4.01	3.62	3.28
2099	3.24	4.5	6.66	6.79	6.42	5.99	5.56	5.13	4.76	4.4	4.03	3.66	3.26
2100	3.24	4.53	6.69	6.79	6.42	5.99	5.56	5.13	4.76	4.38	4	3.65	3.26
2101	3.24	4.75	6.85	6.91	6.32	5.63	4.94	4.25	3.64	3.12	2.46	2.01	0.85
2102	3.24	4.75	6.85	6.91	6.32	5.63	4.95	4.28	3.67	3.05	2.4	1.85	0
2103	3.24	4.7	6.84	6.91	6.32	5.63	4.94	4.28	3.68	3.11	2.5	1.69	0
2104	3.24	4.74	6.85	6.91	6.32	5.63	4.95	4.27	3.67	3.03	2.38	1.43	0
2105	3.24	4.71	6.85	6.91	6.32	5.63	4.94	4.23	3.64	2.99	2.37	1.86	0.48
2106	3.24	4.74	6.85	6.91	6.32	5.62	4.94	4.26	3.65	3.11	2.64	2.13	1.87
2107	3.24	4.79	6.88	6.99	6.62	6.19	5.77	5.34	4.96	4.58	4.2	3.86	3.43
2108	3.24	4.79	6.88	6.99	6.62	6.19	5.77	5.33	4.96	4.55	4.16	3.8	3.34
2109	3.24	4.78	6.88	6.99	6.62	6.19	5.77	5.34	4.97	4.59	4.22	3.83	3.44
2110	3.24	4.74	6.87	6.99	6.62	6.19	5.76	5.34	4.96	4.58	4.21	3.82	3.45
2111	3.24	4.73	6.87	6.99	6.62	6.2	5.77	5.34	4.96	4.59	4.23	3.86	3.54
2112	3.24	4.78	6.88	6.99	6.62	6.19	5.76	5.33	4.96	4.57	4.18	3.8	3.38
2113	3.24	3.29	4.02	5.02	6.9	6.68	6.25	5.82	5.45	5.08	4.71	4.34	3.99
2114	3.24	3.36	4.09	5.08	6.91	6.67	6.24	5.81	5.44	5.05	4.67	4.28	3.9
2115	3.24	3.42	4.11	5.09	6.92	6.67	6.24	5.81	5.44	5.07	4.69	4.31	3.92
2116	3.24	3.45	4.13	5.12	6.92	6.66	6.23	5.8	5.43	5.05	4.68	4.3	3.91
2117	3.24	3.36	4.09	5.07	6.91	6.67	6.24	5.81	5.44	5.06	4.68	4.31	3.96
2118	3.24	3.46	4.14	5.13	6.92	6.66	6.23	5.8	5.43	5.06	4.67	4.29	3.9
2119	3.24	3.67	4.74	6.28	7.02	6.84	6.66	6.48	6.33	6.17	6.01	5.86	5.7
2120	3.24	3.69	4.76	6.3	7.02	6.84	6.66	6.48	6.33	6.17	6.02	5.86	5.7
2121	3.24	3.7	4.76	6.3	7.02	6.84	6.66	6.48	6.33	6.17	6.02	5.86	5.7
2122	3.24	3.65	4.74	6.28	7.02	6.84	6.66	6.48	6.33	6.17	6.02	5.86	5.71
2123	3.24	3.73	4.81	6.34	7.01	6.84	6.66	6.48	6.32	6.17	6.01	5.86	5.7
2124	3.24	3.71	4.79	6.32	7.02	6.84	6.66	6.48	6.33	6.17	6.01	5.86	5.7
2125	3.24	3.98	5.17	6.8	7.22	6.85	6.42	5.99	5.62	5.25	4.87	4.48	4.13
2126	3.24	3.96	5.14	6.78	7.22	6.85	6.43	6	5.63	5.26	4.88	4.52	4.12
2127	3.24	3.88	5.05	6.7	7.23	6.86	6.43	6	5.64	5.26	4.87	4.49	4.12
2128	3.24	3.85	5.02	6.68	7.23	6.86	6.44	6.01	5.64	5.28	4.9	4.51	4.11
2129	3.24	3.95	5.14	6.78	7.22	6.85	6.43	6	5.62	5.26	4.88	4.51	4.13
2130	3.24	3.92	5.09	6.74	7.22	6.85	6.43	6	5.62	5.25	4.86	4.48	4.07
2131	3.24	3.68	4.71	6.21	6.86	6.43	6	5.57	5.19	4.82	4.44	4.08	3.67
2132	3.24	3.68	4.72	6.24	6.86	6.43	6	5.58	5.2	4.83	4.47	4.08	3.71
2133	3.24	3.63	4.68	6.19	6.86	6.43	6	5.57	5.2	4.82	4.46	4.08	3.73
2134	3.24	3.69	4.72	6.23	6.86	6.43	6	5.57	5.2	4.83	4.46	4.09	3.66
2135	3.24	3.7	4.75	6.25	6.86	6.43	6	5.57	5.2	4.83	4.44	4.06	3.68
2136	3.24	3.62	4.63	6.15	6.86	6.44	6.01	5.57	5.19	4.82	4.45	4.08	3.7
2137	3.24	3.49	4.42	5.76	6.7	6.27	5.84	5.41	5.03	4.65	4.29	3.9	3.52
2138	3.24	3.39	4.31	5.67	6.71	6.28	5.85	5.42	5.05	4.68	4.3	3.93	3.56
2139	3.24	3.43	4.34	5.69	6.71	6.28	5.85	5.41	5.04	4.65	4.27	3.89	3.54
2140	3.24	3.33	4.22	5.56	6.72	6.29	5.86	5.43	5.05	4.68	4.29	3.94	3.57
2141	3.24	3.4	4.3	5.64	6.71	6.28	5.85	5.43	5.06	4.68	4.32	3.95	3.56
2142	3.24	3.38	4.35	5.68	6.71	6.28	5.85	5.42	5.04	4.66	4.29	3.92	3.55
2143	3.24	3.65	4.66	6.13	6.71	6.03	5.34	4.65	4.07	3.38	2.81	2.11	1.65
2144	3.24	3.68	4.73	6.19	6.7	6.01	5.32	4.63	4.04	3.37	2.76	2.24	1.76
2145	3.24	3.65	4.66	6.14	6.71	6.03	5.34	4.66	4.05	3.38	2.85	2.17	0.95
2146	3.24	3.69	4.71	6.18	6.7	6.02	5.32	4.63	4.04	3.35	2.9	2.39	1.76
2147	3.24	3.65	4.67	6.14	6.71	6.03	5.34	4.65	4.05	3.43	2.8	2.32	1.77
2148	3.24	3.7	4.72	6.18	6.7	6.02	5.33	4.66	4.06	3.45	2.81	2.26	1.58
2149	3.24	4.25	5.99	7.09	7.04	6.94	6.85	6.75	6.67	6.59	6.51	6.43	6.34
2150	3.24	4.22	5.98	7.09	7.04	6.94	6.85	6.75	6.67	6.59	6.51	6.42	6.34
2151	3.24	4.24	5.99	7.09	7.04	6.94	6.85	6.75	6.67	6.59	6.51	6.42	6.34

2152	3.24	4.24	5.99	7.09	7.04	6.94	6.85	6.75	6.67	6.59	6.51	6.42	6.34
2153	3.24	4.28	6.02	7.09	7.04	6.94	6.85	6.75	6.67	6.59	6.51	6.42	6.34
2154	3.24	4.21	5.97	7.09	7.04	6.94	6.85	6.76	6.67	6.59	6.51	6.42	6.34
2155	3.24	4.45	6.28	7.31	7.34	7.18	7	6.82	6.66	6.51	6.35	6.19	6.04
2156	3.24	4.46	6.29	7.31	7.34	7.18	7	6.82	6.67	6.51	6.36	6.2	6.04
2157	3.24	4.49	6.32	7.31	7.34	7.17	7	6.82	6.66	6.51	6.35	6.2	6.05
2158	3.24	4.48	6.32	7.31	7.34	7.18	7	6.82	6.66	6.51	6.36	6.2	6.04
2159	3.24	4.48	6.3	7.31	7.34	7.18	7	6.82	6.66	6.51	6.35	6.2	6.04
2160	3.24	4.46	6.3	7.31	7.34	7.18	7	6.82	6.67	6.51	6.36	6.2	6.04
2161	3.24	4.33	6.07	7.07	6.95	6.77	6.59	6.41	6.26	6.1	5.95	5.79	5.64
2162	3.24	4.26	5.99	7.07	6.95	6.77	6.59	6.42	6.26	6.1	5.95	5.79	5.64
2163	3.24	4.21	5.95	7.07	6.95	6.77	6.59	6.42	6.26	6.1	5.95	5.79	5.64
2164	3.24	4.23	5.98	7.07	6.95	6.77	6.59	6.41	6.26	6.1	5.95	5.79	5.63
2165	3.24	4.2	5.96	7.07	6.95	6.77	6.59	6.41	6.26	6.1	5.95	5.79	5.64
2166	3.24	4.21	5.95	7.07	6.95	6.77	6.59	6.42	6.26	6.11	5.95	5.8	5.64
2167	3.24	3.91	5.56	6.9	6.75	6.57	6.4	6.22	6.06	5.9	5.75	5.59	5.44
2168	3.24	3.92	5.56	6.9	6.75	6.58	6.4	6.22	6.06	5.91	5.75	5.59	5.44
2169	3.24	4	5.64	6.9	6.75	6.57	6.4	6.22	6.06	5.91	5.75	5.59	5.44
2170	3.24	3.95	5.6	6.9	6.75	6.58	6.4	6.22	6.06	5.91	5.75	5.59	5.44
2171	3.24	3.99	5.64	6.9	6.75	6.57	6.39	6.22	6.06	5.9	5.75	5.59	5.43
2172	3.24	3.96	5.61	6.9	6.75	6.57	6.4	6.22	6.06	5.91	5.75	5.6	5.44
2173	3.24	4.55	6.4	7.37	7.4	7.12	6.75	6.35	5.99	5.63	5.27	4.9	4.53
2174	3.24	4.52	6.37	7.37	7.4	7.12	6.75	6.35	5.99	5.62	5.25	4.88	4.51
2175	3.24	4.52	6.37	7.37	7.41	7.12	6.75	6.36	6	5.63	5.27	4.9	4.53
2176	3.24	4.56	6.4	7.37	7.4	7.12	6.75	6.35	5.99	5.62	5.25	4.87	4.5
2177	3.24	4.57	6.41	7.37	7.4	7.12	6.75	6.36	6	5.63	5.26	4.87	4.47
2178	3.24	4.54	6.4	7.37	7.4	7.12	6.75	6.35	5.99	5.63	5.25	4.88	4.5
2179	3.24	4.52	6.34	7.27	7.12	6.72	6.3	5.87	5.49	5.12	4.75	4.4	4.03
2180	3.24	4.49	6.31	7.27	7.12	6.72	6.29	5.87	5.49	5.1	4.73	4.35	3.97
2181	3.24	4.47	6.29	7.27	7.12	6.72	6.3	5.87	5.49	5.1	4.73	4.36	3.99
2182	3.24	4.52	6.33	7.27	7.12	6.72	6.29	5.87	5.49	5.12	4.74	4.37	3.99
2183	3.24	4.49	6.31	7.27	7.12	6.72	6.3	5.87	5.48	5.11	4.72	4.33	3.96
2184	3.24	4.49	6.31	7.27	7.12	6.72	6.3	5.87	5.49	5.12	4.74	4.37	4
2185	3.24	4.27	5.99	7.02	6.69	6.26	5.83	5.41	5.03	4.65	4.28	3.86	3.46
2186	3.24	4.26	5.98	7.02	6.69	6.26	5.83	5.41	5.03	4.67	4.29	3.92	3.51
2187	3.24	4.21	5.94	7.02	6.69	6.26	5.84	5.4	5.03	4.65	4.29	3.93	3.52
2188	3.24	4.27	6	7.02	6.69	6.26	5.83	5.41	5.03	4.65	4.25	3.86	3.47
2189	3.24	4.22	5.95	7.02	6.69	6.26	5.83	5.4	5.02	4.65	4.28	3.92	3.54
2190	3.24	4.3	6.03	7.02	6.68	6.26	5.83	5.39	5.02	4.66	4.28	3.91	3.51
2191	3.24	3.97	5.61	6.86	6.49	6.06	5.63	5.21	4.83	4.45	4.09	3.72	3.33
2192	3.24	4.04	5.67	6.85	6.49	6.06	5.63	5.2	4.82	4.44	4.06	3.65	3.3
2193	3.24	3.93	5.55	6.86	6.49	6.07	5.64	5.21	4.84	4.46	4.09	3.72	3.35
2194	3.24	3.98	5.61	6.86	6.49	6.06	5.63	5.21	4.83	4.47	4.09	3.71	3.33
2195	3.24	3.96	5.57	6.86	6.49	6.06	5.64	5.2	4.82	4.45	4.07	3.65	3.31
2196	3.24	3.96	5.59	6.86	6.49	6.06	5.63	5.21	4.83	4.45	4.07	3.74	3.38
2197	3.24	3.81	5.4	6.82	6.45	6.02	5.59	5.17	4.8	4.43	4.05	3.7	3.28
2198	3.24	3.89	5.47	6.81	6.44	6.02	5.58	5.16	4.78	4.41	4.06	3.71	3.35
2199	3.24	3.81	5.4	6.82	6.45	6.02	5.59	5.16	4.78	4.41	4.04	3.69	3.34
2200	3.24	3.92	5.51	6.81	6.44	6.01	5.58	5.16	4.78	4.41	4.04	3.67	3.29
2201	3.24	3.8	5.39	6.82	6.45	6.02	5.59	5.17	4.79	4.42	4.06	3.67	3.28
2202	3.24	3.91	5.5	6.81	6.44	6.01	5.59	5.16	4.78	4.4	4.04	3.66	3.29
2203	3.24	4.49	6.29	7.22	6.91	6.28	5.6	4.92	4.33	3.77	3.12	2.3	0.95
2204	3.24	4.49	6.3	7.22	6.91	6.28	5.59	4.9	4.28	3.63	3.02	2.47	2.17
2205	3.24	4.5	6.3	7.22	6.91	6.27	5.6	4.91	4.31	3.7	3.14	2.48	1.46

2206	3.24	4.46	6.26	7.22	6.91	6.28	5.6	4.92	4.31	3.72	3.26	2.61	2.3
2207	3.24	4.48	6.28	7.22	6.91	6.28	5.59	4.9	4.3	3.69	3.13	2.41	1.94
2208	3.24	4.48	6.29	7.22	6.91	6.28	5.6	4.9	4.25	3.61	2.93	2.53	2.18
2209	3.24	4.28	5.98	6.97	6.42	5.74	5.05	4.37	3.77	3.09	2.42	1.84	0
2210	3.24	4.23	5.93	6.97	6.42	5.73	5.04	4.34	3.71	3.12	2.51	1.96	1.71
2211	3.24	4.28	5.98	6.97	6.42	5.73	5.05	4.38	3.78	3.21	2.52	1.76	0
2212	3.24	4.26	5.96	6.97	6.42	5.74	5.05	4.37	3.77	3.16	2.5	1.88	0.48
2213	3.24	4.18	5.88	6.98	6.43	5.74	5.05	4.36	3.74	3.14	2.31	1.83	0
2214	3.24	4.18	5.88	6.98	6.43	5.74	5.06	4.35	3.73	3.03	2.45	1.98	0
2215	3.24	3.98	5.57	6.81	6.22	5.53	4.85	4.16	3.61	3.04	2.46	1.26	1.15
2216	3.24	3.98	5.57	6.81	6.22	5.53	4.85	4.17	3.51	2.84	2.05	1.62	1.11
2217	3.24	3.97	5.59	6.81	6.22	5.53	4.84	4.16	3.58	2.98	2.22	1.63	1.15
2218	3.24	3.89	5.49	6.82	6.23	5.55	4.86	4.16	3.52	2.89	2.15	1.66	0.78
2219	3.24	3.92	5.52	6.82	6.23	5.54	4.86	4.19	3.56	3.02	2.39	1.98	0
2220	3.24	3.91	5.49	6.82	6.23	5.54	4.86	4.18	3.63	3.02	2.36	1.85	1.23
2221	3.24	4.25	5.94	6.96	6.33	5.55	4.78	4	3.22	2.61	2.11	1.62	1.7
2222	3.24	4.25	5.95	6.96	6.33	5.55	4.76	3.98	3.29	2.59	2.08	1.23	0
2223	3.24	4.27	5.97	6.96	6.33	5.55	4.76	4	3.33	2.63	1.81	1.3	0
2224	3.24	4.22	5.91	6.96	6.34	5.56	4.78	3.99	3.33	2.54	1.79	0.3	0
2225	3.24	4.25	5.95	6.96	6.33	5.55	4.77	3.99	3.35	2.78	2.24	1.49	1.4
2226	3.24	4.26	5.96	6.96	6.33	5.55	4.77	4	3.3	2.55	1.57	0	0
2227	3.24	4.62	6.74	7.07	6.93	6.75	6.57	6.39	6.24	6.08	5.93	5.77	5.62
2228	3.24	4.56	6.69	7.07	6.93	6.75	6.57	6.4	6.24	6.08	5.93	5.77	5.62
2229	3.24	4.66	6.77	7.07	6.93	6.75	6.57	6.39	6.24	6.09	5.93	5.78	5.62
2230	3.24	4.65	6.76	7.07	6.93	6.75	6.57	6.4	6.24	6.08	5.93	5.77	5.61
2231	3.24	4.61	6.73	7.07	6.93	6.75	6.57	6.4	6.24	6.08	5.93	5.77	5.61
2232	3.24	4.64	6.75	7.07	6.93	6.75	6.57	6.39	6.24	6.08	5.92	5.77	5.61
2233	3.24	4.87	6.93	7.29	7.09	6.68	6.25	5.82	5.45	5.08	4.71	4.34	3.94
2234	3.24	4.82	6.91	7.29	7.09	6.68	6.25	5.83	5.45	5.08	4.71	4.34	3.96
2235	3.24	4.82	6.91	7.29	7.09	6.68	6.25	5.82	5.45	5.08	4.7	4.35	3.97
2236	3.24	4.83	6.92	7.29	7.09	6.68	6.25	5.82	5.45	5.07	4.68	4.29	3.92
2237	3.24	4.82	6.91	7.29	7.09	6.68	6.25	5.81	5.44	5.06	4.68	4.27	3.87
2238	3.24	4.8	6.91	7.29	7.09	6.68	6.25	5.82	5.44	5.07	4.68	4.29	3.91
2239	3.24	4.63	6.74	7	6.64	6.21	5.78	5.35	4.98	4.61	4.25	3.93	3.56
2240	3.24	4.63	6.74	7	6.64	6.21	5.78	5.35	4.97	4.6	4.23	3.85	3.44
2241	3.24	4.67	6.76	7	6.63	6.21	5.78	5.35	4.98	4.62	4.24	3.84	3.44
2242	3.24	4.64	6.74	7	6.64	6.21	5.78	5.35	4.98	4.62	4.27	3.86	3.47
2243	3.24	4.6	6.71	7	6.64	6.21	5.78	5.36	4.98	4.6	4.24	3.88	3.49
2244	3.24	4.6	6.71	7	6.64	6.21	5.78	5.35	4.99	4.61	4.22	3.85	3.45
2245	3.24	4.37	6.44	6.8	6.43	6	5.57	5.14	4.76	4.39	4.01	3.63	3.32
2246	3.24	4.29	6.36	6.81	6.43	6.01	5.57	5.14	4.77	4.39	4.02	3.65	3.25
2247	3.24	4.36	6.43	6.8	6.43	6	5.57	5.14	4.77	4.4	4.02	3.65	3.24
2248	3.24	4.37	6.44	6.8	6.43	6	5.57	5.14	4.77	4.39	4.03	3.64	3.25
2249	3.24	4.38	6.45	6.8	6.43	6	5.57	5.14	4.76	4.38	4.03	3.65	3.29
2250	3.24	4.39	6.45	6.8	6.43	6	5.57	5.14	4.76	4.39	4.04	3.66	3.29
2251	3.24	4.61	6.7	6.92	6.34	5.66	4.97	4.27	3.72	3.18	2.58	1.77	1.04
2252	3.24	4.6	6.69	6.92	6.34	5.65	4.96	4.28	3.69	3.09	2.64	2.04	1.51
2253	3.24	4.65	6.74	6.92	6.34	5.64	4.96	4.26	3.65	3.05	2.44	1.86	1.36
2254	3.24	4.59	6.69	6.92	6.34	5.65	4.96	4.28	3.69	3.04	2.29	1.85	0
2255	3.24	4.61	6.7	6.92	6.34	5.65	4.96	4.28	3.65	2.97	2.29	1.46	0
2256	3.24	4.62	6.71	6.92	6.34	5.65	4.96	4.26	3.65	3.03	2.49	1.38	0
2257	3.24	4.79	6.87	6.99	6.62	6.19	5.77	5.34	4.97	4.57	4.2	3.83	3.44
2258	3.24	4.74	6.86	6.99	6.63	6.2	5.77	5.35	4.98	4.58	4.21	3.82	3.44
2259	3.24	4.73	6.86	6.99	6.63	6.2	5.77	5.34	4.96	4.58	4.2	3.83	3.44

2260	3.24	4.72	6.86	6.99	6.63	6.2	5.77	5.34	4.96	4.57	4.18	3.81	3.41
2261	3.24	4.8	6.87	6.99	6.62	6.19	5.76	5.33	4.96	4.59	4.2	3.81	3.46
2262	3.24	4.75	6.86	6.99	6.63	6.2	5.77	5.34	4.96	4.58	4.2	3.84	3.5
2263	3.24	3.72	4.78	6.28	6.85	6.42	5.99	5.56	5.19	4.81	4.43	4.08	3.75
2264	3.24	3.69	4.73	6.24	6.86	6.43	6	5.57	5.19	4.81	4.42	4.05	3.68
2265	3.24	3.69	4.75	6.26	6.85	6.43	6	5.57	5.19	4.82	4.46	4.08	3.71
2266	3.24	3.74	4.8	6.31	6.85	6.42	5.99	5.56	5.18	4.8	4.42	4.08	3.7
2267	3.24	3.62	4.67	6.18	6.86	6.44	6	5.57	5.2	4.83	4.44	4.1	3.74
2268	3.24	3.67	4.7	6.2	6.86	6.43	6	5.57	5.19	4.82	4.43	4.06	3.65
2269	3.24	4.23	5.98	7.07	6.95	6.77	6.59	6.42	6.26	6.1	5.95	5.79	5.64
2270	3.24	4.26	6	7.07	6.95	6.77	6.59	6.42	6.26	6.1	5.95	5.79	5.63
2271	3.24	4.26	6	7.07	6.95	6.77	6.59	6.42	6.26	6.1	5.95	5.79	5.63
2272	3.24	4.26	6	7.07	6.95	6.77	6.59	6.41	6.26	6.1	5.95	5.79	5.63
2273	3.24	4.28	6.02	7.07	6.95	6.77	6.59	6.42	6.26	6.1	5.95	5.79	5.63
2274	3.24	4.25	6	7.07	6.95	6.77	6.59	6.41	6.26	6.1	5.95	5.79	5.64
2275	3.24	4.46	6.29	7.27	7.12	6.72	6.3	5.87	5.49	5.11	4.74	4.36	3.97
2276	3.24	4.48	6.3	7.27	7.12	6.72	6.3	5.87	5.5	5.13	4.75	4.37	3.99
2277	3.24	4.48	6.3	7.27	7.12	6.72	6.3	5.87	5.5	5.13	4.76	4.39	4.01
2278	3.24	4.47	6.29	7.27	7.12	6.72	6.3	5.87	5.49	5.11	4.72	4.34	3.94
2279	3.24	4.48	6.3	7.27	7.12	6.72	6.3	5.87	5.5	5.13	4.75	4.35	3.97
2280	3.24	4.49	6.31	7.27	7.12	6.72	6.3	5.87	5.5	5.12	4.75	4.38	4.02
2281	3.24	4.28	5.99	7.02	6.69	6.26	5.83	5.41	5.03	4.67	4.31	3.92	3.56
2282	3.24	4.25	5.96	7.02	6.69	6.26	5.83	5.41	5.04	4.66	4.27	3.91	3.55
2283	3.24	4.21	5.93	7.02	6.69	6.26	5.83	5.41	5.03	4.66	4.28	3.9	3.54
2284	3.24	4.25	5.97	7.02	6.69	6.26	5.83	5.41	5.03	4.64	4.27	3.91	3.56
2285	3.24	4.23	5.94	7.02	6.69	6.26	5.83	5.41	5.03	4.65	4.27	3.88	3.48
2286	3.24	4.2	5.92	7.02	6.69	6.26	5.83	5.41	5.03	4.67	4.29	3.9	3.56
2287	3.24	3.91	5.53	6.86	6.5	6.07	5.64	5.21	4.84	4.46	4.09	3.67	3.33
2288	3.24	4.02	5.64	6.86	6.49	6.06	5.63	5.2	4.83	4.46	4.07	3.71	3.33
2289	3.24	3.96	5.59	6.86	6.49	6.06	5.64	5.21	4.84	4.46	4.06	3.69	3.26
2290	3.24	3.95	5.58	6.86	6.49	6.06	5.63	5.2	4.82	4.46	4.1	3.71	3.27
2291	3.24	3.98	5.59	6.86	6.49	6.06	5.63	5.21	4.84	4.47	4.1	3.71	3.32
2292	3.24	3.95	5.58	6.86	6.49	6.06	5.63	5.2	4.82	4.46	4.08	3.67	3.27
2293	3.24	4.23	5.93	6.98	6.42	5.74	5.04	4.34	3.7	3.11	2.5	1.92	1.85
2294	3.24	4.21	5.91	6.98	6.43	5.74	5.05	4.37	3.76	3.23	2.57	1.9	1.38
2295	3.24	4.23	5.92	6.98	6.43	5.74	5.06	4.38	3.79	3.23	2.67	1.86	1.2
2296	3.24	4.28	5.98	6.97	6.42	5.73	5.05	4.34	3.77	3.21	2.56	2.03	1.81
2297	3.24	4.26	5.97	6.97	6.42	5.73	5.04	4.34	3.72	3.12	2.6	1.98	1.69
2298	3.24	4.17	5.87	6.98	6.43	5.74	5.06	4.37	3.79	3.2	2.64	2.1	1.34
2299	3.24	4.65	6.75	7	6.64	6.21	5.78	5.35	4.97	4.59	4.2	3.82	3.44
2300	3.24	4.66	6.76	7	6.64	6.21	5.78	5.35	4.96	4.58	4.2	3.84	3.46
2301	3.24	4.61	6.73	7	6.64	6.21	5.78	5.35	4.98	4.61	4.21	3.84	3.46
2302	3.24	4.63	6.74	7	6.64	6.21	5.78	5.35	4.98	4.61	4.22	3.84	3.41
2303	3.24	4.6	6.71	7	6.64	6.21	5.78	5.36	4.98	4.61	4.23	3.86	3.53
2304	3.24	4.65	6.75	7	6.64	6.21	5.79	5.35	4.98	4.61	4.21	3.85	3.46
2305	3.24	4.24	5.97	7.02	6.69	6.26	5.83	5.4	5.02	4.65	4.28	3.88	3.51
2306	3.24	4.24	5.97	7.02	6.69	6.26	5.83	5.4	5.02	4.64	4.27	3.9	3.56
2307	3.24	4.18	5.91	7.02	6.69	6.26	5.84	5.41	5.05	4.67	4.3	3.93	3.55
2308	3.24	4.2	5.92	7.02	6.69	6.26	5.83	5.41	5.04	4.66	4.28	3.91	3.55
2309	3.24	4.22	5.95	7.02	6.69	6.26	5.84	5.41	5.03	4.66	4.3	3.93	3.55
2310	3.24	4.26	5.99	7.02	6.69	6.26	5.83	5.41	5.04	4.67	4.3	3.95	3.59
