# Supplementary material for: Development of an Agent-Based Model (ABM) to Simulate the Immune System and Integration of a Regression Method to Estimate the Key ABM Parameters by Fitting the Experimental Data
Source: PLoS One. 2015 Nov 4;10(11):e0141295. doi: 10.1371/journal.pone.0141295 (PMC4633145; doi:10.1371/journal.pone.0141295)
Supplement: S10 Table — (PDF) [file pone.0141295.s011.pdf]

S10 Table. Sample size 5x3 with noise $\sqrt{0.75}$

samples	time points					
	0	1	2	3	4	5
1	2.56	6.76	7.91	8.69	6.48	5.55
2	2.31	6.48	8.1	8.59	5.87	7.8
3	4.01	5.51	7.09	9.15	6.32	7.35
4	2.85	5.43	8.74	7.12	7.28	6.03
5	3.54	4.9	6.01	7.22	6.45	7.64
6	2.56	6.9	7	6.49	6.63	8.32
7	3.86	6.97	8.41	8.85	7.95	7.15
8	2.93	7.15	5.89	7.79	8.06	6.82
9	2.89	4.99	6.78	5.74	7.02	6.14
10	4.02	5.22	6.98	7.84	6.44	6.32
11	4.39	6.15	10	7.01	6.61	7.39
12	3.82	6.39	7.34	7.33	7.35	7.5
13	3.8	7.4	7.19	6.38	6.63	6.67
14	1.87	5.65	7.89	6.47	8.28	7.12
15	1.72	6.57	7.03	7.61	5.99	7.57
16	3.29	6.05	6.71	7.95	6.11	6.26
17	3.26	7.73	6.65	6.13	6.46	7.53
18	3.46	5.13	6.5	8.89	7.62	7.12
19	2.93	6.36	6.73	7.78	7.03	5.77
20	1.42	7.1	7.22	7.09	6.54	6.83
21	3.23	5.33	7.65	8.52	6.15	6.79
22	3.6	5.12	5.76	6.28	6.67	7.22
23	1.14	6.69	5.88	6.66	6.65	6.14
24	2.01	7.1	7.25	7.95	7.36	6.72
25	1.44	5.86	6.28	7.77	6.7	6.38
26	3.35	5.18	6.4	6.45	6.55	7.29
27	2.93	6.35	6.58	8.25	6.41	8.99
28	2.77	6.01	7.49	7.77	6.59	4.95
29	4.89	6.77	6.62	6.66	5.76	6.96
30	3.13	6.28	6.56	5.55	7.52	6.52
31	3.44	5.97	7.47	7.74	6.01	8.25
32	3.21	4.66	8.4	7.81	7.23	6.71
33	3.37	4.88	6.47	7.93	7.5	5.25
34	3.39	5.81	7.44	7.21	7.23	8.67
35	1.97	7.06	8.28	7.01	5.5	5.9
36	3.79	4.85	7.46	7.2	7.23	8.36
37	3.17	6.77	7.26	9.88	7.93	6.03
38	2.29	7	8.85	7.58	7.06	5.63
39	3.85	5.78	7.26	7.79	5.32	6.95
40	1.77	6.77	6.79	6.84	6.08	6.5
41	2.51	6.05	6.64	6.3	7.69	6.17
42	3.5	5.89	5.67	7.19	7.5	7.35
43	2.63	6.02	7.29	6.85	7.41	7.91
44	4.2	4.71	6.56	6.88	6.02	7.03
45	4.31	5.74	7.4	6.46	7.66	5.8

46	3.18	6.5	8.01	6.86	6.14	6.3
47	3.75	7.82	6.55	6.75	6.38	6.65
48	2.88	6.24	8.67	6.98	7.66	7.86
49	4.03	7.49	7.49	5.93	7.88	5.86
50	2.97	5.81	8.38	6.41	7.37	8.07
51	2.72	5.54	7.27	6.86	6.25	7.2
52	3.6	7.01	8.37	6.92	7.64	5.53
53	2.45	4.77	6.77	7.79	7.81	7.67
54	4.81	5.73	6.19	4.87	7.14	7.17
55	3.4	5.62	8.42	6.17	9.19	6.93
56	3	7.01	7.18	8.17	8.36	5.71
57	3.91	6.31	8.12	5.55	7.93	7.2
58	4.7	4.25	7.15	6.98	7.14	9.05
59	3.59	7.42	6.4	5.92	6.63	6.1
60	2.87	6.58	7.15	6.23	8.16	6.12
61	4.32	6.56	6.01	7.85	7.92	7.29
62	3.05	6.45	8.56	7.7	7.2	6.01
63	2.62	5.31	7.08	7.25	7.38	7.54
64	3.46	7.11	7.44	5.29	5.56	7.53
65	4.13	5.87	8.66	5.84	6.49	6.57
66	2.71	5.71	6.5	7.76	6.92	5.05
67	3.72	6.33	8.54	7.27	6.45	7.99
68	2.57	5.69	6.71	6.46	7.26	6.93
69	1.65	6.48	6.45	7.06	6.54	7.05
70	3.65	5.37	7.56	7.02	6.44	6.79
71	4.05	6.69	6.66	6.68	7.7	6.68
72	4.29	5.03	7.6	7.31	7.65	7.25
73	3.96	5.39	8.45	7.27	7.7	6.25
74	4.3	6.06	5.89	7.08	6.03	5.46
75	3.24	6.39	5.43	8.4	7.55	7.27
76	3.38	8.08	7.02	7.08	6.92	6.83
77	3.09	6.27	6.02	6.95	7.12	5.5
78	3.42	5.68	7.77	5.49	7.47	6.32
79	2.83	6.06	7.35	6.81	5.5	7.57
80	1.79	5.82	6.9	7.17	6.51	6.72
81	2.82	5.75	5.79	7.16	7.28	6.1
82	4.26	5.84	8.68	6.09	4.91	5.95
83	1.75	6.44	8.18	6.75	6.82	6.56
84	4.15	7.57	6.2	8.08	6.87	6.93
85	4.93	5.16	7.56	6.4	6.2	6.4
86	3.74	7	8.19	5.73	6.82	8.82
87	3.11	5.32	6.38	7.71	7.89	6.28
88	3.78	5.24	8.7	7.61	7.43	6.99
89	3.79	4.32	7.7	6.68	5.48	7.43
90	3.32	5.06	6.9	7.9	6.7	6.47
91	2.02	5.7	7.44	6.77	6.42	6.33
92	4.08	6.22	7.78	6.58	8.62	8.17
93	3.07	7.42	7.86	6.12	4.57	6.18
94	2.74	6.24	5.89	7.8	6.47	6.94
95	3.87	7.14	7.54	6.21	7.82	6.68
96	2.8	6.36	7.12	6.23	7.98	6.81
97	3.48	4.6	6.76	6.87	6.66	9.24
98	2.7	7.29	9.85	7.17	6.14	5.5
99	3.54	5.16	8.4	6.42	6.79	6.71

100	2.99	6.69	7.63	6.81	5.75	6.62
101	4.39	5.72	6.04	7.49	7.19	5.87
102	4.73	6.34	7.43	6.84	5.78	7.16
103	4.08	5.6	8.19	8.1	6.16	7.19
104	2.47	6.46	7.37	8.7	7.96	6.33
105	4.15	6.31	6.14	7.09	6.35	7.22
106	3.26	5.14	7.87	7.63	7.03	7.8
107	2.45	5.56	7.32	7.06	7.37	6.75
108	4.18	7.68	7.72	7.13	8.31	6.15
109	4.64	6.66	8.95	6.1	7.11	7.57
110	4.88	4.6	7.23	8.31	7.93	6.11
111	2.82	4.11	5.16	6.73	7.32	6.74
112	5.08	6.08	7.65	7.18	6.09	6.61
113	3.03	4.34	7.37	6.86	7.44	5.08
114	2.06	7.42	7.09	6.3	6.36	8.45
115	3.48	5.49	7.5	6.96	7.39	7.42
116	2.53	6.11	7.29	7.18	6.68	7.06
117	3.74	6.17	7.37	6.61	6.87	5.15
118	3.95	7.48	8.13	6.67	7.92	6.68
119	4.13	5.91	7.68	7.42	5.06	7.33
120	2.69	6.22	6.48	8.64	7.39	6.05
121	3.81	6.26	7.99	6.34	6.32	5.93
122	2.68	6.84	8.73	8.21	6.21	6.26
123	3.05	5.66	8.15	6.49	7.29	6.67
124	3.81	5.73	7.68	6.32	6.29	6.47
125	3.14	6.92	7.39	7.22	7.27	5.75
126	3.13	6.61	6.79	6.46	7.47	6.41
127	3.15	5.71	7.99	7.95	6.73	5.81
128	3.63	5.71	8.44	7.71	6.23	6.38
129	2.56	6.84	6.18	7.76	5.73	6.14
130	3.56	5.1	8.17	7.93	6.25	6.66
131	3.36	6.29	5.86	5.71	6.96	6.79
132	1.13	5.83	7.03	6.69	7.24	7.11
133	4.02	6.11	7.62	6.65	5.53	7.45
134	2.37	6.02	9.05	8.49	7.92	6.02
135	3.73	6.34	7.81	7.78	5.58	6.84
136	3.1	7.48	6.12	6.21	6.58	7.48
137	3.49	6.35	7.71	6.72	7.69	6.04
138	3.56	5.88	7.44	7.16	6.61	4.63
139	2.83	5.69	7.18	6.22	6.36	6.23
140	3.27	6.25	7.07	7.88	7.72	5.54
141	4.4	6.19	7.66	7.34	6.04	7.15
142	3.92	4.89	7.81	7.34	7.28	7.35
143	3.41	7.77	8.07	5.48	7.73	6.85
144	2.23	4.53	7.96	6.34	7.44	7.35
145	3.46	6.8	7.77	5.83	7.61	7.8
146	2.29	6.33	6.29	6.44	7.13	7.46
147	3.35	5.78	6.75	7.39	7.08	6.96
148	2.35	6.79	8.86	8.1	6.78	5.65
149	2.68	5.97	7.95	6.59	7.3	6.95
150	3.17	6.59	6.24	7.03	6.64	5.9
151	4.06	6.64	7.4	8.07	8.83	7.93
152	4	6.34	5.84	6.88	5.8	6.31
153	3.79	7.46	4.98	6.04	7.19	8.94

154	1.39	5.94	7.08	8.38	7.22	6.31
155	1.99	5.41	8.31	7.68	6.67	5.45
156	4.4	5.62	7.68	7.61	7.79	6.81
157	5.02	6.21	7.93	7.07	5.62	6.18
158	2.91	6.53	6.36	7.1	8.08	6.47
159	3.05	5.98	8.46	8.33	6.88	5.73
160	3.78	7.05	6.28	6.01	7.05	6.87
161	3.42	5.31	7.62	6.97	6.61	8.14
162	5.49	6.28	7.19	6.67	6.35	5.37
163	3.1	6.4	6.59	7.05	6.71	6.42
164	3.77	3.84	8.02	8.33	6.58	7.67
165	2.71	5.7	6.52	6.49	5.35	7
166	5.01	5.96	7.21	7.91	7.85	6.32
167	2.67	7.35	7.39	6.58	8.44	7.71
168	3.77	6.39	6.54	7.16	7.2	7
169	3.03	6.68	7.71	6.79	7.8	5.89
170	2.78	5.86	7.63	6.79	7.48	7.17
171	3.04	5.94	7.74	6.72	7.46	6.46
172	5.09	5.58	7.03	6.61	7.81	5.85
173	3.77	4.59	7.45	6.48	8.47	6.46
174	3.44	5.96	6.62	6.28	6.34	7.78
175	2.98	6.43	7.3	7.37	6.63	6.83
176	3.51	6.08	7.17	6.11	7.73	7.53
177	1.65	6.82	7.52	7.62	5.6	6.86
178	3.84	4.45	6.17	7.49	6.64	7.05
179	2.54	6.7	6	6.6	6.9	7.5
180	3.4	6.57	7.07	9.69	6.18	6.3
181	3.72	5.87	7.55	8.22	8.15	6.28
182	2.89	6.35	7.98	7.47	7.98	8.45
183	2.59	6.92	7.24	8.23	7.43	7.12
184	2.8	6.81	7.3	6.94	7.57	8.36
185	2.57	7.26	6.28	6.68	6.26	7.02
186	3.03	6.95	7.24	6.84	6.59	6.97
187	1.64	6.53	6.17	6.06	6.99	6.87
188	2.71	6.42	7.98	7.43	7.47	6.61
189	3.13	6.22	6.12	7.43	5.97	7.02
190	3.4	6.94	6.22	6.63	7.8	7.63
191	2.07	6.58	8.1	7.85	7.56	6.61
192	2.26	5.9	7.48	5.76	7.33	6.64
193	3.68	4.91	6.86	5.96	6.33	5.27
194	4.7	5.07	5.73	7.34	6.72	8.34
195	3.33	7.28	9.89	7.12	6.56	8.1
196	3.69	5.19	8.63	7.93	6.72	7.52
197	2.84	6.18	5.77	7.82	6.88	7.92
198	3.63	5.98	6.59	7.99	6.01	6.24
199	3.6	6.74	6.76	6.65	6.96	6.93
200	3.73	6.43	7.89	7.95	7.41	6.31
201	4.2	7.03	8.1	6.68	6.31	6.62
202	4.24	6.04	7.87	6.77	7.57	6.89
203	4.1	5.07	8.54	7.44	6.68	5.54
204	3.6	6.68	6.86	7	7.73	6.74
205	3.69	6.93	8.59	7.78	7.28	6.14
206	1.88	6.33	8.06	7.66	7.39	6.8
207	3.19	7.33	7.89	7.3	7.16	6.86

208	5.28	7.02	6.66	8.43	5.88	6.94
209	3.26	6.02	8.14	8.52	8.12	6.68
210	2.63	4.31	7.82	7.72	5.93	6.59
211	3.53	6.28	7.37	6.54	6.67	8.5
212	4.89	6.26	7.34	6.57	7.03	7.13
213	3.8	6.75	8.02	6	6.68	6.59
214	4.94	6.33	7.93	6.77	7.46	7.61
215	4.87	7.08	6.45	7.81	6.24	6.15
216	3.28	5.98	6.28	6.16	5.22	6.68
217	3.81	5.67	7.2	8.01	6.38	6.56
218	1.43	6.55	6.09	7.2	6.3	6.41
219	4.04	5.28	7.64	6.94	7.25	5.91
220	1.16	5.7	7.55	7.05	9.24	6.34
221	2.74	6.08	7.59	7.09	5.61	7.65
222	3.16	5.67	5.81	7.11	8.37	8.17
223	3.2	6.05	8.54	6.01	5.77	7.1
224	4.53	7.05	7.41	6.79	6.7	5.73
225	2.8	6.44	8.64	7.4	6.52	7
226	4	5.71	7.44	7.07	6.63	5.94
227	3.99	5.61	6.78	6.84	6.12	7.31
228	1.83	6.08	7.7	7.17	7.69	7.16
229	4.03	5.86	8.06	7.26	6.73	7.23
230	3.83	5.86	7.44	5.92	7.64	6.03
231	3.46	7.42	8.98	7.42	7.67	6.84
232	1.9	6.67	7.97	6.41	6.64	6.29
233	3.52	5.28	8.59	8.36	7.29	6.68
234	3.93	5.27	6.63	8.71	6.89	6.48
235	3.32	7.18	7.3	6.6	7.56	6.31
236	4.26	7.67	8.13	7.76	5.5	7.24
237	3.92	6.74	8.67	7.58	6.29	6.36
238	2.16	4.36	5.99	7.03	6.82	6.79
239	3.6	5.88	6.46	7.18	8.28	7.32
240	4.55	5.67	6.36	7.63	6.61	6.6
241	2.66	5.59	8	7.48	7.21	7.74
242	2.63	7.14	7.74	6.55	7.89	5.8
243	3.67	5.61	7.56	7.31	6.63	7.73
244	3.9	5.53	8.25	5.86	8.09	7.55
245	4.46	5.9	7.25	6.66	7.23	8.52
246	1.92	6.17	6.98	6.89	6.83	7.67
247	2.4	6.24	6.91	6.73	7.12	6.67
248	1.85	6.32	4.82	6.25	6.9	5.79
249	2.87	6.26	6.86	6.36	7	6.51
250	3.7	5.59	7.02	6.91	6.43	6.9
251	1.43	6.33	8.33	7.76	7.95	6.72
252	1.79	6	7.86	6.44	9.25	7.04
253	1.99	5.46	6.14	7.8	6.41	7.12
254	1.63	5.69	6.74	5.7	7.28	7.87
255	4.8	6.42	8.44	6.21	5.45	6.78
256	2.87	6.29	8.13	7.43	4.23	6.95
257	3.78	6.11	8.85	7.16	8.55	7.5
258	3.95	6.24	8.33	6.95	6.35	6.12
259	3.71	6.45	6.28	7.59	8.13	7.95
260	2.92	5.22	6.18	7.79	6.88	6.8
261	2.49	6.36	7.21	7.36	6.91	6.6

262	4.2	5.83	7.27	6.39	8.04	7.47
263	2.33	6.06	5.82	6.8	6.34	6.33
264	1.76	5.61	6.44	6.02	7.73	5.96
265	2.74	5.68	7.24	7.25	6.48	6.32
266	3.9	5.54	7.31	7.45	7.8	7.53
267	3.67	6.83	7.63	7.79	7.41	6.89
268	3.46	5.24	7.53	6.42	7.53	6.73
269	3.86	6.51	7.87	7.72	7.12	5.07
270	3.77	6.62	7.45	5.55	6.71	6.68
271	2.89	6.43	6.78	7.97	5.7	5.98
272	2.72	6.64	6.75	6.6	7.03	5.8
273	3.8	7.45	6.86	6.25	6.78	7.53
274	4.42	5.27	6.83	7.4	6.52	7.36
275	3.24	5.97	7.93	6.92	7.24	6.95
276	4.15	6.99	8.16	9.19	4.23	6.49
277	3.62	5.99	7.55	6.83	5.66	6.44
278	2.74	5.52	7.56	7.07	6.04	8.72
279	3.28	5.59	7.12	7.99	5.4	5.91
280	1.98	6.82	7.21	7.87	6.19	8.45
281	3.78	6.35	7.33	6.53	6.88	6.44
282	2.26	4.88	7.54	6.97	6.86	6.12
283	1.99	6.5	6.08	6.01	7.34	4.54
284	3.66	6.04	5.82	5.38	5.65	5.89
285	4.02	4.74	7.01	7.84	7.38	7.93
286	2.88	6.82	8.43	6.19	5.32	7.6
287	4.63	5.22	7.8	7.59	6.72	7.37
288	2.84	5.88	7.86	7.59	6.32	5.99
289	2.53	7.41	8.54	8.98	6.78	6.1
290	2.93	5.83	6.96	8.4	6.63	6.66
291	2.33	4.92	6.27	7.89	7.06	5.76
292	3.61	4.91	6.54	7.27	7.42	7.21
293	4	7.13	6.62	7.04	6.71	8
294	4.47	6.44	7.66	6.24	5.72	7.4
295	4.19	5.61	8.22	5.98	7.02	8.56
296	4.5	6.31	7.42	5.61	7.65	5.94
297	2.84	6.74	7.16	8.37	5.74	7.94
298	2.43	7.85	7.42	6.93	7.48	5.27
299	2.84	6.76	7.27	7.23	6.95	5.79
300	3.36	5.53	7.94	6.95	7.49	5.61
