# Supplementary material for: Development of an Agent-Based Model (ABM) to Simulate the Immune System and Integration of a Regression Method to Estimate the Key ABM Parameters by Fitting the Experimental Data
Source: PLoS One. 2015 Nov 4;10(11):e0141295. doi: 10.1371/journal.pone.0141295 (PMC4633145; doi:10.1371/journal.pone.0141295)
Supplement: S11 Table — (PDF) [file pone.0141295.s012.pdf]

S11 Table. Sample size 5x3 with noise $\sqrt{1.50}$

samples	time points					
	0	1	2	3	4	5
1	2.1	6.45	6.48	6.38	6.96	9.4
2	3.2	5.25	5.83	6.92	7.63	6.8
3	4.16	6.61	8.08	9.23	7.88	6.16
4	2.92	2.94	7.56	8.04	8.46	6.85
5	2.48	5.49	9.49	7.38	7.97	8.07
6	2.91	5.08	9.92	6.49	5.29	5.79
7	3.85	6.9	6.03	7.85	7.31	7.65
8	4.75	6.21	8.83	7.27	5.86	7.06
9	0.24	4.36	7.41	7.18	8.56	5.24
10	3.91	7.47	5.66	8.63	6.06	6.86
11	3.09	7.01	6.86	7.18	4.94	6.25
12	4.04	6.06	8.48	7.6	7.21	6.19
13	2.95	5.85	9.21	4.29	7.95	5.47
14	4.32	7.09	6.23	10.95	6.4	7.22
15	4.18	5.29	9.39	8.42	9.7	6.49
16	2.33	6.72	8.86	8.34	7.32	6.85
17	2.71	6.07	8.05	7.16	7.55	5.83
18	1.28	5.56	8.82	8.38	7.12	7.34
19	4.19	5.92	6.46	6.97	8.55	7.68
20	4.16	5.33	8.72	6.07	5.81	6.6
21	1.37	5.75	6.83	4.66	6.4	8.27
22	2.19	8.04	6.01	6.66	6.62	7.26
23	2.14	5.65	6.94	8.18	4.78	7.84
24	2.97	7.18	6.8	6.48	4.73	9.8
25	2.34	8.34	7.24	6.9	7.91	6.62
26	2.83	5.22	8.89	6.63	4.04	7.51
27	3.15	4.85	7.75	6.82	4.86	6.5
28	2.84	5.69	6.04	7.94	7.26	6.17
29	4.24	5.39	6.39	7.44	4.24	5.82
30	1.65	5.86	7.74	6.51	5	7
31	3.43	5	5.44	8.46	6.16	6.92
32	2.89	6.15	7.64	6.46	5.62	7.32
33	5.53	5.6	6.58	7.82	7.46	4.44
34	3.48	5.39	6.85	6.23	7.28	4.95
35	3.62	6.24	7.89	6.71	8.44	6.25
36	1.57	7.01	6.66	3.9	8.6	6.25
37	1.65	7.58	5.76	6.33	8.81	6.53
38	3.42	5.62	5.08	5.83	9.07	6.61
39	3.57	7.11	5.53	6.89	6.83	7.19
40	3.95	5.56	6.6	5.29	5.94	8.75
41	3.84	6.79	5.28	6.04	5.29	7.9
42	2.69	5.9	7.15	5.6	7.56	6.82
43	3.63	7.43	6.73	8.79	3.95	7.34
44	1	4.64	4.57	7.45	5.59	6.53
45	3.14	4.34	6.45	9.56	5.3	7.76
46	2.44	7.31	7.9	8.2	7.63	4.95

47	2.66	5.93	7.45	6.53	6.11	6.22
48	1.98	5.7	4.19	8.03	6.16	5
49	5.53	6.69	8.98	8.17	5.69	6.57
50	2.95	5.97	7.77	7.98	7.12	8.35
51	3.79	5.62	5.81	6.03	4.19	6.82
52	3.18	5.58	8.33	8.68	5.75	6.77
53	2.66	5.9	7.21	7.61	6.66	7.38
54	2.86	5.84	5.54	7.81	6.99	6.12
55	3.25	4.42	7.55	7.32	6.41	5.65
56	2.68	3.78	5.17	5.76	7.22	7.55
57	4.33	6.39	5.99	5.57	6.64	4.97
58	3.01	5.29	7.88	6.81	6.5	6.26
59	4.68	6.87	8.4	6.84	6.44	4.76
60	0.85	6.5	6.67	5.89	7.5	6.36
61	3.31	5.09	8.06	4.95	6.84	5.64
62	4.08	6.19	8.73	7.7	6.19	6.74
63	3.77	6.61	5.63	7.98	8.97	7.52
64	3.94	2.94	6.91	5.36	7.64	5.89
65	2.75	4.97	7.98	5.89	7.78	7.48
66	2.77	6.63	5.14	9.48	6.93	5.61
67	3.89	6.43	7.41	8.13	6.09	8.3
68	3.16	5.77	7.16	5.37	6.4	6.17
69	1.43	6.15	7.91	5.88	6.04	7.86
70	3.24	5.48	6.48	5.87	8.17	5.75
71	4.36	6.59	6.54	6.97	6.9	6.37
72	4.45	7.41	6.71	7.17	7.79	8.06
73	2.17	4.26	5.91	9.78	6.72	5.75
74	2.74	6.67	8.06	5.65	7.63	6.22
75	5.41	6.16	6.92	7	7.58	7.07
76	3.97	5.68	8.32	8.99	7.29	6.39
77	1.96	5.82	8.4	7.13	8.1	5.62
78	0.36	7.76	8.36	6.5	7.79	7.69
79	5.85	8.27	7.4	7.06	7.19	4.88
80	4.99	4.89	8.09	8.2	7.7	6
81	2.38	4.02	8.02	7.1	6.72	7.22
82	2.43	4.59	7.52	6.98	4.52	5.15
83	3.84	5.84	7.53	7.24	9.53	7.39
84	3.49	5.18	6.77	8.93	7.25	4.58
85	3.89	5.78	7.04	6.34	6.16	6.21
86	3.77	6.4	6.47	6.66	4.1	5.43
87	2.43	6.53	8.04	6.78	5.94	6.99
88	4.64	5.79	7.08	6.99	8.22	7.89
89	1.6	5.02	8.26	8.15	6.74	6.05
90	2.16	7.43	7.37	7.17	5.66	8.16
91	1.85	8.06	4.84	8.4	6.64	8.77
92	5.39	6.88	9.77	8.08	6.77	8.38
93	2.27	5.62	8.28	8.18	5.52	7.28
94	0.85	6.67	5.15	6.55	8.24	6.92
95	3.29	7.53	5.93	6.68	8.56	6.83
96	1.35	5.7	9.02	6.42	8.69	8.45
97	1.43	8.06	6.81	7.27	6.23	6.07
98	4	5.7	4.98	7.16	6.5	8.21
99	6.62	6.72	7.76	7.07	7.98	6.38
100	3.53	2.73	7.62	6.65	5.5	6.03

101	3.66	3.1	4.57	8.98	6.31	6.21
102	0.92	6.54	9.44	4.95	7.84	7.51
103	1.84	6.02	7.82	9.69	6.05	6.02
104	4.94	5.37	5.41	7.96	6.85	6.95
105	6.04	5.13	7.67	4.7	8.2	8.58
106	3.3	5.54	8.05	6.81	7.9	5.44
107	1.7	4.04	7.27	6.99	7.16	7.47
108	3.99	5.36	7.48	7.16	6.69	7.87
109	3.98	6.19	3.34	6.05	6.26	8.24
110	3.6	6.74	6.83	9.16	8.57	6.43
111	4.14	5.96	5.63	6.07	6.09	7.43
112	2.85	3.15	7.36	7.27	7.19	6.77
113	5.17	4.91	6.28	6.06	7.49	6.05
114	4.6	7.28	7.16	8.65	8.41	7.19
115	3.65	8.1	10.29	7.58	5.87	4.78
116	2.9	5.5	7.48	6.51	5.94	6.84
117	3.08	4.92	8.31	7.17	6.48	5.86
118	2.91	6.08	6.91	6.09	7.65	6.03
119	2.41	6.24	7.48	8.95	6.89	6.73
120	3.24	7.71	6.5	8.24	8.15	4.16
121	2.47	7.19	9.74	6.75	8.07	8.37
122	4.33	6.14	7.76	6.83	5.36	5.33
123	1.99	8.94	7.07	8.36	7.65	6.69
124	3.1	6.8	7.32	6.73	6.13	5.77
125	4.86	6.52	7.35	9.15	5.72	7.57
126	4.13	4.62	8.07	8.48	6.09	7
127	2.49	5.15	7.24	7.58	6.89	9.57
128	2.9	7.93	5.27	8.59	5.9	6.27
129	3.78	6.98	7.69	8.15	6.97	6.49
130	3.39	5.09	7.85	6.57	5.48	7.15
131	2.73	8.28	6.65	7.85	8.52	4.34
132	2.21	6.53	8.39	7.47	5.01	7.38
133	5.03	4.21	4.88	6.75	9.31	7.94
134	4.95	7.04	7.68	7.29	6.75	6.57
135	0.34	4.02	8.31	8.22	6.1	6.25
136	2.71	6.42	5.92	5.14	6.62	6.03
137	3.88	5.8	9.36	7.1	8.35	6.88
138	2.86	4.12	7.74	6.55	7.13	7.57
139	4.64	6.02	5.08	5.43	6.23	6.88
140	2.7	6.98	8.63	7.52	6.67	5.55
141	4.03	4.47	8	7.61	6.45	6.96
142	3.02	5.46	7.44	8.05	6.82	6.42
143	3.06	5.64	7.88	8.01	8.15	5.73
144	4.46	5.67	8.84	8.78	4.97	6.49
145	4.31	6.06	7.6	5.29	7.22	8.19
146	4.05	6.84	5.23	7.61	6.49	7.54
147	1.88	6.45	6.1	6.61	9.38	5.3
148	3.23	5	9.18	7.66	4.69	8.79
149	1.97	4.7	7.98	8.61	7.13	5.21
150	2.14	5.57	5.76	8.88	8.21	5.41
151	2.63	2.75	6.75	7.76	8.27	3.73
152	4.02	4.1	5.66	8.39	8.39	6.36
153	4.2	6.25	6.25	8.75	6.06	6.94
154	3.84	6.64	7.19	7.01	6.09	5.32

155	3.97	5.63	5.57	8.21	5.59	8.51
156	3.34	4.35	8.02	6.65	5.52	4.17
157	4.82	6.97	5.81	5.97	7.45	4.4
158	5.1	9.87	7.82	7.95	5.78	8.42
159	5.42	6.34	8.4	9.69	6.32	6.66
160	3.58	6.34	7.09	6.95	6.22	4.82
161	2.78	4.97	5.69	6.52	9.6	8.07
162	0.14	5.49	6	7.32	5.74	6.45
163	3.28	4.84	7.26	8.74	5.82	7.54
164	3.2	8.07	7.4	6.33	8.91	7.59
165	2.98	7.85	9.27	6.43	5.97	7.3
166	1.8	5.34	4.75	7.09	7.67	6.45
167	1	5.24	7.28	9.29	6.13	7.43
168	3.43	7.33	7.1	5.3	6.36	8.19
169	5.58	4.07	7.18	6.49	8.8	7.52
170	1.79	6.74	7.6	6.91	8.69	4.2
171	3.84	4.02	6.64	6.8	7.4	6.65
172	3.04	6.82	5.28	8.48	5.47	7.69
173	4.34	5.88	8.19	6.07	6	8.09
174	2.69	6.66	9.14	8.9	6.93	9
175	3.33	6.55	7.02	7.24	7.82	7.33
176	3.59	4.9	5.2	6.1	7.78	6
177	2.71	4.99	6.74	5.04	7.75	8.15
178	0.17	8.58	6.05	8.43	4.45	9.07
179	1.24	6.34	8.52	6.31	8.36	8.59
180	3.98	6.87	7.59	7.92	8.03	9.94
181	2.99	6.99	8.01	6.64	5.1	7.78
182	3.38	5.2	5.17	7.75	4.37	8.34
183	0.82	5.04	6.38	8.87	4	4.75
184	5.02	4.54	6.3	5.84	8.05	5.59
185	3.04	6.47	7.07	7.84	7.57	6.93
186	1.97	7.33	7.36	9.3	7.2	7.71
187	4.85	4.51	8.68	5.74	9.68	5.6
188	2.13	5.32	5.96	6.48	6.91	5.7
189	5.08	3.79	7.46	8.02	4.32	7.17
190	2.1	6.6	7.05	7.56	6.57	5.99
191	5.87	6.71	3.77	6.69	6.84	5.12
192	3.22	5.31	8.79	7.35	7.19	6.62
193	2.71	5.87	4.78	6.65	7.94	5.3
194	4.78	6.76	6.84	7.33	7.32	8.5
195	1.88	5.39	7.09	6.21	7.36	7
196	6.1	5.99	7.05	7.41	7.87	8.31
197	2.23	7.08	7.53	6.03	8.23	7.45
198	5.73	7.45	6.32	7.79	6.82	5.72
199	4.29	6.2	9.12	5.81	5.81	7.31
200	3.09	4.83	8.82	7.27	7.21	5.16
201	2.68	6.43	9.96	5.44	5.89	6.68
202	3.12	6.18	5.18	9.51	5.72	8.68
203	3.14	8.71	5.02	6.01	5.64	6.53
204	3.17	7.69	7.4	6.99	7.01	4.71
205	3.11	8.14	7.8	10.69	7.95	5.72
206	2	8.54	8.67	5.21	3.77	5.53
207	5.07	5.7	6.55	7.48	5.62	8.57
208	2.91	6.33	6.22	7.76	8.37	6.72

209	1.27	4.79	7.51	6.68	8.55	5.28
210	4.18	6.47	8.3	5.05	5.87	8.03
211	2.21	7.71	7.28	8.22	8.98	7.39
212	2.02	4.81	8.51	7.65	6.16	7.12
213	1.72	7	5.36	5.2	9.92	8.22
214	5.83	5.9	7.13	8.7	6.42	5.24
215	1.81	5.93	8.4	6.72	6.67	7.65
216	0.86	7.22	9.09	7.38	6.31	9.62
217	5.08	6.89	7.16	7.23	5.98	6.89
218	1.5	5.2	5.35	6.97	6.94	8.82
219	2.74	3.63	7.79	7.64	7.97	4.83
220	1.35	7.21	8.14	8.92	4.63	7.69
221	1.15	5.84	6.88	6.74	8.91	7.63
222	3.46	6.77	6.27	3.98	6.02	5.27
223	4.5	5.38	5.23	4.9	7.24	6.26
224	3.77	4.91	7.66	8.27	6.24	9.04
225	1.21	8.13	8.52	7.49	8.47	6.5
226	1.09	6.79	5.75	6.22	7.25	6.86
227	3.87	7.53	9.1	6.73	5.71	7.68
228	3.35	5.03	7.68	6	6.43	7.36
229	5.92	5.34	10.76	5.33	8.24	3.73
230	5.89	7.7	9.47	6.49	8.28	7.23
231	2.42	7.42	6.64	6.27	6.72	7.09
232	1.51	4.71	8.5	5.64	6.28	6.07
233	4.8	6.68	6.44	6.46	5.62	6.45
234	2.73	7.58	6.19	5.5	6.08	3.75
235	3.6	7.58	6.27	4.46	6.73	7.86
236	2.05	5.66	5.52	6.13	7.19	6.61
237	2.93	6.23	6.55	7.31	6.25	7.13
238	1.8	8.77	5.64	8.44	6.05	4.73
239	2	5.83	7.04	5.85	5.29	9.21
240	3.54	6.98	5.38	6.04	5.86	8.44
241	2.55	8.16	5.97	7.5	8	9.53
242	2.25	6.37	6.44	7.47	6.04	7.2
243	2.22	5.56	7.84	6.29	5.51	7.52
244	3.53	2.98	8.18	7.67	8.06	7.09
245	2.52	5.49	7.54	5.97	8.1	8.03
246	3.34	6.66	8.7	6.08	6.46	6.72
247	2.03	4.96	9.31	9.28	7.43	5.49
248	1.32	7.77	9.02	7.39	9.7	7.37
249	4.52	6.83	5.86	5.5	6.14	6.23
250	1.34	5.66	5.32	6.7	6.14	7.05
251	0.76	3.82	6.74	6.71	3.03	5.74
252	2.81	6.86	6.83	7.95	6.31	6.21
253	3.68	6.35	6.9	7.81	8.83	7.52
254	2.76	4.66	5.56	7.5	6.66	7.99
255	3.28	5.64	5.68	8.22	9.55	8.97
256	2.53	5.37	5.69	6.82	4.94	5.17
257	4.03	7.23	7.11	7.58	8.43	6.84
258	3.48	7.43	7.99	7.31	6.83	6.29
259	1.02	6.8	8.33	7.47	7.08	5.91
260	2.19	6.27	5.51	5.9	8.37	7.56
261	3.89	5.89	5.73	4.75	5.75	4.45
262	3.58	4.31	8.07	5.26	6.85	5.87

263	4.06	7.1	6.16	6.84	8.45	6.37
264	4.28	6.42	5.27	6.68	5.92	8.03
265	2.79	7.56	6.3	7.46	6.87	4.53
266	3.63	4.24	8.02	7.25	9.12	4.95
267	3.03	4.41	8.92	6.74	6.17	7.04
268	2.66	7.68	6.44	6.97	7.35	7.1
269	2.41	6.04	7.63	5.52	5.7	6.75
270	4.28	5.72	9.09	9.67	5.45	9.11
271	1.75	5.79	5.25	8.55	7.92	8.48
272	3.17	4.34	5.87	6.9	6.46	8.8
273	3.46	4.91	7.17	5.3	5.45	6.28
274	3.59	5.91	8.41	8.29	5.67	7.09
275	2.4	7.02	4.82	6.83	5.42	8.14
276	4.61	4.67	7.5	7.57	6.25	6.7
277	1.83	5.92	8.76	5.67	9.08	6.23
278	4.47	4.23	6.62	6.82	6.75	4.2
279	3.32	8.51	8.04	9.08	6.74	5.74
280	2.7	4.91	6.43	4.44	7.84	9.6
281	2.96	5.77	8.29	6.93	7.2	5.55
282	2.76	5.11	5.36	8.84	8.06	4.12
283	2.99	4.46	7.07	7.03	8.96	5.77
284	3.7	7.01	7.33	6.34	7.44	8.27
285	2.07	6.33	8.69	5.31	6.57	7.43
286	3.49	5.45	6.06	6.45	9.05	6.68
287	1.46	6.37	9.07	7.9	7.3	6.46
288	2.19	8.03	6.87	6.37	7.24	6.68
289	2.31	7.26	7.47	8.23	5.51	7.07
290	4.5	5.49	8.96	6.36	6.07	6.1
291	2.66	5.96	6.7	10.37	6.04	7.28
292	4.17	4.79	5.45	3.65	6.42	7.47
293	5.16	5.36	7.21	5.78	6.63	7.45
294	2.05	4.95	4.58	6.88	8.44	4.73
295	4.22	7.21	7.14	6.93	7.1	6.19
296	3.57	8.79	7.13	7.56	6.55	7.67
297	6.22	6.95	5.7	7.22	6.09	6.44
298	5.41	4.55	9.04	7.53	6.46	7.29
299	4.55	7	6.76	7.51	7.46	5.74
300	2.01	7.75	6.47	6.02	7.01	6.35
