# Supplementary material for: Development of an Agent-Based Model (ABM) to Simulate the Immune System and Integration of a Regression Method to Estimate the Key ABM Parameters by Fitting the Experimental Data
Source: PLoS One. 2015 Nov 4;10(11):e0141295. doi: 10.1371/journal.pone.0141295 (PMC4633145; doi:10.1371/journal.pone.0141295)
Supplement: S12 Table — (PDF) [file pone.0141295.s013.pdf]

S12 Table. Sample size 5x3 with noise $\sqrt{3.00}$

samples	time points					
	0	1	2	3	4	5
1	4.22	8.1	11.29	6.3	7.15	7.41
2	6.5	4.91	5.41	6.66	7.57	7.66
3	2.45	7.32	6.16	3.52	5.38	7.25
4	1.84	4.56	6.91	4.79	5.65	6.34
5	8.41	6.9	6.91	5.32	8.93	6.45
6	2.21	5.13	7.3	9.24	9.96	7.63
7	6.99	3.73	6.05	8.96	8.43	7.64
8	2.14	6.46	7.94	10.27	10.54	6.15
9	2.8	6.11	7.2	2.33	4.73	6
10	0.31	6.67	6.52	7.38	6.22	8.84
11	3.67	6.89	4.03	7.05	6.17	7.14
12	1.37	2.97	7.42	6.61	7.66	10.59
13	2.48	5.26	8.53	8.37	8.28	6.7
14	3.37	11.43	5.45	4.96	8.96	6.2
15	3.02	6.46	6.95	6.23	4.24	2.4
16	1.47	4.46	7.18	6.43	6.4	9.09
17	6.83	7.37	3.69	8.74	6.18	4.5
18	3.82	6.73	7.68	4.3	8.71	6.65
19	1.54	7.45	5.23	7.6	7.74	3.05
20	0.75	4.4	8.4	9.7	6.83	6.81
21	3.3	5.41	6.59	6.3	9.7	3.93
22	4.09	6.52	7.89	6.02	7.1	8.67
23	3.27	4.46	3.36	7.92	9.24	6.94
24	2.88	7.48	9.89	7.49	3.19	8.21
25	3.97	5.97	6.18	6.97	8.38	5.94
26	5.41	8.06	7.22	7.65	7.35	7.77
27	1	5.36	8.52	4.57	6.28	8.64
28	3.46	5.87	9.14	3.87	8.82	5.17
29	4.91	5.67	8.31	6.66	9.32	6.03
30	1.37	6.48	6.39	4.97	5.16	5.62
31	2.57	6.44	7.52	6.93	3.48	8.1
32	2.31	5.55	4.67	8.73	7.17	7.98
33	1.66	4.81	7.2	6.61	3.81	5.36
34	1.56	10.25	6.55	5.05	6.46	8.66
35	1.71	4.2	8.06	5.34	7.1	8.74
36	2.51	8.23	6.46	4.45	6.19	7.72
37	0.01	4.78	6.9	6.92	6.5	6.55
38	0.57	2.73	7.69	9.96	7.01	7.48
39	1.57	3.76	6.25	6.26	4.18	5.09
40	3.69	5.2	5.98	7.79	6.42	6.8
41	1.65	1.79	3.91	8.18	8.49	7.95
42	0.31	2.33	11.07	6.16	12.47	7.74
43	6.81	5.58	7.52	7.12	6.48	8.85
44	2.83	5.46	8.45	6.59	3.28	4.98
45	3.46	4.1	7.97	4.52	9	7.51
46	4.64	4.54	6.56	8.31	7.48	6

47	4.7	7	7.65	4.44	7.46	8.74
48	4.56	4.83	8.19	10	5.82	4.93
49	2.91	5.27	5.9	6.29	7.23	6.52
50	4.24	5.66	11.34	6.96	8.42	4.74
51	1.83	7.32	8.43	7.41	9.51	7.24
52	2.77	7.03	6.26	5.56	7.61	7.74
53	2.16	5.56	7.45	10.03	9.34	7.27
54	1.18	6.34	6.2	7.58	8.44	6.88
55	3.1	4.15	8.58	8.14	6.46	4.59
56	4.84	4.53	5.4	8.49	7.98	6.78
57	2.35	5.99	8.24	7.03	8.7	6.09
58	2.31	0.81	8.38	9.93	5.57	8.16
59	4.59	6.46	4.07	8.67	9.43	6.34
60	1.24	8.86	9.3	7.83	5.72	7.36
61	3.57	3.74	5.36	1.22	6.66	8.21
62	3.76	4.44	5.73	9.33	8.74	9.19
63	2.01	6.66	8.65	7.99	4.55	6.61
64	9.11	6.62	6.69	11.09	5.61	5.38
65	2.97	7.08	4.55	4.96	7.04	7.86
66	0.78	7.59	8.5	3.88	5.83	3.97
67	3.58	5.91	8.04	7.11	6.55	8.04
68	3.74	7.06	7.5	9.24	6	8.39
69	4.42	4.18	6.36	8.09	8.1	6.63
70	3.92	4.93	10.11	5.91	10.34	8.99
71	3.38	7.37	5.28	6.09	4.79	7.98
72	4.2	5.22	6.52	8.03	5.13	6.4
73	2.16	5.64	6.91	5.72	6	5.82
74	2.69	5.44	6.52	5.83	4.06	6.94
75	4.6	3.86	7.64	7.25	5.93	3.91
76	4.56	4.62	5.12	7.83	6.84	4.95
77	3.67	2.81	8.46	7.51	6.69	5.43
78	3.57	7.46	5.52	8.74	5.76	7.59
79	4.52	4.74	7.22	6.95	7.25	5.47
80	5.29	4.38	6.57	7.52	5.79	8.28
81	5.74	7.26	7.31	8.9	8.46	6.79
82	3.5	9.12	3.85	4.63	6.11	6.69
83	4.45	8.11	7.81	5.15	5.95	8.71
84	1.65	7.39	9.37	5.58	9.37	7.71
85	2.98	8.5	6.61	5.17	6.1	7.44
86	3.98	4.25	5.43	8.05	7.3	3.35
87	4.51	6.96	4.06	8.95	8.46	5.68
88	3.27	8.34	8.81	8.99	5.99	6.89
89	3.38	7.85	9.31	8.88	6.44	6.63
90	0.64	5.55	5.62	8.27	5.56	6.58
91	1.14	3.39	7.89	5.47	7.91	4.76
92	2.12	6.21	7.77	7.27	3.74	2.61
93	5.23	4.05	5.22	5.08	6.11	7.69
94	3.39	7.39	6.99	9.49	9.08	6.74
95	2.06	5.53	6.3	7.56	7.81	7.95
96	3.72	9.34	7.48	8.08	5.38	10.32
97	4.03	6.09	6.8	6.32	9.29	5.08
98	4.66	4.05	4.19	3.77	8.7	7.94
99	3.31	6.77	7.37	10.09	7.48	7.9
100	1.25	7.06	4.02	8.75	8.04	5.68

101	4.07	3.39	9.84	4.2	7.51	4.1
102	4.56	5.07	7.74	10.28	7.47	3.17
103	1.43	4.28	5.46	7.19	9.3	8.8
104	2.96	2.23	6.81	6.04	8.77	7.35
105	1.81	6.76	5.73	9.34	8.62	7.75
106	3.55	6.37	3.65	6.25	8.15	5.64
107	0.58	6.88	7.38	4.47	8.23	5.59
108	5.28	6.66	7.47	6.73	7.95	6.11
109	4.39	4.34	4.91	6.26	6.54	8.43
110	4.67	6.05	4.17	7.69	7.25	4.97
111	3.33	5.46	6.87	5.38	7.34	7.86
112	2.28	6.88	5.99	6.59	14.39	10.7
113	2.54	7.34	7.95	5.34	7.49	7.32
114	2.05	6.66	5.78	12.17	8.55	6.78
115	2.58	8.68	5.68	9.89	6.94	8.12
116	2.37	5.11	7.23	5.53	7	6.41
117	4.22	8.9	6.86	8.58	6.95	4.85
118	3.31	3.82	9.1	4.28	7.41	6.51
119	2.82	7.95	7.93	9.12	7.14	5.79
120	1.02	6.07	5.25	6.55	5.38	7.61
121	1.76	8.88	9.33	6.89	6.59	7.12
122	3.97	7.19	5.64	8.45	6.28	5.54
123	3.81	6.46	7.39	6.25	8.66	6.42
124	1.25	3.8	3.72	7.71	8	3.93
125	1.57	7.5	6.48	6.29	10.2	8.41
126	2.81	5.15	4.78	6.29	7.01	7.45
127	2.23	9.02	5.88	7.09	5.92	8.19
128	4.08	2.68	9.69	8.57	7.87	4.05
129	4.55	3.97	4.53	6.75	5.66	8.91
130	5	5.88	10.18	8.35	7.15	5.68
131	3.42	3.14	8.78	8.79	6.74	6.95
132	2.06	3.44	8.5	8.49	7.8	4.6
133	4.03	6.5	4.16	10.11	11.5	8.13
134	3.84	6.05	5.22	8.49	4.82	6.72
135	1.45	3.99	7.82	6.35	6.35	5.53
136	6.52	7.57	6.09	10.36	6.57	5.55
137	2.84	7.96	7.3	7.66	9.46	6.02
138	2.14	9.07	9.46	5.42	8.31	8.05
139	5.39	5.67	6.88	11.98	9.5	6.82
140	2.74	5.27	7.12	4.93	4.58	7.95
141	0.77	4.05	8.38	5.9	8.42	7.44
142	0.1	7.27	8.69	9.2	9.87	6.01
143	3.05	4.69	5.46	7.42	5.1	6.36
144	2.34	6.08	8.51	2.09	8.1	8.18
145	2.46	8.7	4.45	6.51	5.87	8.36
146	2.21	2.4	5.92	8.91	8.48	8.4
147	1.69	6.77	5.7	7.43	11.58	5.74
148	5.97	7.78	5.67	4.4	9.07	5.6
149	4.03	7.33	7.23	6.89	7.28	6.17
150	3.99	9.4	9.87	8.36	7.24	7.38
151	4.11	5.33	7.53	5.16	6.16	6.21
152	5.31	4.72	5.6	8.77	6.54	8.29
153	3.5	6.35	8.78	6.38	8.95	5.99
154	4.37	8.32	9.19	7.07	5.97	7.09

155	4.04	6.31	9.02	9.24	4.52	7.42
156	0.65	8.06	5.14	8.6	10.41	8.38
157	1.84	9.64	9.03	7.11	5.97	5.87
158	3.74	8.88	8.06	6.15	6.15	11
159	1.15	4.8	9.06	6.77	9.09	5.1
160	1.53	3.59	4.17	7.19	5.57	10.74
161	0.39	7.14	7.87	8.4	6.72	7.01
162	2.68	5.14	7.78	5.89	6.49	7.42
163	4.65	5.83	5.92	7.81	6.71	5.92
164	1.65	3.97	5.78	5.89	6.22	8.95
165	1.62	6.51	7.84	6.16	4.53	9.39
166	4.66	7.28	6.06	9.34	7.57	4.88
167	1.76	7.8	3.66	6.24	8.81	5.93
168	0.63	5.19	8.26	7.76	6.47	6.16
169	7.23	7.32	5.71	6.74	7.57	3.28
170	2.91	5.08	7.29	6.46	11.29	7.99
171	3.62	8	8.87	7.78	6.29	3.55
172	5.78	4.01	4.03	6.46	2.89	3.8
173	0.01	6.52	5.95	7.11	7.32	5.7
174	1.62	10.35	8.35	9.4	7.48	6.81
175	2.69	1.96	6.66	7.78	4.79	6.96
176	3.91	5.97	5.65	8.12	4.58	8.47
177	4.62	5.35	2.98	7.71	9.57	5.39
178	6.08	6.91	3.98	8.8	8.58	6.59
179	0.53	4.52	9.28	4.53	5.64	8.34
180	1.74	8.42	7.27	11.2	7.25	8.42
181	3.71	7.09	7.74	7.59	8.26	6.52
182	3.25	6.96	7.4	8.29	7.64	8.16
183	4.12	6.17	7.63	8.01	8.2	5.03
184	2.65	6.22	6.68	7.66	4.91	4.02
185	5.51	3.51	6.89	5.59	5.04	9.34
186	1.41	5.87	8.04	6.53	8.84	7.04
187	6.35	6.23	5.87	6.89	4.98	5.48
188	4.17	4.16	3.79	4.57	7.21	8.89
189	2.28	5.18	8.6	7.06	4.91	5.87
190	0.17	4.75	8.81	7.47	3.47	4.85
191	1.4	6.1	4.81	8.13	7.59	6
192	1.47	4.71	5.78	6.34	6.76	8.96
193	4.97	6.51	6.23	9.96	7.52	8.5
194	5.26	7.13	5.13	6.75	7.11	6.8
195	3.22	7.09	4.79	4.32	7.81	6.16
196	6.66	3.41	4.93	6.91	4.39	7.79
197	2.13	5.86	4.94	9.77	7.84	1.26
198	3.32	5.34	8.1	5.11	9.06	7.44
199	3.99	3.26	8	10.53	9.34	6.91
200	3.46	5.82	8.74	9.74	8.47	5.51
201	4.98	7.92	5.62	9.43	5.81	9.61
202	3.03	5.45	4.54	3.72	8.03	6.42
203	0.88	8.49	8.3	4.89	6.79	4.23
204	2.71	6.6	12.58	8.95	7.14	8.41
205	4.39	5.35	5.4	2.99	7.59	6.71
206	5.87	3.5	4.88	6.25	4.76	5.69
207	1.03	7.73	6.8	6.74	5.46	6.73
208	4.63	4.42	7.18	9.34	5.94	5.75

209	1.85	4.65	6.9	7.27	4.69	7.85
210	3.34	3.27	8.5	8.17	7.44	7.8
211	1.41	5.35	6.46	8.55	7.52	5.78
212	2.56	6.74	2.79	7.75	7.48	8.11
213	7.17	6.41	5.96	7.93	8.05	5.87
214	6.5	2.43	5.49	5.83	6.58	8.06
215	6.43	7.86	7.41	11.82	5.19	7.08
216	3.97	6.93	7.99	5.42	5.06	3.8
217	3.68	6.3	8.82	5.79	5.59	7.69
218	1.47	8.3	6.19	7.05	5.79	4.99
219	2.61	4.3	6.09	6.3	5.94	5.99
220	3.09	6.97	6.4	6.47	6.29	5.83
221	3.97	5.99	7.4	6.01	10.4	8.46
222	4.46	5.89	7.81	6.94	7.97	5.43
223	0.84	8.11	4.03	9.08	6.75	6.21
224	4.95	4.8	8.52	8.75	7.05	5.28
225	3.68	7.15	5.85	3.82	6.64	7.13
226	2.88	7.52	7.76	3.94	5.2	6.98
227	5.16	4.17	7.36	6.75	8.01	7.71
228	4.7	6.71	5.59	4.18	6.08	8.08
229	5.84	8.5	8.12	7.24	5.32	7.07
230	2.99	10.29	4.92	5.78	3.57	5.65
231	5.18	4.34	10.89	6.38	4.81	5.13
232	1.09	3.43	5.96	8.15	7.81	6.49
233	0.65	6.04	7.54	8.82	8.17	3.2
234	1.75	7.68	2.8	6.36	6.18	5.1
235	2.08	3.86	5.25	4.57	6.84	6.94
236	2.08	6.4	6.19	10.11	9.91	6.14
237	2.27	5.25	6.95	5.84	8.81	4.75
238	1.36	6.43	6.53	7.48	6.76	5.01
239	3.78	5.94	8.17	5.71	8.1	9.21
240	1.88	8.76	7.65	9.19	6.28	10.33
241	3.98	6.54	4.79	8.74	9.12	5.6
242	1.85	6.23	9.74	8.2	5.31	8.69
243	3.57	5.08	10.01	7.37	7.27	5.78
244	5.57	5.14	3.94	6.04	4.16	6.29
245	6.23	5.58	8.18	9.64	6.99	6.39
246	2.65	5.74	11.31	5.66	3.26	6.88
247	5	5.21	5.41	8.11	7.95	3.49
248	4.01	4.65	10.32	8.26	7.04	5.59
249	2.97	3.42	6.34	7.28	9.34	5.83
250	4.85	4.78	8.58	9.28	6.14	7.34
251	2.52	5.15	7.75	11.87	8.16	7.81
252	3.63	5.27	4.46	7.31	9.28	5.61
253	3.72	5.89	9.62	6.3	9.28	6.94
254	4.87	4.53	6.17	5.02	5.69	7.12
255	2.96	4.63	9.48	6.42	7.14	4.59
256	2.97	6.89	5.58	7.46	6.72	5.26
257	4.82	6.43	5.64	6.39	7.51	3.57
258	1.92	6.48	7.5	7.62	6.2	7.68
259	2.46	6.97	4.89	10.34	5.92	5.96
260	2	4.2	9.15	5.41	6.52	7.12
261	4.48	9.6	7.31	4.87	11.59	6
262	2.19	4.07	3.69	7.31	6.9	9.73

263	2.14	6.85	10.16	7.95	7.37	7.28
264	3.18	7	6.86	5.75	7.11	6.77
265	5.44	2.85	5.72	5.13	7.74	5.97
266	3.69	8.96	8.39	6.88	5.17	10.42
267	2.58	4.61	9.98	5.35	5.68	5.97
268	3.55	7.19	5.95	7.69	5.57	3.32
269	3.61	6.55	10.1	6.69	9.16	9.68
270	1.94	7.37	8.38	6.04	7.48	4.8
271	3.52	6.17	9.21	7.27	9.44	5.21
272	2.28	9.09	8.83	9.03	5.76	4.79
273	1.96	5.78	7.47	6.39	8.86	6.24
274	3.57	2.41	7.89	7.64	6.2	5.72
275	2.69	3.67	7.58	2.01	8.28	7.69
276	3.04	6.89	6.15	6.49	8.82	5.87
277	4.13	5.08	5.75	8.16	9.01	8.73
278	1.79	9.78	8.09	8.3	7.64	6.06
279	1.32	8.04	7.82	6.34	6.95	4.02
280	2.68	5.47	6.1	7.78	5.62	8.15
281	4.31	5.2	5.92	6.51	8.86	8.09
282	4.68	4.89	6.88	0.93	8.34	9.54
283	2.24	4.29	8.16	4.99	6.9	8.16
284	0.67	8.16	6.79	6.98	8.42	4.91
285	2.46	7.41	8.27	8.34	7.39	7.07
286	1.02	8.96	6.1	5.96	10.34	9.31
287	0.58	9.9	9.67	4.85	6.73	5.79
288	4.5	4.6	8.46	7.9	5.48	7.12
289	1.76	3.65	5.1	9.7	5.41	8.14
290	1.62	5.56	5.84	7.44	6.85	6.93
291	7.17	2.12	6.56	6.62	5	4.98
292	3.05	5.67	8.33	8.8	7.49	5.23
293	4.1	3.46	6.12	8.68	7.08	3.29
294	2.47	6.08	7.62	7.13	8.43	7.02
295	4.18	10.35	9.12	6.2	6.37	6
296	3.45	7.71	9.56	5.67	5.99	5.51
297	5.89	8.39	7.6	6.59	5.26	3.18
298	3.85	7.56	8.79	7.22	7.54	8.93
299	2.29	7.71	8.1	5.88	5.26	4.22
300	3.48	5.78	5.87	9.13	7.15	7.93
